# Collection and Analysis of Adherence Information for Software as a Medical Device Clinical Trials: Systematic Review

**DOI:** 10.2196/46237

**Published:** 2023-11-15

**Authors:** Emily Grayek, Tamar Krishnamurti, Lydia Hu, Olivia Babich, Katherine Warren, Baruch Fischhoff

**Affiliations:** 1 Department of Engineering and Public Policy Carnegie Mellon University Pittsburgh, PA United States; 2 Division of General Internal Medicine University of Pittsburgh Pittsburgh, PA United States; 3 University of Pittsburgh School of Medicine Pittsburgh, PA United States; 4 Department of Mechanical Engineering Carnegie Mellon University Pittsburgh, PA United States; 5 Department of Engineering and Public Policy Institute for Politics and Strategy Carnegie Mellon University Pittsburgh, PA United States

**Keywords:** mobile health, mHealth, adherence, evaluation, usability, efficacy, systematic review, application, compliance, safety, effectiveness, engagement, risk, medical device, clinical trials

## Abstract

**Background:**

The rapid growth of digital health apps has necessitated new regulatory approaches to ensure compliance with safety and effectiveness standards. Nonadherence and heterogeneous user engagement with digital health apps can lead to trial estimates that overestimate or underestimate an app’s effectiveness. However, there are no current standards for how researchers should measure adherence or address the risk of bias imposed by nonadherence through efficacy analyses.

**Objective:**

This systematic review aims to address 2 critical questions regarding clinical trials of software as a medical device (SaMD) apps: How well do researchers report adherence and engagement metrics for studies of effectiveness and efficacy? and What efficacy analyses do researchers use to account for nonadherence and how appropriate are their methods?

**Methods:**

We searched the Food and Drug Administration’s registration database for registrations of repeated-use, patient-facing SaMD therapeutics. For each such registration, we searched ClinicalTrials.gov, company websites, and MEDLINE for the corresponding clinical trial and study articles through March 2022. Adherence and engagement data were summarized for each of the 24 identified articles, corresponding to 10 SaMD therapeutics. Each article was analyzed with a framework developed using the Cochrane risk-of-bias questions to estimate the potential effects of imperfect adherence on SaMD effectiveness. This review, funded by the Richard King Mellon Foundation, is registered on the Open Science Framework.

**Results:**

We found that although most articles (23/24, 96%) reported collecting information about SaMD therapeutic engagement, of the 20 articles for apps with prescribed use, only 9 (45%) reported adherence information across all aspects of prescribed use: 15 (75%) reported metrics for the *initiation* of therapeutic use, 16 (80%) reported metrics reporting adherence between the initiation and discontinuation of the therapeutic (*implementation*), and 4 (20%) reported the discontinuation of the therapeutic (*persistence*). The articles varied in the reported metrics. For trials that reported adherence or engagement, there were 4 definitions of initiation, 8 definitions of implementation, and 4 definitions of persistence. All articles studying a therapeutic with a prescribed use reported effectiveness estimates that might have been affected by nonadherence; only a few (2/20, 10%) used methods appropriate to evaluate efficacy.

**Conclusions:**

This review identifies 5 areas for improving future SaMD trials and studies: use consistent metrics for reporting adherence, use reliable adherence metrics, preregister analyses for observational studies, use less biased efficacy analysis methods, and fully report statistical methods and assumptions.

## Introduction

### Background

There are over 350,000 health-related apps on the market, each claiming to improve certain aspects of physical or mental health [1]. A small fraction of these apps is subject to Food and Drug Administration (FDA) regulations. Regulators, health care providers, and patients need to understand how these apps compare with alternatives (eg, pharmaceuticals) that undergo rigorous evaluation. As with pharmaceuticals, the risks and benefits of apps depend on how well people use them. Incorrect assumptions about adherence in clinical trials can lead to incorrect regulatory and treatment decisions. With pharmaceuticals, these risks are reduced by the gold standard practice of intent-to-treat analysis, which estimates effectiveness based on actual, typically imperfect, use. This standard is not the norm in trials of digital health apps, leading to an unknown risk of bias (ROB) in the estimated effects. Here, we provide a systematic review of current practices in FDA-regulated apps, leading to recommendations for reducing the risks of bias revealed by the review.

The FDA focuses on the regulation of software as a medical device (SaMD) therapeutics intended to prevent, diagnose, or treat diseases [2]. If a predicate therapeutic exists, applicants may use the FDA’s 510k pathway to prove that their therapeutic is substantially equivalent to the predicate therapeutic (ie, with the same intended use, technological characteristics, and benefits and risks of an approved or cleared therapeutic [3]). In the absence of a predicate therapeutic, SaMD therapeutics follow the FDA’s De Novo pathway, which requires evidence that the therapeutic is safe and effective. The FDA established the Digital Health Center of Excellence to create innovative ways to regulate SaMDs [4], which, for example, are easier to update than pharmaceuticals. One such innovation, reviewed under the FDA’s precertification pilot program, conducted *excellence appraisals* of software companies. This program tested a streamlined approach to approving and updating therapeutics for companies that have demonstrated quality practices [5,6]. Other innovations have been applied across all FDA departments, such as allowing clearance, approval, and marketing claims based on “real-world evidence” [7]. There are also proposals, created outside FDA, specifying standard processes (eg, performance reporting standards) for clinical trials of low-risk digital health apps not subject to regulatory oversight [8]. Given the novelty of SaMDs and the associated regulatory environment, the FDA has the need and opportunity to create guidance and requirements for addressing adherence in future trials. We hope to inform that process.

A systematic review by Milne-Ives et al [9] found that approximately three-fourths of digital health app trials collected and reported basic adherence information, such as the number of dropouts. These trials reported a variety of app engagement metrics, with only one-third reporting >60% use. Prior systematic reviews of digital health apps reported similar simple summary statistics (eg, average adherence and dropout rates), with few details on how adherence data were collected and analyzed [9-14]. This systematic review extends that work by examining, in detail, how adherence and engagement information is collected, analyzed, and reported. It considers how those practices affect the estimates of *effectiveness* and *efficacy*, defined as the app’s effect in the entire sample, regardless of adherence, and the app’s effect in the adherent subgroup, reflecting the moderating effect of adherence. This review focuses on digital health apps with a reasonably well-defined evidentiary base, namely, those that followed the FDA’s De Novo or 510k pathways.

### Criteria for Evaluation

#### ROB Framework

Imperfect adherence can cause underestimation or overestimation of the safety and efficacy of a SaMD. For example, a therapeutic’s efficacy and side effects may be underestimated, if trial participants use it sparingly, but consistent use is assumed. Conversely, efficacy may be overestimated if adherence reflects neglected confounding variables (eg, income and lifestyle factors). As a hypothetical example, researchers evaluating an app to reduce the risk of preeclampsia may observe a reduced rate not because of participant adherence but because participants adhering to the app were recipients of commercial health insurance. To evaluate the ROB owing to imperfect adherence, we used the adherence components of the Cochrane ROB Assessment (version 2.0) [15], a well-documented tool for systematic reviews and meta-analyses. To determine the ROB from nonadherence, the ROB tool first asks, “Was there nonadherence to the assigned intervention regimen that could have affected participants’ outcomes?” If outcomes could have been affected, the ROB tool then asks, “Was an appropriate analysis used to estimate the effect of adhering to the intervention?” We developed criteria to answer each question based on research regarding adherence metrics and common methods of analyzing efficacy.

#### Adherence and Engagement Metrics

Adherence refers to how well participants use an intervention, as defined by a protocol or recommendation for use. Engagement refers to how participants use an intervention, irrespective of the intended use of the app. Engagement data can be used to measure adherence for a digital health app. As both adherence and engagement can affect the outcomes of a trial, we have reported both. When collecting and reporting adherence and engagement statistics, researchers must consider 3 facets of use [16]: *initiation*, when a person starts using an intervention; *implementation*, how a person uses the intervention between initiation and discontinuation; and *persistence*, how long a person uses the intervention before discontinuation.

Which metrics are collected and how they are collected can also affect the ability to conduct efficacy analyses and the analyses’ potential bias. For instance, adherence with recommendations from the therapeutic (eg, using backup contraception when an app detects fertility) could also affect effectiveness estimates. Without collecting this information, researchers would be unable to analyze efficacy in terms of adherence to behavioral recommendations. Therefore, we report adherence and engagement with both the therapeutic and its recommendations. The mechanism of collecting adherence and engagement information can act as a potential confounder if it prompts additional engagement with the therapeutic compared with real-world engagement. Reminders used to increase adherence (eg, email messages) can also be confounders if they are not part of the therapeutic design. To account for these potential confounders, we recorded whether reminders and mechanisms for measuring adherence and engagement were internal to the app or external (ie, an additional component not found in the marketed app). We found few prior studies or analysis plans that determined the level of adherence or engagement required to have a clinical effect. This level of adherence can vary depending on the therapeutic being used. Without a study or trial analysis plan defining low adherence or evidence of the level of adherence needed to produce a clinical effect, we cannot conclusively assess whether adherence is low or not because of insufficient information.

#### Analysis of Efficacy

In evaluating efficacy analyses, we ask how well a trial or study fulfills the assumptions required by its efficacy analysis method. There are 3 commonly used estimates of efficacy: the *average treatment effect* (ATE), *per-protocol effect*, and *dose-response effect*. Table 1 describes each estimate, the common analysis methods for calculating estimates, and the assumptions required for unbiased estimates. Multimedia Appendix 1 [17-22] includes definitions of the following assumptions: consistency, positivity, ignorability, exclusion restriction, strong monotonicity, and the stable unit treatment value assumption (SUTVA). In addition to the requirements in Table 1, researchers should preregister their analyses of effectiveness and efficacy to reduce the risk of capitalization on chance [23].

**Table 1:** Methods of analysis commonly used to account for imperfect adherence and the assumptions required for unbiased estimates.

Estimate of efficacy and common analysis methods		Assumptions for unbiased estimates
**ATE^a^: estimates the average effect of treatment**		
	ATE analysis Evaluates groups according to their treatment group regardless of adherence. Estimates efficacy if adherence is modified with regular reminders to participants.	SUTVA^b^ Consistency^c^ Positivity Ignorability
	ITT^d^ analysis Evaluates groups according to their assigned treatment regardless of adherence. Estimates efficacy if adherence is modified with regular reminders to participants.	SUTVA Consistency^c^ Randomization (fulfills positivity, exclusion restriction, and ignorability)
**Per-protocol effect: estimates the average effect of adhering to the treatment assignment**		
	Complier average causal effect or local average treatment effect Evaluates the per-protocol effect for the adherent subpopulation. Evaluates groups based on an adherence threshold. Nonadherent participants in the treatment group are labeled as never-takers. It is assumed that the effect of the never-takers is equal in both groups.	SUTVA Consistency^c,e^ Randomization (fulfills positivity, ignorability, exclusion restriction, and strong monotonicity)
	Generalized estimation Evaluates groups based on an adherence threshold. Groups are evaluated based on adherence over time such as never-takers, early-takers, late-takers, and always-takers.	SUTVA Consistency^c,e^ Positivity Ignorability (sequential exchangeability)
	As-treated analysis Evaluates groups based on an adherence threshold. Nonadherent participants in the treatment group are considered part of the control group.	SUTVA Consistency^c,e^ Positivity Ignorability (conditional independence of adherence and outcomes)
	Per-protocol analysis Evaluates groups based on an adherence threshold. Excludes nonadherent participants in the treatment group.	SUTVA Consistency^c,e^ Positivity Ignorability (conditional independence of adherence and outcomes)
**Dose-response effect: estimates the effect of adherence on the treatment**		
	Dose-response analysis (IV^f^ method) Evaluates adherence as a mediator for all participants using an IV to fulfill the mechanism ignorability assumption.	SUTVA Consistency^c,e^ Randomization (fulfills positivity, ignorability, exclusion restriction, and strong monotonicity)
	Dose-response analysis (confounder adjustment) Evaluates adherence as a mediator for all participants using confounder adjustment to fulfill the mechanism ignorability assumption.	SUTVA Consistency^c,e^ Positivity Ignorability (conditional independence of adherence and outcomes)

^a^ATE: average treatment effect.

^b^SUTVA: stable unit treatment value assumption.

^c^Consistent definition of treatment.

^d^ITT: intent-to-treat.

^e^Consistent definition of adherence.

^f^IV instrumental variable.

We applied our framework, which was developed based on the Cochrane ROB, to evaluate how well existing trials and studies meet our standards, with the goal of improving future trials. We examined the completeness of their reporting and the appropriateness of the procedures reported. By focusing on SaMD therapeutics, the most rigorously evaluated digital health apps, we sought to identify improvements for future studies on all digital health apps.

## Methods

### Screening

A 2-stage search strategy was used to identify all product codes and registrations for patient-facing SaMDs, with intended repeated use for at least 2 weeks, that the FDA had approved or cleared before March 2022. In the first stage, 2 reviewers independently searched the FDA product code database for product codes related to SaMDs. We searched the device name, definition, physical state, and technical method attributes for the keywords “software,” “mobile,” “digital,” and “application.” In the second stage, we searched the FDA registration database for these product codes. We examined each registration’s supporting documents, De Novo decision summaries, and 510k decision summaries to determine whether the product met our inclusion criteria.

We then searched ClinicalTrials.gov, product websites, and MEDLINE for peer-reviewed publications corresponding to each included product. For the ClinicalTrials.gov search, we used the product and company names as keywords, individually and in combination, to identify clinical trials. We included all publications that evaluated the effectiveness or efficacy of the included products, including both randomized controlled trials (RCTs) and observational studies. We reviewed all publications listed at the end of the ClinicalTrials.gov registration for potential inclusion. For the MEDLINE search, product and company names were used as keywords. For the product website search, publications listed as clinical evidence on company websites were included. Two reviewers independently screened each publication, examining the title and abstract as well as the full text, where appropriate. Reviewer disagreements were reconciled by discussion. We screened and included only those articles published before March 2022. We did not include pilot or feasibility studies.

For example, the first stage of the search identified the PYT product code when the “device name” field was searched for “software.” All registrations coded as PYT (ie, “Device, Fertility Diagnostic, Contraceptive, Software Application”) were then evaluated for inclusion based on corresponding supporting documents, 510k decision summaries, and De Novo decision summaries. One included 510k for this product code was for the Clue app, K193330. In the second stage, we searched ClinicalTrials.gov using the keywords “Clue,” “Clue Birth Control,” “Biowink,” “Dynamic Optimal Timing,” and “Cycle Technologies.” We searched MEDLINE using the keywords “Dynamic Optimal Timing,” “Biowink,” and “NCT02833922.” Finally, we searched the product website [24] for clinical trial documents.

### Data Extraction

For each publication, one reviewer extracted data and the other reviewer checked the accuracy of the data. Differences were reconciled by discussion between the reviewers. The Cochrane Data Collection Form for Intervention Reviews [25] was completed with clinical trial characteristics, including the design, number of participants, sampling method, interventions, and outcomes.

The remainder of the data extraction form was created using the criteria for reporting adherence metrics described in the *Adherence and Engagement Metrics* section and the assumptions for the associated efficacy analysis method described in the *Analysis of Efficacy* section. Given the diversity of the apps and outcomes, we reported each metric that a clinical trial or study reported separately, without averaging across different metrics. When evaluating efficacy analyses, we categorized trials or studies as fulfilling the positivity condition if they had a control group. We categorized trials as fulfilling the consistency condition if they had definitions of treatment and adherence that avoided hidden variations of treatment that might affect participants differently.

Some assumptions, referenced in Table 1 and described in Multimedia Appendix 1, could not be fully evaluated. One such assumption is SUTVA, which requires no interaction between units of observation that could affect a result. Although it is impossible to prove that this assumption holds, some trial designs afford greater confidence than others. For example, if a trial has no central clinical team and treatment is administered only through an app, it would be difficult for participants to interact with the clinical research staff. By contrast, if clinical research staff interact with both the control and treatment groups, they might treat participants in the 2 groups in ways that affect their independence. We categorized a trial as fulfilling SUTVA if it had no central clinical team or if it had mechanisms for reducing the risk of interaction between participants or between participants and staff.

Similarly, it is impossible to fully evaluate the assumption that there are no unmeasured confounders. Instead, we asked whether the researchers demonstrated awareness of confounders by listing potential confounders explicitly and reporting their rationale for selecting them.

The results in the *Adherence Metrics* section and *Analysis of Efficacy* section below summarize practices for the included trials using means or counts as appropriate. Given the heterogeneity of the therapeutics and outcomes, we did not estimate the overall impact of all biases. The protocols and preregistrations referenced in the included articles were used as supporting documents. The protocol for this review was registered on the Open Science Framework [26], which includes the data extraction forms and extracted data. Article screening data, extracted data, and summarized extracted data are also available in Multimedia Appendices 2-4 [27-50].

## Results

### Included Trials

**Figure 1:**
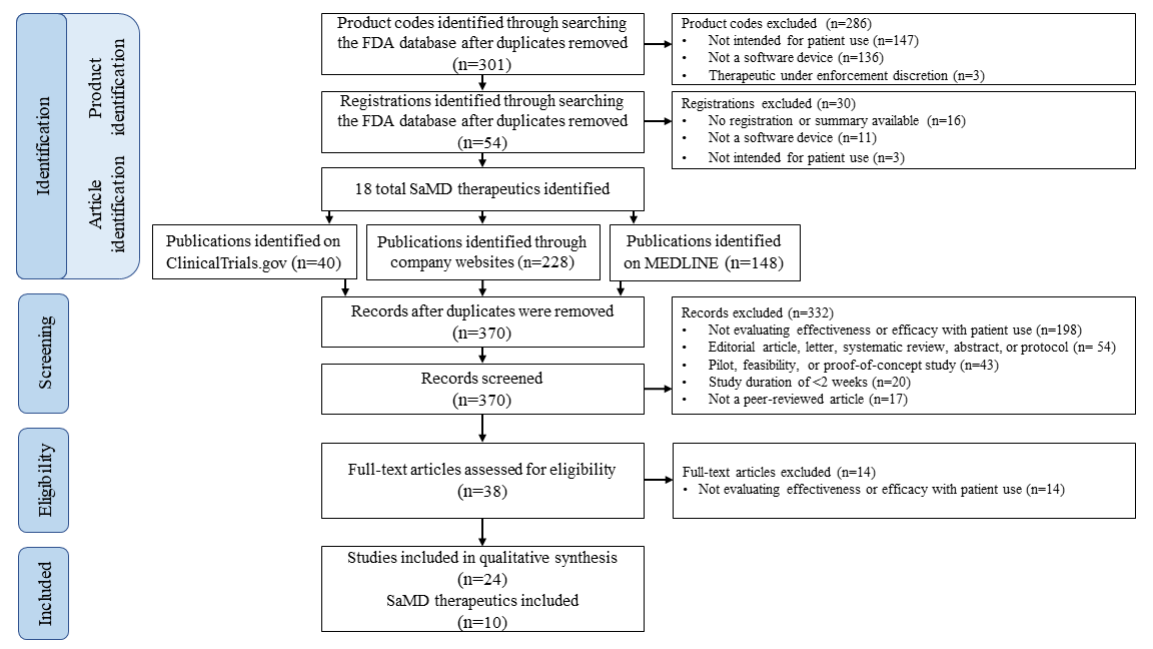
The 2-stage strategy used to identify trials and studies of software as a medical device (SaMD) therapeutics. The Food and Drug Administration (FDA) databases were first searched for SaMD therapeutics that would be used by patients for at least 2 weeks. In the second stage, ClinicalTrials.gov, MEDLINE, and company websites were then searched for articles evaluating effectiveness or efficacy for these products when used by patients for at least 2 weeks.

Figure 1 shows the completed PRISMA (Preferred Reporting Items for Systematic Reviews and Meta-Analyses) diagram. The 2-stage search for SaMD therapeutics identified 5% (15/301) of product codes and 44% (24/54) of registrations as potential SaMDs. These registrations included 18 unique SaMD therapeutics. Our search of ClinicalTrials.gov, company websites, and MEDLINE identified 40, 228, and 148 articles, respectively. After screening and removal of duplicate articles, 24 articles, involving 10 products, met all the inclusion criteria. A total of 8 products were excluded because clinical trials or observational studies evaluating efficacy for at least 2 weeks were not found in our literature search.

As seen in Tables 2 and 3, the 24 included articles (22 total trials) studied a variety of SaMD therapeutics, including those intended to treat irritable bowel syndrome, insomnia, substance use disorder, and attention-deficit/hyperactivity disorder. All the SaMD therapeutics were mobile apps and will be referred to as apps for the remainder of the article. Table 3 shows an even mix of apps intended for continual use or module-based apps. Most trials (18/22, 82%) specified a recommended dose for their app, such as the frequency of use or the number of modules to complete. Overall, 11 (50%) trials or studies studied apps used a module-based design with a recommended dose for the app [27,34-39,45-50], whereas 7 (32%) trials or studies studied apps used a continual use design with a recommended dose for the app [31,32,40-44]. Apps without a recommended dose only used the continual use design (4/22, 18%) [28-30,33].

**Table 2:** Included articles and associated products.

Product and condition treated		Study, year	Title
**Apple Irregular Arrhythmia Notification**			
	Irregular arrhythmia notification	Perez et al [27], 2019	Large-Scale Assessment of a Smartwatch to Identify Atrial Fibrillation
**BlueStar**			
	Diabetes management	Quinn et al [28], 2011	Cluster-randomized trial of a mobile phone personalized behavioral intervention for blood glucose control
	Diabetes management	Agarwal et al [29], 2019	Mobile App for Improved Self-Management of Type 2 Diabetes: Multicenter Pragmatic Randomized Controlled Trial
	Diabetes management	Dugas et al [30], 2020	Engagement and Outcomes Associated with Contextual Annotation Features of a Digital Health Solution
**Clue**			
	Contraceptive	Jennings et al [31], 2018	Estimating six-cycle efficacy of the Dot app for pregnancy prevention
	Contraceptive	Jennings et al [32], 2019	Perfect- and typical-use effectiveness of the Dot fertility app over 13 cycles: results from a prospective contraceptive effectiveness trial
**DexCom G6**			
	Diabetes management	Akturk et al [33], 2021	Real-World Evidence and Glycemic Improvement Using Dexcom G6 Features
**EndeavorRx**			
	Videogame treatment for ADHD^a^	Kollins et al [34], 2020	A novel digital intervention for actively reducing severity of paediatric ADHD (STARS-ADHD): a randomised controlled trial
	Videogame treatment for ADHD	Kollins et al [35], 2021	Effectiveness of a digital therapeutic as adjunct to treatment with medication in pediatric ADHD
	Videogame treatment for ADHD	Gallen et al [36], 2022	Enhancing neural markers of attention in children with ADHD using a digital therapeutic
**Mahana**			
	CBT^b^ for IBS^c^	Everitt et al [37]^d^, 2019	Assessing telephone-delivered cognitive–behavioural therapy (CBT) and web-delivered CBT versus treatment as usual in irritable bowel syndrome (ACTIB): a multicentre randomised trial
	CBT for IBS	Everitt et al [38]^d^, 2019	Therapist telephone-delivered CBT and web-based CBT compared with treatment as usual in refractory irritable bowel syndrome: the ACTIB three-arm RCT
	CBT for IBS	Everitt et al [39], 2019	Cognitive behavioural therapy for irritable bowel syndrome: 24-month follow-up of participants in the ACTIB randomised trial
**Natural Cycles**			
	Contraceptive	Berglund Scherwitzl et al [40], 2016	Fertility awareness-based mobile application for contraception
	Contraceptive	Berglund Scherwitzl et al [41], 2017	Perfect-use and typical-use Pearl Index of a contraceptive mobile app
	Contraceptive	Bull et al [42], 2019	Typical use effectiveness of Natural Cycles: postmarket surveillance study investigating the impact of previous contraceptive choice on the risk of unintended pregnancy
	Contraceptive	Pearson et al [43], 2021	Natural Cycles app: contraceptive outcomes and demographic analysis of UK users
	Contraceptive	Pearson et al [44], 2021	Contraceptive Effectiveness of an FDA-Cleared Birth Control App: Results from the Natural Cycles U.S. Cohort
**ReSet**			
	CBT for SUD^e^	Campbell et al [45], 2014	Internet-delivered treatment for substance abuse: a multisite randomized controlled trial
**ReSet-O**			
	CBT for OUD^f^	Christensen et al [46]^g^, 2014	Adding an Internet-delivered treatment to an efficacious treatment package for opioid dependence
	CBT for OUD	Maricich et al [47], 2021	Real-world evidence for a prescription digital therapeutic to treat opioid use disorder
	CBT for OUD	Maricich et al [48], 2021	Real-world use and clinical outcomes after 24 weeks of treatment with a prescription digital therapeutic for opioid use disorder
	CBT for OUD	Maricich et al [49]^g^, 2021	Safety and efficacy of a prescription digital therapeutic as an adjunct to buprenorphine for treatment of opioid use disorder
**Somryst**			
	CBT for Insomnia	Ritterband et al [50], 2017	Effect of a Web-Based Cognitive Behavior Therapy for Insomnia Intervention With 1-Year Follow-up A Randomized Clinical Trial

^a^ADHD: attention-deficit/hyperactivity disorder.

^b^CBT: cognitive behavioral therapy.

^c^IBS: irritable bowel syndrome.

^d^Everitt et al [37] and Everitt et al [38] were based on the same trial.

^e^SUD: substance use disorder.

^f^OUD: opioid use disorder.

^g^Christensen et al [46] and Maricich et al [49] were based on the same trial.

**Table 3:** Summary of devices and trials included in the study (n=22).

Characteristics				Values
**Therapeutic indication for use, n (%)**				
	Contraceptive		7 (32)	
	Videogame treatment for ADHD^a^		3 (14)	
	Irregular arrhythmia notification		1 (5)	
	Diabetes management		4 (18)	
	**Cognitive behavioral therapy**		7 (32)	
		IBS^b^	2 (9)	
		Insomnia	1 (5)	
		Substance use disorder	4 (18)	
**Type of therapeutic, n (%)**				
	Recommended use with module design		11 (50)	
	Recommended use with continual use design		7 (32)	
	No recommended use with module design		0 (0)	
	No recommended use with continual use design		4 (18)	
**Trial design**				
	**RCT^c^ [28,29,34,37-39,45,46,50], n (%)**		8 (36)	
		Participants (in comparison groups), mean (SD)	290 (120)	
		Trial length (d), mean (SD)	300 (270)	
	**Observational [27,30-33,35,36,40-44,47-49], n (%)**		14 (64)	
		Participants (in comparison groups), mean (SD)	5100 (7000)	
		Trial length (d), mean (SD)	230 (140)	

^a^ADHD: attention-deficit/hyperactivity disorder.

^b^IBS: irritable bowel syndrome.

^c^RCT: randomized controlled trial.

Most trials (14/22, 64%) were observational, with the remainder being RCTs (8/22, 36%). On average, the RCTs recruited 290 (SD 120) participants and lasted 300 (SD 270) days. On average, the observational trials recruited 5100 (SD 7000) participants and lasted 230 (SD 140) days.

### Adherence Metrics

Table 4 summarizes how the articles measured and reported each of the 3 aspects of adherence. As each article could report different adherence metrics for the same trial or study and report separate analyses, duplicate trials and studies were counted twice. Of the 24 articles, 23 (96%) collected information about app engagement. All apps that provided recommendations (8/8, 100%) also collected information about adherence to their recommendations [27,31,32,40-44]. Of the 23 articles that collected adherence information, 2 (9%) reported that adherence information was collected externally from the marketed app [31,32]. Three articles reported that researchers attempted to increase adherence by notifying inactive patients [34-36]. One reported the use of in-app notifications and 2 reported using email notifications.

**Table 4:** Summary of adherence metrics (N=24)^a^.

Adherence metrics					Values, n (%)			Each reported metric (%), mean (SD)
Trial collected information about app engagement					23 (96)			N/A^b^
Trial collected information about adherence to recommendations (n=8 articles for apps that gave recommendations)					8 (100)			N/A
Adherence information collected outside of the marketed app (n=23 articles for apps that collected adherence information)					2 (9)			N/A
Adherence notification sent outside of app (n=3 articles reported sending adherence notifications)					2 (67)			N/A
**Engagement metrics (metric is not measuring prescribed use)**								
	**Initiation**			2 (8)			N/A	
		Initial app use, core completion, or activity use [30,33]	2 (8)			52 (35)		
	**Implementation**			2 (8)			N/A	
		Completed sessions, modules, or activities [29,30]	2 (8)			20 (22)		
		Log-in days [29]	1 (4)			23^c^		
	**Persistence**			7 (29)			N/A	
		Percentage of participants continuing use at 1 y [31,32,40,41,43,44]	6 (25)			52 (12)		
		Number of days participants used the app [30]	1 (4)			153^c^		
**Adherence metrics (metric is measuring prescribed use)**								
	**Initiation**			15 (63)			N/A	
		Provided at least 20 d of data [40-44]	5 (21)			100 (0)		
		Initial app use, core completion, or activity use [35,36,38,47-50]	7 (29)			98 (4)		
		Entered at least 2 period start dates [31,32]	2 (8)			100 (0)		
		Initiation of video in response to app alert [27]	1 (4)			44^c^		
	**Implementation**			16 (67)			N/A	
		Completed sessions, modules, or activities [34-36,45,49]	5 (21)			88 (16)		
		Completed at least 4 sessions and 1 call [37-39]	3 (13)			64 (5)		
		Completed half of the modules [47,48]	2 (8)			76 (13)		
		Completed ≥8 core modules [47,48]	2 (8)			87 (9)		
		Percentage of logged intercourse on red days [43,44]	2 (8)			23 (0)		
		Percentage of total days intercourse logged on red days (ie, days where the user did not follow app recommendations) [42]	1 (4)			2^c^		
		Percentage of perfect use cycles (ie, menstruation cycles where the user followed all trial recommendations) [32,41]	2 (8)			17 (10)		
		Log-in days [40,43,44]	3 (13)			47 (19)		
	**Persistence**			4 (17)			N/A	
		Participants using the app at week 12 [47,48]	2 (8)			4 (17)		
		Completed all core modules [38,47,48,50]	2 (8)			49 (19)		
	Study reported all prescribed facets of adherence (n=20 studies that prescribed a recommended use of the app)			9 (45)			N/A	

^a^The left-hand columns report what percentage of articles reported adherence or engagement information and what metrics were used by each article. The right-hand columns report the mean and SD for all the articles that reported that metric.

^b^N/A: not applicable for summary of facets of adherence.

^c^SD values are not applicable as only 1 article was included.

A total of 4 articles studied a product without prescribing how often to use the app. Engagement was reported in 3 articles on these products. Of the 24 articles, engagement was reported for 2 (8%) in terms of initiation, 2 (8%) in terms of implementation, and 1 (4%) in terms of persistence. Two continual use therapeutics prescribed app use in terms of initiation and implementation but not persistence. As such, 25% (6/24) of the articles studying these apps reported engagement persistence metrics.

Of the 24 articles, 15 (63%) reported initiation in 4 different ways (eg, the number of users who finished the first app module and the number of users who entered 20 data points into the app). Seven articles excluded participants who did not initiate app use, leading to a high adherence for their adherence metrics. Of the 24 articles, 16 (67%) reported implementation, with 9 different definitions (eg, proportion of days between starting and stopping the use of an app that users logged their temperature and the number of perfect use cycles reported by women [ie, abstaining or using contraception on all high-risk days]). Of the 24 articles, 4 (17%) reported persistence, with 2 different definitions (participants using the app over the prescribed period and participants completing the prescribed number of modules). Table 4 reports the percentage of studies and the average adherence across trials and studies that used each metric. Of the 20 articles that prescribed use of the app, only 9 (45%) reported all prescribed facets of adherence [32,39-44,47,48].

### ROB: “Nonadherence to the Assigned Intervention Regimen”

Of the 24 articles, 4 (17%) only reported engagement information, as there was no prescribed amount of app use. We found that the outcomes of the remaining articles could have been affected by nonadherence. Of the 83% (20/24) of articles for apps with prescribed use, 25% (5/20) reported adherence at or below their definition of low adherence for at least 1 facet of adherence. Of the remaining 15 articles, 12 (80%) reported that there was some nonadherence with the app for any prescribed facet of adherence or the app’s behavior recommendations but did not provide a definition of low adherence. These articles provided insufficient information to determine whether adherence was sufficient for each app. The remaining 3 articles did not report sufficient information about each prescribed facet of adherence to judge adherence.

### Analysis of Efficacy

Table 5 summarizes the effectiveness and efficacy estimates from each article. Of the 24 articles, 20 (83%) estimated the app’s effectiveness as the ATE for all participants. Of these 20 articles, 11 (55%) preregistered their analysis of effectiveness. A higher percentage of RCTs preregistered their effectiveness analysis (7/9, 78%) compared with observational studies (4/11, 36%). Of the 24 articles, 15 (63%) estimated efficacy in terms of the ATE, per-protocol effect, or dose-response effect. Of these 15 articles, only 5 (33%) preregistered an efficacy analysis. Preregistration was more common for RCTs (3/6, 50%) than for observational trials (2/9, 22%).

**Table 5:** Summary of efficacy estimates (N=24).

Efficacy estimates				Values, n (%)		References
**Effectiveness estimate**				20 (83)		—^a^
	None			4 (17)		[30,33,34,36]
	Average treatment effect			20 (83)		[27-29,31,32,35,37-50]
	**Preregistered effectiveness analysis (n=20)**			11 (55)		—
		RCT^b^ (n=9)	7 (78)		[28,29,37,39,45,49,50]	
		Observational (n=11)	4 (36)		[27,31,32,35]	
**Efficacy estimate**				15 (63)		—
	None			9 (38)		[27,28,31,35,40,42,45,49]
	Average treatment effect			2 (8)		[34,36]
	Per-protocol effect			10 (42)		[30,32,33,37-39,41,43,44,50]
	Dose-response effect			3 (13)		[29,47,48]
	**Preregistered efficacy analysis (n=15)**			5 (33)		—
		RCT (n=6)	3 (50)		[34,37,39]	
		Observational (n=9)	2 (22)		[32,36]	

^a^References not listed for summary rows.

^b^RCT: randomized controlled trial.

Table 6 characterizes the articles in terms of how well they meet the assumptions for their method of analysis. Of the 24 articles, 2 (8%) estimated efficacy in terms of ATE [34,36]. One of them used intent-to-treat analysis and met the relevant reporting requirement [34], and the other article calculated the ATE for an observational trial [36]. It met the criteria for SUTVA and had a clear definition of the treatment condition. However, it did not meet the positivity condition and lacked a control condition. The study adjusted for 1 confounder without saying how it was chosen.

**Table 6:** Fulfillment of required assumptions for efficacy analyses (n=14).

Estimate category, analysis method, and article		SUTVA^a^, n (%)	Positivity, n (%)	Consistency, n (%)		Exclusion restriction, n (%)	Strong monotonicity, n (%)	Assignment mechanism ignorability			
				Clear treatment definition	Clear adherence definition			Randomization, n (%)	Conditional independence of treatment and outcomes	Sequential exchangeability	Conditional independence of adherence and outcomes
									Control variables	Control variables	Control variables
**Average treatment effect (n=2)**											
	*Intent-to-treat analysis* (n=1)	1 (100)	1 (100)	1 (100)	NR^b^	1 (100)	1 (100)	1 (100)	NR	NR	NR
	Kollins et al [34] (n=1)	1 (100)	1 (100)	1 (100)	NR	1 (100)	1 (100)	1 (100)	NR	NR	NR
	*Average treatment effect analysis* (n=1)	1 (100)	0 (0)	1 (100)	NR	NR	NR	NR	NR	NR	NR
	Gallen et al [36] (n=1)	1 (100)	0 (0)	1 (100)	NR	NR	NR	NR	Basic response time	NR	NR
**Per-protocol effect (n=9)**											
	*Complier average causal effect analysis* (n=1)	1 (100)	1 (100)	1 (100)	1 (100)	1 (100)	1 (100)	1 (100)	NR	NR	NR
	Everitt et al [37,38] (n=1)	1 (100)	1 (100)	1 (100)	1 (100)	1 (100)	1 (100)	1 (100)	NR	NR	NR
	*Generalized estimation* (n=0)	—^c^	—	—	—	NR	NR	NR	—	NR	—
	*As-treated analysis* (n=3)	2 (67)	1 (33)	3 (100)	3 (100)	NR	NR	NR	NR	NR	N/A^d^
	Ritterband et al [50] (n=1)	1 (100)	0 (0)	1 (100)	1 (100)	NR	NR	NR	NR	NR	Baseline ISI^e^
	Dugas et al [30] (n=1)	1 (100)	1 (100)	1 (100)	1 (100)	NR	NR	NR	NR	NR	Time and demographic characteristics
	Akturk et al [33] (n=1)	1 (100)	0 (0)	0 (0)	0 (0)	NR	NR	NR	NR	NR	None
	*Per-protocol analysis* (n=5)	5 (100)	1 (20)	5 (100)	5 (100)	NR	NR	NR	NR	NR	N/A
	Everitt [39] (n=1)	1 (100)	1 (100)	1 (100)	1 (100)	NR	NR	NR	NR	NR	Known baseline predictors of missingness at 12 months (IMD^f^ and IBS-SSS^g^)
	Berglund Scherwitzl et al [41] (n=1)	1 (100)	0 (0)	1 (100)	1 (100)	NR	NR	NR	NR	NR	None
	Jennings et al [32] (n=1)	1 (100)	0 (0)	1 (100)	1 (100)	NR	NR	NR	NR	NR	None
	Pearson et al [43] (n=1)	1 (100)	0 (0)	1 (100)	1 (100)	NR	NR	NR	NR	NR	None
	Pearson et al [44] (n=1)	1 (100)	0 (0)	1 (100)	1 (100)	NR	NR	NR	NR	NR	None
**Dose-response effect (n=3)**											
	*Dose-response analysis (IV*^h^*method;* n=0*)*	—	—	—	—	—	—	—	—	—	—
	*Dose-response analysis (confounder adjustment method;* n=3*)*	3 (100)	1 (33)	3 (100)	3 (100)	NR	NR	NR	NR	NR	N/A
	Agarwal et al [29] (n=1)	1 (100)	1 (100)	1 (100)	1 (100)	NR	NR	NR	NR	NR	Baseline hemoglobin A_1c_
	Maricich et al [47] (n=1)	1 (100)	0 (0)	1 (100)	1 (100)	NR	NR	NR	NR	NR	None
	Maricich et al [48] (n=1)	1 (100)	0 (0)	1 (100)	1 (100)	NR	NR	NR	NR	NR	None

^a^SUTVA: stable unit treatment value assumption.

^b^NR: not required (for the analysis method).

^c^No included articles used the analysis method.

^d^N/A: not applicable (count is not applicable for listed control variables).

^e^ISI: insomnia severity index.

^f^IMD: index of multiple deprivation.

^g^IBS-SSS: irritable bowel syndrome symptom severity score.

^h^IV: instrumental variable.

Of the 14 articles that estimated efficacy, 9 (64%) estimated efficacy in per-protocol effect terms (ie, treatment effect for adherent participants). One trial (2 articles) calculated the complier average causal effect (CACE), or local ATE (LATE), and provided evidence of meeting its assumptions [37,38]. Three articles used as-treated analysis [30,33,50]. Three of these articles had strong support for the SUTVA assumption. Two articles met the requirements for consistency, whereas the third article did not, as it defined treatment loosely. Two articles accounted for confounders but did not mention how they were chosen. Five articles used per-protocol analysis [32,39,41,43,44]. All articles had strong support for the SUTVA assumption and clear definitions of treatment and adherence. One article used an RCT design, provided evidence of positivity, and accounted for the baseline predictors of missingness. Four articles had no control cohort and did not account for any potential confounders of adherence.

Three articles estimated dose-response effects [29,47,48], treating adherence as a moderator. All 3 articles had strong support for the SUTVA assumption and provided clear definitions of treatment and adherence. In total, 33% (1/3) of the articles used an RCT design, providing evidence of positivity. This paper corrected for 1 confounder without saying how it was chosen.

### ROB: “Analysis Used to Estimate the Effect of Adhering to the Intervention”

Of the 20 articles with a recommended dose, only 2 (10%) used an appropriate method of analysis to estimate the impact of nonadherence. Both reported on a trial that calculated CACE or LATE based on a preregistered plan, demonstrating compliance with its assumptions [37,38].

## Discussion

### Recommendations for Future Trials

Our systematic review of the SaMD literature found 24 articles evaluating the clinical evidence for 10 unique apps. These apps covered a breadth of treatment areas, risk levels, and prescribed uses.

#### Adherence Metrics

On the basis of our evaluation of adherence metrics, we identified the following key issues and opportunities to address them in future SaMD trials and studies:

1. Trial and study reporting was inconsistent. Many trials did not report all 3 facets of adherence. Trials used many definitions for each facet of adherence, limiting comparisons.
2. Some trials measure, analyze, and report adherence in ways likely to produce estimates inconsistent with those experienced with actual use.

As mentioned in the *Adherence Metrics* section of the results, most trials (23/24, 96%) collected some engagement information, but only a minority (9/20, 45%) reported all the prescribed facets of adherence. Most trials reported metrics for initiation (17/24, 71%) and implementation (18/24, 75%), and fewer trials (11/24, 46%) reported metrics for persistence. Persistence may have been reported less often because studies often reported persistence solely in terms of study dropout (adherence to trial or study protocols) but not discontinued app use. For example, 1 common outcome of trials evaluating an app treating substance use disorder was the number of days until the last face-to-face therapy session. This metric addresses 1 aspect of persistence for the treatment but neglects persistence for use of the app.

When an app offered behavior recommendations, adherence was often reported only for adherence to app recommendations or app use. For example, many contraceptive studies had complete reports on sexual activity but no reports on how often the temperature or cycle start information was logged. Such missing information could help physicians reviewing the literature to provide recommendations or warnings to patients regarding products with low adherence or engagement, better informing their patients’ consumer choices.

Within just these few articles, there were many definitions of adherence, even for apps with similar treatment mechanisms or application areas. This variety limits the possibilities for meta-analysis or app comparisons (eg, is engagement higher when 75% of users complete half of the modules or when users complete 75% of the modules on average?). In an ideal world, patients or their care providers would be able to compare adherence and engagement metrics across similar apps to choose the app that has the best outcomes and highest levels of engagement.

We recommend that the FDA’s guidance or voluntary standards determine which metrics should be collected and reported. Both guidance and standards could recognize that the most important metrics would vary across treatment areas and app design. The FDA’s guidance could provide broad recommendations for researchers to collect and report adherence information for all prescribed facets of adherence. Voluntary standards for each treatment could benefit from further studies of engagement, which would identify which metrics are most important for each treatment area. Standards would enable developers, providers, and consumers to compare the usability and efficacy of similar apps and researchers to conduct meta-analyses for apps in a treatment area. STROBE (Strengthening the Reporting of Observational Studies in Epidemiology) and CONSORT (Consolidated Standards of Reporting Trials) provide examples of reporting protocols that could be adapted. The required information could be reported in the text, as supplemental material, or on an external site such as the Open Science Framework.

Several trials included external notifications intended to increase engagement that nontrial users would not have, such as manual notifications prompted by nonadherence. For example, most trials of an app treating attention-deficit/hyperactivity disorder sent email notifications to participants in response to nonactivity. This raises the question of how the app would perform without these notifications. The collection of trial-related information can itself introduce statistical bias if the information is solicited differently than would be the case with normal use. For example, studies on a contraceptive app collected information about sexual behavior daily within the study’s app version, whereas the marketed version did not collect that information at the time of approval. Users in the trial might have been more engaged with the app; therefore, they were more likely to follow its recommendations compared with typical users. The marketed version of the app has since been updated to allow users to report sexual activity.

We recommend either regulatory guidance or voluntary standards that require trials to collect adherence information in ways similar to actual use. We recognize that, for some therapeutics, in-app notifications would not be practical or as effective. In such cases, regulators could demand postmarket evidence that measured effectiveness in real-world settings without manual notifications or external mechanisms for collecting adherence used in trials or studied are absent, aligns with measured effectiveness.

#### Analysis of Efficacy

On the basis of our evaluation of efficacy analysis, we identified the following key issues and opportunities to address them in future SaMD trials and studies:

1. Preregistration is common for analyses of effectiveness, especially for RCTs. Preregistration is uncommon for efficacy analyses of RCTs or observational studies.
2. Many articles analyzed efficacy with statistically biased methods.
3. Few articles reported evidence of meeting the assumptions for their efficacy analyses.

Preregistration is the accepted practice for protecting studies from *p-hacking* (ie, capitalization on chance), such as trying different analyses until a desired or expected result is obtained. Efficacy analyses for app trials are particularly susceptible to such practices, given their many variables and alternative definitions of metrics. For example, estimates of per-protocol effects depend on how researchers dichotomize participants into adherent and nonadherent groups. Different thresholds can produce different estimates.

Currently, regulators recommend that observational studies used to generate real-world evidence create and follow a protocol and analysis plan [7]. They also recommend that manufacturers follow a presubmission process to receive feedback on the plan. This consultation process is different than preregistration, where researchers publicly state their planned analyses and outcomes. Although both processes can reduce the risk of p-hacking, preregistration has the advantage of allowing the public to access study information in a standard, time-stamped manner.

In addition, we found that preregistration was more common for effectiveness analyses than for efficacy analyses. Effectiveness is likely to be the primary outcome of interest for a study and would be the primary concern for regulators reviewing an analysis plan. To protect against statistical bias, it is also important to specify in advance which analyses will be conducted in the case of low adherence and how low adherence will be defined. The analysis plan for 2 articles studying an app for the treatment of irritable bowel syndrome provides an excellent reference for defining adherence metrics and specifying analysis for low-adherence cases [37,38].

We recommend that regulators require preregistration of all app trials, in a standard format that specifies the planned metrics and analyses. Prespecified plans should include the analytical method used, any threshold for dichotomizing adherence, and the plan to account for confounders. Preregistrations should also address the conditions and methods for analyzing efficacy, such as the threshold for low adherence that would trigger efficacy analyses. Voluntary standards could mandate preregistration before updated regulatory requirements are implemented. Scientific journals might impose standards more quickly than regulatory bodies, as they have for preregistration of interventional trials [51,52].

Most trials studied efficacy and effectiveness using various methods. Most studies estimated a per-protocol effect using as-treated or per-protocol analysis, methods that studies have found to produce statistically biased results owing to insufficient adjustments for selection bias and confounders [53,54]. Our review also found insufficient accounting for confounders.

Only 1 trial (2 articles) used the preferred CACE, or LATE, analysis [37,38]. However, many other trials could have used this method, given their RCT designs. CACE, or LATE, analysis accounts for confounders without perfect knowledge of the relationships between outcomes and confounders using randomization as an instrumental variable.

We recommend that regulatory guidance or voluntary standards require less biased methods of estimating the per-protocol effects when the trial or study design allows. For example, an RCT using per-protocol analysis should use CACE, or LATE, analysis instead, treating access to treatment assignment as an instrumental variable. With observational studies, less biased instrumental variables approach methods are often not possible, given the lack of a control condition. In such cases, confounder adjustment could be used, with explicit acknowledgment of its limitations. Most articles reported satisfying the requirements for SUTVA, positivity, and consistency. However, the validity of all analytical methods also depends on satisfying ignorability, namely, accounting for confounders related to treatment and adherence, using an instrumental variable or confounder adjustment. Confounder adjustment is needed for ATE analysis with observational studies, as-treated analysis, per-protocol analysis, and dose-response analysis with confounder adjustment. Table 6 shows that confounders were often not even considered for these efficacy analyses. Even when confounders were considered, the rationale for choosing them was often not stated.

We recommend regulatory guidance or voluntary standards that clearly specify how researchers should choose and report confounders for efficacy analyses. Given that confounders will vary by treatment area, regulatory guidance should focus on general best practices, such as including transparent, preregistered methods for confounder selection. Whenever possible, confounders should be selected based on prior knowledge of causal relationships [55,56]. Voluntary standards could identify confounders for common treatment areas when such research exists. Without such research, empirical methods of confounder selection could be used, with the disclosure of potential bias in the selection method.

### Conclusions

Most of the trials included in our systematic review report data suggesting nonadherence that could have affected the effectiveness of the app, without sufficiently evaluating efficacy in these circumstances. Appropriate use of SaMDs requires an understanding of how adherence could function as a moderator of the outcomes. Realistic, unbiased efficacy estimates are needed by regulators evaluating apps, health care providers potentially prescribing them, consumers deciding whether to use them (or seek other treatments), and vendors trying to improve their products.

The challenge of producing unbiased estimates will grow if real-world evidence studies are used more often to estimate the effectiveness of SaMD. Together, our findings illustrate the range (and inconsistencies) of the approaches used to measure and account for adherence. Without clear regulatory guidance or voluntary standards that specify how researchers should choose adherence metrics, perform efficacy analyses, and report their methods, it is unreasonable to expect that researchers will provide the information necessary to evaluate the potential effect of adherence on trial outcomes. More rigorous and consistent reporting and analyses are needed to facilitate decisions about individual products and to aggregate knowledge across products. Future SaMD clinical trials and studies may be improved by producing consensus standards on the definitions of adherence for similar products and studying the role of confounders for product areas. Without accurate efficacy estimates, SaMDs will not fulfill their potential to improve health outcomes with minimal risk.

### Limitations and Future Work

Our review excluded qualitative and exploratory studies, thus potentially missing insights found in them. For example, exploratory studies might reveal how prescribed dosages were determined, filling a gap in this study. Our review may have also missed proprietary studies that identified confounders of adherence or developed ways to improve adherence, filling other gaps. Although our search method was thorough, following the protocol described in the *Screening* section, studies that would have been found with other protocols may have been missed. A complementary strategy for future reviews would be to use the Digital Therapeutics Alliance product page to identify additional products as a starting point for looking for related evaluation studies. As few digital health apps qualify as SaMDs, our review reflects only a small portion of the clinical trials studying digital health apps. However, as these apps are subject to the most stringent regulatory requirements, they might be expected to have the highest quality evaluations. If so, future trials and studies on all digital health apps could benefit from implementing the recommendations of this study.

## Appendix

Multimedia Appendix 1 Definitions of terms and assumptions.

Multimedia Appendix 2 Article screening.

Multimedia Appendix 3 Extracted data.

Multimedia Appendix 4 Summarized data.

Multimedia Appendix 5 PRISMA Checklist.

Multimedia Appendix 6 PRISMA Abstract Checklist.

## Abbreviations

ATE: average treatment effect
CACE: complier average causal effect
CONSORT: Consolidated Standards of Reporting Trials
FDA: Food and Drug Administration
LATE: local average treatment effect
PRISMA: Preferred Reporting Items for Systematic Reviews and Meta-Analyses
RCT: randomized controlled trial
ROB: risk of bias
SaMD: software as a medical device
STROBE: Strengthening the Reporting of Observational Studies in Epidemiology
SUTVA: stable unit treatment value assumption
