# Supplementary material for: Collection and Analysis of Adherence Information for Software as a Medical Device Clinical Trials: Systematic Review
Source: JMIR Mhealth Uhealth. 2023 Nov 15;11:e46237. doi: 10.2196/46237 (PMC10687688; doi:10.2196/46237)
Supplement: Multimedia Appendix 1 [file mhealth_v11i1e46237_app1.docx]

Multimedia Appendix 1

**Table S1.** Definitions of terms and assumptions

| Assumption | Definition |
| --- | --- |
| **Stable-Unit Treatment Value Assumption (SUTVA)** | Requires that there is no interference among study units [17]. Interference could occur if participants interact with each other in a group setting or if treatment that participants receive could be affected by participants in another group. For instance, if physicians review data received from a group treated with an app, they could change how they interact with control participants in response to that data. For an app, this could occur if researchers attempt to study one feature of the app without considering how participants interact with other features in addition to the studied feature. |
| **Consistency** | The treatment is clearly defined so that the measured outcome is equal to the potential outcome and there are no hidden variations in treatment [18]. For analysis methods requiring dichotomization, this would also require that the definition of adherence is clearly defined. In all cases, the definition of treatment and adherence should be preregistered to reduce the risk of p-hacking. |
| **Positivity** | Each participant has a non-zero chance of receiving the treatment [18]. Because all participants in a randomized trial should have an equal chance of receiving treatment, this assumption is guaranteed. |
| **Assignment Mechanism Ignorability (Ignorability)** | Treatment assignment is independent of potential outcomes [19]. This can be satisfied by random assignment or by controlling for all confounders that affect treatment.  Sequential exchangeability fits under the category of ignorability assumptions. It requires that there is conditional independence between treatment assignment and potential outcomes after conditioning on time varying confounders and the historical pattern of adherence for each participant [20].  Another version of ignorability, is conditional independence between adherence and potential outcomes after accounting for confounders. Under this assumption, the dose received is unrelated to patient characteristics that would also affect potential outcomes. |
| **Exclusion Restriction** | The effect of an instrumental variable on the outcome must be through the treatment [18]. For example, the treatment group could not be used as an instrumental variable for an observational trials since factors affecting a participant’s decision to use a treatment could also affect the outcome. |
| **Strong Monotonicity** | Requires that there are no defiers, or participants that would act in opposition of their treatment assignment if placed in the treatment or control group [18]. It also requires that the instrument has a non-zero effect on the likelihood of receiving treatment. |

Treatment Effect Definitions

Average treatment effect

*Average Treatment Effect* (ATE) is the average effect of using the app for all participants, in both the control and treatment groups. While the ATE is most often used to calculate effectiveness, it can be used to calculate efficacy if adherence is high for the trial. This could occur naturally, or, more commonly, researchers could attempt to improve adherence through regular reminders to research participants.

Estimating any causal effect, including ATE, requires satisfying four assumptions: stable unit treatment value assumption (SUTVA), consistency, positivity, and treatment assignment ignorability. To satisfy SUTVA [17], the treatment of one person (unit) cannot affect the treatment of any other unit. Thus, if one person is assigned (or chooses) to use an app, it does not affect the treatment of another person in the trial. For example, SUTVA might be violated if providers change their care for control group patients based on their observations of treatment group patients. Consistency requires that the definition of the treatment is clearly defined so that the potential outcome under treatment is equal to the observed outcome [18]. Positivity requires that the likelihood of receiving treatment for all participants is greater than zero [18]. Treatment assignment ignorability, means that treatment assignment is unrelated to confounders of the outcome [19]. For ATE, this can be satisfied by statistically controlling for all confounds of the outcome, such as demographics or baseline disease state. Given that all four assumptions are required for all causal estimates, only additional assumptions will be covered for the proceeding analysis methods.

Intent-to-treat (ITT) analysis, changes the question from “what is the effect of *using* the treatment?” to “what is the effect of being *assigned* to use the treatment?” To estimate the ITT effect, a random mechanism of assignment is needed which acts as an instrumental variable. Because an instrumental variable is used, there are additional assumptions: exclusion restriction and strong monotonicity [21]. Exclusion restriction means that the instrumental variable cannot be related to any unobserved variables that could affect the outcome. Strong monotonicity means that there are no *defiers* in the sample, individuals who abstain from treatment in the treatment group, but would obtain it in the control group, if given the opportunity. Randomization can be used to satisfy many of the required assumptions including positivity, ignorability, exclusion restriction, and strong monotonicity.

Per-protocol effect

The *per-protocol effect* estimates the effect of an intervention when users have fulfilled the terms of a protocol. With an app, that might include completing half of the assigned modules or using the app on at least X% of days. Common methods for estimating per-protocol effects include complier average causal effect (CACE)/local average treatment effect (LATE) analysis, g-estimation, as-treated analysis and per-protocol analysis. These methods dichotomize participants into adherent and non-adherent groups, as defined by an adherence threshold.^[[1]](#footnote-1)^ This adds an additional consistency requirement, where the definition of adherence needs to have a clear and natural meaning.

The *Complier Average Causal Effect* (CACE/LATE) estimates the effect of the intervention in the sub-population of compliers. CACE/LATE assumes (a) that the treatment would have had the same effect for adherent participants in the control group, had they been provided treatment; (b) that the treatment would not have had the same effect on non-adherent participants in the treatment group, had they taken it; and (c) that the effects for non-adherent participants in the treatment group is equal to that for people in the control group who would not have taken the treatment had they been in the treatment group. Under this assumption, CACE/LATE analysis subtracts the effect of non-adherence on outcomes, as measured in the treatment group, from the change in outcomes in the control group. When adherent and non-adherent participants are different because of confounding factors, CACE/LATE is more appropriate than AT or PP, which cannot account for unmeasured confounds.

Often adherence is longitudinal and past adherence can affect future adherence. In the case of the insomnia app, if someone did not adhere to the first module, they could be more fatigued when starting the second module, making adherence more challenging for them to achieve in this time-period as well. In this scenario, there is a feedback loop between adherence and confounders that is not accounted for with IV methods such as CACE/LATE. G-estimation is a method that addresses this feedback loop [18]. It estimates the effect of the intervention given different adherence scenarios such as if all participants never adhered, adhered in early time periods, late time periods, or always adhered [20]. To use g-estimation, sequential ignorability, which requires the measurement and control for pre-randomization and post-randomization confounders of adherence needs to hold.

Neither AT or PP use an instrumental variable to calculate the per-protocol effect. AT moves non-adherent participants from the treatment group to the control group; PP removes them from the analysis altogether. Because group assignment is based on adherence, conditional independence between adherence and outcomes is required for ignorability to hold. In other words, all confounds that affect adherence need to be accounted for. AT and PP can overestimate efficacy, when reasons for non-adherence in the treatment group are related to outside factors that often occur in the real world (e.g., lack of wifi-accessibility) and confounds are not controlled for.

Dose-response effect

As described so far, these estimates (ATE, and PP) treat adherence as dichotomous, ignoring any dose-response relationship between the degree of adherence and health outcome. Such estimates can inform decisions about average users. Dose-response estimates could refine these decisions when it is possible to (a) measure user adherence patterns and (b) account for variables that confound the effects of adherence.

The latter need (b) can be addressed either by assessing the relationships between potential confounds and measures of adherence or through instrumental variable regression [22]. For this analysis method, conditional independence between adherence and outcomes is needed to satisfy ignorability [22]. In a randomized controlled trial, treatment assignment may be used as an instrumental variable for adherence; instrumental variable regression could then be used to estimate the dose-response effect. In order to have an unbiased estimate of the dose-response effect with this method, randomization or an instrumental variable that fulfills exclusion restriction and strong monotonicity are needed.

1. To maintain scientific rigor, one should preregister what threshold or thresholds they plan to test and report all of the results corresponding to the preregistration. [↑](#footnote-ref-1)
