# Supplementary material for: Collection and Analysis of Adherence Information for Software as a Medical Device Clinical Trials: Systematic Review
Source: JMIR Mhealth Uhealth. 2023 Nov 15;11:e46237. doi: 10.2196/46237 (PMC10687688; doi:10.2196/46237)
Supplement: Multimedia Appendix 2 [file mhealth_v11i1e46237_app2.pdf]

Product	Document Title	DOI	Initial Decision	Reason
Apple Electroca	Accuracy of Apple Watch for Detection of Atrial Fibril	10.1161/CIRCULATIONAHA.119.0	No	Less than two week study duration
Apple Electroca	Clinical evaluation and diagnostic yield following eval	10.1093/jamia/ocaa137	No	Not a clinical trial studying efficacy or effectiveness for the included SaMD with outpatient use
Apple Electroca	Rationale and design of a large-scale, app-based stu	10.1016/j.ahj.2018.09.002	No	Editorial article, Letter, Systematic Review, Abstract or Protocol
Apple Electroca	Smartwatch Electrocardiograms for Automated and M	10.3389/fcvm.2022.836375	No	Not a clinical trial studying efficacy or effectiveness for the included SaMD with outpatient use
Apple Irregular	Accuracy of Commercially Available Heart Rate Monitors in Athletes (HRM)		No	Not a clinical trial studying efficacy or effectiveness for the included SaMD with outpatient use
Apple Irregular	Large-Scale Assessment of a Smartwatch to Identify	DOI: 10.1056/NEJMoa1901183	Yes	Trial of an SaMD device for at least two weeks
Bluestar	A Framework for Optimizing Technology-Enabled Dia	DOI: 10.1177/0145721720935125	No	Not a clinical trial studying efficacy or effectiveness for the included SaMD with outpatient use
Bluestar	A Mobile App to Improve Self-Management of Individ	http://dx.doi.org/10.2196/jmir.8712	No	Not a clinical trial studying efficacy or effectiveness for the included SaMD with outpatient use
Bluestar	A Payer Digital Health Study Shows Scalable Approa	https://doi.org/10.1177/193229682	No	Editorial article, Letter, Systematic Review, Abstract or Protocol
Bluestar	A randomized wait-list control trial to evaluate the imp	10.1186/s12911-016-0381-5	No	Editorial article, Letter, Systematic Review, Abstract or Protocol
Bluestar	A Systematic Review of Reviews Evaluating Technol	https://doi.org/10.1177/193229681	No	Editorial article, Letter, Systematic Review, Abstract or Protocol
Bluestar	An Effective Model of Diabetes Care and Education	https://doi.org/10.1177/014572171	No	Not a clinical trial studying efficacy or effectiveness for the included SaMD with outpatient use
Bluestar	Are You Ready to Be an eEducator?	https://doi.org/10.1177/232516031	No	Editorial article, Letter, Systematic Review, Abstract or Protocol
Bluestar	Cluster-randomized trial of a mobile phone personalized behavioral intervention for bl		Yes	Trial of an SaMD device for at least two weeks
Bluestar	Complementarity of Digital Health and Peer Support	https://doi.org/10.3389/fcdhc.2021.	No	Not a clinical trial studying efficacy or effectiveness for the included SaMD with outpatient use
Bluestar	Diabetes Management & Nutrition Guide: Digital Health		No	Not a peer reviewed article
Bluestar	Engagement and Outcomes Associated with Context	10.1177/1932296820976409	Yes	Trial of an SaMD device for at least two weeks
Bluestar	Glucose Self-monitoring in Non–Insulin-Treated Patients With Type 2 Diabetes in Prima		Yes	Trial of an SaMD device for at least two weeks
Bluestar	Lessons from a community-based mHealth diabetes	DOI: 10.1080/10810730.2012.650	Yes	Trial of an SaMD device for at least two weeks
Bluestar	Mobile App for Improved Self-Management of Type 2	DOI: 10.2196/10321	Yes	Trial of an SaMD device for at least two weeks
Bluestar	Technology to overcome therapeutic inertia	http://dx.doi.org/10.21037/mhealth.	No	Editorial article, Letter, Systematic Review, Abstract or Protocol
Bluestar	WellDoc mobile diabetes management randomized c	10.1089/dia.2008.0283	Yes	Trial of an SaMD device for at least two weeks
Clue	A Cross-Country Comparison of Reasons for Condor	https://doi.org/10.1080/19317611.2	No	Not a clinical trial studying efficacy or effectiveness for the included SaMD with outpatient use
Clue	A Generative Modeling Approach to Calibrated Predi	*PMC8782440	No	Not a clinical trial studying efficacy or effectiveness for the included SaMD with outpatient use
Clue	A predictive model for next cycle start date that acco	10.1093/jamia/ocab182	No	Not a clinical trial studying efficacy or effectiveness for the included SaMD with outpatient use
Clue	Assessing the Efficacy of an App-Based Method of F	10.2196/resprot.6886	No	Editorial article, Letter, Systematic Review, Abstract or Protocol
Clue	Assessment of menstrual health status and evolution	10.1038/s41746-019-0139-4	No	Not a clinical trial studying efficacy or effectiveness for the included SaMD with outpatient use
Clue	Characterizing physiological and symptomatic variati	https://doi.org/10.48550/arXiv.190	No	Not a clinical trial studying efficacy or effectiveness for the included SaMD with outpatient use
Clue	Characterizing physiological and symptomatic variati	10.1038/s41746-020-0269-8	No	Not a clinical trial studying efficacy or effectiveness for the included SaMD with outpatient use
Clue	Condom use from a female perspective: Clue's study with KI-CURT		No	Not a peer reviewed article
Clue	Daily, weekly, seasonal and menstrual cycles in wom	10.1038/s41562-020-01046-9	No	Not a clinical trial studying efficacy or effectiveness for the included SaMD with outpatient use
Clue	Data from a Menstrual Cycle Tracking App Informs our Knowledge of the Menstrual Cycl		No	Not a peer reviewed article
Clue	Decision making over condom use during menses to	https://doi.org/10.1071/sh18136	No	Not a clinical trial studying efficacy or effectiveness for the included SaMD with outpatient use
Clue	Decision making over condom use during menses to	10.1071/SH18136	No	Not a clinical trial studying efficacy or effectiveness for the included SaMD with outpatient use
Clue	Do sexually transmitted infections exacerbate negati	https://doi.org/10.1093/emph/eoy0	No	Not a clinical trial studying efficacy or effectiveness for the included SaMD with outpatient use
Clue	Estimating six-cycle efficacy of the Dot app for pregn	10.1016/j.contraception.2018.10.0	Yes	Trial of an SaMD device for at least two weeks
Clue	Identifying Women at Risk for Polycystic Ovary Synd	https://doi.org/10.2196/15094	No	Not a clinical trial studying efficacy or effectiveness for the included SaMD with outpatient use
Clue	Is Female Health Cyclical? Evolutionary Perspectives	https://doi.org/10.1016/j.tree.2018.	No	Not a clinical trial studying efficacy or effectiveness for the included SaMD with outpatient use
Clue	Lessons From the Dot Contraceptive Efficacy Study:	10.2196/mhealth.9661	No	Not a clinical trial studying efficacy or effectiveness for the included SaMD with outpatient use
Clue	Mobile sex-tech apps: How use differs across global	https://doi.org/10.1371/journal.pon	No	Not a clinical trial studying efficacy or effectiveness for the included SaMD with outpatient use
Clue	Modeling Individual Cyclic Variation in Human Behav	https://doi.org/10.1145/3178876.31	No	Not a clinical trial studying efficacy or effectiveness for the included SaMD with outpatient use
Clue	Perfect- and typical-use effectiveness of the Dot fertil	doi: 10.1080/13625187.2019.1581	Yes	Trial of an SaMD device for at least two weeks
Clue	Personalised estimation of a woman's most fertile da	doi: 10.1080/13625187.2016.1196	No	Not a clinical trial studying efficacy or effectiveness for the included SaMD with outpatient use
Clue	Predicting pregnancy using large-scale data from a w	https://doi.org/10.48550/arXiv.1812	No	Not a clinical trial studying efficacy or effectiveness for the included SaMD with outpatient use
Clue	The menstrual cycle is a primary contributor to cyclic	https://doi.org/10.1101/583153	No	Not a clinical trial studying efficacy or effectiveness for the included SaMD with outpatient use
Clue	Towards Personalized Modeling of the Female Horm	https://doi.org/10.48550/arXiv.1712	No	Not a clinical trial studying efficacy or effectiveness for the included SaMD with outpatient use
Dexcom G6; De	A clinical trial of the accuracy and treatment experience of the Dexcom G4 sensor (Dex		No	Less than two week study duration
Dexcom G6; De	A Modified CGM Algorithm Enhances Data Availabilit	10.1177/19322968211007521	No	Less than two week study duration
Dexcom G6; De	A randomized controlled trial of transition from insulir	10.1111/dom.14423	No	Not a clinical trial studying efficacy or effectiveness for the included SaMD with outpatient use
Dexcom G6; De	A Randomized Trial of Closed-Loop Control in Children with Type 1 Diabetes		No	Not a clinical trial studying efficacy or effectiveness for the included SaMD with outpatient use
Dexcom G6; De	A1C Reductions and Improved Patient-Reported Outcomes Following CGM Initiation in		No	Not a peer reviewed article
Dexcom G6; De	Accuracy of a Factory-Calibrated, Real-Time Continu	10.1089/dia.2018.0150	No	Less than two week study duration
Dexcom G6; De	Accuracy of Dexcom G6 Continuous Glucose Monito	10.2337/dc20-2856	No	Not a clinical trial studying efficacy or effectiveness for the included SaMD with outpatient use
Dexcom G6; De	Accuracy of the Dexcom G6 Glucose Sensor during /	10.3390/bios10100138	No	Less than two week study duration
Dexcom G6; De	Accuracy, Utilization, and Effectiveness Comparisons	10.1089/dia.2018.0374	No	Not a clinical trial studying efficacy or effectiveness for the included SaMD with outpatient use
Dexcom G6; De	Closed-Loop Insulin Therapy Improves Glycemic Cor	10.1089/dia.2020.0572	No	Not a clinical trial studying efficacy or effectiveness for the included SaMD with outpatient use
Dexcom G6; De	Continuous Glucose Monitor with Siri Integration Impi	10.1089/dia.2020.0320	No	Not a clinical trial studying efficacy or effectiveness for the included SaMD with outpatient use
Dexcom G6; De	Continuous Glucose Monitoring in Critically Ill Patient	10.1177/1932296820964264	No	Pilot, feasibility, or proof-of-concept study
Dexcom G6; De	Continuous Glucose Monitoring in the Intensive Care	10.2337/dc20-2219	No	Not a clinical trial studying efficacy or effectiveness for the included SaMD with outpatient use
Dexcom G6; De	Continuous Glucose Monitoring Profiles in Healthy N	10.1210/jc.2018-02763	No	Less than two week study duration
Dexcom G6; De	CREATE (Community deRivEd AutomaTEd insulin d	10.1007/s40200-020-00547-8	No	Editorial article, Letter, Systematic Review, Abstract or Protocol
Dexcom G6; De	Demonstrating the Clinical Impact of Continuous Glu	10.1177/1932296820955228	No	Not a clinical trial studying efficacy or effectiveness for the included SaMD with outpatient use
Dexcom G6; De	Diabetes Telehealth Solutions: Improving Self-Manag	10.1210/jendso/bvaa076	No	Pilot, feasibility, or proof-of-concept study
Dexcom G6; De	DYNAMIC: Dynamic glucose management strategies	10.1111/pedi.13155	No	Not a clinical trial studying efficacy or effectiveness for the included SaMD with outpatient use
Dexcom G6; De	Early use of continuous glucose monitoring in childre	10.1111/pedi.13168	No	Less than two week study duration
Dexcom G6; De	Effect of Continuous Glucose Monitoring on Glycemic Control in Adolescents and Youn		No	Not a clinical trial studying efficacy or effectiveness for the included SaMD with outpatient use
Dexcom G6; De	Effect of Continuous Glucose Monitoring on Glycemic Control in Adults With Type 1 Dia		No	Not a clinical trial studying efficacy or effectiveness for the included SaMD with outpatient use
Dexcom G6; De	Effect of Continuous Glucose Monitoring on Hypoglycemia in Older Adults With Type 1		No	Not a clinical trial studying efficacy or effectiveness for the included SaMD with outpatient use
Dexcom G6; De	Effectiveness of real-time continuous glucose monito	10.1136/bmjopen-2020-040498	No	Editorial article, Letter, Systematic Review, Abstract or Protocol
Dexcom G6; De	First Look at Control-IQ: A New-Generation Automate	10.2337/dc18-1249	No	Pilot, feasibility, or proof-of-concept study
Dexcom G6; De	Glucose as the Fifth Vital Sign: A Randomized Contr	10.2337/dc20-1016	No	Not a clinical trial studying efficacy or effectiveness for the included SaMD with outpatient use
Dexcom G6; De	Glycemic Control Improvement in Italian Children anc	10.3389/fendo.2020.595735	No	Not a clinical trial studying efficacy or effectiveness for the included SaMD with outpatient use
Dexcom G6; De	Health-Related Quality of Life and Treatment Satisfaction in Parents and Children with		No	Not a clinical trial studying efficacy or effectiveness for the included SaMD with outpatient use
Dexcom G6; De	Impact of Human Factors Testing on Medical Device	10.1177/1932296819831071	No	Not a clinical trial studying efficacy or effectiveness for the included SaMD with outpatient use
Dexcom G6; De	Mitigation of Rebound Hyperglycemia With Real-Tim	10.1177/1932296820982584	Yes	Trial of an SaMD device for at least two weeks
Dexcom G6; De	Modeling the error of factory-calibrated continuous gl	10.1109/EMBC.2019.8856790	No	Not a clinical trial studying efficacy or effectiveness for the included SaMD with outpatient use
Dexcom G6; De	Outcomes and Healthcare Provider Perceptions of R	10.1177/1932296820985263	No	Not a clinical trial studying efficacy or effectiveness for the included SaMD with outpatient use
Dexcom G6; De	Performance of a Factory-Calibrated Real-Time Cont	10.1089/dia.2018.0143	No	Less than two week study duration
Dexcom G6; De	Performance of a factory-calibrated, real-time contin	10.1111/dom.14073	No	Less than two week study duration
Dexcom G6; De	Performance of a Factory-Calibrated, Real-Time Con	10.1177/1932296818798816	No	Less than two week study duration
Dexcom G6; De	Performance of the Dexcom G6 Continuous Glucose	10.1089/dia.2020.0085	No	Less than two week study duration
Dexcom G6; De	Rationale and protocol for the Assessment of Impact of Real-time Continuous Glucose		No	Editorial article, Letter, Systematic Review, Abstract or Protocol
Dexcom G6; De	Real-time continuous glucose monitoring in adults with type 1 diabetes and impaired h		No	Not a clinical trial studying efficacy or effectiveness for the included SaMD with outpatient use
Dexcom G6; De	Real-World Evidence and Glycemic Improvement Us	10.1089/dia.2020.0654	Yes	Trial of an SaMD device for at least two weeks
Dexcom G6; De	Real-World Hypoglycemia Avoidance with a Continuc	10.1089/dia.2018.0359	Yes	Trial of an SaMD device for at least two weeks
Dexcom G6; De	Real-World Hypoglycemia Avoidance With a Predicti	10.1177/1932296819840691	Yes	Trial of an SaMD device for at least two weeks
Dexcom G6; De	Reliability of the Dexcom G6 Continuous Glucose Mc	10.1089/dia.2019.0390	No	Less than two week study duration
Dexcom G6; De	Sustainable Use of a Real-Time Continuous Glucose	10.1089/dia.2021.0014	No	Not a clinical trial studying efficacy or effectiveness for the included SaMD with outpatient use
Dexcom G6; De	Use of Factory-Calibrated Real-time Continuous Gluc	10.2337/dc20-0736	No	Not a clinical trial studying efficacy or effectiveness for the included SaMD with outpatient use
Dexcom G6; De	Validity of continuous glucose monitoring for categori	10.1093/ajcn/nqac026	No	Not a clinical trial studying efficacy or effectiveness for the included SaMD with outpatient use
EndeavorRx	A digital intervention targeting cognitive control network dysfunction in middle age an		No	Not a clinical trial studying efficacy or effectiveness for the included SaMD with outpatient use
EndeavorRx	A Game-Based Repeated Assessment for Cognitive Monitoring: Initial Usability and Adl		No	Pilot, feasibility, or proof-of-concept study
EndeavorRX	A novel digital intervention for actively reducing sev	https://doi.org/10.1016/S2589-750	Yes	Trial of an SaMD device for at least two weeks
EndeavorRx	A novel in-home digital treatment to improve proce	doi: 10.1177/1352458520930371	No	Not a clinical trial studying efficacy or effectiveness for the included SaMD with outpatient use

EndeavorRX	A pilot study to determine the feasibility of enhancin	https://doi.org/10.1371/journal.pon	No	Pilot, feasibility, or proof-of-concept study
EndeavorRx	A Study To Evaluate The Difference In iPad-Based Cognitive Video Game (Akili Interacti		No	Not a clinical trial studying efficacy or effectiveness for the included SaMD with outpatient use
EndeavorRx	A Videogame-Based Digital Therapeutic to Improve Processing Speed in People with M		No	Pilot, feasibility, or proof-of-concept study
EndeavorRX	Across 4 studies and 1 month of treatment with AKL-T01 over one-third of children wit		No	Not a peer reviewed article
EndeavorRX	AKL-T01 - a digital treatment for inattention in ADHD - improved ADHD-related impairr		No	Not a peer reviewed article
EndeavorRx	Application of an Adaptive, Digital, Game-Based Approach for Cognitive Assessment in		No	Not a clinical trial studying efficacy or effectiveness for the included SaMD with outpatient use
EndeavorRx	Assessing Cognitive Function in Multiple Sclerosis Wi	doi: 10.2196/25748	No	Not a clinical trial studying efficacy or effectiveness for the included SaMD with outpatient use
EndeavorRX	Brief Report: Pilot Study of a Novel Interactive Digita	https://doi.org/10.1007/s10803-018	No	Pilot, feasibility, or proof-of-concept study
EndeavorRx	Characterizing cognitive control abilities in children with 16p11.2 deletion using adapti		No	Pilot, feasibility, or proof-of-concept study
EndeavorRx	Conducting a fully mobile and randomised clinical trial for depression: access, engagem		No	Not a clinical trial studying efficacy or effectiveness for the included SaMD with outpatient use
EndeavorRX	Digital health interventions (DHI) for the treatment of	10.1016/j.psychres.2021.113742	No	Editorial article, Letter, Systematic Review, Abstract or Protocol
EndeavorRX	Effectiveness of a digital therapeutic as adjunct to tre	https://doi.org/10.1038/s41746-021	Yes	Trial of an SaMD device for at least two weeks
EndeavorRx	Enhancing neural markers of attention in children with ADHD using a digital therapeuti		Yes	Trial of an SaMD device for at least two weeks
EndeavorRX	Improvements in objective atention (TOVA API/ACS) with AKL-T01 relate to improveme		No	Not a peer reviewed article
EndeavorRx	Improving late life depression and cognitive control through the use of therapeutic vid		No	Not a clinical trial studying efficacy or effectiveness for the included SaMD with outpatient use
EndeavorRX	Proof-of-concept study of an at-home, engaging, dig	https://doi.org/10.1371/journal.pon	No	Pilot, feasibility, or proof-of-concept study
EndeavorRx	Sustained benefits of cognitive training in children w	https://doi.org/10.1371/journal.pon	No	Pilot, feasibility, or proof-of-concept study
EndeavorRx	The Use and Effectiveness of Mobile Apps for Depression: Results From a Fully Remote		No	Not a clinical trial studying efficacy or effectiveness for the included SaMD with outpatient use
EndeavorRX	Treatment with AKL-T01 showed similar improvements in ADHD-related symptoms anc		No	Not a peer reviewed article
EndeavorRx	Video game training enhances cognitive control in older adults		No	Not a clinical trial studying efficacy or effectiveness for the included SaMD with outpatient use
Fitbit ECG app	Accuracy of Heart Rate Watches: Implications for We	10.1371/journal.pone.0154420	No	Not a clinical trial studying efficacy or effectiveness for the included SaMD with outpatient use
Fitbit ECG app	Accuracy of PurePulse photoplethysmography techno	10.1080/07420528.2019.1596947	No	Not a clinical trial studying efficacy or effectiveness for the included SaMD with outpatient use
Fitbit ECG app	Evaluation of Commercial Self-Monitoring Devices fo	10.3390/s17010211	No	Not a clinical trial studying efficacy or effectiveness for the included SaMD with outpatient use
Fitbit ECG app	Heart rate detection by Fitbit ChargeHR ™ : A validat	10.1111/jsr.13346	No	Not a clinical trial studying efficacy or effectiveness for the included SaMD with outpatient use
Fitbit ECG app	Increasing and Evolving Role of Smart Devices in Mo	10.15420/ecr.2019.02	No	Editorial article, Letter, Systematic Review, Abstract or Protocol
Fitbit ECG app	Is There a Benefit to Patients Using Wearable Device	10.1016/j.amjmed.2019.06.018	No	Editorial article, Letter, Systematic Review, Abstract or Protocol
Fitbit ECG app	Rationale and design of a large population study to v	10.1016/j.ahj.2021.04.003	No	Editorial article, Letter, Systematic Review, Abstract or Protocol
Fitbit ECG app	Smart watches for heart rate assessment in atrial arrl	10.1016/j.ijcard.2018.02.073	No	Not a clinical trial studying efficacy or effectiveness for the included SaMD with outpatient use
ID-Cap System	A Novel Digital Pill System for Medication Adherence	10.2196/30786	No	Not a clinical trial studying efficacy or effectiveness for the included SaMD with outpatient use
ID-Cap System	DigiPrEP: A Pilot Trial to Evaluate the Feasibility, Acc	10.1097/QAI.0000000000002854	No	Pilot, feasibility, or proof-of-concept study
ID-Cap System	Performance, Reliability, Usability, and Safety of the	*PMC5141592	No	Pilot, feasibility, or proof-of-concept study
Ingestible Ever	A Brief Training Program to Support the Use of a Dig	https://doi.org/10.2196/26213	No	Not a peer reviewed article
Ingestible Ever	Design and Delivery of Real-Time Adherence Data to	https://doi.org/10.1007/s10461-020	No	Not a peer reviewed article
Ingestible Ever	Development of a digital pill and respondent behavi	https://doi.org/10.1093/tbm/ibab11	No	Editorial article, Letter, Systematic Review, Abstract or Protocol
Ingestible Ever	Digital Pills to Measure Opioid Ingestion Patterns in	http://dx.doi.org/10.2196/jmir.7050	No	Not a peer reviewed article
Ingestible Ever	Formative qualitative response of MSM with substance use to digital pills for PrEP adher		No	Editorial article, Letter, Systematic Review, Abstract or Protocol
Ingestible Ever	High Patient Engagement with the ID-Cap™ System (	High Patient Engagement with the	No	Not a clinical trial studying efficacy or effectiveness for the included SaMD with outpatient use
Ingestible Ever	Long-Term Stability of the Electronic Sensor Compor	https://doi.org/10.1177%2F875512	No	Not a clinical trial studying efficacy or effectiveness for the included SaMD with outpatient use
Ingestible Ever	Patient Response to an Ingestible Biosensor Medication Adherence System		No	Not a peer reviewed article
Ingestible Ever	Short Communication: Bioequivalence of Tenofovir (	DOI: 10.1089/aid.2018.0073	No	Not a peer reviewed article
Ingestible Ever	Smart Capsules are a Promising Tool to Improve Medication Adherence in Clinical Trials		No	Not a peer reviewed article
Ingestible Ever	The Case for Digital Pill Use in Clinical Trials	http://dx.doi.org/10.17140/CTPOJ-	No	Not a clinical trial studying efficacy or effectiveness for the included SaMD with outpatient use
KardiaBand	Accuracy of a smartwatch based single-lead electrocardiogram device in detection of a		No	Not a clinical trial studying efficacy or effectiveness for the included SaMD with outpatient use
KardiaBand	Artificial Neural Network for Atrial Fibrillation Identification in Portable Devices		No	Not a clinical trial studying efficacy or effectiveness for the included SaMD with outpatient use
KardiaBand	Assessment of a standalone photoplethysmography (PPG) algorithm for detection of at		No	Not a clinical trial studying efficacy or effectiveness for the included SaMD with outpatient use
KardiaBand	Assessment of Heart Rhythm Disorders Using the AliveCor Heart Monitor: Beyond the		No	Not a clinical trial studying efficacy or effectiveness for the included SaMD with outpatient use
KardiaBand	Automated atrial fibrillation detection algorithm using smartwatch technology.		No	Not a clinical trial studying efficacy or effectiveness for the included SaMD with outpatient use
KardiaMobile	2017 HRS/EHRA/ECAS/APHRS/SOLAECE expert consensus statement on catheter and s		No	Editorial article, Letter, Systematic Review, Abstract or Protocol
KardiaMobile	2017 ISHNE-HRS expert consensus statement on ambulatory ECG and external cardiac		No	Editorial article, Letter, Systematic Review, Abstract or Protocol
KardiaMobile	2019 AHA/ACC/HRS focused update of the 2014 AHA/ACC/HRS guideline for the mana		No	Editorial article, Letter, Systematic Review, Abstract or Protocol
KardiaMobile	A randomized trial of pocket-echocardiography integrated mobile health device assess		No	Not a clinical trial studying efficacy or effectiveness for the included SaMD with outpatient use
KardiaMobile	A single-center randomized, controlled trial investigating the efficacy of a mHealth ECG		No	Pilot, feasibility, or proof-of-concept study
kardiamobile	Accuracy of a single-lead mobile smartphone electrocardiogram for QT interval measur		No	Not a clinical trial studying efficacy or effectiveness for the included SaMD with outpatient use
KardiaMobile	Accuracy of blinded clinician interpretation of single	https://doi.org/10.1016/j.ahj.2018.0	No	Not a clinical trial studying efficacy or effectiveness for the included SaMD with outpatient use
KardiaMobile	Age-and-sex stratified prevalence of atrial fibrillation in rural Western India: Results of		No	Not a clinical trial studying efficacy or effectiveness for the included SaMD with outpatient use
KardiaMobile	Alcohol consumption, sinus tachycardia, and cardiac arrhythmias at the Munich Octobe		No	Editorial article, Letter, Systematic Review, Abstract or Protocol
KardiaMobile	Assessing the accuracy of an automated atrial fibrillation detection algorithm using sm		No	Not a clinical trial studying efficacy or effectiveness for the included SaMD with outpatient use
KardiaMobile	Assessment of remote heart rhythm sampling using the AliveCor heart monitor to scre		Yes	Trial of an SaMD device for at least two weeks
KardiaMobile	Awareness campaigns of atrial fibrillation as an opportunity for early detection by phar		No	Not a clinical trial studying efficacy or effectiveness for the included SaMD with outpatient use
KardiaMobile	Cardiac symptom burden and arrhythmia recurrence drives digital health use: results f		No	Not a clinical trial studying efficacy or effectiveness for the included SaMD with outpatient use
KardiaMobile	Clinical stage of acquired immunodeficiency syndrome in HIV-positive patients impacts		No	Less than two week study duration
KardiaMobile	Clinical Validation of a Smartphone Based, 6-lead ECG Device		No	Not a clinical trial studying efficacy or effectiveness for the included SaMD with outpatient use
KardiaMobile	Clinical validation of heart rate apps: mixed-methods evaluation study.		No	Not a clinical trial studying efficacy or effectiveness for the included SaMD with outpatient use
KardiaMobile	Comparing a mobile ECG device with Holter monitoring for patients with palpitations ir		No	Pilot, feasibility, or proof-of-concept study
KardiaMobile	Crowd-sourcing syncope diagnosis: mobile smartphone ECG apps.		No	Not a clinical trial studying efficacy or effectiveness for the included SaMD with outpatient use
KardiaMobile	Detection of atrial fibrillation with a smartphone camera: first prospective, internationa		No	Not a clinical trial studying efficacy or effectiveness for the included SaMD with outpatient use
KardiaMobile	Detection of recurrent atrial fibrillation using novel technology.		No	Not a clinical trial studying efficacy or effectiveness for the included SaMD with outpatient use
KardiaMobile	Detection rate and treatment gap for atrial fibrillation identified through screening in c		No	Less than two week study duration
KardiaMobile	Diagnosing symptomatic arrhythmia via mobile phone.		No	Not a clinical trial studying efficacy or effectiveness for the included SaMD with outpatient use
KardiaMobile	Diagnostic accuracy of a smartphone-operated, single-lead electrocardiography device		No	Not a clinical trial studying efficacy or effectiveness for the included SaMD with outpatient use
KardiaMobile	Diagnostic accuracy of handheld electrocardiogram	http://dx.doi.org/10.1136/heartjnl-2	No	Editorial article, Letter, Systematic Review, Abstract or Protocol
KardiaMobile	Diagnostic utility of real-time smartphone ECG in the initial investigation of palpitations		Yes	Trial of an SaMD device for at least two weeks
KardiaMobile	Early detection of atrial fibrillation-atrial flutter using remote patient monitoring.		No	Not a clinical trial studying efficacy or effectiveness for the included SaMD with outpatient use
KardiaMobile	ECG measurement parameters of athletes are reliable when made with a smartphone		No	Not a clinical trial studying efficacy or effectiveness for the included SaMD with outpatient use
KardiaMobile	Effect of Smartphone-Enabled Health Monitoring Devices vs Regular Follow-up on Bloo		No	Pilot, feasibility, or proof-of-concept study
KardiaMobile	Effectiveness of a nongovernmental organization-led large-scale community atrial fibri		No	Not a clinical trial studying efficacy or effectiveness for the included SaMD with outpatient use
KardiaMobile	Effectiveness of a single lead AliveCor electrocardiogram application for the screening		No	Editorial article, Letter, Systematic Review, Abstract or Protocol
KardiaMobile	eHealth Tools to Provide Structured Assistance for Atrial Fibrillation Screening, Manage		No	Not a clinical trial studying efficacy or effectiveness for the included SaMD with outpatient use
KardiaMobile	Electrode placement in a smartphone ECG device to aid in the diagnosis of atrial flutter		No	Not a clinical trial studying efficacy or effectiveness for the included SaMD with outpatient use
KardiaMobile	Establishing a Smartphone Ambulatory ECG Service for Patients Presenting to the Emer		No	Not a clinical trial studying efficacy or effectiveness for the included SaMD with outpatient use
KardiaMobile	Estimated stroke risk, yield, and number needed to screen for atrial fibrillation detecte		No	Editorial article, Letter, Systematic Review, Abstract or Protocol
KardiaMobile	Evaluating the utility of mHealth ECG heart monitoring for the detection and managem		No	Pilot, feasibility, or proof-of-concept study
KardiaMobile	Evaluation of general practitioners' single-lead electrocardiogram interpretation skills:		No	Not a clinical trial studying efficacy or effectiveness for the included SaMD with outpatient use
KardiaMobile	Expanding a single-lead mobile electrocardiographic device to multiple-lead recordings		No	Not a clinical trial studying efficacy or effectiveness for the included SaMD with outpatient use
KardiaMobile	Feasibility and acceptability of atrial fibrillation screening using a hand-held ECG device		No	Pilot, feasibility, or proof-of-concept study
KardiaMobile	Feasibility and cost-effectiveness of stroke prevention through community screening fo		No	Pilot, feasibility, or proof-of-concept study
KardiaMobile	Feasibility of Atrial Fibrillation Screening With Mobil	https://doi.org/10.1177%2F107424	No	Pilot, feasibility, or proof-of-concept study
KardiaMobile	Feasibility of Commercially Marketed Health Devices for Potential Clinical Application		No	Pilot, feasibility, or proof-of-concept study
KardiaMobile	Feasibility of using mobile ECG recording technology to detect atrial fibrillation in low-r		No	Pilot, feasibility, or proof-of-concept study
KardiaMobile	High burden of unrecognized atrial fibrillation in rural India: an innovative community-l		No	Not a clinical trial studying efficacy or effectiveness for the included SaMD with outpatient use
KardiaMobile	Illustrating clinical relevance in the preclerkship medical school curriculum through act		No	Not a clinical trial studying efficacy or effectiveness for the included SaMD with outpatient use
KardiaMobile	Integration of novel monitoring devices with machine learning technology for scalable		No	Editorial article, Letter, Systematic Review, Abstract or Protocol
KardiaMobile	iPhone ECG application for community screening to detect silent atrial fibrillation: a no		No	Editorial article, Letter, Systematic Review, Abstract or Protocol

KardiaMobile	iPhone ECG screening by practice nurses and receptionists for atrial fibrillation in gene		No	Pilot, feasibility, or proof-of-concept study
KardiaMobile	Lead-I ECG for detecting atrial fibrillation in patients with an irregular pulse using single		No	Editorial article, Letter, Systematic Review, Abstract or Protocol
KardiaMobile	Living with the handheld ECG.		No	Editorial article, Letter, Systematic Review, Abstract or Protocol
KardiaMobile	Mobile Electrocardiogram Monitoring and Health-Related Quality of Life in Patients Wi		Yes	Trial of an SaMD device for at least two weeks
KardiaMobile	Mobile health applications for the detection of atrial fibrillation: a systematic review		No	Editorial article, Letter, Systematic Review, Abstract or Protocol
KardiaMobile	Mobile phones in cryptogenic strOke patients Bringing sIngle Lead ECGs for Atrial Fibril		No	Pilot, feasibility, or proof-of-concept study
KardiaMobile	Modified positioning of a smartphone based single-lead electrocardiogram device impr		No	Not a clinical trial studying efficacy or effectiveness for the included SaMD with outpatient use
KardiaMobile	Monitoring patients with implantable cardioverter defibrillators using mobile phone el		No	Not a clinical trial studying efficacy or effectiveness for the included SaMD with outpatient use
KardiaMobile	Multi-centre randomised controlled trial of a smartphone-based event recorder alongs		Yes	Trial of an SaMD device for at least two weeks
KardiaMobile	NICE guidance: lead-I ECG devices for detecting symptomatic atrial fibrillation using sin		No	Editorial article, Letter, Systematic Review, Abstract or Protocol
KardiaMobile	Opportunistic atrial fibrillation screening and detection in "self-service health check-up		No	Editorial article, Letter, Systematic Review, Abstract or Protocol
KardiaMobile	Opportunistic screening for atrial fibrillation by clinical pharmacists in UK general pract		No	Pilot, feasibility, or proof-of-concept study
KardiaMobile	Opportunistic screening to detect atrial fibrillation in Aboriginal adults in Australia.		No	Not a clinical trial studying efficacy or effectiveness for the included SaMD with outpatient use
KardiaMobile	Performance of handheld electrocardiogram devices to detect atrial fibrillation in a car		No	Not a clinical trial studying efficacy or effectiveness for the included SaMD with outpatient use
KardiaMobile	Pharmacy-based screening for atrial fibrillation in high-risk Maori and Pacific populatio		No	Editorial article, Letter, Systematic Review, Abstract or Protocol
KardiaMobile	Population screening for atrial fibrillation by student pharmacists at health fairs		No	Not a clinical trial studying efficacy or effectiveness for the included SaMD with outpatient use
KardiaMobile	Prospective blinded evaluation of smartphone-based ECG for differentiation of suprave		No	Not a clinical trial studying efficacy or effectiveness for the included SaMD with outpatient use
KardiaMobile	Prospective blinded Evaluation of the smartphone-b	https://doi.org/10.1016/j.ejim.2019.	No	Not a clinical trial studying efficacy or effectiveness for the included SaMD with outpatient use
KardiaMobile	Raising awareness and early detection of atrial fibrillation, an experience resorting to r		No	Not a clinical trial studying efficacy or effectiveness for the included SaMD with outpatient use
KardiaMobile	Rationale and design of the Atrial Fibrillation health Literacy Information Technology Tı		No	Pilot, feasibility, or proof-of-concept study
KardiaMobile	Recurrent atrial fibrillation/flutter detection after ablation or cardioversion using the A		Yes	Trial of an SaMD device for at least two weeks
KardiaMobile	Rise of the smart device ECG and what it means for the general cardiologist.		No	Not a clinical trial studying efficacy or effectiveness for the included SaMD with outpatient use
KardiaMobile	Safety and compatibility of smart device heart rhythm monitoring in patients with card		No	Less than two week study duration
KardiaMobile	Screening for atrial fibrillation during influenza vaccinations by primary care nurses usi		No	Not a clinical trial studying efficacy or effectiveness for the included SaMD with outpatient use
KardiaMobile	Screening for atrial fibrillation in 13,122 Hong Kong citizens with smartphone electroca		No	Less than two week study duration
KardiaMobile	Screening for atrial fibrillation in high-risk nursing home residents		No	Not a clinical trial studying efficacy or effectiveness for the included SaMD with outpatient use
KardiaMobile	Screening for atrial fibrillation is feasible in US managed care outpatient facilities.		No	Editorial article, Letter, Systematic Review, Abstract or Protocol
KardiaMobile	Screening for atrial fibrillation using a mobile, single-lead electrocardiogram in Canadia		No	Not a clinical trial studying efficacy or effectiveness for the included SaMD with outpatient use
KardiaMobile	Screening for Atrial Fibrillation Using a Smartphone-Based Electrocardiogram in Korean		No	Not a clinical trial studying efficacy or effectiveness for the included SaMD with outpatient use
KardiaMobile	Screening for Atrial Fibrillation Using Economical an	https://doi.org/10.1016/j.amjcard.2(	No	Less than two week study duration
KardiaMobile	Screening for atrial fibrillation: a call for evidence		No	Editorial article, Letter, Systematic Review, Abstract or Protocol
KardiaMobile	Searching for Atrial Fibrillation Poststroke		No	Editorial article, Letter, Systematic Review, Abstract or Protocol
KardiaMobile	Self-monitoring for atrial fibrillation recurrence in the discharge period post-cardiac su		No	Pilot, feasibility, or proof-of-concept study
KardiaMobile	Single-lead ECGs (AliveCor) are a feasible, cost-effective and safer alternative to 12-lea		No	Not a clinical trial studying efficacy or effectiveness for the included SaMD with outpatient use
KardiaMobile	Smart wearable devices in cardiovascular care: where we are and how to move forwar		No	Editorial article, Letter, Systematic Review, Abstract or Protocol
KardiaMobile	Smart Wearables for Cardiac Monitoring-Real-World Use beyond Atrial Fibrillation.		No	Editorial article, Letter, Systematic Review, Abstract or Protocol
KardiaMobile	Smartphone ECG aids real time diagnosis of palpitations in the competitive college athl		No	Editorial article, Letter, Systematic Review, Abstract or Protocol
KardiaMobile	Smartphone electrocardiogram for detecting atrial fibrillation after a cerebral ischaemi		Yes	Trial of an SaMD device for at least two weeks
KardiaMobile	Smartphone electrocardiographic monitoring for atrial fibrillation in acute ischemic stro		No	Not a clinical trial studying efficacy or effectiveness for the included SaMD with outpatient use
KardiaMobile	Supraventricular tachycardia diagnosed by smartphone ECG.		No	Not a clinical trial studying efficacy or effectiveness for the included SaMD with outpatient use
KardiaMobile	The Atrial Fibrillation Health Literacy Information Technology Trial: Pilot Trial of a Mobi		No	Not a clinical trial studying efficacy or effectiveness for the included SaMD with outpatient use
kardiamobile	The Danish Future Patient Telerehabilitation Program for Patients With Atrial Fibrillatic		No	Pilot, feasibility, or proof-of-concept study
KardiaMobile	The effectiveness of a mobile ECG device in identifying AF: sensitivity, specificity, and p		No	Not a clinical trial studying efficacy or effectiveness for the included SaMD with outpatient use
KardiaMobile	The efficacy of a smartphone ECG application for cardiac screening in an unselected isl		No	Less than two week study duration
KardiaMobile	The Heart Rhythm Society/American College of Physicians Atrial Fibrillation Screening a		No	Not a clinical trial studying efficacy or effectiveness for the included SaMD with outpatient use
KardiaMobile	Ubiquitous wireless ECG recording: a powerful tool physicians should embrace.		No	Editorial article, Letter, Systematic Review, Abstract or Protocol
KardiaMobile	Uptake of a primary care atrial fibrillation screening program (AF-SMART): a realist eva		No	Not a clinical trial studying efficacy or effectiveness for the included SaMD with outpatient use
KardiaMobile	Use of Smart Technology for the Early Diagnosis of Complications After Cardiac Surger		No	Editorial article, Letter, Systematic Review, Abstract or Protocol
KardiaMobile	Usefulness of Mobile Electrocardiographic Devices to Reduce Urgent Healthcare Visits		No	Not a clinical trial studying efficacy or effectiveness for the included SaMD with outpatient use
KardiaMobile	Using a novel wireless system for monitoring patients after the atrial fibrillation ablatic		No	Not a clinical trial studying efficacy or effectiveness for the included SaMD with outpatient use
KardiaMobile	Using mobile ECG devices to increase detection of atrial fibrillation across a range of se		No	Less than two week study duration
KardiaMobile	Using smart technology to improve outcomes in myocardial infarction patients: rationa		No	Pilot, feasibility, or proof-of-concept study
KardiaMobile	Validation of a smartphone-based event recorder for arrhythmia detection.		No	Not a clinical trial studying efficacy or effectiveness for the included SaMD with outpatient use
KardiaMobile	Wearable devices for cardiac rhythm diagnosis and management.		No	Editorial article, Letter, Systematic Review, Abstract or Protocol
KardiaMobile	Wide complex tachycardia recorded with a smartphone cardiac rhythm monitor.		No	Not a clinical trial studying efficacy or effectiveness for the included SaMD with outpatient use
Kardiamobile (	Atrial fibrillation (AF) pilot screening programme in pr	10.1136/bmjopen-2021-054324	No	Editorial article, Letter, Systematic Review, Abstract or Protocol
Kardiamobile (	Cardiac symptom burden and arrhythmia recurrence	10.1093/eurjcn/zvab009	No	Not a clinical trial studying efficacy or effectiveness for the included SaMD with outpatient use
Kardiamobile (	Diagnostic Accuracy of a Smartphone-Operated, Sin	10.1370/afm.2438	No	Not a clinical trial studying efficacy or effectiveness for the included SaMD with outpatient use
Kardiamobile (	Improving care for patients with atrial fibrillation throu	10.1097/JXX.0000000000000550	No	Not a clinical trial studying efficacy or effectiveness for the included SaMD with outpatient use
Kardiamobile (	Initial Experience in Monitoring QT Intervals Using a	10.19102/icrm.2021.120301	No	Pilot, feasibility, or proof-of-concept study
Kardiamobile (	Manual QT interval measurement with a smartphone-	10.1136/bmjopen-2021-055072	No	Not a clinical trial studying efficacy or effectiveness for the included SaMD with outpatient use
Kardiamobile (	Mobile Single-Lead Electrocardiogram Technology fo	10.3390/jcm11030665	No	Not a clinical trial studying efficacy or effectiveness for the included SaMD with outpatient use
Kardiamobile (	Multi-centre randomised controlled trial of a smart ph	10.1186/s13063-018-3098-1	No	Editorial article, Letter, Systematic Review, Abstract or Protocol
Kardiamobile (	Multi-centre Randomised Controlled Trial of a Smartp	10.1016/j.eclinm.2019.02.005	No	Not a clinical trial studying efficacy or effectiveness for the included SaMD with outpatient use
Kardiamobile (	Performance and Integration of Smartphone Wireless	10.1177/11795476211069194	No	Pilot, feasibility, or proof-of-concept study
Kardiamobile (	Performance of a Mobile Single-Lead Electrocardiogı	10.2196/24470	No	Not a clinical trial studying efficacy or effectiveness for the included SaMD with outpatient use
Kardiamobile (	Recurrent atrial fibrillation/flutter detection after ablati	10.1111/jce.14160	No	Not a clinical trial studying efficacy or effectiveness for the included SaMD with outpatient use
Kardiamobile (	Screening for Atrial Fibrillation in Older Adults at Prin	10.1161/CIRCULATIONAHA.121.0	No	Not a clinical trial studying efficacy or effectiveness for the included SaMD with outpatient use
Kardiamobile (	Screening for Atrial Fibrillation Using a Mobile, Single	10.1016/j.cjca.2019.03.024	No	Not a clinical trial studying efficacy or effectiveness for the included SaMD with outpatient use
Kardiamobile (	Smartphone electrocardiogram for detecting atrial fib	10.1093/europace/euab036	No	Not a clinical trial studying efficacy or effectiveness for the included SaMD with outpatient use
KardiaPro	A mobile one-lead ECG device incorporated in a sym	https://doi.org/10.1007/s12471-018	Yes	Trial of an SaMD device for at least two weeks
KardiaPro	At last, mobile health leading to a diagnosis in a you	https://doi.org/10.1007/s12471-018	No	Not a clinical trial studying efficacy or effectiveness for the included SaMD with outpatient use
KardiaPro	Atrial fibrillation future clinic. Novel platform to inte	https://doi.org/10.1016/j.cvdhj.202	No	Pilot, feasibility, or proof-of-concept study
KardiaPro	First real-world experience with mobile health telen	https://doi.org/10.1007/s12471-018	No	Pilot, feasibility, or proof-of-concept study
KardiaPro	Long-term intermittent versus short continuous hea	https://doi.org/10.1016/j.ijcard.202(	No	Not a clinical trial studying efficacy or effectiveness for the included SaMD with outpatient use
KardiaPro	The in-ear region as a novel anatomical site for ECG	https://doi.org/10.1007/s10840-02(	No	Less than two week study duration
Mahana IBS	A pilot feasibility study of an unguided, internet-delive	10.1111/nmo.14108	No	Pilot, feasibility, or proof-of-concept study
Mahana IBS	Assessing telephone-delivered cognitive-behavioural	10.1136/gutjnl-2018-317805	Yes	Trial of an SaMD device for at least two weeks
Mahana IBS	Cognitive behavioural therapy for irritable bowel synd	10.1016/S2468-1253(19)30243-2	Yes	Trial of an SaMD device for at least two weeks
Mahana IBS	Therapist telephone-delivered CBT and web-based C	10.3310/hta23170	Yes	Trial of an SaMD device for at least two weeks
Natural Cycles	(Not) talking about fertility: the role of digital technolo	http://dx.doi.org/10.1136/bmjsrh-20	No	Not a clinical trial studying efficacy or effectiveness for the included SaMD with outpatient use
Natural Cycles	Advantages of determining the fertile window with the	https://doi.org/10.1080/13625187.2	No	Not a clinical trial studying efficacy or effectiveness for the included SaMD with outpatient use
Natural Cycles	Association Between Menstrual Cycle Length and Co	https://doi.org/10.1097/AOG.0000(	No	Not a clinical trial studying efficacy or effectiveness for the included SaMD with outpatient use
Natural Cycles	Contraceptive Effectiveness of an FDA-Cleared Birth	10.1089/jwh.2020.8547	Yes	Trial of an SaMD device for at least two weeks
Natural Cycles	Detecting variations in ovulation and menstruation du	https://doi.org/10.1371/journal.pon	No	Not a clinical trial studying efficacy or effectiveness for the included SaMD with outpatient use
Natural Cycles	Fertility and digital technology: narratives of using sm	10.1111/1467-9566.13199	No	Not a clinical trial studying efficacy or effectiveness for the included SaMD with outpatient use
Natural Cycles	Fertility awareness-based mobile application for conti	https://doi.org/10.3109/13625187.2	Yes	Trial of an SaMD device for at least two weeks
Natural Cycles	Identification and prediction of the fertile window u	https://doi.org/10.3109/13625187.2	No	Not a clinical trial studying efficacy or effectiveness for the included SaMD with outpatient use
Natural Cycles	Natural Cycles app: contraceptive outcomes and den	https://doi.org/10.1080/13625187.2	Yes	Trial of an SaMD device for at least two weeks
Natural Cycles	Perfect-use and typical-use Pearl Index of a contrace	10.1016/j.contraception.2017.08.0	Yes	Trial of an SaMD device for at least two weeks
Natural Cycles	Real-world menstrual cycle characteristics of more th	https://doi.org/10.1038/s41746-019	No	Not a clinical trial studying efficacy or effectiveness for the included SaMD with outpatient use
Natural Cycles	Short- and long-term effect of contraceptive methods	https://doi.org/10.1080/13625187.2	No	Not a clinical trial studying efficacy or effectiveness for the included SaMD with outpatient use
Natural Cycles	Time to Pregnancy for Women Using a Fertility Awar	10.1089/jwh.2021.0026	No	Not a clinical trial studying efficacy or effectiveness for the included SaMD with outpatient use

Natural Cycles	Typical use effectiveness of Natural Cycles: postmar	http://dx.doi.org/10.1136/bmjopen-	Yes	Trial of an SaMD device for at least two weeks
Nightware	Efficacy of Blended Collaborative Care for Patients W	10.1001/jamainternmed.2021.497	No	Not a clinical trial studying efficacy or effectiveness for the included SaMD with outpatient use
Nightware	Enhancing behavioral sleep care with digital technolo	10.1186/s13063-020-04974-z	No	Editorial article, Letter, Systematic Review, Abstract or Protocol
Nightware	The Military Service Sleep Assessment: an instrumer	10.5664/jcsm.9206	No	Not a clinical trial studying efficacy or effectiveness for the included SaMD with outpatient use
Omron Model E	Accuracy and feasibility of portable blood pressure m	10.31083/j.rcm.2021.01.802	No	Pilot, feasibility, or proof-of-concept study
Omron Model E	Comparison of Microlife BP A200 Plus and Omron M	10.1007/s12325-011-0087-0	No	Not a clinical trial studying efficacy or effectiveness for the included SaMD with outpatient use
Omron Model E	Comparison of the Microlife blood pressure monitor w	10.1016/j.amjcard.2014.07.016	No	Not a clinical trial studying efficacy or effectiveness for the included SaMD with outpatient use
Omron Model E	Detection of Atrial Fibrillation Using a Home Blood Pr	10.2147/VHRM.S317859	No	Not a clinical trial studying efficacy or effectiveness for the included SaMD with outpatient use
Omron Model E	Diagnostic Value of Atrial Fibrillation by Built-in Elect	https://doi.org/10.1253/circrep.CR-	No	Not a clinical trial studying efficacy or effectiveness for the included SaMD with outpatient use
Omron Model E	Effect of Home Blood Pressure Monitoring via a Sma	10.1001/jamanetworkopen.2020.0	No	Not a clinical trial studying efficacy or effectiveness for the included SaMD with outpatient use
Omron Model E	Electrocardiogram-assisted blood pressure estimatio	10.1109/TBME.2011.2180019	No	Pilot, feasibility, or proof-of-concept study
Omron Model E	Feasibility of a randomized hypertension screening initiative in the perioperative settin		No	Not a clinical trial studying efficacy or effectiveness for the included SaMD with outpatient use
Omron Model E	Self-monitoring of blood pressure in hypertension: A	10.1371/journal.pmed.1002389	No	Editorial article, Letter, Systematic Review, Abstract or Protocol
Omron Model E	Triage tests for identifying atrial fibrillation in primary	10.1136/bmjopen-2013-004565	No	Not a clinical trial studying efficacy or effectiveness for the included SaMD with outpatient use
Omron Model E	Validation of the OMRON M6 AC (HEM-7322-E) upper arm blood pressure monitor, in		No	Not a peer reviewed article
Omron Model E	Validation of two automatic devices for the self-measurement of blood pressure accor		No	Not a clinical trial studying efficacy or effectiveness for the included SaMD with outpatient use
Omron Model E	Validation of two watch-type wearable blood pressure monitors according to the ANSI/		No	Pilot, feasibility, or proof-of-concept study
Mahana IBS	Assessing Cognitive behavioural Therapy in Irritable	http://dx.doi.org/10.1136/bmjopen-	No	Editorial article, Letter, Systematic Review, Abstract or Protocol
Mahana IBS	Exploring Patients' Views of a Cognitive Behavioral T	https://doi.org/10.2196/jmir.2672	No	Pilot, feasibility, or proof-of-concept study
Mahana IBS	Management of irritable bowel syndrome in primary c	https://doi.org/10.1186/1471-230X-	No	Editorial article, Letter, Systematic Review, Abstract or Protocol
Proteus	A digital health solution for using and managing medications: wirelessly observed thera		No	Not a clinical trial studying efficacy or effectiveness for the included SaMD with outpatient use
Proteus	Developing a Digital Medicine System in Psychiatry: Ir	doi: 10.4088/JCP.16m10643	No	Not a clinical trial studying efficacy or effectiveness for the included SaMD with outpatient use
Proteus	Digital medicines: clinical review on the safety of tabl	10.1080/14740338.2018.1508447	No	Not a clinical trial studying efficacy or effectiveness for the included SaMD with outpatient use
Proteus	Effectiveness of Digital Medicines to Improve Clinica	10.2196/jmir.7833	No	Pilot, feasibility, or proof-of-concept study
Proteus	Feasibility of an ingestible sensor-based system for r	10.1371/journal.pone.0053373	No	Pilot, feasibility, or proof-of-concept study
Proteus	First experience with a wireless system incorporating	doi: 10.4088/JCP.12m08222	No	Not a clinical trial studying efficacy or effectiveness for the included SaMD with outpatient use
Proteus	Human factors evaluation of a novel digital medicine	10.2147/NDT.S157102	No	Not a clinical trial studying efficacy or effectiveness for the included SaMD with outpatient use
Proteus	Hummingbird Study: a study protocol for a multicentr	doi: 10.1136/bmjopen-2018-02595	No	Editorial article, Letter, Systematic Review, Abstract or Protocol
Proteus	Let Visuals Tell the Story: Medication Adherence in Patients with Type II Diabetes Capt		No	Not a clinical trial studying efficacy or effectiveness for the included SaMD with outpatient use
Proteus	Medication adherence assessment: high accuracy of	doi: 10.1097/TP.0b013e31829b75	No	Not a clinical trial studying efficacy or effectiveness for the included SaMD with outpatient use
Proteus	Mobile technology and the digitization of healthcare		No	Not a clinical trial studying efficacy or effectiveness for the included SaMD with outpatient use
Proteus	Optimization of a Digital Medicine System in Psychiat	doi: 10.4088/JCP.16m10693	No	Editorial article, Letter, Systematic Review, Abstract or Protocol
Proteus	Patient-Centered Home Care Using Digital Medicine	10.1111/jch.12787	No	Pilot, feasibility, or proof-of-concept study
Proteus	Pharmacokinetics of Coencapsulated Antiretrovirals	10.1089/AID.2019.0202	No	Not a clinical trial studying efficacy or effectiveness for the included SaMD with outpatient use
Proteus	Pharmacokinetics of tenofovir monoester and associ	10.1093/jac/dkz187	No	Not a clinical trial studying efficacy or effectiveness for the included SaMD with outpatient use
Proteus	Role for direct electronic verification of pharmaceutic	10.1016/j.cct.2012.03.008	No	Editorial article, Letter, Systematic Review, Abstract or Protocol
Proteus	Short Communication: Bioequivalence of Tenofovir ar	doi: 10.1089/AID.2018.0081	No	Not a clinical trial studying efficacy or effectiveness for the included SaMD with outpatient use
Proteus	Usability of a novel digital medicine system in adults	10.2147/NDT.S116029	No	Not a clinical trial studying efficacy or effectiveness for the included SaMD with outpatient use
Proteus	Wirelessly observed therapy compared to directly obs	10.1371/journal.pmed.1002891	No	Not a clinical trial studying efficacy or effectiveness for the included SaMD with outpatient use
Proteus	Wirelessly Observed Therapy to Optimize Adherence	10.2196/15532	No	Pilot, feasibility, or proof-of-concept study
Regulora	AGA technical review on irritable bowel syndrome	https://doi.org/10.1053/gast.2002.3	No	Not a clinical trial studying efficacy or effectiveness for the included SaMD with outpatient use
Regulora	An ultra-brief screening scale for anxiety and depres	https://doi.org/10.1176/appi.psy.50	No	Not a clinical trial studying efficacy or effectiveness for the included SaMD with outpatient use
Regulora	Assessing Cognitive behavioural Therapy in Irritable	10.1136/bmjopen-2015-008622	No	Editorial article, Letter, Systematic Review, Abstract or Protocol
Regulora	Blinding assessment in clinical trials: A review of stat	https://doi.org/10.3109/10601331	No	Not a clinical trial studying efficacy or effectiveness for the included SaMD with outpatient use
Regulora	Disseminating hypnosis to health care settings: Appl	https://doi.org/10.1037/cns00000	No	Not a clinical trial studying efficacy or effectiveness for the included SaMD with outpatient use
Regulora	Effect of hypnotherapy and educational intervention	https://doi.org/10.1111/apt.12319	No	Not a clinical trial studying efficacy or effectiveness for the included SaMD with outpatient use
Regulora	Efficacy of individual and group hypnotherapy in irrita	https://doi.org/10.1016/s2468-125	No	Not a clinical trial studying efficacy or effectiveness for the included SaMD with outpatient use
Regulora	Global prevalence of and risk factors for irritable bow	https://doi.org/10.1016/j.cgh.2012.	No	Not a clinical trial studying efficacy or effectiveness for the included SaMD with outpatient use
Regulora	Hypnosis Treatment of Gastrointestinal Disorders: A	https://doi.org/10.1080/00029157.	No	Not a clinical trial studying efficacy or effectiveness for the included SaMD with outpatient use
Regulora	Impact of irritable bowel syndrome: prevalence and effect on health-related quality of life		No	Not a clinical trial studying efficacy or effectiveness for the included SaMD with outpatient use
Regulora	Improvement in Gastrointestinal Symptoms After Co	https://doi.org/10.1053/j.gastro.201	No	Not a clinical trial studying efficacy or effectiveness for the included SaMD with outpatient use
Regulora	Long term benefits of hypnotherapy for irritable bowe	https://doi.org/10.1136/gut.52.11.1	No	Not a clinical trial studying efficacy or effectiveness for the included SaMD with outpatient use
Regulora	Management of irritable bowel syndrome in primary c	10.1186/1471-230X-13-68	No	Pilot, feasibility, or proof-of-concept study
Regulora	Out-of-illness experience: hypnotically induced disso	https://doi.org/10.1176/appi.psych	No	Not a clinical trial studying efficacy or effectiveness for the included SaMD with outpatient use
Regulora	Pain management in inflammatory bowel disease: fe	10.1186/s40814-021-00829-9	No	Pilot, feasibility, or proof-of-concept study
Regulora	Patients' Experiences of Telephone-Based and Web-	10.2196/18691	No	Not a clinical trial studying efficacy or effectiveness for the included SaMD with outpatient use
Regulora	Quality of life in persons with irritable bowel syndrom	https://doi.org/10.1023/a:10188311	No	Not a clinical trial studying efficacy or effectiveness for the included SaMD with outpatient use
Regulora	Relaxation training as a treatment for irritable bowel	https://doi.org/10.1007/bf0099978	No	Not a clinical trial studying efficacy or effectiveness for the included SaMD with outpatient use
Regulora	Standardized hypnosis treatment for irritable bowel s	https://doi.org/10.1080/00207140	No	Not a clinical trial studying efficacy or effectiveness for the included SaMD with outpatient use
Regulora	Stool form scale as a useful guide to intestinal transit	https://doi.org/10.3109/00365529	No	Not a clinical trial studying efficacy or effectiveness for the included SaMD with outpatient use
Regulora	The brain-gut axis in abdominal pain syndromes	https://doi.org/10.1146/annurev-m	No	Not a clinical trial studying efficacy or effectiveness for the included SaMD with outpatient use
Regulora	The economic consequences of irritable bowel syndr	https://doi.org/10.1001/archinte.16	No	Not a clinical trial studying efficacy or effectiveness for the included SaMD with outpatient use
Regulora	The effects of hypnosis on dissociative identity disor	https://doi.org/10.1177/07067437	No	Not a clinical trial studying efficacy or effectiveness for the included SaMD with outpatient use
Regulora	The Efficacy, Safety and Applications of Medical Hyp	https://doi.org/10.3238/arztebl.201	No	Not a clinical trial studying efficacy or effectiveness for the included SaMD with outpatient use
Regulora	The role of psychosocial factors in functional gastroir	https://doi.org/10.1159/000356785	No	Not a clinical trial studying efficacy or effectiveness for the included SaMD with outpatient use
Regulora	The validity and reproducibility of a work productivity	https://doi.org/10.2165/00019053-1	NO	Not a clinical trial studying efficacy or effectiveness for the included SaMD with outpatient use
Regulora	Validation of the Rome III criteria for the diagnosis of	https://doi.org/10.1053/j.gastro.201	NO	Not a clinical trial studying efficacy or effectiveness for the included SaMD with outpatient use
Regulora	Variation in Care for Patients with Irritable Bowel Syn	https://doi.org/10.1371/journal.pon	NO	Not a clinical trial studying efficacy or effectiveness for the included SaMD with outpatient use
Regulora	What is hypnosis and how might it work?	https://doi.org/10.1177/11782242	NO	Not a clinical trial studying efficacy or effectiveness for the included SaMD with outpatient use
Reset, Reset-C	Adding an Internet-delivered treatment to an efficaci	https://content.apa.org/doi/10.1037	Yes	Trial of an SaMD device for at least two weeks
Reset, Reset-C	Approaches for Implementing App-Based Digital Tre	10.2196/25866	No	Not a clinical trial studying efficacy or effectiveness for the included SaMD with outpatient use
Reset, Reset-C	Comparison of Healthcare Resource Utilization Betw	10.2147/CEOR.S334274	No	Not a clinical trial studying efficacy or effectiveness for the included SaMD with outpatient use
Reset, Reset-C	Computerized Cognitive Behavioral Therapy for Inso	https://doi.org/10.5664/jcsm.6460	Yes	Trial of an SaMD device for at least two weeks
Reset, Reset-C	Cost-Effectiveness Analysis of a Prescription Digital	10.1080/20016689.2021.1966187	No	Not a clinical trial studying efficacy or effectiveness for the included SaMD with outpatient use
Reset, Reset-C	Effect of a Web-Based Cognitive Behavior Therapy f	doi.org/10.1001/jamapsychiatry.20	Yes	Trial of an SaMD device for at least two weeks
Reset, Reset-C	Evaluation of the cost-utility of a prescription digital t	10.1080/00325481.2021.1884471	No	Not a clinical trial studying efficacy or effectiveness for the included SaMD with outpatient use
Reset, Reset-C	Evidence of long-term real-world reduction in health	10.1080/14737167.2021.1939687	No	Editorial article, Letter, Systematic Review, Abstract or Protocol
Reset, Reset-C	Internet-Delivered Treatment for Substance Abuse: A	https://doi.org/10.1176/appi.ajp.20	Yes	Trial of an SaMD device for at least two weeks
Reset, Reset-C	Patient Engagement With a Game-Based Digital The	10.2196/32759	No	Editorial article, Letter, Systematic Review, Abstract or Protocol
Reset, Reset-C	Profile of Somryst Prescription Digital Therapeutic fo	10.1080/17434440.2020.1852929	No	Editorial article, Letter, Systematic Review, Abstract or Protocol
Reset, Reset-C	Protocol for Digital Real-world Evidence trial for Adul	10.2217/cer-2021-0004	No	Editorial article, Letter, Systematic Review, Abstract or Protocol
Reset, Reset-C	Real-world changes in US health system hospital-ba	10.1080/21548331.2021.1956256	No	Not a clinical trial studying efficacy or effectiveness for the included SaMD with outpatient use
Reset, Reset-C	Real-world evidence for a prescription digital therape	10.1080/03007995.2020.1846023	Yes	Trial of an SaMD device for at least two weeks
Reset, Reset-C	Real-world reduction in healthcare resource utilizatio	10.1080/14737167.2021.1840357	No	Not a clinical trial studying efficacy or effectiveness for the included SaMD with outpatient use
Reset, Reset-C	Real-world use and clinical outcomes after 24 weeks	10.1080/21548331.2021.1974243	Yes	Trial of an SaMD device for at least two weeks
Reset, Reset-C	Safety and efficacy of a prescription digital therapeut	10.1080/03007995.2020.1846022	Yes	Trial of an SaMD device for at least two weeks
Samsung ECG	FDA-Cleared Electrocardiogram Monitoring App is Available Starting Today on Galaxy V		No	Not a peer reviewed article
Samsung ECG	Samsung Health Monitor Application (ECG App) Instructions for Use		No	Not a peer reviewed article
Samsung ECG	The Use of Samsung Health and ECG M-Trace Base	10.3390/ijerph18115753	No	Not a clinical trial studying efficacy or effectiveness for the included SaMD with outpatient use
Samsung ECG	The Use of Samsung Health and ECG M-Trace Base II Applications in the Secondary Pre		No	Not a clinical trial studying efficacy or effectiveness for the included SaMD with outpatient use

Product	Document Title	DOI	Final Decision	Reason
Apple Irregular Rhythm N	Large-Scale Assessment of a Smartwatch to Identify Atrial Fil	DOI: 10.1056/N	Yes	Trial of an SaMD device for at least two weeks
Bluestar	Cluster-randomized trial of a mobile phone personalized behavioral interventi		Yes	Trial of an SaMD device for at least two weeks
Bluestar	Engagement and Outcomes Associated with Contextual Ann	10.1177/19322	Yes	Trial of an SaMD device for at least two weeks
Bluestar	Glucose Self-monitoring in Non–Insulin-Treated Patients With Type 2 Diabete		No	Not a clinical trial studying efficacy or effectiveness for the included SaMD with outpatient use
Bluestar	Lessons from a community-based mHealth diabetes self-mar	DOI: 10.1080/1	No	Not a clinical trial studying efficacy or effectiveness for the included SaMD with outpatient use
Bluestar	Mobile App for Improved Self-Management of Type 2 Diabete	DOI: 10.2196/1	Yes	Trial of an SaMD device for at least two weeks
Bluestar	WellDoc mobile diabetes management randomized controllec	10.1089/dia.20	No	Not a clinical trial studying efficacy or effectiveness for the included SaMD with outpatient use
Clue	Estimating six-cycle efficacy of the Dot app for pregnancy pre	10.1016/j.contr	Yes	Trial of an SaMD device for at least two weeks
Clue	Perfect- and typical-use effectiveness of the Dot fertility app c	doi: 10.1080/13	Yes	Trial of an SaMD device for at least two weeks
Dexcom G6; Dexcom Pro	Mitigation of Rebound Hyperglycemia With Real-Time Contin	10.1177/19322	No	Not a clinical trial studying efficacy or effectiveness for the included SaMD with outpatient use
Dexcom G6; Dexcom Pro	Real-World Evidence and Glycemic Improvement Using Dex	10.1089/dia.20	Yes	Trial of an SaMD device for at least two weeks
Dexcom G6; Dexcom Pro	Real-World Hypoglycemia Avoidance with a Continuous Gluc	10.1089/dia.20	No	Not a clinical trial studying efficacy or effectiveness for the included SaMD with outpatient use
Dexcom G6; Dexcom Pro	Real-World Hypoglycemia Avoidance With a Predictive Low (	10.1177/19322	No	Not a clinical trial studying efficacy or effectiveness for the included SaMD with outpatient use
EndeavorRX	A novel digital intervention for actively reducing severity of	https://doi.org/1	Yes	Trial of an SaMD device for at least two weeks
EndeavorRX	Effectiveness of a digital therapeutic as adjunct to treatment	https://doi.org/1	Yes	Trial of an SaMD device for at least two weeks
EndeavorRx	Enhancing neural markers of attention in children with ADHD using a digital		Yes	Trial of an SaMD device for at least two weeks
KardiaMobile	Assessment of remote heart rhythm sampling using the AliveCor heart mon		No	Not a clinical trial studying efficacy or effectiveness for the included SaMD with outpatient use
KardiaMobile	Mobile Electrocardiogram Monitoring and Health-Related Quality of Life in I		No	Not a clinical trial studying efficacy or effectiveness for the included SaMD with outpatient use
KardiaMobile	Multi-centre randomised controlled trial of a smartphone-based event reco		No	Not a clinical trial studying efficacy or effectiveness for the included SaMD with outpatient use
KardiaMobile	Recurrent atrial fibrillation/flutter detection after ablation or cardioversion		No	Not a clinical trial studying efficacy or effectiveness for the included SaMD with outpatient use
KardiaMobile	Smartphone electrocardiogram for detecting atrial fibrillation after a cerebral i		No	Not a clinical trial studying efficacy or effectiveness for the included SaMD with outpatient use
KardiaMobile	Diagnostic utility of real-time smartphone ECG in the initial investigation of		No	Not a clinical trial studying efficacy or effectiveness for the included SaMD with outpatient use
KardiaPro	A mobile one-lead ECG device incorporated in a symptom-d	https://doi.org/1	No	Not a clinical trial studying efficacy or effectiveness for the included SaMD with outpatient use
Mahana IBS	Assessing telephone-delivered cognitive-behavioural therapy	10.1136/gutjnl-	Yes	Trial of an SaMD device for at least two weeks
Mahana IBS	Cognitive behavioural therapy for irritable bowel syndrome: 2	10.1016/S2468	Yes	Trial of an SaMD device for at least two weeks
Mahana IBS	Therapist telephone-delivered CBT and web-based CBT com	10.3310/hta231	Yes	Trial of an SaMD device for at least two weeks
Natural Cycles	Contraceptive Effectiveness of an FDA-Cleared Birth Control	10.1089/jwh.20	Yes	Trial of an SaMD device for at least two weeks
Natural Cycles	Fertility awareness-based mobile application for contraceptio	https://doi.org/1	Yes	Trial of an SaMD device for at least two weeks
Natural Cycles	Natural Cycles app: contraceptive outcomes and demographi	https://doi.org/1	Yes	Trial of an SaMD device for at least two weeks
Natural Cycles	Perfect-use and typical-use Pearl Index of a contraceptive m	10.1016/j.contr	Yes	Trial of an SaMD device for at least two weeks
Natural Cycles	Typical use effectiveness of Natural Cycles: postmarket surv	http://dx.doi.org	Yes	Trial of an SaMD device for at least two weeks
Reset, Reset-O, Somryst	Adding an Internet-delivered treatment to an efficacious treat	https://content.	Yes	Trial of an SaMD device for at least two weeks
Reset, Reset-O, Somryst	Computerized Cognitive Behavioral Therapy for Insomnia in a	https://doi.org/1	No	Not a clinical trial studying efficacy or effectiveness for the included SaMD with outpatient use
Reset, Reset-O, Somryst	Effect of a Web-Based Cognitive Behavior Therapy for Insom	doi.org/10.1001	Yes	Trial of an SaMD device for at least two weeks
Reset, Reset-O, Somryst	Internet-Delivered Treatment for Substance Abuse: A Multisit	https://doi.org/1	Yes	Trial of an SaMD device for at least two weeks
Reset, Reset-O, Somryst	Real-world evidence for a prescription digital therapeutic to tr	10.1080/03007	Yes	Trial of an SaMD device for at least two weeks
Reset, Reset-O, Somryst	Real-world use and clinical outcomes after 24 weeks of treatr	10.1080/21548	Yes	Trial of an SaMD device for at least two weeks
Reset, Reset-O, Somryst	Safety and efficacy of a prescription digital therapeutic as an	10.1080/03007	Yes	Trial of an SaMD device for at least two weeks
