# Supplementary material for: Collection and Analysis of Adherence Information for Software as a Medical Device Clinical Trials: Systematic Review
Source: JMIR Mhealth Uhealth. 2023 Nov 15;11:e46237. doi: 10.2196/46237 (PMC10687688; doi:10.2196/46237)
Supplement: Multimedia Appendix 3 [file mhealth_v11i1e46237_app3.pdf]

Study ID

Agarwal 2019
Akturk 2021
Bull 2019
Campbell 2014
Christensen 2014
Dugas 2020
Everitt 2019a
Everitt 2019b
Everitt 2019c
Gallen 2021
Jennings 2018
Jennings 2019
Kollins 2020
Kollins 2021
Maricich 2021a
Maricich 2021b
Maricich 2021c
Pearson 2021 a
Pearson 2021 b
Perez 2019
Quinn 2011
Ritterband 2017
Scherwitzl 2016
Scherwitzl 2017

Blank Sheet

Article Key

**Criteria for Adherence Metrics and Analysis**		**Notes**
**Device name and indication for use**	Bluestar (self-management of diabetes)	
**Type of device** (Long-term vs short-term use)	Long-term use	
**Type of device** (Presence or absence of a prescribed dosage)	Absence of a prescribed dose	
**Was adherence information collected?**	Yes	
**What information was collected about usage adherence?**	Daily use of app<br>Use of each app feature such as blood glucose tracking and exercise tracking per week<br>Mean number of engagements per week	
**What information was collected about adherence to recommendations?**	NA	No explicit app recommendations
**Was information collected internally in the app?**	Yes	
**Was information modified internally?**	NA	
**Was initiation reported?**	No	
**What was average adherence?** (if reported)		
**Was implementation reported?**	Yes	
**What was average adherence?** (if reported)	Mean number of login days was 42.4 (SD 52.1) over a 26 week period<br>46.4% of participants used the app 10 days or less<br>18.2% used the app 100 days or more	
**Was persistence reported?**	No	The study plotted the mean use of the app over time which combines implementation and persistence
**Was adherence low?**	Yes	On average participants logged into the app on 23% of days during the trial period
**Was adherence not reported or low and could have affected outcomes?**	Yes	
**What was average adherence?** (if reported)		
**Was adherence information analyzed?**	Yes	
**What method was used?**	Dose-response model	General linearized models acounting for baseline variables
**Were efficacy analyses preregistered?**	No	
**What assumptions are required for that method to study efficacy?**	SUTVA, positivity, consistency, conditional independence of adherence and outcomes	
**Did the article report evidence that the assumptions were met?**	Probably not	Unclear how confounder was chosen or if controlling for one confounder was sufficient
**SUTVA**	Yes	Care was primarily provided through the app
**Positivity**	Yes	Guaranteed by randomization
**Consistency** (treatment definition)	Yes	Clear treatment definition
**Consistency** (adherence definition)		
**Exclusion restriction**		
**Strong Monotonicity**		
**Ignorability**	Baseline A1c	
**Overall Notes**	They note that adherence was likely low due to the app being on a second phone. Reporting of persistence could be improved by separating this measure from implementation.	
**Cochrane CDPLG**		**Notes**
**Data form completed date** (dd/mm/yyyy)	3/23/2022	
**Study author contact details**	payal.agarwal@wchospital.ca	
Methods	**Descriptions as stated in report/paper**	**Location in text or source** (pg & ¶/fig/table/other)
Aim of study (e.g. efficacy, equivalence, pragmatic)	Effectiveness	pg. 2 Trial Design
Design (e.g. parallel, crossover, non-RCT)	Wait-list randomized controlled trial	pg. 1 Abstract; pg. 2 Trial Design
Unit of allocation (by individuals, cluster/ groups or body parts)	Individual	pg. 2 Trial Design
Participants	**Descriptions as stated in report/paper**	**Location in text or source** (pg & ¶/fig/table/other)
**Inclusion criteria**	"(1) adults aged older than 18 years<br>(2) obtaining care for T2DM at a participating DEP<br>(3) HbA1c ≥8.0% (and at least 1% above the participant's target level) on most recent laboratory report within the last 3 months<br>(4) currently using an active email address or able and willing to obtain one<br>(5) able to read the English language (self-reported)."	pg. 3 Participants
**Exclusion criteria**	"(1) have type 1 diabetes<br>(2) were on continuous glucose monitoring<br>(3) had an insulin pump<br>(4) were on dialysis, pregnant, or are unable to use a computer or mobile phone because of severe mental or physical impairment"	pg. 3 Participants
**Total no. randomised** (or total pop. at start of study for NRCTs)	240	pg. 5 Figure 1
**Clusters** (if applicable, no., type, no. people per cluster)	NA	
**Withdrawals and exclusions** (if not provided below by outcome)	17 excluded before allocation due to missing A1c or too low A1c	pg. 5 Figure 1
Intervention Groups	**Descriptions as stated in report/paper**	**Location in text or source** (pg & ¶/fig/table/other)
**Group name**	Immediate Treatment Group (ITG)	pg. 1 Abstract; pg. 2 Trial Design
**No. randomised to group** (specify whether no. people or clusters)	110	pg. 5 Figure 1
**Timing** (e.g. frequency, duration of each episode)	No recommended timing for app use listed	
**Co-interventions**	Participants were given a samsung phone without other apps to use the app<br>Providers were able to see data for the app	pg. 3 Intervention
**Integrity of delivery**	No issues of integrity of delivery reported.<br>Unclear how many participants initiated use of app.	
**Compliance**	46.4% of ITG participants used the app 10 days or less in a 26 week time frame (primary outcome tested at 14 weeks)	pg. 8 Mobile App Utilization and Satisfaction
Intervention Groups	**Descriptions as stated in report/paper**	**Location in text or source** (pg & ¶/fig/table/other)
**Group name**	Wait-list Control Group (WLC)	pg. 1 Abstract; pg. 2 Trial Design
**No. randomised to group** (specify whether no. people or clusters)	113	pg. 5 Figure 1
**Timing** (e.g. frequency, duration of each episode)	NA	
**Co-interventions**	None	
**Integrity of delivery**	No issues of integrity of delivery reported.	
**Compliance**	NA	
Outcomes	**Descriptions as stated in report/paper**	**Location in text or source** (pg & ¶/fig/table/other)
**Outcome name**	HbA1c at 3 months	pg. 1 Abstract
**Outcome definition** (with diagnostic criteria if relevant)(include name, time, and analysis method)	Difference in HbA1c between ITG and WLC at 3 months analyzed using ANCOVA and ITT analysis	pg. 3 Statistical Analysis
**Imputation of missing data** (e.g. assumptions made for ITT analysis)	Inclusion of complete cases<br>Sensitivity analysis identified predictors of missing data and included those in the model	pg. 3 Statistical Analysis
**Power** (e.g. power & sample size calculation, level of power achieved)	"Power was determined assuming an ANCOVA analysis with an estimate correlation between baseline and follow-up HbA1c measurements of 0.80. The power to detect a difference of 0.7% in HbA1c levels using an SD of 2% between treatment groups at 3 months is 99.7% at a significance level of 5%, based on a sample size of 255 (which assumes a dropout rate of 15% from the target sample size of 300 participants)."<br>Compared to the initial goal, the study was underpowered.	pg. 4 Statistical Analysis
**Risk of Bias** (Based on Cochrane RoB Tool 2 and ROBINS-I)		Link to RoB Algorithm
**Primary Analysis RoB Assessment**		Notes
**Is the primary analysis evaluating effectiveness or efficacy?**	Effectiveness	
**Comparator**	ITG compared to WLC	
**Outcome Being Assessed**	A1c at 3 months (ITT effect analyzed with ANCOVA)	
**Specify the Numerical Result Being Assessed**	"mean difference [ITG-WLC] -0.42, 95% CI -1.05 to 0.21; p=0.19"	
**Bias due to randomization (RCT only)**		Notes
1.1 Was the allocation sequence random?	Yes	
1.2. Was the allocation sequence concealed until participants were enrolled and assigned to interventions?	Yes	

1.3. Did baseline differences between intervention groups suggest a problem with the randomization process?	No	
**Bias due to confounding**		Notes
1.1 Is there potential for confounding of the effect of intervention in this study?	Probably No	ITT analysis for RCT design
1.2. If Y/PY to 1.1 Was the analysis based on splitting participants' follow up time according to intervention received?		
1.3. If Y/PY to 1.2 Were intervention discontinuations or switches likely to be related to factors that are prognostic for the outcome?		
**Questions relating to baseline confounding only**		
1.4. If Y/PY to 1.1 Did the authors use an appropriate analysis method that controlled for all the important confounding domains?		Baseline values were controlled for
1.5. If Y/PY to 1.4: Were confounding domains that were controlled for measured validly and reliably by the variables available in this study?		
1.6. Did the authors control for any post-intervention variables that could have been affected by the intervention?		
**Questions relating to baseline and time-varying confounding**		
1.7. If Y/PY to 1.3 Did the authors use an appropriate analysis method that controlled for all the important confounding domains and for time-varying confounding?		
1.8. If Y/PY to 1.7: Were confounding domains that were controlled for measured validly and reliably by the variables available in this study?		
**Bias in selection of participants into the study (Not evaluating for analyses of effectiveness)**		Notes
2.1. Was selection of participants into the analysis based on participant characteristics observed after the start of intervention?		
2.2. If Y/PY to 2.1: Were the post-intervention variables that influenced selection likely to be associated with intervention?		
2.3 If Y/PY to 2.2: Were the post-intervention variables that influenced selection likely to be influenced by the outcome or a cause of the outcome?		
2.4. Do start of follow-up and start of intervention coincide for most participants?		
2.5. If Y/PY to 2.2 and 2.3, or N/PN to 2.4: Were adjustment techniques used that are likely to correct for the presence of selection biases?		
**Bias in classification of interventions**		Notes
3.1 Were intervention groups clearly defined?	Yes	Intervention groups were clearly defined
3.2 Was the information used to define intervention groups recorded at the start of the intervention?	Yes	Preregistered trial
3.3 Could classification of intervention status have been affected by knowledge of the outcome or risk of the outcome?	No	Preregistered trial
**Bias due to deviations from intended interventions**		Notes
**ROBINS-I**		
4.1. Were there deviations from the intended intervention beyond what would be expected in usual practice?	No	
4.2. If Y/PY to 4.1: Were these deviations from intended intervention unbalanced between groups and likely to have affected the outcome?		
4.3. Were important co-interventions balanced across intervention groups?	Yes	I don't think the cell phone without access to other apps would have had a large effect
4.4. Was the intervention implemented successfully for most participants?	No information	
4.5. Did study participants adhere to the assigned intervention regimen?	No	While we don't know how adherence looked for the 14 week period specifically, we do know that half of the treatment participants used the app for less than 10 days<br>On average participants logged into the app on 23% of days during the trial period
4.6. If N/PN to 4.3, 4.4 or 4.5: Was an appropriate analysis used to estimate the effect of starting and adhering to the intervention?	No	They did adjust for baseline A1c but to do a dose response model properly they would have had to
Risk of bias: Assignment to intervention	**Low** / Moderate / Serious / Critical / NI	
Risk of bias: Starting and adhering to intervention	Low / Moderate / Serious / **Critical** / NI	
**Risk of bias judgement**	Low / Moderate / Serious / **Critical** / NI	
**RoB Tool (Risk of bias due to deviations from the intended interventions (effect of adhering to intervention))**		
2.1. Were participants aware of their assigned intervention during the trial?	Yes	
2.2. Were carers and people delivering the interventions aware of participants' assigned intervention during the trial?	Yes	
2.3. If Y/PY/NI to 2.1 or 2.2: Were important non protocol interventions balanced across intervention groups?	NA	
2.4. [If applicable:] Were there failures in implementing the intervention that could have affected the outcome?	No information	
2.5. [If applicable:] Was there non-adherence to the assigned intervention regimen that could have affected participants' outcomes?	Yes	While we don't know how adherence looked for the 14 week period specifically, we do know that half of the treatment participants used the app for less than 10 days
2.6. If N/PN/NI to 2.3, or Y/PY/NI to 2.4 or 2.5: Was an appropriate analysis used to estimate the effect of adhering to the intervention?	No	They did adjust for baseline A1c but to do a dose response model properly they would have had to account for confounders of adherence or use an IV model and with either method they needed a large sample size. This analysis was not preregistered.
**Risk of bias judgement**	Low/**High**/Some Concerns	
**Bias due to missing data**		Notes
5.1 Were outcome data available for all, or nearly all, participants?	No	65.5% had a 3 month A1c value
5.2 Were participants excluded due to missing data on intervention status?	No	
5.3 Were participants excluded due to missing data on other variables needed for the analysis?	No	
5.4 If PN/N to 5.1, or Y/PY to 5.2 or 5.3: Are the proportion of participants and reasons for missing data similar across interventions?	Yes	
5.5 If PN/N to 5.1, or Y/PY to 5.2 or 5.3: Is there evidence that results were robust to the presence of missing data?	Yes	No significant baseline differences were found between participants with complete or incomplete 3 month A1c information.
**Bias in measurement of outcomes**		Notes
6.1 Could the outcome measure have been influenced by knowledge of the intervention received?	No	
6.2 Were outcome assessors aware of the intervention received by study participants? (blinding of assessors)	No	
6.3 Were the methods of outcome assessment comparable across intervention groups?	Yes	
6.4 Were any systematic errors in measurement of the outcome related to intervention received?	No	
**Bias in selection of the reported result**		Notes
Were the data that produced this result analysed in accordance with a pre-specified analysis plan that was finalized before unblinded outcome data were available for analysis?	Yes	https://bmcmedinformdecismak.biomedcentral.com/articles/10.1186/s12911-016-0381-5
Is the reported effect estimate likely to be selected, on the basis of the results, from...		
7.1. ... multiple outcome measurements within the outcome domain?	No	
7.2 ... multiple analyses of the intervention-outcome relationship?	No	
7.3 ... different subgroups?	No	
**Secondary Analysis RoB Assessment**		Notes
**Is the seconday analysis evaluating effectiveness or efficacy?**	Efficacy	
**Comparator**	Treatment group only	

**Outcome Being Assessed**	Dose-response of app use in treatment group	Generalized linear model accounting for baseline variables
**Specify the Numerical Result Being Assessed**	"each additional day of app use corresponded with a 0.016-point decrease in participants' 3-month HbA1c levels (95% CI −0.03 to −0.003; P=0.02)"	
**Bias due to randomization (See primary analysis section)**		Notes
**Bias due to confounding**		Notes
1.1 Is there potential for confounding of the effect of intervention in this study?	Yes	Only the treatment group was included in this analysis. They could have used an IV method since they had a randomized controlled trial design.
1.2. If Y/PY to 1.1 Was the analysis based on splitting participants' follow up time according to intervention received?	No	
1.3. If Y/PY to 1.2 Were intervention discontinuations or switches likely to be related to factors that are prognostic for the outcome?		
**Questions relating to baseline confounding only**		
1.4. If Y/PY to 1.1 Did the authors use an appropriate analysis method that controlled for all the important confounding domains?	No	They controlled for baseline A1c levels but didn't analyze what variables had an impact on adherence. They did determine that site and when participants were diagnosed with diabetes had an impact on usage but these were not incorporated into the analysis.
1.5. If Y/PY to 1.4: Were confounding domains that were controlled for measured validly and reliably by the variables available in this study?	Yes	
1.6. Did the authors control for any post-intervention variables that could have been affected by the intervention?		
**Questions relating to baseline and time-varying confounding**		
1.7. If Y/PY to 1.3 Did the authors use an appropriate analysis method that controlled for all the important confounding domains and for time-varying confounding?		
1.8. If Y/PY to 1.7: Were confounding domains that were controlled for measured validly and reliably by the variables available in this study?		
**Bias in selection of participants into the study (Not evaluating for analyses of effectiveness)**		Notes
2.1. Was selection of participants into the analysis based on participant characteristics observed after the start of intervention?	Probably Yes	57/69 participants with 3 month A1c values were included in the analysis
2.2. If Y/PY to 2.1: Were the post-intervention variables that influenced selection likely to be associated with intervention?	No information	
2.3 If Y/PY to 2.2: Were the post-intervention variables that influenced selection likely to be influenced by the outcome or a cause of the outcome?		
2.4. Do start of follow-up and start of intervention coincide for most participants?	Yes	
2.5. If Y/PY to 2.2 and 2.3, or N/PN to 2.4: Were adjustment techniques used that are likely to correct for the presence of selection biases?	No	Only the treatment group was included in the analysis and it was not clear if the chosen confounders sufficiently addressed selection bias
**Bias in classification of interventions**		Notes
3.1 Were intervention groups clearly defined?	Yes	Looked at a daily dose, not clear if this was associated with a prescribed dosage to use the app
3.2 Was the information used to define intervention groups recorded at the start of the intervention?	No	The daily dosage pattern was not predefined
3.3 Could classification of intervention status have been affected by knowledge of the outcome or risk of the outcome?	Yes	Different dosage patterns could have been analyzed
**Bias due to deviations from intended interventions (See primary analysis section)**		Notes
**Bias due to missing data**		Notes
5.1 Were outcome data available for all, or nearly all, participants?	No	57/110 included, 57/223 if you consider the control group. Only complete cases included which mean that the participant had a baseline and 3 month A1c value
5.2 Were participants excluded due to missing data on intervention status?	No	
5.3 Were participants excluded due to missing data on other variables needed for the analysis?	Yes	12 participants who had 3 month values were excluded since they didn't have baseline values
5.4 If PN/N to 5.1, or Y/PY to 5.2 or 5.3: Are the proportion of participants and reasons for missing data similar across interventions?	No	Only the treatment group was included
5.5 If PN/N to 5.1, or Y/PY to 5.2 or 5.3: Is there evidence that results were robust to the presence of missing data?	No	
**Bias in measurement of outcomes**		Notes
6.1 Could the outcome measure have been influenced by knowledge of the intervention received?	No	
6.2 Were outcome assessors aware of the intervention received by study participants? (blinding of assessors)	No	
6.3 Were the methods of outcome assessment comparable across intervention groups?	Yes	
6.4 Were any systematic errors in measurement of the outcome related to intervention received?	No	
6.5 Could adherence have been influenced by study participation?	Probably No	The study did not try to influence adherence
**Bias in selection of the reported result**		Notes
Were the data that produced this result analysed in accordance with a pre-specified analysis plan that was finalized before unblinded outcome data were available for analysis?	No	
Is the reported effect estimate likely to be selected, on the basis of the results, from...		
7.1. ... multiple outcome measurements within the outcome domain?	No	This was not preregistered so it is impossible to know if this was the outcome of multiple analyses or definitions of adherence
7.2 ... multiple analyses of the intervention-outcome relationship?	Yes	This was not preregistered so it is impossible to know if this was the outcome of multiple analyses or definitions of adherence
7.3 ... different subgroups?	Yes	

Article Key

Criteria for Adherence Metrics and Analysis		Notes
**Device name and indication for use**	CLARITY analytics software (Dexcom G6) for diabetes management	
**Type of device** (Long-term vs short-term use)	Long-term use	
**Type of device** (Presence or absence of a prescribed dosage)	Absence of a prescribed dosage	
**Was adherence information collected?**	Yes	
**What information was collected about usage adherence?**	Daily information about if CLARITY was used to process patient data	
**What information was collected about adherence to recommendations?**	NA	No explicit app recommendations
**Was information collected internally in the app?**	Yes	
**Was information modified internally?**	NA	
**Was initiation reported?**	Yes	
**What was average adherence?** (if reported)	92.2% of the population used CLARITY at least once (not the same population as the analysis (US based patients who uploaded data in October 2019)); 38% did not use clarity, 30% used the web based reports, 32% used the web based reports and notifications	
**Was implementation reported?**	No	The study looked at different levels of initiating the CLARITY feature but not at use of those features
**What was average adherence?** (if reported)		
**Was persistence reported?**	No	
**Was adherence low?**	NA	
**Was adherence not reported or low?**	Yes	
**What was average adherence?** (if reported)		
**Was efficacy analyzed?**	Yes	Compared initiation of feature use to outcomes
**What method was used?**	As-treated analysis	
**Were efficacy analyses preregistered?**	No	
**What assumptions are required for that method to study efficacy?**	SUTVA, positivity, consistency, ignorability, conditional independence of adherence and outcomes	
**Did the article report evidence that the assumptions were met?**	No	Participants could have used other features more often as well, there wasn't random assignment or a random mechanism of non compliance and there were no controls for confounders
**SUTVA**	Yes	
**Positivity**	Yes	Participants likely had a non-zero chance of using the app feature
**Consistency** (treatment definition)	No	Consistency likely didn't hold since they could have used other features more often as well meaning that there could be hidden forms of treatment
**Consistency** (adherence definition)	No	Consistency likely didn't hold since they could have used other features more often as well meaning that there could be hidden forms of treatment
**Exclusion restriction**		
**Strong Monotonicity**		
**Ignorability**	None	
**Overall Notes**	The article states that there is only a claim of correlation and not causation but estimates could have been improved by prespecifying the analysis and controlling for confounders	
**Cochrane CDPLG**		**Notes**
**Data form completed date** (dd/mm/yyyy)	3/31/2022	
**Study author contact details**	halis.akturk@cuanschutz.edu	
Methods	**Descriptions as stated in report/paper**	**Location in text or source** (pg & ¶/fig/table/other)
Aim of study (e.g. efficacy, equivalence, pragmatic)	Efficacy of app feature	pg. 1 Abstract
Design (e.g. parallel, crossover, non-RCT)	Retrospective cohort study	pg. 1 Abstract
Unit of allocation (by individuals, cluster/ groups or body parts)	Individual	
Participants	**Descriptions as stated in report/paper**	**Location in text or source** (pg & ¶/fig/table/other)
**Inclusion criteria**	"US-based patients who uploaded data in October 2019"	pg. 3 CLARITY Notifications
**Exclusion criteria**		
**Total no. randomised** (or total pop. at start of study for NRCTs)	2637 users	pg. 4 CLARITY Notifications
**Clusters** (if applicable, no., type, no. people per cluster)	NA	
**Withdrawals and exclusions** (if not provided below by outcome)	NA	
Intervention Groups	**Descriptions as stated in report/paper**	**Location in text or source** (pg & ¶/fig/table/other)
**Group name**	Group 1: Users who never accessed the CLARITY feature	pg. 3 CLARITY Notifications
**No. randomised to group** (specify whether no. people or clusters)	1000	pg. 4 Table 3
**Timing** (e.g. frequency, duration of each episode)		
**Co-interventions**	Access to the G6 CGM system	pg. 2 Introduction
**Integrity of delivery**	Participants were included in the cohort based upload of data (not CLARITY use)	pg. 3 CLARITY Notifications
**Compliance**	All participants would not have accessed the CLARITY at all	
Intervention Groups	**Descriptions as stated in report/paper**	**Location in text or source** (pg & ¶/fig/table/other)
**Group name**	Group 2: Users who accessed the web-based reports but declined to receive automated notifications	pg. 3 CLARITY Notifications
**No. randomised to group** (specify whether no. people or clusters)	794	pg. 4 CLARITY Notifications
**Timing** (e.g. frequency, duration of each episode)		
**Co-interventions**	Access to the G6 CGM system	pg. 2 Introduction
**Integrity of delivery**	Participants were included in the cohort based on use of CLARITY	pg. 3 CLARITY Notifications
**Compliance**	No information	
Intervention Groups	**Descriptions as stated in report/paper**	**Location in text or source** (pg & ¶/fig/table/other)
**Group name**	Group 3: Users who accessed web-based reports and opted into receiving all automated notifications	pg. 3 CLARITY Notifications
**No. randomised to group** (specify whether no. people or clusters)	843	pg. 4 CLARITY Notifications
**Timing** (e.g. frequency, duration of each episode)		
**Co-interventions**	Access to the G6 CGM system	pg. 2 Introduction
**Integrity of delivery**	Participants were included in the cohort based on use of CLARITY	pg. 3 CLARITY Notifications
**Compliance**	No information	
Outcomes	**Descriptions as stated in report/paper**	**Location in text or source** (pg & ¶/fig/table/other)
**Outcome name**	Percent of patients meeting consensus goals (>70% of values 70-180 mg/dL; <25% of values >180 mg/dL; <5% of values >250 mg/dL; <4% of values <70 mg/dL; <1% of values <54 mg/dL)	pg. 4 CLARITY Notifications
**Outcome definition** (with diagnostic criteria if relevant)(include name, time, and analysis method)	Percent of patients in group 2 and 3 who met their consensus goals over a six month time period in comparison to group 1 with two proportion z tests	pg. 4 CLARITY Notifications
**Imputation of missing data** (e.g. assumptions made for ITT analysis)	None	
**Power** (e.g. power & sample size calculation, level of power achieved)		
**Risk of Bias** (Based on Cochrane RoB Tool 2 and ROBINS-I)		Link to RoB Algorithm
**Primary Analysis RoB Assessment**		Notes
**Is the primary analysis evaluating effectiveness or efficacy?**	Efficacy	
**Comparator**	Group 2 compared to Group 1; Group 3 compared to Group 1	
**Outcome Being Assessed**	Percent of time in various ranges and percent of patients meeting consensus goals	Likely over a 6 month period
**Specify the Numerical Result Being Assessed**	Table 3 (e.g. Group 2 had 56.4% (20.9%of values in the 70-180 mg/dL range compared to Group 1 who had 52.8% (24.5%) and p<0.1)	
**Bias due to randomization (RCT only)**		Notes

1.1 Was the allocation sequence random?	NA	
1.2. Was the allocation sequence concealed until participants were enrolled and assigned to interventions?	NA	
1.3. Did baseline differences between intervention groups suggest a problem with the randomization process?	NA	
**Bias due to confounding**		Notes
1.1 Is there potential for confounding of the effect of intervention in this study?	Yes	
1.2. If Y/PY to 1.1 Was the analysis based on splitting participants' follow up time according to intervention received?	No	
1.3. If Y/PY to 1.2 Were intervention discontinuations or switches likely to be related to factors that are prognostic for the outcome?		
**Questions relating to baseline confounding only**		
1.4. If Y/PY to 1.1 Did the authors use an appropriate analysis method that controlled for all the important confounding domains?	No	No confounders were accounted for despite the observational design of the study
1.5. If Y/PY to 1.4: Were confounding domains that were controlled for measured validly and reliably by the variables available in this study?		
1.6. Did the authors control for any post-intervention variables that could have been affected by the intervention?	No	
**Questions relating to baseline and time-varying confounding**		
1.7. If Y/PY to 1.3 Did the authors use an appropriate analysis method that controlled for all the important confounding domains and for time-varying confounding?		
1.8. If Y/PY to 1.7: Were confounding domains that were controlled for measured validly and reliably by the variables available in this study?		
**Bias in selection of participants into the study (Not evaluating for analyses of effectiveness)**		Notes
2.1. Was selection of participants into the analysis based on participant characteristics observed after the start of intervention?	Yes	
2.2. If Y/PY to 2.1: Were the post-intervention variables that influenced selection likely to be associated with intervention?	Yes	Participants were likely more likely to use CLARITY if they regularly used other app features
2.3 If Y/PY to 2.2: Were the post-intervention variables that influenced selection likely to be influenced by the outcome or a cause of the outcome?	Yes	It is likely that factors influencing someone to use CLARITY would have affected the decision to adhere to other diabetes care regimens
2.4. Do start of follow-up and start of intervention coincide for most participants?	Probably Yes	
2.5. If Y/PY to 2.2 and 2.3, or N/PN to 2.4: Were adjustment techniques used that are likely to correct for the presence of selection biases?	No	
**Bias in classification of interventions**		Notes
3.1 Were intervention groups clearly defined? Was the definition of adherence clearly defined?	Probably No	Participants could have used other features as well and this was not captured by their definition of treatment
3.2 Was the information used to define intervention groups recorded at the start of the intervention?	No	The analysis was not preregistered
3.3 Could classification of intervention status have been affected by knowledge of the outcome or risk of the outcome?	Yes	The analysis was not preregistered
**Bias due to deviations from intended interventions**		Notes
**ROBINS-I**		
4.1. Were there deviations from the intended intervention beyond what would be expected in usual practice?	No	
4.2. If Y/PY to 4.1: Were these deviations from intended intervention unbalanced between groups and likely to have affected the outcome?		
4.3. Were important co-interventions balanced across intervention groups?	Probably No	Participants in each study group likely used the CGM system in different ways
4.4. Was the intervention implemented successfully for most participants?	Yes	All participants needed to upload data in October 2019 to be included in the study
4.5. Did study participants adhere to the assigned intervention regimen?	No information	
4.6. If N/PN to 4.3, 4.4 or 4.5: Was an appropriate analysis used to estimate the effect of starting and adhering to the intervention?	No	
Risk of bias: Assignment to intervention	**Low** / Moderate / Serious / Critical / NI	
Risk of bias: Starting and adhering to intervention	Low / Moderate / Serious / **Critical** / NI	
**Risk of bias judgement**	Low / Moderate / Serious / **Critical** / NI	
**RoB Tool (Risk of bias due to deviations from the intended interventions (effect of adhering to intervention))**		
2.1. Were participants aware of their assigned intervention during the trial?	Yes	
2.2. Were carers and people delivering the interventions aware of participants' assigned intervention during the trial?	Yes	
2.3. If Y/PY/NI to 2.1 or 2.2: Were important non protocol interventions balanced across intervention groups?	NA	
2.4. [If applicable:] Were there failures in implementing the intervention that could have affected the outcome?	No	
2.5. [If applicable:] Was there non-adherence to the assigned intervention regimen that could have affected participants' outcomes?	No information	
2.6. If N/PN/NI to 2.3, or Y/PY/NI to 2.4 or 2.5: Was an appropriate analysis used to estimate the effect of adhering to the intervention?	No	The required assumptions to use this method were not met
**Risk of bias judgement**	Low/**High**/Some Concerns	
**Bias due to missing data**		Notes
5.1 Were outcome data available for all, or nearly all, participants?	Yes	
5.2 Were participants excluded due to missing data on intervention status?	No	
5.3 Were participants excluded due to missing data on other variables needed for the analysis?	No	
5.4 If PN/N to 5.1, or Y/PY to 5.2 or 5.3: Are the proportion of participants and reasons for missing data similar across interventions?		
5.5 If PN/N to 5.1, or Y/PY to 5.2 or 5.3: Is there evidence that results were robust to the presence of missing data?		
**Bias in measurement of outcomes**		Notes
6.1 Could the outcome measure have been influenced by knowledge of the intervention received?	No	Objective blood glucose measurement from the CGM system
6.2 Were outcome assessors aware of the intervention received by study participants? (blinding of assessors)	No	
6.3 Were the methods of outcome assessment comparable across intervention groups?	Yes	
6.4 Were any systematic errors in measurement of the outcome related to intervention received?	No	
**Bias in selection of the reported result**		Notes
Were the data that produced this result analysed in accordance with a pre-specified analysis plan that was finalized before unblinded outcome data were available for analysis?	No	
Is the reported effect estimate likely to be selected, on the basis of the results, from...		
7.1. ... multiple outcome measurements within the outcome domain?	Probably yes	Study was not preregistered so there could have been many outcomes compared
7.2 ... multiple analyses of the intervention-outcome relationship?	Probably yes	Study was not preregistered so there could have been many analyses considered
7.3 ... different subgroups?	Probably yes	Study was not preregistered so there could have been many subgroups considered
**Secondary Analysis RoB Assessment**		Notes
**Is the seconday analysis evaluating effectiveness or efficacy?**		
**Comparator**		
**Outcome Being Assessed**		
**Specify the Numerical Result Being Assessed**		

**Bias due to randomization (See primary analysis section)**		Notes
**Bias due to confounding**		Notes
1.1 Is there potential for confounding of the effect of intervention in this study?		
1.2. If Y/PY to 1.1 Was the analysis based on splitting participants' follow up time according to intervention received?		
1.3. If Y/PY to 1.2 Were intervention discontinuations or switches likely to be related to factors that are prognostic for the outcome?		
**Questions relating to baseline confounding only**		
1.4. If Y/PY to 1.1 Did the authors use an appropriate analysis method that controlled for all the important confounding domains?		
1.5. If Y/PY to 1.4: Were confounding domains that were controlled for measured validly and reliably by the variables available in this study?		
1.6. Did the authors control for any post-intervention variables that could have been affected by the intervention?		
**Questions relating to baseline and time-varying confounding**		
1.7. If Y/PY to 1.3 Did the authors use an appropriate analysis method that controlled for all the important confounding domains and for time-varying confounding?		
1.8. If Y/PY to 1.7: Were confounding domains that were controlled for measured validly and reliably by the variables available in this study?		
**Bias in selection of participants into the study (Not evaluating for analyses of effectiveness)**		Notes
2.1. Was selection of participants into the analysis based on participant characteristics observed after the start of intervention?		
2.2. If Y/PY to 2.1: Were the post-intervention variables that influenced selection likely to be associated with intervention?		
2.3 If Y/PY to 2.2: Were the post-intervention variables that influenced selection likely to be influenced by the outcome or a cause of the outcome?		
2.4. Do start of follow-up and start of intervention coincide for most participants?		
2.5. If Y/PY to 2.2 and 2.3, or N/PN to 2.4: Were adjustment techniques used that are likely to correct for the presence of selection biases?		
**Bias in classification of interventions**		Notes
3.1 Were intervention groups clearly defined?		
3.2 Was the information used to define intervention groups recorded at the start of the intervention?		
3.3 Could classification of intervention status have been affected by knowledge of the outcome or risk of the outcome?		
**Bias due to deviations from intended interventions (See primary analysis section)**		Notes
**Bias due to missing data**		Notes
5.1 Were outcome data available for all, or nearly all, participants?		
5.2 Were participants excluded due to missing data on intervention status?		
5.3 Were participants excluded due to missing data on other variables needed for the analysis?		
5.4 If PN/N to 5.1, or Y/PY to 5.2 or 5.3: Are the proportion of participants and reasons for missing data similar across interventions?		
5.5 If PN/N to 5.1, or Y/PY to 5.2 or 5.3: Is there evidence that results were robust to the presence of missing data?		
**Bias in measurement of outcomes**		Notes
6.1 Could the outcome measure have been influenced by knowledge of the intervention received?		
6.2 Were outcome assessors aware of the intervention received by study participants? (blinding of assessors)		
6.3 Were the methods of outcome assessment comparable across intervention groups?		
6.4 Were any systematic errors in measurement of the outcome related to intervention received?		
6.5 Could adherence have been influenced by study participation?		
**Bias in selection of the reported result**		Notes
Were the data that produced this result analysed in accordance with a pre-specified analysis plan that was finalized before unblinded outcome data were available for analysis?		
Is the reported effect estimate likely to be selected, on the basis of the results, from...		
7.1. ... multiple outcome measurements within the outcome domain?		
7.2 ... multiple analyses of the intervention-outcome relationship?		
7.3 ... different subgroups?		

Criteria for Adherence Metrics and Analysis		Notes
**Device name and indication for use**	Natural Cycles (contraception)	
**Type of device** (Long-term vs short-term use)	Long-term use	
**Type of device** (Presence or absence of a prescribed dosage)	Presence of a prescribed dosage	
**Was adherence information collected?**	Yes	
**What information was collected about usage adherence?**	Days of data input	
**What information was collected about adherence to recommendations?**	Unprotected sex on red days	Reported as the percent of all days of Natural Cycles use
**Was information collected internally in the app?**	Yes	
**Was information modified internally?**	NA	
**Was initiation reported?**	Yes	Included participants needed to provide at least 20 data points to be includeed in the study
**What was average adherence?** (if reported)	100%	
**Was implementation reported?**	Yes	No information about daily temperature readings but info about sexual behavior
**What was average adherence?** (if reported)	Participants reported having sex on 1-3% of red days depending on their previous contraceptive use	
**Was persistence reported?**	No	
**What was average adherence?** (if reported)		
**Was adherence low?**	NA	Implementation was reported but not in a way that could be used to assess if adherence should be considered low
**Was adherence not reported or low?**	Yes	
**Was efficacy analyzed?**	No	
**What method was used?**	None	
**Were efficacy analyses preregistered?**	NA	
**What assumptions are required for that method to study efficacy?**		
**Did the article report evidence that the assumptions were met?**		
**SUTVA**		
**Positivity**		
**Consistency** (treatment definition)		
**Consistency** (adherence definition)		
**Exclusion restriction**		
**Strong Monotonicity**		
**Ignorability**		
**Overall Notes**	The study reports that women were typically able to measure their temperature at least five days a week but do not specify what typically means. This study does address one aspect of confounding, previous contraception use. They have multiple cohorts in this study say that additional research is needed to understand the impact of demographics and behavioural differences (frequency of sex, frequency of sex on red days). The study does not address condom use on green days.	
Cochrane CDPLG		Notes
**Data form completed date** (dd/mm/yyyy)	3/30/2022	
**Study author contact details**	raoul.scherwitzl@naturalcycles.com	
Methods	**Descriptions as stated in report/paper**	**Location in text or source** (pg & ¶/fig/table/other)
Aim of study (e.g. efficacy, equivalence, pragmatic)	Effectiveness moderated by previous contraceptive choice	pg. 1 Abstract
Design (e.g. parallel, crossover, non-RCT)	Real-world prospective observational study	pg. 2 Introduction
Unit of allocation (by individuals, cluster/ groups or body parts)	Individual	
Participants	**Descriptions as stated in report/paper**	**Location in text or source** (pg & ¶/fig/table/other)
**Inclusion criteria**	"all women who had registered as paying annual subscribers to Natural Cycles between 1st September 2016 and 30th October 2017 with the intent of preventing a pregnancy." "The women were using Natural Cycles as their primary method of contraception, resident in Sweden (determined from the postal address for annual subscribers), aged 18–45 and required to have entered at least 20 days of data (any combination of temperature, LH test, pregnancy test, sexual activity or personal notes) into the application"	pg. 3 Study Design
**Exclusion criteria**		
**Total no. randomised** (or total pop. at start of study for NRCTs)	16,331	pg. 4 Results
**Clusters** (if applicable, no., type, no. people per cluster)	NA	
**Withdrawals and exclusions** (if not provided below by outcome)	11365 participants were included in the comparison. The remainder were excluded because they had not provided adequate information about previous contraceptive use.	pg. 4 Table 1
Intervention Groups	**Descriptions as stated in report/paper**	**Location in text or source** (pg & ¶/fig/table/other)
**Group name**	Natural Cycles Cohort (grouped by previous contraceptive use)	pg. 2 Introduction
**No. randomised to group** (specify whether no. people or clusters)	16,331	pg. 4 Results
**Timing** (e.g. frequency, duration of each episode)	Daily measurement of basal body temperature upon waking<br>Optional luteinishing hormone tests<br>Alternative method of contraception or abstinence on red days	pg. 2 Introduction
**Co-interventions**		
**Integrity of delivery**	All women included needed to provide at least 20 data points (any combination of	pg. 3 Study Design
**Compliance**	No information about daily temperature readings; participants reported having sex on 1-3% of red days	Figure 5
Outcomes	**Descriptions as stated in report/paper**	**Location in text or source** (pg & ¶/fig/table/other)
**Outcome name**	Typical Use Pearl Index	pg. 4 Data Collection and Analysis
**Outcome definition** (with diagnostic criteria if relevant)(include name, time, and analysis method)	Risk of unintended pregnancy measured with the one year pearl index	pg. 4 Data Collection and Analysis
**Imputation of missing data** (e.g. assumptions made for ITT analysis)	The pregnancy status of users who were lost to follow-up was estimated from their temperature measurements and from the point in the cycle at which they dropped out.	pg. 3 Study Design
**Power** (e.g. power & sample size calculation, level of power achieved)		
**Risk of Bias** (Based on Cochrane RoB Tool 2 and ROBINS-I)		Link to RoB Algorithm
**Primary Analysis RoB Assessment**		Notes
**Is the primary analysis evaluating effectiveness or efficacy?**	Effectiveness	
**Comparator**	Pearl index of previous contraception	
**Outcome Being Assessed**	One year typical use pearl index based on previous contraception use	
**Specify the Numerical Result Being Assessed**	Table 2 (e.g. typical PI for those in the male condom cohort was 3.5 +/- 0.5 and the previous contraception PI was 18 leading to a 14.5 decrease in PI)	
**Bias due to randomization (RCT only)**		Notes
1.1 Was the allocation sequence random?	NA	
1.2. Was the allocation sequence concealed until participants were enrolled and assigned to interventions?	NA	
1.3. Did baseline differences between intervention groups suggest a problem with the randomization process?	NA	
**Bias due to confounding**		Notes
1.1 Is there potential for confounding of the effect of intervention in this study?	Yes	
1.2. If Y/PY to 1.1 Was the analysis based on splitting participants' follow up time according to intervention received?	No	
1.3. If Y/PY to 1.2 Were intervention discontinuations or switches likely to be related to factors that are prognostic for the outcome?		
**Questions relating to baseline confounding only**		

Article Key

1.4. If Y/PY to 1.1 Did the authors use an appropriate analysis method that controlled for all the important confounding domains?	Probably No	This study compared the effects of previous contraceptive use, they feel that age was balanced between the groups so that it didn't have an impact but it could still have been a confounder if there was an interaction between the age and previous method of contraception, they did investigate the effect of unprotected sex on red days compared to effectiveness; other confounders such as contraceptive use on green days and frequency of sex for all days were not accounted fo
1.5. If Y/PY to 1.4: Were confounding domains that were controlled for measured validly and reliably by the variables available in this study?	Yes	
1.6. Did the authors control for any post-intervention variables that could have been affected by the intervention?		
**Questions relating to baseline and time-varying confounding**		
1.7. If Y/PY to 1.3 Did the authors use an appropriate analysis method that controlled for all the important confounding domains and for time-varying confounding?		
1.8. If Y/PY to 1.7: Were confounding domains that were controlled for measured validly and reliably by the variables available in this study?		
**Bias in selection of participants into the study (Not evaluating for analyses of effectiveness)**		Notes
2.1. Was selection of participants into the analysis based on participant characteristics observed after the start of intervention?		
2.2. If Y/PY to 2.1: Were the post-intervention variables that influenced selection likely to be associated with intervention?		
2.3 If Y/PY to 2.2: Were the post-intervention variables that influenced selection likely to be influenced by the outcome or a cause of the outcome?		
2.4. Do start of follow-up and start of intervention coincide for most participants?		
2.5. If Y/PY to 2.2 and 2.3, or N/PN to 2.4: Were adjustment techniques used that are likely to correct for the presence of selection biases?		
**Bias in classification of interventions**		Notes
3.1 Were intervention groups clearly defined? Was the definition of adherence clearly defined?	Probably Yes	The groups are defined clearly but it is possible for there to be overlap between the groups (e.g. withdrawal and condom use)
3.2 Was the information used to define intervention groups recorded at the start of the intervention?	Yes	Participants were asked at the time of registration
3.3 Could classification of intervention status have been affected by knowledge of the outcome or risk of the outcome?	No	Participants were asked at the time of registration
**Bias due to deviations from intended interventions**		Notes
**ROBINS-I**		
4.1. Were there deviations from the intended intervention beyond what would be expected in usual practice?	No	
4.2. If Y/PY to 4.1: Were these deviations from intended intervention unbalanced between groups and likely to have affected the outcome?		
4.3. Were important co-interventions balanced across intervention groups?	No information	It is unclear if alternate forms of contraceptives were used on green days across the groups
4.4. Was the intervention implemented successfully for most participants?	Yes	Inclusion criteria required that you had to provide 20 data points
4.5. Did study participants adhere to the assigned intervention regimen?	Probably No	Non-adherence on red days was reported but the frequency of uploading data was not reported
4.6. If N/PN to 4.3, 4.4 or 4.5: Was an appropriate analysis used to estimate the effect of starting and adhering to the intervention?	No	The Pearl index was used to estimate the effect of perfect adherencein a previous study but this did not account for confounders of adherence
Risk of bias: Assignment to intervention	**Low** / Moderate / Serious / Critical / NI	
Risk of bias: Starting and adhering to intervention	Low / Moderate / Serious / **Critical** / NI	
**Risk of bias judgement**	Low / Moderate / Serious / **Critical** / NI	
**RoB Tool (Risk of bias due to deviations from the intended interventions (effect of adhering to intervention))**		
2.1. Were participants aware of their assigned intervention during the trial?	Yes	
2.2. Were carers and people delivering the interventions aware of participants' assigned intervention during the trial?	Yes	
2.3. If Y/PY/NI to 2.1 or 2.2: Were important non protocol interventions balanced across intervention groups?	NA	
2.4. [If applicable:] Were there failures in implementing the intervention that could have affected the outcome?	No	Inclusion criteria required that you had to provide 20 data points
2.5. [If applicable:] Was there non-adherence to the assigned intervention regimen that could have affected participants' outcomes?	Probably Yes	Non-adherence on red days was reported but the frequency of uploading data was not reported
2.6. If N/PN/NI to 2.3, or Y/PY/NI to 2.4 or 2.5: Was an appropriate analysis used to estimate the effect of adhering to the intervention?	No	The Pearl index was used to estimate the effect of perfect adherence in a prior study but this did not account for confounders of adherence and they did not specify who was excluded using this analysis method
**Risk of bias judgement**	Low/**High**/Some Concerns	
**Bias due to missing data**		Notes
5.1 Were outcome data available for all, or nearly all, participants?	Yes	1.3% of sample lost to follow-up
5.2 Were participants excluded due to missing data on intervention status?	No	
5.3 Were participants excluded due to missing data on other variables needed for the analysis?	Yes	Participants needed to answer a survey questions to be included in the sub cohort analysis
5.4 If PN/N to 5.1, or Y/PY to 5.2 or 5.3: Are the proportion of participants and reasons for missing data similar across interventions?	Probably yes	Similar percentages of women who answered yes or no to Q1
5.5 If PN/N to 5.1, or Y/PY to 5.2 or 5.3: Is there evidence that results were robust to the presence of missing data?	No	
**Bias in measurement of outcomes**		Notes
6.1 Could the outcome measure have been influenced by knowledge of the intervention received?	Probably No	
6.2 Were outcome assessors aware of the intervention received by study participants? (blinding of assessors)	Yes	
6.3 Were the methods of outcome assessment comparable across intervention groups?	Yes	
6.4 Were any systematic errors in measurement of the outcome related to intervention received?	No	
**Bias in selection of the reported result**		Notes
Were the data that produced this result analysed in accordance with a pre-specified analysis plan that was finalized before unblinded outcome data were available for	No	
Is the reported effect estimate likely to be selected, on the basis of the results, from...		
7.1. ... multiple outcome measurements within the outcome domain?	No	Only one measurement
7.2 ... multiple analyses of the intervention-outcome relationship?	Probably No	Same analysis method used in previous studies but this study was not preregistered
7.3 ... different subgroups?	Probably No	This study was not preregistered so it is possible that they analyzed many subgroups and reported this analysis since there was an effect

Secondary Analysis RoB Assessment		Notes
**Is the seconday analysis evaluating effectiveness or efficacy?**		
**Comparator**		
**Outcome Being Assessed**		
**Specify the Numerical Result Being Assessed**		
**Bias due to randomization (See primary analysis section)**		Notes
**Bias due to confounding**		Notes
1.1 Is there potential for confounding of the effect of intervention in this study?		
1.2. If Y/PY to 1.1 Was the analysis based on splitting participants' follow up time according to intervention received?		
1.3. If Y/PY to 1.2 Were intervention discontinuations or switches likely to be related to factors that are prognostic for the outcome?		
**Questions relating to baseline confounding only**		
1.4. If Y/PY to 1.1 Did the authors use an appropriate analysis method that controlled for all the important confounding domains?		
1.5. If Y/PY to 1.4: Were confounding domains that were controlled for measured validly and reliably by the variables available in this study?		
1.6. Did the authors control for any post-intervention variables that could have been affected by the intervention?		
**Questions relating to baseline and time-varying confounding**		
1.7. If Y/PY to 1.3 Did the authors use an appropriate analysis method that controlled for all the important confounding domains and for time-varying confounding?		
1.8. If Y/PY to 1.7: Were confounding domains that were controlled for measured validly and reliably by the variables available in this study?		
**Bias in selection of participants into the study (Not evaluating for analyses of effectiveness)**		Notes
2.1. Was selection of participants into the analysis based on participant characteristics observed after the start of intervention?		
2.2. If Y/PY to 2.1: Were the post-intervention variables that influenced selection likely to be associated with intervention?		
2.3 If Y/PY to 2.2: Were the post-intervention variables that influenced selection likely to be influenced by the outcome or a cause of the outcome?		
2.4. Do start of follow-up and start of intervention coincide for most participants?		
2.5. If Y/PY to 2.2 and 2.3, or N/PN to 2.4: Were adjustment techniques used that are likely to correct for the presence of selection biases?		
**Bias in classification of interventions**		Notes
3.1 Were intervention groups clearly defined?		
3.2 Was the information used to define intervention groups recorded at the start of the intervention?		
3.3 Could classification of intervention status have been affected by knowledge of the outcome or risk of the outcome?		
**Bias due to deviations from intended interventions (See primary analysis section)**		Notes
**Bias due to missing data**		Notes
5.1 Were outcome data available for all, or nearly all, participants?		
5.2 Were participants excluded due to missing data on intervention status?		
5.3 Were participants excluded due to missing data on other variables needed for the analysis?		
5.4 If PN/N to 5.1, or Y/PY to 5.2 or 5.3: Are the proportion of participants and reasons for missing data similar across interventions?		
5.5 If PN/N to 5.1, or Y/PY to 5.2 or 5.3: Is there evidence that results were robust to the presence of missing data?		
**Bias in measurement of outcomes**		Notes
6.1 Could the outcome measure have been influenced by knowledge of the intervention received?		
6.2 Were outcome assessors aware of the intervention received by study participants? (blinding of assessors)		
6.3 Were the methods of outcome assessment comparable across intervention groups?		
6.4 Were any systematic errors in measurement of the outcome related to intervention received?		
6.5 Could adherence have been influenced by study participation?		
**Bias in selection of the reported result**		Notes
Were the data that produced this result analysed in accordance with a pre-specified analysis plan that was finalized before unblinded outcome data were available for analysis?		
Is the reported effect estimate likely to be selected, on the basis of the results, from...		
7.1. ... multiple outcome measurements within the outcome domain?		
7.2 ... multiple analyses of the intervention-outcome relationship?		
7.3 ... different subgroups?		

Article Key

Criteria for Adherence Metrics and Analysis		Notes
**Device name and indication for use**	ReSET (Cognitive Behavioral Therapy for Substance use disorder)	
**Type of device** (Long-term vs short-term use)	Short-term use	
**Type of device** (Presence or absence of a prescribed dosage)	Presence of a prescribed dose	12 weeks of prescribed use
**Was adherence information collected?**	Yes	
**What information was collected about usage adherence?**	Number of completed modules and therapy sessions	
**What information was collected about adherence to recommendations?**	NA	No explicity app recommendations
**Was information collected internally in the app?**	Yes	
**Was information modified internally?**	NA	
**Was initiation reported?**	No	
**What was average adherence?** (if reported)		
**Was implementation reported?**	Yes	
**What was average adherence?** (if reported)	76.2% completed; mean of 36.6 computer-delivered modules (SD=18.1) out of a recommended 48 (range=0–72)	
**Was persistence reported?**	Yes	Time to treatment dropout was a primary outcome
**What was average adherence?** (if reported)	55% of participants had dropped out by week 12	
**Was adherence low?**	Yes	Average adherence was less than 80%
**Was adherence not reported or low?**	Yes	
**Was efficacy analyzed?**	No	The effect of the app on dropout time was analyzed but the effect of using the app on abstinence or dropout time was not
**What method was used?**		
**Were efficacy analyses preregistered?**		
**What assumptions are required for that method to study efficacy?**		
**Did the article report evidence that the assumptions were met?**		
**SUTVA**		
**Positivity**		
**Consistency** (treatment definition)		
**Consistency** (adherence definition)		
**Exclusion restriction**		
**Strong Monotonicity**		
**Ignorability**		
**Overall Notes**		
**Cochrane CDPLG**		**Notes**
**Data form completed date** (dd/mm/yyyy)	3/16/2022	
**Study author contact details**	anc2002@columbia.edu	
Methods	**Descriptions as stated in report/paper**	**Location in text or source** (pg & ¶/fig/table/other)
Aim of study (e.g. efficacy, equivalence, pragmatic)	Effectiveness	pg. 1 Introduction
Design (e.g. parallel, crossover, non-RCT)	Randomized controlled trial	pg. 2 Study Design
Unit of allocation (by individuals, cluster/ groups or body parts)	Individual	pg. 2 Study Design
Participants	**Descriptions as stated in report/paper**	**Location in text or source** (pg & ¶/fig/table/other)
**Inclusion criteria**	"Patients were eligible if they were age 18 or older; indicated by self-report that they had used illicit substances in the 30 days before study entry, or 60 days for those exiting a controlled environment (alcohol could be the primary problem, but patients had to have used at least one illicit drug as well); had entered the treatment episode within the past 30 days (randomization occurred on average 9.5 days [SD=7.4] after treatment entry); were planning to remain in the area and in the treatment program for at least 3 months; and were proficient in English."	pg. 2 Participants
**Exclusion criteria**	"Patients were excluded if they were being treated with opioid replacement therapy (e.g., buprenorphine, methadone) or were unable to provide informed consent."	pg. 2 Participants
**Total no. randomised** (or total pop. at start of study for NRCTs)	507	pg. 3 Results
**Clusters** (if applicable, no., type, no. people per cluster)	NA	
**Withdrawals and exclusions** (if not provided below by outcome)	45% (TAU) and 30% (TES) dropped out by week 8 and by week 12 60% (TAU) and 55% (TES) had dropped	pg. 6 Figure 2
Intervention Groups	**Descriptions as stated in report/paper**	**Location in text or source** (pg & ¶/fig/table/other)
**Group name**	Treatment as Usual	pg. 2 Study Design
**No. randomised to group** (specify whether no. people or clusters)	252	pg. 4 Table 1
**Timing** (e.g. frequency, duration of each episode)	"Programs had to offer at least two face-to-face therapeutic group or individual sessions per week, lasting at least 2 hours, with most offering two to six sessions per week."	pg. 2 Recruitement Sites
**Co-interventions**		
**Integrity of delivery**	Unclear	
**Compliance**	Participants attended 1.3 (SD=.9) therapy sessions per week prior to dropout (2-6 sessions recommended)	pg. 3 Treatment Adherence
Intervention Groups	**Descriptions as stated in report/paper**	**Location in text or source** (pg & ¶/fig/table/other)
**Group name**	Treatment as Usual + TES	pg. 2 Study Design
**No. randomised to group** (specify whether no. people or clusters)	255	pg. 4 Table 1
**Timing** (e.g. frequency, duration of each episode)	"TES includes contingency management and 62 interactive multimedia modules, based on the community reinforcement approach, requiring approximately 20–30 minutes each to complete."<br>Four modules per week were recommended.	pg. 2 Internet-Delivered Intervention
**Co-interventions**	TAU was also included<br>Clinicians were asked to incorporate a discussion of module completion	pg. 2 Internet-Delivered Intervention
**Integrity of delivery**	Unclear	
**Compliance**	"Patients in the TES group completed a mean of 36.6 computer-delivered modules (SD=18.1) out of a recommended 48 (range=0–72)"<br>Participants attended an average of 21.2 TAU therapy sessions (SD = 17.5)<br>Participants attended 1.4 (SD=.9) sessions per week prior to dropout (2-6 sessions recommended)	pg. 3 Treatment Adherence
Outcomes	**Descriptions as stated in report/paper**	**Location in text or source** (pg & ¶/fig/table/other)
**Outcome name**	Abstinence from drug or heavy alcohol use in the last 4 weeks of treatment	pg. 3 Sample Size, Power, and Statistical Analysis
**Outcome definition** (with diagnostic criteria if relevant)(include name, time, and analysis method)	"The dichotomous abstinence scores for each of the 24 half weeks in the 12-week treatment phase were analyzed using a repeated-measures piecewise logistic model, where a linear time-by-treatment interaction was allowed during the first 16 half weeks (8 weeks) but a constant study intervention effect was assumed during the last 8 half weeks (4 weeks)" (TAU vs TAU + TES)	pg. 3 Sample Size, Power, and Statistical Analysis
**Imputation of missing data** (e.g. assumptions made for ITT analysis)	Missing half week data were excluded (median of 1 for each arm for the last 8 weeks of treatment)	pg. 3 Sample Size, Power, and Statistical Analysis
**Power** (e.g. power & sample size calculation, level of power achieved)	80% power to detect an odds ratio of 1.5 for absence from drug or heavy alcohol use	pg. 3 Sample Size, Power, and Statistical Analysis
Outcomes	**Descriptions as stated in report/paper**	**Location in text or source** (pg & ¶/fig/table/other)
**Outcome name**	Retention in treatment (time to dropout) measured as the the last week that a particpant attended a face-to-face group or indiviual session	pg. 3 Sample Size, Power, and Statistical Analysis
**Outcome definition** (with diagnostic criteria if relevant)(include name, time, and analysis method)	"The primary retention outcome (time to dropout) was ana lyzed with survival methods stratified by site, using a log-rank test<br>and a proportional hazards model to consider effects of the strat ification factors as covariates (24)." (TAU vs TAU + TES)	pg. 3 Sample Size, Power, and Statistical Analysis
**Imputation of missing data** (e.g. assumptions made for ITT analysis)	NA	

**Power** (e.g. power & sample size calculation, level of power achieved)	90% power to detect 50% versus 35% (Internet-based intervention versus treatment as usual) retention	pg. 3 Sample Size, Power, and Statistical Analysis
**Risk of Bias** (Based on Cochrane RoB Tool 2 and ROBINS-I)		Link to RoB Algorithm
**Primary Analysis RoB Assessment**		Notes
**Is the primary analysis evaluating effectiveness or efficacy?**	Effectiveness	
**Comparator**	TES + TAU vs TAU	
**Outcome Being Assessed**	Abstinence from drug or heavy alcohol use in the last 4 weeks of treatment (analyzed with a repeated-measures piecewise logistic model)	
**Specify the Numerical Result Being Assessed**	OR = 1.62 (p=0.010)	
**Bias due to randomization (RCT only)**		Notes
1.1 Was the allocation sequence random?	Yes	Randomization handled by an independent statistician
1.2. Was the allocation sequence concealed until participants were enrolled and assigned to interventions?	Yes	
1.3. Did baseline differences between intervention groups suggest a problem with the randomization process?	No	There were no significant differences between groups for any baseline variable
**Bias due to confounding**		Notes
1.1 Is there potential for confounding of the effect of intervention in this study?	Probably No	There could have been spillover effects of treatment from providers
1.2. If Y/PY to 1.1 Was the analysis based on splitting participants' follow up time according to intervention received?		
1.3. If Y/PY to 1.2 Were intervention discontinuations or switches likely to be related to factors that are prognostic for the outcome?		
**Questions relating to baseline confounding only**		
1.4. If Y/PY to 1.1 Did the authors use an appropriate analysis method that controlled for all the important confounding domains?	Yes	Included stratification factors as main factors in the model and tested an additional model with significant factors
1.5. If Y/PY to 1.4: Were confounding domains that were controlled for measured validly and reliably by the variables available in this study?	Probably Yes	
1.6. Did the authors control for any post-intervention variables that could have been affected by the intervention?	No	
**Questions relating to baseline and time-varying confounding**		
1.7. If Y/PY to 1.3 Did the authors use an appropriate analysis method that controlled for all the important confounding domains and for time-varying confounding?		
1.8. If Y/PY to 1.7: Were confounding domains that were controlled for measured validly and reliably by the variables available in this study?		
**Bias in selection of participants into the study (Not evaluating for analyses of effectiveness)**		Notes
2.1. Was selection of participants into the analysis based on participant characteristics observed after the start of intervention?		
2.2. If Y/PY to 2.1: Were the post-intervention variables that influenced selection likely to be associated with intervention?		
2.3 If Y/PY to 2.2: Were the post-intervention variables that influenced selection likely to be influenced by the outcome or a cause of the outcome?		
2.4. Do start of follow-up and start of intervention coincide for most participants?		
2.5. If Y/PY to 2.2 and 2.3, or N/PN to 2.4: Were adjustment techniques used that are likely to correct for the presence of selection biases?		
**Bias in classification of interventions**		Notes
3.1 Were intervention groups clearly defined? Was the definition of adherence clearly defined?	Yes	
3.2 Was the information used to define intervention groups recorded at the start of the intervention?	Yes	
3.3 Could classification of intervention status have been affected by knowledge of the outcome or risk of the outcome?	No	
**Bias due to deviations from intended interventions**		Notes
**ROBINS-I**		
4.1. Were there deviations from the intended intervention beyond what would be expected in usual practice?	Probably No	There were incentives that increased retention, but these are also offered with the app on the market
4.2. If Y/PY to 4.1: Were these deviations from intended intervention unbalanced between groups and likely to have affected the outcome?		
4.3. Were important co-interventions balanced across intervention groups?	NA	
4.4. Was the intervention implemented successfully for most participants?	No information	No information about how many participants finished an initial module
4.5. Did study participants adhere to the assigned intervention regimen?	Probably No	On average 36.6/48 modules completed SD=18.1 (76.2% completed)
4.6. If N/PN to 4.3, 4.4 or 4.5: Was an appropriate analysis used to estimate the effect of starting and adhering to the intervention?		
Risk of bias: Assignment to intervention	**Low** / Moderate / Serious / Critical / NI	
Risk of bias: Starting and adhering to intervention	**Low** / Moderate / Serious / Critical / NI	
**Risk of bias judgement**	**Low** / Moderate / Serious / Critical / NI	
**RoB Tool (Risk of bias due to deviations from the intended interventions (effect of adhering to intervention))**		
2.1. Were participants aware of their assigned intervention during the trial?	Yes	
2.2. Were carers and people delivering the interventions aware of participants' assigned intervention during the trial?	Yes	Providers were not blinded
2.3. If Y/PY/NI to 2.1 or 2.2: Were important non protocol interventions balanced across intervention groups?	NA	
2.4. [If applicable:] Were there failures in implementing the intervention that could have affected the outcome?	No information	No information about how many participants finished an initial module
2.5. [If applicable:] Was there non-adherence to the assigned intervention regimen that could have affected participants' outcomes?	Probably Yes	On average 36.6/48 modules completed SD=18.1 (76.2% completed)
2.6. If N/PN/NI to 2.3, or Y/PY/NI to 2.4 or 2.5: Was an appropriate analysis used to estimate the effect of adhering to the intervention?		
**Risk of bias judgement**	Low/High/**Some Concerns**	
**Bias due to missing data**		Notes
5.1 Were outcome data available for all, or nearly all, participants?	Probably No	The majority of participants had dropped out by the end of the trial
5.2 Were participants excluded due to missing data on intervention status?	No	
5.3 Were participants excluded due to missing data on other variables needed for the analysis?	No	
5.4 If PN/N to 5.1, or Y/PY to 5.2 or 5.3: Are the proportion of participants and reasons for missing data similar across interventions?	Probably No	Proportions are not similar, but this was likely moderated through use of the intervention
5.5 If PN/N to 5.1, or Y/PY to 5.2 or 5.3: Is there evidence that results were robust to the presence of missing data?	No	Missing data were excluded and no sensitivity analyses were conducted
**Bias in measurement of outcomes**		Notes
6.1 Could the outcome measure have been influenced by knowledge of the intervention received?	Probably No	The drug test was an empirical measure but the self-reports could have been affected by participants believing they were in the treatment group
6.2 Were outcome assessors aware of the intervention received by study participants? (blinding of assessors)	Yes	Self-reports were used and paticipants were aware of the intervention
6.3 Were the methods of outcome assessment comparable across intervention groups?	Yes	
6.4 Were any systematic errors in measurement of the outcome related to intervention received?	No	
**Bias in selection of the reported result**		Notes

Were the data that produced this result analysed in accordance with a pre-specified analysis plan that was finalized before unblinded outcome data were available for analysis?	Yes	https://www.ncbi.nlm.nih.gov/pmc/articles/PMC3268951/ NCT01104805
Is the reported effect estimate likely to be selected, on the basis of the results, from...		
7.1. ... multiple outcome measurements within the outcome domain?	No	
7.2 ... multiple analyses of the intervention-outcome relationship?	No	
7.3 ... different subgroups?	No	
**Primary Analysis RoB Assessment**		Notes
**Is the primary analysis evaluating effectiveness or efficacy?**	Effectiveness	
**Comparator**	TES + TAU vs TAU	
**Outcome Being Assessed**	Time to dropout during the 12 week treatment period	
**Specify the Numerical Result Being Assessed**	hazard ratio=0.72, 95% CI=0.57, 0.92, p=0.010)	
**Bias due to randomization (RCT only)**		Notes
1.1 Was the allocation sequence random?	Yes	Randomization handled by an independent statistician
1.2. Was the allocation sequence concealed until participants were enrolled and assigned to interventions?	Yes	
1.3. Did baseline differences between intervention groups suggest a problem with the randomization process?	No	There were no significant differences between groups for any baseline variable
**Bias due to confounding**		Notes
1.1 Is there potential for confounding of the effect of intervention in this study?	Probably No	There could have been spillover effects of treatment from providers
1.2. If Y/PY to 1.1 Was the analysis based on splitting participants' follow up time according to intervention received?		
1.3. If Y/PY to 1.2 Were intervention discontinuations or switches likely to be related to factors that are prognostic for the outcome?		
**Questions relating to baseline confounding only**		
1.4. If Y/PY to 1.1 Did the authors use an appropriate analysis method that controlled for all the important confounding domains?	Yes	Included stratification factors as main factors in the model and tested an additional model with significant factors
1.5. If Y/PY to 1.4: Were confounding domains that were controlled for measured validly and reliably by the variables available in this study?	Probably Yes	some factors were self-reports but differences were likely even across groups
1.6. Did the authors control for any post-intervention variables that could have been affected by the intervention?	No	
**Questions relating to baseline and time-varying confounding**		
1.7. If Y/PY to 1.3 Did the authors use an appropriate analysis method that controlled for all the important confounding domains and for time-varying confounding?		
1.8. If Y/PY to 1.7: Were confounding domains that were controlled for measured validly and reliably by the variables available in this study?		
**Bias in selection of participants into the study (Not evaluating for analyses of effectiveness)**		Notes
2.1. Was selection of participants into the analysis based on participant characteristics observed after the start of intervention?		
2.2. If Y/PY to 2.1: Were the post-intervention variables that influenced selection likely to be associated with intervention?		
2.3 If Y/PY to 2.2: Were the post-intervention variables that influenced selection likely to be influenced by the outcome or a cause of the outcome?		
2.4. Do start of follow-up and start of intervention coincide for most participants?		
2.5. If Y/PY to 2.2 and 2.3, or N/PN to 2.4: Were adjustment techniques used that are likely to correct for the presence of selection biases?		
**Bias in classification of interventions**		Notes
3.1 Were intervention groups clearly defined? Was the definition of adherence clearly defined?	Yes	
3.2 Was the information used to define intervention groups recorded at the start of the intervention?	Yes	
3.3 Could classification of intervention status have been affected by knowledge of the outcome or risk of the outcome?	No	
**Bias due to deviations from intended interventions**		Notes
**ROBINS-I**		
4.1. Were there deviations from the intended intervention beyond what would be expected in usual practice?	Probably No	There were incentives that increased retention, but these are also offered with the app on the market
4.2. If Y/PY to 4.1: Were these deviations from intended intervention unbalanced between groups and likely to have affected the outcome?		
4.3. Were important co-interventions balanced across intervention groups?	NA	
4.4. Was the intervention implemented successfully for most participants?	No information	No information about how many participants finished an initial module
4.5. Did study participants adhere to the assigned intervention regimen?	Probably Yes	On average 36.6/48 modules completed SD=18.1 (76.2% completed)
4.6. If N/PN to 4.3, 4.4 or 4.5: Was an appropriate analysis used to estimate the effect of starting and adhering to the intervention?		
Risk of bias: Assignment to intervention	**Low** / Moderate / Serious / Critical / NI	
Risk of bias: Starting and adhering to intervention	**Low** / Moderate / Serious / Critical / NI	
**Risk of bias judgement**	**Low** / Moderate / Serious / Critical / NI	
**RoB Tool (Risk of bias due to deviations from the intended interventions (effect of adhering to intervention))**		
2.1. Were participants aware of their assigned intervention during the trial?	Yes	
2.2. Were carers and people delivering the interventions aware of participants' assigned intervention during the trial?	Yes	Providers were not blinded
2.3. If Y/PY/NI to 2.1 or 2.2: Were important non protocol interventions balanced across intervention groups?	NA	
2.4. [If applicable:] Were there failures in implementing the intervention that could have affected the outcome?	No information	No information about how many participants finished an initial module
2.5. [If applicable:] Was there non-adherence to the assigned intervention regimen that could have affected participants' outcomes?	Probably No	On average 36.6/48 modules completed SD=18.1 (76.2% completed)
2.6. If N/PN/NI to 2.3, or Y/PY/NI to 2.4 or 2.5: Was an appropriate analysis used to estimate the effect of adhering to the intervention?		
**Risk of bias judgement**	Low/High/**Some Concerns**	
**Bias due to missing data**		Notes
5.1 Were outcome data available for all, or nearly all, participants?	NA	Outcome is missing data
5.2 Were participants excluded due to missing data on intervention status?	No	
5.3 Were participants excluded due to missing data on other variables needed for the analysis?	No	
5.4 If PN/N to 5.1, or Y/PY to 5.2 or 5.3: Are the proportion of participants and reasons for missing data similar across interventions?	NA	Outcome is missing data
5.5 If PN/N to 5.1, or Y/PY to 5.2 or 5.3: Is there evidence that results were robust to the presence of missing data?	NA	Outcome is missing data
**Bias in measurement of outcomes**		Notes
6.1 Could the outcome measure have been influenced by knowledge of the intervention received?	No	
6.2 Were outcome assessors aware of the intervention received by study participants? (blinding of assessors)	Yes	
6.3 Were the methods of outcome assessment comparable across intervention groups?	Yes	
6.4 Were any systematic errors in measurement of the outcome related to intervention received?	No	
**Bias in selection of the reported result**		Notes

Were the data that produced this result analysed in accordance with a pre-specified analysis plan that was finalized before unblinded outcome data were available for analysis?	Yes	https://www.ncbi.nlm.nih.gov/pmc/articles/PMC3268951/ NCT01104805
Is the reported effect estimate likely to be selected, on the basis of the results, from...		
7.1. ... multiple outcome measurements within the outcome domain?	No	
7.2 ... multiple analyses of the intervention-outcome relationship?	No	
7.3 ... different subgroups?	No	
**Secondary Analysis RoB Assessment**		Notes
**Is the seconday analysis evaluating effectiveness or efficacy?**		
**Comparator**		
**Outcome Being Assessed**		
**Specify the Numerical Result Being Assessed**		
**Bias due to randomization (See primary analysis section)**		Notes
**Bias due to confounding**		Notes
1.1 Is there potential for confounding of the effect of intervention in this study?		
1.2. If Y/PY to 1.1 Was the analysis based on splitting participants' follow up time according to intervention received?		
1.3. If Y/PY to 1.2 Were intervention discontinuations or switches likely to be related to factors that are prognostic for the outcome?		
**Questions relating to baseline confounding only**		
1.4. If Y/PY to 1.1 Did the authors use an appropriate analysis method that controlled for all the important confounding domains?		
1.5. If Y/PY to 1.4: Were confounding domains that were controlled for measured validly and reliably by the variables available in this study?		
1.6. Did the authors control for any post-intervention variables that could have been affected by the intervention?		
**Questions relating to baseline and time-varying confounding**		
1.7. If Y/PY to 1.3 Did the authors use an appropriate analysis method that controlled for all the important confounding domains and for time-varying confounding?		
1.8. If Y/PY to 1.7: Were confounding domains that were controlled for measured validly and reliably by the variables available in this study?		
**Bias in selection of participants into the study (Not evaluating for analyses of effectiveness)**		Notes
2.1. Was selection of participants into the analysis based on participant characteristics observed after the start of intervention?		
2.2. If Y/PY to 2.1: Were the post-intervention variables that influenced selection likely to be associated with intervention?		
2.3 If Y/PY to 2.2: Were the post-intervention variables that influenced selection likely to be influenced by the outcome or a cause of the outcome?		
2.4. Do start of follow-up and start of intervention coincide for most participants?		
2.5. If Y/PY to 2.2 and 2.3, or N/PN to 2.4: Were adjustment techniques used that are likely to correct for the presence of selection biases?		
**Bias in classification of interventions**		Notes
3.1 Were intervention groups clearly defined?		
3.2 Was the information used to define intervention groups recorded at the start of the intervention?		
3.3 Could classification of intervention status have been affected by knowledge of the outcome or risk of the outcome?		
**Bias due to deviations from intended interventions (See primary analysis section)**		Notes
**Bias due to missing data**		Notes
5.1 Were outcome data available for all, or nearly all, participants?		
5.2 Were participants excluded due to missing data on intervention status?		
5.3 Were participants excluded due to missing data on other variables needed for the analysis?		
5.4 If PN/N to 5.1, or Y/PY to 5.2 or 5.3: Are the proportion of participants and reasons for missing data similar across interventions?		
5.5 If PN/N to 5.1, or Y/PY to 5.2 or 5.3: Is there evidence that results were robust to the presence of missing data?		
**Bias in measurement of outcomes**		Notes
6.1 Could the outcome measure have been influenced by knowledge of the intervention received?		
6.2 Were outcome assessors aware of the intervention received by study participants? (blinding of assessors)		
6.3 Were the methods of outcome assessment comparable across intervention groups?		
6.4 Were any systematic errors in measurement of the outcome related to intervention received?		
6.5 Could adherence have been influenced by study participation?		
**Bias in selection of the reported result**		Notes
Were the data that produced this result analysed in accordance with a pre-specified analysis plan that was finalized before unblinded outcome data were available for analysis?		
Is the reported effect estimate likely to be selected, on the basis of the results, from...		
7.1. ... multiple outcome measurements within the outcome domain?		
7.2 ... multiple analyses of the intervention-outcome relationship?		
7.3 ... different subgroups?		

Criteria for Adherence Metrics and Analysis		Notes
**Device name and indication for use**	ReSET-O (Cognitive Behavioral Therapy for Opioid use disorder)	
**Type of device** (Long-term vs short-term use)	Short-term use	
**Type of device** (Presence or absence of a prescribed dosage)	Prescribed dosage	
**Was adherence information collected?**	Yes	
**What information was collected about usage adherence?**	Retention in treatment	
**What information was collected about adherence to recommendations?**	NA	No explicit app recommendations
**Was information collected internally in the app?**	Yes	
**Was information modified internally?**	NA	
**Was initiation reported?**	No	
**What was average adherence?** (if reported)		
**Was implementation reported?**	No	
**What was average adherence?** (if reported)		
**Was persistence reported?**	Yes	
**What was average adherence?** (if reported)	80% retained at the end of 12 weeks	Time to dropout was a reported outcome but time till discontinuation of app was not reported
**Was adherence low?**	NA	
**Was adherence not reported or low?**	Yes	Implementation was not reported
**Was efficacy analyzed?**	No	
**What method was used?**	None	
**Were efficacy analyses preregistered?**	NA	
**What assumptions are required for that method to study efficacy?**		
**Did the article report evidence that the assumptions were met?**		
**SUTVA**		
**Positivity**		
**Consistency** (treatment definition)		
**Consistency** (adherence definition)		
**Exclusion restriction**		
**Strong Monotonicity**		
**Ignorability**		
**Overall Notes**		
**Cochrane CDPLG**		**Notes**
**Data form completed date** (dd/mm/yyyy)	3/18/2022	
**Study author contact details**	wkbickel@vtc.vt.edu	
Methods	**Descriptions as stated in report/paper**	**Location in text or source** (pg & ¶/fig/table/other)
Aim of study (e.g. efficacy, equivalence, pragmatic)	Effectiveness (by the definition set in our introduction)	pg. 4 Community Reinforcement Approach
Design (e.g. parallel, crossover, non-RCT)	Parallel RCT	pg. 3 Procedure
Unit of allocation (by individuals, cluster/ groups or body parts)	Individual	pg. 3 Procedure
Participants	**Descriptions as stated in report/paper**	**Location in text or source** (pg & ¶/fig/table/other)
**Inclusion criteria**	"Those who were eligible met the Diagnostic and Statistical Manual of Mental Disorders (4th ed.) criteria for opioid dependence and met the Food and Drug Administration qualification criteria for buprenorphine treatment. Additionally, participants could not be pregnant or incarcerated nor have shown evidence of an active (nonsubstance dependence) psychiatric disorder or significant medical illness"	pg. 3 Participants
**Exclusion criteria**	Participants with "very high" drug concentration were excluded based on study physician's recommendations. Six participants were excluded for other reasons including unreliable transport to clinic.	pg. 3 Participants; pg. 3 Figure 1
**Total no. randomised** (or total pop. at start of study for NRCTs)	170	pg. 3 Figure 1
**Clusters** (if applicable, no., type, no. people per cluster)	NA	
**Withdrawals and exclusions** (if not provided below by outcome)	No exclusions, Missing data was considered not abstinent	pg. 4 Abstinence
Intervention Groups	**Descriptions as stated in report/paper**	**Location in text or source** (pg & ¶/fig/table/other)
**Group name**	Internet-based community reinforcement approach intervention plus contingency management (CRA+)	pg. 1 Abstract
**No. randomised to group** (specify whether no. people or clusters)	92	pg. 3 Figure 1
**Timing** (e.g. frequency, duration of each episode)	Visits 3 days per week (max of 36 visits)	pg. 4 Measures
**Co-interventions**	Buprenorphine treatment; Counseling every 2 weeks	pg. 1 Abstract
**Integrity of delivery**	No information	
**Compliance**	No information	
Intervention Groups	**Descriptions as stated in report/paper**	**Location in text or source** (pg & ¶/fig/table/other)
**Group name**	Contingency management alone (CM)	pg. 1 Abstract
**No. randomised to group** (specify whether no. people or clusters)	78	pg. 3 Figure 1
**Timing** (e.g. frequency, duration of each episode)	Visits and modules 3 days per week (30 minutes per visit)	pg. 4 Measures
**Co-interventions**	Buprenorphine treatment; Counseling every 2 weeks	pg. 1 Abstract
**Integrity of delivery**	No information	
**Compliance**	NA	
Outcomes	**Descriptions as stated in report/paper**	**Location in text or source** (pg & ¶/fig/table/other)
**Outcome name**	Longest Continuous Abstinence (LCA)	pg. 4 Measures
**Outcome definition** (with diagnostic criteria if relevant)(include name, time, and analysis method)	LCA over 12 weeks analyzed with ITT using a t-test	pg. 4 Retention and Drug Abstinence
**Imputation of missing data** (e.g. assumptions made for ITT analysis)	A missing visit was considered a positive drug test	pg. 4 Measures
**Power** (e.g. power & sample size calculation, level of power achieved)	80% power to detect a 3 week difference in mean weeks of continuous abstinence	pg. 3 Participants
Outcomes	**Descriptions as stated in report/paper**	**Location in text or source** (pg & ¶/fig/table/other)
**Outcome name**	Total Abstinence (TA)	pg. 4 Measures
**Outcome definition** (with diagnostic criteria if relevant)(include name, time, and analysis method)	TA over 12 weeks analyzed with ITT using a t-test	pg. 4 Retention and Drug Abstinence
**Imputation of missing data** (e.g. assumptions made for ITT analysis)	A missing visit was considered a positive drug test	pg. 4 Measures
**Power** (e.g. power & sample size calculation, level of power achieved)	80% power to detect a 3 week difference in mean weeks of continuous abstinence	pg. 3 Participants
**Risk of Bias** (Based on Cochrane RoB Tool 2 and ROBINS-I)		Link to RoB Algorithm
**Primary Analysis RoB Assessment**		Notes
**Is the primary analysis evaluating effectiveness or efficacy?**	Effectiveness	
**Comparator**	CRA+ vs CM	
**Outcome Being Assessed**	TA and LCA over 12 weeks analyzed with ITT using a t-test (ITT)	
**Specify the Numerical Result Being Assessed**	"On average, the LCA for CRA+ participants was 55.0 days compared to CM-alone participants' mean of 49.5 days, t(152.4)=1.25, p= .214.<br>The mean TA was 67.1 days for the CRA+ group and 57.3 days for the CM-alone group, t(133.4)= 2.59, p= .011."	
**Bias due to randomization (RCT only)**		Notes
1.1 Was the allocation sequence random?	Yes	
1.2. Was the allocation sequence concealed until participants were enrolled and assigned to interventions?	No	Unblinded allocation due to stratification based on dose of buprenorphine
1.3. Did baseline differences between intervention groups suggest a problem with the randomization process?	No	Two covariates had statistical differences which would be about expected for the number of covariates analyzed
**Bias due to confounding**		Notes
1.1 Is there potential for confounding of the effect of intervention in this study?	No	RCT, ITT effect, statistical plan to control for potential confounders
1.2. If Y/PY to 1.1 Was the analysis based on splitting participants' follow up time according to intervention received?		
1.3. If Y/PY to 1.2 Were intervention discontinuations or switches likely to be related to factors that are prognostic for the outcome?		

Article Key

**Questions relating to baseline confounding only**		
1.4. If Y/PY to 1.1 Did the authors use an appropriate analysis method that controlled for all the important confounding domains?		
1.5. If Y/PY to 1.4: Were confounding domains that were controlled for measured validly and reliably by the variables available in this study?		
1.6. Did the authors control for any post-intervention variables that could have been affected by the intervention?		
**Questions relating to baseline and time-varying confounding**		
1.7. If Y/PY to 1.3 Did the authors use an appropriate analysis method that controlled for all the important confounding domains and for time-varying confounding?		
1.8. If Y/PY to 1.7: Were confounding domains that were controlled for measured validly and reliably by the variables available in this study?		
**Bias in selection of participants into the study (Not evaluating for analyses of effectiveness)**		Notes
2.1. Was selection of participants into the analysis based on participant characteristics observed after the start of intervention?		
2.2. If Y/PY to 2.1: Were the post-intervention variables that influenced selection likely to be associated with intervention?		
2.3 If Y/PY to 2.2: Were the post-intervention variables that influenced selection likely to be influenced by the outcome or a cause of the outcome?		
2.4. Do start of follow-up and start of intervention coincide for most participants?		
2.5. If Y/PY to 2.2 and 2.3, or N/PN to 2.4: Were adjustment techniques used that are likely to correct for the presence of selection biases?		
**Bias in classification of interventions**		Notes
3.1 Were intervention groups clearly defined? Was the definition of adherence clearly defined?	Yes	
3.2 Was the information used to define intervention groups recorded at the start of the intervention?	Yes	Treatment arm definitions were preregistered on clinicaltrials.gov
3.3 Could classification of intervention status have been affected by knowledge of the outcome or risk of the outcome?	No	
**Bias due to deviations from intended interventions**		Notes
**ROBINS-I**		
4.1. Were there deviations from the intended intervention beyond what would be expected in usual practice?	No	
4.2. If Y/PY to 4.1: Were these deviations from intended intervention unbalanced between groups and likely to have affected the outcome?		
4.3. Were important co-interventions balanced across intervention groups?	Yes	They stratified groups based on Buprenorphine use
4.4. Was the intervention implemented successfully for most participants?	No information	
4.5. Did study participants adhere to the assigned intervention regimen?	No information	
4.6. If N/PN to 4.3, 4.4 or 4.5: Was an appropriate analysis used to estimate the effect of starting and adhering to the intervention?	No information	No information about adherence
Risk of bias: Assignment to intervention	**Low** / Moderate / Serious / Critical / NI	
Risk of bias: Starting and adhering to intervention	Low / Moderate / Serious / **Critical** / NI	
**Risk of bias judgement**	Low / Moderate / Serious / **Critical** / NI	
**RoB Tool (Risk of bias due to deviations from the intended interventions (effect of adhering to intervention))**		
2.1. Were participants aware of their assigned intervention during the trial?	Yes	
2.2. Were carers and people delivering the interventions aware of participants' assigned intervention during the trial?	Yes	
2.3. If Y/PY/NI to 2.1 or 2.2: Were important non protocol interventions balanced across intervention groups?	NA	
2.4. [If applicable:] Were there failures in implementing the intervention that could have affected the outcome?	No information	
2.5. [If applicable:] Was there non-adherence to the assigned intervention regimen that could have affected participants' outcomes?	No information	
2.6. If N/PN/NI to 2.3, or Y/PY/NI to 2.4 or 2.5: Was an appropriate analysis used to estimate the effect of adhering to the intervention?	No information	No information about adherence
**Risk of bias judgement**	Low/**High**/Some Concerns	
**Bias due to missing data**		Notes
5.1 Were outcome data available for all, or nearly all, participants?	Yes	Missing data categorized as not abstinent
5.2 Were participants excluded due to missing data on intervention status?	No	
5.3 Were participants excluded due to missing data on other variables needed for the analysis?	No	
5.4 If PN/N to 5.1, or Y/PY to 5.2 or 5.3: Are the proportion of participants and reasons for missing data similar across interventions?		
5.5 If PN/N to 5.1, or Y/PY to 5.2 or 5.3: Is there evidence that results were robust to the presence of missing data?		
**Bias in measurement of outcomes**		Notes
6.1 Could the outcome measure have been influenced by knowledge of the intervention received?	No	Drug test is an objective measure
6.2 Were outcome assessors aware of the intervention received by study participants? (blinding of assessors)	Yes	
6.3 Were the methods of outcome assessment comparable across intervention groups?	Yes	
6.4 Were any systematic errors in measurement of the outcome related to intervention received?	No	
**Bias in selection of the reported result**		Notes
Were the data that produced this result analysed in accordance with a pre-specified analysis plan that was finalized before unblinded outcome data were available for analysis?	Yes	
Is the reported effect estimate likely to be selected, on the basis of the results, from...		
7.1. ... multiple outcome measurements within the outcome domain?	Yes	Not all of the primary outcomes were registered on clinicaltrials.gov
7.2 ... multiple analyses of the intervention-outcome relationship?	Yes	They did not provide the published protocol or their SAP
7.3 ... different subgroups?	No	
**Secondary Analysis RoB Assessment**		Notes
**Is the seconday analysis evaluating effectiveness or efficacy?**		
**Comparator**		
**Outcome Being Assessed**		
**Specify the Numerical Result Being Assessed**		
**Bias due to randomization (See primary analysis section)**		Notes
**Bias due to confounding**		Notes
1.1 Is there potential for confounding of the effect of intervention in this study?		
1.2. If Y/PY to 1.1 Was the analysis based on splitting participants' follow up time according to intervention received?		
1.3. If Y/PY to 1.2 Were intervention discontinuations or switches likely to be related to factors that are prognostic for the outcome?		
**Questions relating to baseline confounding only**		
1.4. If Y/PY to 1.1 Did the authors use an appropriate analysis method that controlled for all the important confounding domains?		
1.5. If Y/PY to 1.4: Were confounding domains that were controlled for measured validly and reliably by the variables available in this study?		
1.6. Did the authors control for any post-intervention variables that could have been affected by the intervention?		
**Questions relating to baseline and time-varying confounding**		

1.7. If Y/PY to 1.3 Did the authors use an appropriate analysis method that controlled for all the important confounding domains and for time-varying confounding?		
1.8. If Y/PY to 1.7: Were confounding domains that were controlled for measured validly and reliably by the variables available in this study?		
**Bias in selection of participants into the study (Not evaluating for analyses of effectiveness)**		Notes
2.1. Was selection of participants into the analysis based on participant characteristics observed after the start of intervention?		
2.2. If Y/PY to 2.1: Were the post-intervention variables that influenced selection likely to be associated with intervention?		
2.3 If Y/PY to 2.2: Were the post-intervention variables that influenced selection likely to be influenced by the outcome or a cause of the outcome?		
2.4. Do start of follow-up and start of intervention coincide for most participants?		
2.5. If Y/PY to 2.2 and 2.3, or N/PN to 2.4: Were adjustment techniques used that are likely to correct for the presence of selection biases?		
**Bias in classification of interventions**		Notes
3.1 Were intervention groups clearly defined?		
3.2 Was the information used to define intervention groups recorded at the start of the intervention?		
3.3 Could classification of intervention status have been affected by knowledge of the outcome or risk of the outcome?		
**Bias due to deviations from intended interventions (See primary analysis section)**		Notes
**Bias due to missing data**		Notes
5.1 Were outcome data available for all, or nearly all, participants?		
5.2 Were participants excluded due to missing data on intervention status?		
5.3 Were participants excluded due to missing data on other variables needed for the analysis?		
5.4 If PN/N to 5.1, or Y/PY to 5.2 or 5.3: Are the proportion of participants and reasons for missing data similar across interventions?		
5.5 If PN/N to 5.1, or Y/PY to 5.2 or 5.3: Is there evidence that results were robust to the presence of missing data?		
**Bias in measurement of outcomes**		Notes
6.1 Could the outcome measure have been influenced by knowledge of the intervention received?		
6.2 Were outcome assessors aware of the intervention received by study participants? (blinding of assessors)		
6.3 Were the methods of outcome assessment comparable across intervention groups?		
6.4 Were any systematic errors in measurement of the outcome related to intervention received?		
6.5 Could adherence have been influenced by study participation?		
**Bias in selection of the reported result**		Notes
Were the data that produced this result analysed in accordance with a pre-specified analysis plan that was finalized before unblinded outcome data were available for analysis?		
Is the reported effect estimate likely to be selected, on the basis of the results, from...		
7.1. ... multiple outcome measurements within the outcome domain?		
7.2 ... multiple analyses of the intervention-outcome relationship?		
7.3 ... different subgroups?		

Article Key

Criteria for Adherence Metrics and Analysis		Notes
**Device name and indication for use**	Bluestar (annotation feature) management of diabetes	
**Type of device** (Long-term vs short-term use)	Long-term use	
**Type of device** (Presence or absence of a prescribed dosage)	Absence of a prescribed dosage	
**Was adherence information collected?**	Yes	
**What information was collected about usage adherence?**	Use of feature in first 14 days of platform use<br>Number of annotations during trial<br>Number of days user engaged with the platform	
**What information was collected about adherence to recommendations?**	NA	No explicit app recommendations
**Was information collected internally in the app?**	Yes	
**Was information modified internally?**	NA	
**Was initiation reported?**	Yes	Number of participants recording at least one annotation
**What was average adherence?** (if reported)	1045 (33.3%)<br>941 (29.9%)	Structered annotation<br>Unstructured annotation
**Was implementation reported?**	Yes	Classified participants with 2 A1c values reported based on their annotation usage in 90 days
**What was average adherence?** (if reported)	No notes: n=93<br>Moderate notes (1-9 notes): n=120<br>High notes (10+ notes): n=159	
**Was persistence reported?**	Yes	
**What was average adherence?** (if reported)	"Adjusting for covariates including demographic characteristics, other engagement data, and baseline A1C were included in the model as covariates, note-takers persisted for an estimated 174.98 days (95% CI: 152.07-201.33). In contrast, users recording no notes in the first 14 days of engagement used the digital health solution over the course of an estimated 131.30days (95% CI: 119.15-144.69)."	
**Was adherence low?**	Yes	Given the study period, users on average used the annotation feature for 4-35% of the days
**Was adherence not reported or low?**	Yes	Low adherence with an observational design
**Was efficacy analyzed?**	Yes	
**What method was used?**	As-treated analysis	
**Were efficacy analyses preregistered?**	No	
**What assumptions are required for that method to study efficacy?**	SUTVA, positivity, consistency, ignorability, conditional independence of adherence and outcomes	
**Did the article report evidence that the assumptions were met?**	No	
**SUTVA**	Yes	Treatment use only through the app
**Positivity**	Yes	Likely a non-zero chance to use the app
**Consistency** (treatment definition)	Yes	clear defintion of treatment
**Consistency** (adherence definition)	Yes	Clear definition of usage in each group
**Exclusion restriction**		
**Strong Monotonicity**		
**Ignorability**	time, demographic characteristics	
**Overall Notes**		
**Cochrane CDPLG**		**Notes**
**Data form completed date** (dd/mm/yyyy)	3/28/2022	
**Study author contact details**	mdugas@umd.edu	
Methods	**Descriptions as stated in report/paper**	**Location in text or source** (pg & ¶/fig/table/other)
Aim of study (e.g. efficacy, equivalence, pragmatic)	Efficacy	pg. 2 Objectives
Design (e.g. parallel, crossover, non-RCT)	Observational	pg.2 Methods
Unit of allocation (by individuals, cluster/ groups or body parts)	Individual	
Participants	**Descriptions as stated in report/paper**	**Location in text or source** (pg & ¶/fig/table/other)
**Inclusion criteria**	"Users with at least two [self-reported] A1C measures (n = 372)"<br>Users of the BlueStar digital health solution collected between December 2013 and June 2017	pg. 4 Relationship between annotations and clinical outcomes<br>pg. 2 Sample and digital health solution
**Exclusion criteria**		
**Total no. randomised** (or total pop. at start of study for NRCTs)	372 included in analysis; 3142 included in sample	pg. 4 Relationship between annotations and clinical outcomes<br>pg. 2 Sample and digital health solution
**Clusters** (if applicable, no., type, no. people per cluster)	NA	
**Withdrawals and exclusions** (if not provided below by outcome)	28 excluded from sample due to not documenting demographic information	pg. 4 Relationship Between Early Annotation Use and Persistence in Engagement
Intervention Groups	**Descriptions as stated in report/paper**	**Location in text or source** (pg & ¶/fig/table/other)
**Group name**	Bluestar annotation feature users	pg. 1 Abstract
**No. randomised to group** (specify whether no. people or clusters)	372 included in analysis; 3142 included in sample	pg. 3 Table 1
**Timing** (e.g. frequency, duration of each episode)	No prescribed dose	
**Co-interventions**	Usual care	
**Integrity of delivery**	All participants needed to use the app to be included in the sample	pg. 2 Sample and Digital Health Solution
**Compliance**	The majority of users out of the sample did not use the annotation feature (33.3% used structured annotations, 29.9% used unstructured annotations)<br>There was a high degree of overlap between users of the two annotation features	pg. 3 Annotation themes<br>pg. 3 Relationship Between Early Annotation Use and Persistence in Engagement
Outcomes	**Descriptions as stated in report/paper**	**Location in text or source** (pg & ¶/fig/table/other)
**Outcome name**	Change in A1c	pg. 3 Objective 2
**Outcome definition** (with diagnostic criteria if relevant)(include name, time, and analysis method)	Mixed effects model analyzing 90 day usage on A1c	pg. 4 Relationship Between Annotations and Clinical Outcomes
**Imputation of missing data** (e.g. assumptions made for ITT analysis)		
**Power** (e.g. power & sample size calculation, level of power achieved)		
**Risk of Bias** (Based on Cochrane RoB Tool 2 and ROBINS-I)		Link to RoB Algorithm
**Primary Analysis RoB Assessment**		Notes
**Is the primary analysis evaluating effectiveness or efficacy?**	Efficacy	
**Comparator**	No use vs Moderate use<br>No use vs High use	
**Outcome Being Assessed**	Change in A1c	
**Specify the Numerical Result Being Assessed**	"There was no significant interaction effect of Time × Moderate note usage (B = −0.40, P = .17), though both groups saw significant declines in A1C over time (P < .001). The regression analysis did reveal a significant interaction effect of Time x High note usage (B = −0.64, P = .02) indicating that the decrease in A1C was significantly greater for those with a high level of note usage"	
**Bias due to randomization (RCT only)**		Notes
1.1 Was the allocation sequence random?	NA	
1.2. Was the allocation sequence concealed until participants were enrolled and assigned to interventions?	NA	
1.3. Did baseline differences between intervention groups suggest a problem with the randomization process?	NA	
**Bias due to confounding**		Notes
1.1 Is there potential for confounding of the effect of intervention in this study?	Yes	
1.2. If Y/PY to 1.1 Was the analysis based on splitting participants' follow up time according to intervention received?	No	
1.3. If Y/PY to 1.2 Were intervention discontinuations or switches likely to be related to factors that are prognostic for the outcome?		
**Questions relating to baseline confounding only**		
1.4. If Y/PY to 1.1 Did the authors use an appropriate analysis method that controlled for all the important confounding domains?	No information	They controlled for many variables but did not assess how well the covariates accounted for confounders of usage

1.5. If Y/PY to 1.4: Were confounding domains that were controlled for measured validly and reliably by the variables available in this study?	Probably yes	
1.6. Did the authors control for any post-intervention variables that could have been affected by the intervention?	No	
**Questions relating to baseline and time-varying confounding**		
1.7. If Y/PY to 1.3 Did the authors use an appropriate analysis method that controlled for all the important confounding domains and for time-varying confounding?		
1.8. If Y/PY to 1.7: Were confounding domains that were controlled for measured validly and reliably by the variables available in this study?		
**Bias in selection of participants into the study (Not evaluating for analyses of effectiveness)**		Notes
2.1. Was selection of participants into the analysis based on participant characteristics observed after the start of intervention?	Yes	Selection was based on participants having two A1c values and they were grouped based on note usage
2.2. If Y/PY to 2.1: Were the post-intervention variables that influenced selection likely to be associated with intervention?	Probably Yes	Participants who used the app more often were more likely to have 2 a1c data points further apart which means that a1c could have been further reduced due to time and outside factors
2.3 If Y/PY to 2.2: Were the post-intervention variables that influenced selection likely to be influenced by the outcome or a cause of the outcome?	No	
2.4. Do start of follow-up and start of intervention coincide for most participants?	No information	
2.5. If Y/PY to 2.2 and 2.3, or N/PN to 2.4: Were adjustment techniques used that are likely to correct for the presence of selection biases?	Probably No	Control variables were used but it is unclear if they corrected for the presence of selection biases
**Bias in classification of interventions**		Notes
3.1 Were intervention groups clearly defined? Was the definition of adherence clearly defined?	Yes	
3.2 Was the information used to define intervention groups recorded at the start of the intervention?	No	This analysis was not preregistered so the analysis process could have affected how the groups were defined.
3.3 Could classification of intervention status have been affected by knowledge of the outcome or risk of the outcome?	Yes	This analysis was not preregistered so the analysis process could have affected how the groups were defined.
**Bias due to deviations from intended interventions**		Notes
**ROBINS-I**		
4.1. Were there deviations from the intended intervention beyond what would be expected in usual practice?	No	
4.2. If Y/PY to 4.1: Were these deviations from intended intervention unbalanced between groups and likely to have affected the outcome?		
4.3. Were important co-interventions balanced across intervention groups?	No information	
4.4. Was the intervention implemented successfully for most participants?	Yes	
4.5. Did study participants adhere to the assigned intervention regimen?	No	25% of people included in the analysis did not use the feature
4.6. If N/PN to 4.3, 4.4 or 4.5: Was an appropriate analysis used to estimate the effect of starting and adhering to the intervention?	No	Dose-response could have been informative but they did not prove that the criteria needed to use
Risk of bias: Assignment to intervention	**Low** / Moderate / Serious / Critical / NI	
Risk of bias: Starting and adhering to intervention	Low / Moderate / Serious / **Critical** / NI	
**Risk of bias judgement**	Low / Moderate / Serious / **Critical** / NI	
**RoB Tool (Risk of bias due to deviations from the intended interventions (effect of adhering to intervention))**		
2.1. Were participants aware of their assigned intervention during the trial?	Yes	
2.2. Were carers and people delivering the interventions aware of participants' assigned intervention during the trial?	Yes	
2.3. If Y/PY/NI to 2.1 or 2.2: Were important non protocol interventions balanced across intervention groups?	NA	
2.4. [If applicable:] Were there failures in implementing the intervention that could have affected the outcome?	No	
2.5. [If applicable:] Was there non-adherence to the assigned intervention regimen that could have affected participants' outcomes?	Yes	
2.6. If N/PN/NI to 2.3, or Y/PY/NI to 2.4 or 2.5: Was an appropriate analysis used to estimate the effect of adhering to the intervention?	No	Dose-response could have been informative but they did not prove that the criteria needed to use this mehtod were met
**Risk of bias judgement**	Low/**High**/Some Concerns	
**Bias due to missing data**		Notes
5.1 Were outcome data available for all, or nearly all, participants?	No	Outcome data was only available for 12%of participants
5.2 Were participants excluded due to missing data on intervention status?	Probably No	28 participants in the full group were excluded based on having missing demographic data
5.3 Were participants excluded due to missing data on other variables needed for the analysis?	No	
5.4 If PN/N to 5.1, or Y/PY to 5.2 or 5.3: Are the proportion of participants and reasons for missing data similar across interventions?	NA	Only one group
5.5 If PN/N to 5.1, or Y/PY to 5.2 or 5.3: Is there evidence that results were robust to the presence of missing data?	Probably No	The subsample seemed similar to the full sample but this was not tested<br>It is likely that the included group engaged more often with the application than the full sample
**Bias in measurement of outcomes**		Notes
6.1 Could the outcome measure have been influenced by knowledge of the intervention received?	Yes	If the intervention received is considered the usage of the app then greater app usage could have translated to a greater amount of time between the first and second a1c values
6.2 Were outcome assessors aware of the intervention received by study participants? (blinding of assessors)	Yes	Self-reported A1c values by unblinded participants
6.3 Were the methods of outcome assessment comparable across intervention groups?	NA	
6.4 Were any systematic errors in measurement of the outcome related to intervention received?	Yes	If the intervention received is considered the usage of the app then greater app usage could have translated to a greater amount of time between the first and second a1c values
**Bias in selection of the reported result**		Notes
Were the data that produced this result analysed in accordance with a pre-specified analysis plan that was finalized before unblinded outcome data were available for analysis?	No	
Is the reported effect estimate likely to be selected, on the basis of the results, from...		
7.1. ... multiple outcome measurements within the outcome domain?	Probably yes	Because the analysis is not preregistered, it is unclear if researchers originally intended to use exact times for a1c values and what the original plan for dealing with more than 2 alc values was
7.2 ... multiple analyses of the intervention-outcome relationship?	Probably yes	Not clear since this was not a preregistered study
7.3 ... different subgroups?	Probably yes	Not clear since this was not a preregistered study
**Secondary Analysis RoB Assessment**		Notes
**Is the seconday analysis evaluating effectiveness or efficacy?**		
**Comparator**		
**Outcome Being Assessed**		

**Specify the Numerical Result Being Assessed**		
**Bias due to randomization (See primary analysis section)**		Notes
**Bias due to confounding**		Notes
1.1 Is there potential for confounding of the effect of intervention in this study?		
1.2. If Y/PY to 1.1 Was the analysis based on splitting participants' follow up time according to intervention received?		
1.3. If Y/PY to 1.2 Were intervention discontinuations or switches likely to be related to factors that are prognostic for the outcome?		
**Questions relating to baseline confounding only**		
1.4. If Y/PY to 1.1 Did the authors use an appropriate analysis method that controlled for all the important confounding domains?		
1.5. If Y/PY to 1.4: Were confounding domains that were controlled for measured validly and reliably by the variables available in this study?		
1.6. Did the authors control for any post-intervention variables that could have been affected by the intervention?		
**Questions relating to baseline and time-varying confounding**		
1.7. If Y/PY to 1.3 Did the authors use an appropriate analysis method that controlled for all the important confounding domains and for time-varying confounding?		
1.8. If Y/PY to 1.7: Were confounding domains that were controlled for measured validly and reliably by the variables available in this study?		
**Bias in selection of participants into the study (Not evaluating for analyses of effectiveness)**		Notes
2.1. Was selection of participants into the analysis based on participant characteristics observed after the start of intervention?		
2.2. If Y/PY to 2.1: Were the post-intervention variables that influenced selection likely to be associated with intervention?		
2.3 If Y/PY to 2.2: Were the post-intervention variables that influenced selection likely to be influenced by the outcome or a cause of the outcome?		
2.4. Do start of follow-up and start of intervention coincide for most participants?		
2.5. If Y/PY to 2.2 and 2.3, or N/PN to 2.4: Were adjustment techniques used that are likely to correct for the presence of selection biases?		
**Bias in classification of interventions**		Notes
3.1 Were intervention groups clearly defined?		
3.2 Was the information used to define intervention groups recorded at the start of the intervention?		
3.3 Could classification of intervention status have been affected by knowledge of the outcome or risk of the outcome?		
**Bias due to deviations from intended interventions (See primary analysis section)**		Notes
**Bias due to missing data**		Notes
5.1 Were outcome data available for all, or nearly all, participants?		
5.2 Were participants excluded due to missing data on intervention status?		
5.3 Were participants excluded due to missing data on other variables needed for the analysis?		
5.4 If PN/N to 5.1, or Y/PY to 5.2 or 5.3: Are the proportion of participants and reasons for missing data similar across interventions?		
5.5 If PN/N to 5.1, or Y/PY to 5.2 or 5.3: Is there evidence that results were robust to the presence of missing data?		
**Bias in measurement of outcomes**		Notes
6.1 Could the outcome measure have been influenced by knowledge of the intervention received?		
6.2 Were outcome assessors aware of the intervention received by study participants? (blinding of assessors)		
6.3 Were the methods of outcome assessment comparable across intervention groups?		
6.4 Were any systematic errors in measurement of the outcome related to intervention received?		
6.5 Could adherence have been influenced by study participation?		
**Bias in selection of the reported result**		Notes
Were the data that produced this result analysed in accordance with a pre-specified analysis plan that was finalized before unblinded outcome data were available for analysis?		
Is the reported effect estimate likely to be selected, on the basis of the results, from...		
7.1. ... multiple outcome measurements within the outcome domain?		
7.2 ... multiple analyses of the intervention-outcome relationship?		
7.3 ... different subgroups?		

Criteria for Adherence Metrics and Analysis		Notes
**Device name** (indication for use)	Mahana (cognitive behavioral therapy for IBS)	
**Type of device** (Long-term vs short-term use)	Short-term use	
**Type of device** (Presence or absence of a prescribed dosage)	Presence of a prescribed dosage	
**Was adherence information collected?**	Yes	
**What information was collected about usage adherence?**	Completion of web sessions<br>Completion of telephone sessions	
**What information was collected about adherence to recommendations?**	NA	No explicit app recommendations
**Was information collected internally in the app?**	Yes	
**Was information modified internally?**	NA	
**Was initiation reported?**	No	
**What was average adherence?** (if reported)		
**Was implementation reported?**	Yes	Defined as completing four of the telephone calls (not including booster sessions) for the TCBT arm and as four or more of the website sessions and at least one telephone call (not including booster sessions) for the WCBT arm
**What was average adherence?** (if reported)	"For TCBT arm, 84.4% of participants completed at least four phone calls. For WCBT arm, 88.1% of participants completed at least one phone call, and 69.2% completed four web sessions."	
**Was persistence reported?**	No	Only discussed drop-outs from the trial and not persistence in terms of app use
**What was average adherence?** (if reported)		
**Was adherence low?**	Yes	Less than 80% of participants completed four web sessions
**Was adherence not reported or low?**	Yes	
**Was efficacy analyzed?**	Yes	
**What method was used?**	CACE	
**Were efficacy analyses preregistered?**	Yes	
**What assumptions are required for that method to study efficacy?**	SUTVA, positivity, consistency, exclusion restriction, strong monotonicity, ignorability	
**Did the article report evidence that the assumptions were met?**	Yes	
**SUTVA**	Yes	There could have been crossover effects from the therapists but they tested to make sure that treatment was consistent across all providers regardless of treatment group
**Positivity**	Yes	Randomized trial
**Consistency** (treatment definition)	Yes	Clear description of interventions
**Consistency** (adherence definition)	Yes	Clear preregistered definition of adherence
**Exclusion restriction**	Yes	Randomized trial
**Strong Monotonicity**	Yes	Randomized trial
**Ignorability**	Yes	Randomized trial
**Overall Notes**		
Cochrane CDPLG		Notes
**Data form completed date** (dd/mm/yyyy)	2/16/2022	
**Study author contact details**	hae1@soton.ac.uk	
Methods	**Descriptions as stated in report/paper**	**Location in text or source** (pg & ¶/fig/table/other)
Aim of study (e.g. efficacy, equivalence, pragmatic)	Effectiveness	pg. 1 Abstract
Design (e.g. parallel, crossover, non-RCT)	Parallel RCT	pg. 2 Study Design and Participants
Unit of allocation (by individuals, cluster/ groups or body parts)	Individual	pg. 2 Randomizaton and Masking
Participants	**Descriptions as stated in report/paper**	**Location in text or source** (pg & ¶/fig/table/other)
**Inclusion criteria**	"Participants were eligible if they fulfilled criteria for refractory IBS at screening, defined as: fulfilling ROME III criteria for IBS; reported ongoing clinically significant symptoms on IBS Symptom Severity Score (IBS-SSS), that is, ≧75; had been offered first-line therapies (eg, antispasmodics, antidepressants or fibre-based medications); and had IBS symptoms ≧12months. Due to the increased risk of bowel cancer, potential participants aged >60years were only included if they had hospital consultant review ≤2 years to confirm symptoms were IBS related and exclude serious bowel conditions."	pg. 2 Study Design and Participants
**Exclusion criteria**	"Medical exclusion criteria: unexplained rectal bleeding or weight loss, IBD, coeliac disease, peptic ulcer disease, colorectal carcinoma. Other exclusions: patients <18years, unable to participate in CBT due to speech or language difficulties, no access to internet computer, received CBT in the last 2years, previous access to Regul8 during MIBS trial, currently participating in another IBS intervention trial."	pg. 2 Study Design and Participants
**Total no. randomised** (or total pop. at start of study for NRCTs)	558	pg. 4 Figure 1
**Clusters** (if applicable, no., type, no. people per cluster)	NA	
**Withdrawals and exclusions** (if not provided below by outcome)	166 drop-outs in 12 months	pg. 6 Results
Intervention Groups	**Descriptions as stated in report/paper**	**Location in text or source** (pg & ¶/fig/table/other)
**Group name**	Telephone-delivered CBT (TCBT)	pg. 1 Abstract
**No. randomised to group** (specify whether no. people or clusters)	186	pg. 4 Figure 1
**Timing** (e.g. frequency, duration of each episode)	"Participants randomised to TCBT arm received a detailed self-help manual including homework tasks and had six 1-hour telephone sessions with a CBT therapist at weeks 1, 2, 3, 5, 7 and 9. They also received two 1-hour booster sessions at 4 and 8months (total 8hours of therapist support)."	pg. 3 Description of CBT interventions and TAU
**Co-interventions**	Treatment as usual (TAU)	pg. 2 Description of CBT interventions and TAU
**Integrity of delivery**	No info about how many people did not receive at least one session	
**Compliance**	48 drop-outs in 12 months	pg. 4 Figure 1
Intervention Groups	**Descriptions as stated in report/paper**	**Location in text or source** (pg & ¶/fig/table/other)
**Group name**	Web-based CBT (WCBT) with minimal therapist support	pg. 1 Abstract
**No. randomised to group** (specify whether no. people or clusters)	85	pg. 4 Figure 1
**Timing** (e.g. frequency, duration of each episode)	"WCBT participants received online access to Regul8 and three 30min telephone therapy calls at weeks 1, 3 and 5, and two 30 min booster sessions at 4 and 8 months (2.5 hours of therapist support)."	pg. 3 Description of CBT interventions and TAU
**Co-interventions**	Treatment as usual (TAU)	pg. 2 Description of CBT interventions and TAU
**Integrity of delivery**	No info about how many people did not receive at least one session	
**Compliance**	61 drop-outs in 12 months	pg. 4 Figure 1
Intervention Groups	**Descriptions as stated in report/paper**	**Location in text or source** (pg & ¶/fig/table/other)
**Group name**	Treatment as usual (TAU)<br>TAU was a continuation of current medications and usual GP or consultant follow-up with no psychological therapy	pg. 1 Abstract<br>pg. 3 Description of CBT interventions and TAU
**No. randomised to group** (specify whether no. people or clusters)	187	pg. 4 Figure 1
**Timing** (e.g. frequency, duration of each episode)		
**Co-interventions**		
**Integrity of delivery**		
**Compliance**	55 drop-outs in 12 months	pg. 4 Figure 1
Outcomes	**Descriptions as stated in report/paper**	**Location in text or source** (pg & ¶/fig/table/other)
**Outcome name**	IBS-SSS at 12 months post randomization	pg. 3 Primary Outcomes
**Outcome definition** (with diagnostic criteria if relevant)(include name, time, and analysis method)	"Measures severity and duration of abdominal pain, abdominal distension/tightness, bowel habit and quality of life (score 0–500)"	pg. 3 Primary Outcomes
**Imputation of missing data** (e.g. assumptions made for ITT analysis)	MI imputation model	pg. 5 Statistical Analysis
**Power** (e.g. power & sample size calculation, level of power achieved)	Sample size calculated for 90% power for a 35 point difference	pg. 3 Sample Size
Outcomes	**Descriptions as stated in report/paper**	**Location in text or source** (pg & ¶/fig/table/other)

Article Key

**Outcome name**	WSAS at 12 months post randomization	pg. 3 Primary Outcomes
**Outcome definition** (with diagnostic criteria if relevant)(include name, time, and analysis method)	"Measures the effect of IBS on ability to work and manage at home, participate in social and private leisure activities and maintain relationships. WSAS is sensitive to change in IBS and has five domains scored 0 (not affected) to 8 (severely affected), with a total possible score of 40"	pg. 3 Primary Outcomes
**Imputation of missing data** (e.g. assumptions made for ITT analysis)	MI imputation model	pg. 5 Statistical Analysis
**Power** (e.g. power & sample size calculation, level of power achieved)	Sample size calculated for 90% power for a 3.7 point difference	pg. 3 Sample Size
**Risk of Bias** (Based on Cochrane RoB Tool 2 and ROBINS-I)		Link to RoB Algorithm
**Primary Analysis RoB Assessment**		Notes
**Is the primary analysis evaluating effectiveness or efficacy?**	Effectiveness	
**Comparator**	TAU vs WCBT	
**Outcome Being Assessed**	ITT Effect of WBCT compared to TAU at 12 months for IBS-SSS<br>ITT Effect of WBCT compared to TAU at 12 months for WSAS	
**Specify the Numerical Result Being Assessed**	35.2 (95% CI 12.6 to 57.8) points lower (p=0.002)<br>3.0 (95% CI 1.3 to 4.6) points lower (p=0.001)	
**Bias due to randomization (RCT only)**		Notes
1.1 Was the allocation sequence random?	Yes	Randomization was conducted by an outside service
1.2. Was the allocation sequence concealed until participants were enrolled and assigned to interventions?	Yes	
1.3. Did baseline differences between intervention groups suggest a problem with the randomization process?	No	
**Bias due to confounding**		Notes
1.1 Is there potential for confounding of the effect of intervention in this study?	No	Confounders not likely to affect the results due to randomization and ITT analysis
1.2. If Y/PY to 1.1 Was the analysis based on splitting participants' follow up time according to intervention received?		
1.3. If Y/PY to 1.2 Were intervention discontinuations or switches likely to be related to factors that are prognostic for the outcome?		
**Questions relating to baseline confounding only**		
1.4. If Y/PY to 1.1 Did the authors use an appropriate analysis method that controlled for all the important confounding domains?		
1.5. If Y/PY to 1.4: Were confounding domains that were controlled for measured validly and reliably by the variables available in this study?		
1.6. Did the authors control for any post-intervention variables that could have been affected by the intervention?		
**Questions relating to baseline and time-varying confounding**		
1.7. If Y/PY to 1.3 Did the authors use an appropriate analysis method that controlled for all the important confounding domains and for time-varying confounding?		
1.8. If Y/PY to 1.7: Were confounding domains that were controlled for measured validly and reliably by the variables available in this study?		
**Bias in selection of participants into the study (Not evaluating for analyses of effectiveness)**		Notes
2.1. Was selection of participants into the analysis based on participant characteristics observed after the start of intervention?		
2.2. If Y/PY to 2.1: Were the post-intervention variables that influenced selection likely to be associated with intervention?		
2.3 If Y/PY to 2.2: Were the post-intervention variables that influenced selection likely to be influenced by the outcome or a cause of the outcome?		
2.4. Do start of follow-up and start of intervention coincide for most participants?		
2.5. If Y/PY to 2.2 and 2.3, or N/PN to 2.4: Were adjustment techniques used that are likely to correct for the presence of selection biases?		
**Bias in classification of interventions**		Notes
3.1 Were intervention groups clearly defined? Was the definition of adherence clearly defined?	Yes	
3.2 Was the information used to define intervention groups recorded at the start of the intervention?	Yes	Intervnetion groups were defined in published protocol
3.3 Could classification of intervention status have been affected by knowledge of the outcome or risk of the outcome?	No	
**Bias due to deviations from intended interventions**		Notes
**ROBINS-I**		
4.1. Were there deviations from the intended intervention beyond what would be expected in usual practice?	No	
4.2. If Y/PY to 4.1: Were these deviations from intended intervention unbalanced between groups and likely to have affected the outcome?		
4.3. Were important co-interventions balanced across intervention groups?	Probably Yes	They clearly label the groups in this but the product is not marketed for use with telephone CBT
4.4. Was the intervention implemented successfully for most participants?	Probably Yes	This aspect of adherence isn't mentioned but most participants finished 4 web sessions
4.5. Did study participants adhere to the assigned intervention regimen?	No	69.2% of participants completed 4 web sessions
4.6. If N/PN to 4.3, 4.4 or 4.5: Was an appropriate analysis used to estimate the effect of starting and adhering to the intervention?	Yes	CACE was used to evaluate the effectiveness of the app for the adherent subgroup
Risk of bias: Assignment to intervention	**Low** / Moderate / Serious / Critical / NI	
Risk of bias: Starting and adhering to intervention	**Low** / Moderate / Serious / Critical / NI	
**Risk of bias judgement**	**Low** / Moderate / Serious / Critical / NI	
**RoB Tool (Risk of bias due to deviations from the intended interventions (effect of adhering to intervention))**		
2.1. Were participants aware of their assigned intervention during the trial?	Yes	
2.2. Were carers and people delivering the interventions aware of participants' assigned intervention during the trial?	Yes	
2.3. If Y/PY/NI to 2.1 or 2.2: Were important non protocol interventions balanced across intervention groups?	NA	
2.4. [If applicable:] Were there failures in implementing the intervention that could have affected the outcome?	No information	This aspect of adherence isn't mentioned but most participants finished 4 web sessions
2.5. [If applicable:] Was there non-adherence to the assigned intervention regimen that could have affected participants' outcomes?	Yes	30.8% did not complete 4 web sessions and this was related to missingness of outcomes
2.6. If N/PN/NI to 2.3, or Y/PY/NI to 2.4 or 2.5: Was an appropriate analysis used to estimate the effect of adhering to the intervention?	Yes	CACE was used to evaluate the effectiveness of the app for the adherent subgroup
**Risk of bias judgement**	Low/High/**Some Concerns**	
**Bias due to missing data**		Notes
5.1 Were outcome data available for all, or nearly all, participants?	No	166 participants dropped out
5.2 Were participants excluded due to missing data on intervention status?	No	
5.3 Were participants excluded due to missing data on other variables needed for the analysis?	No	
5.4 If PN/N to 5.1, or Y/PY to 5.2 or 5.3: Are the proportion of participants and reasons for missing data similar across interventions?	Yes	
5.5 If PN/N to 5.1, or Y/PY to 5.2 or 5.3: Is there evidence that results were robust to the presence of missing data?	Yes	MICE was used to impute missing data and they report the results with CC in an appendix
**Bias in measurement of outcomes**		Notes
6.1 Could the outcome measure have been influenced by knowledge of the intervention received?	Yes	Participants were not blind to the intervention
6.2 Were outcome assessors aware of the intervention received by study participants? (blinding of assessors)	Yes	Self-reported outcomes
6.3 Were the methods of outcome assessment comparable across intervention groups?	Yes	
6.4 Were any systematic errors in measurement of the outcome related to intervention received?	No	
**Bias in selection of the reported result**		Notes

Were the data that produced this result analysed in accordance with a pre-specified analysis plan that was finalized before unblinded outcome data were available for analysis?	Yes	
Is the reported effect estimate likely to be selected, on the basis of the results, from...		
7.1. ... multiple outcome measurements within the outcome domain?	No	
7.2 ... multiple analyses of the intervention-outcome relationship?	No	
7.3 ... different subgroups?	No	https://bmjopen.bmj.com/content/bmjopen/5/7/e008622.full.pdf
**Secondary Analysis RoB Assessment**		Notes
**Is the seconday analysis evaluating effectiveness or efficacy?**	Efficacy	
**Comparator**	TAU vs WCBT	
**Outcome Being Assessed**	IBS-SSS CACE<br>WSAS CACE	
**Specify the Numerical Result Being Assessed**	-50.4 points (CI from -75.2 to -25.5, p<0.001) for WCBT compared with TAU<br>-4.2 points (CI from -5.9 to -2.4, p<0.001) for WCBT compared with TAU	
**Bias due to randomization (See primary analysis section)**		Notes
**Bias due to confounding**		Notes
1.1 Is there potential for confounding of the effect of intervention in this study?	Yes	Those that dropped from the study were more likely to have missing data
1.2. If Y/PY to 1.1 Was the analysis based on splitting participants' follow up time according to intervention received?	No	
1.3. If Y/PY to 1.2 Were intervention discontinuations or switches likely to be related to factors that are prognostic for the outcome?		
**Questions relating to baseline confounding only**		
1.4. If Y/PY to 1.1 Did the authors use an appropriate analysis method that controlled for all the important confounding domains?	Yes	They used regression to see what was most predictive of having missing data and included those in the model
1.5. If Y/PY to 1.4: Were confounding domains that were controlled for measured validly and reliably by the variables available in this study?	Yes	
1.6. Did the authors control for any post-intervention variables that could have been affected by the intervention?	No	
**Questions relating to baseline and time-varying confounding**		
1.7. If Y/PY to 1.3 Did the authors use an appropriate analysis method that controlled for all the important confounding domains and for time-varying confounding?		
1.8. If Y/PY to 1.7: Were confounding domains that were controlled for measured validly and reliably by the variables available in this study?		
**Bias in selection of participants into the study (Not evaluating for analyses of effectiveness)**		Notes
2.1. Was selection of participants into the analysis based on participant characteristics observed after the start of intervention?	No	Because CACE was used, they did not need to select a subgroup of participants into the analysis
2.2. If Y/PY to 2.1: Were the post-intervention variables that influenced selection likely to be associated with intervention?		
2.3 If Y/PY to 2.2: Were the post-intervention variables that influenced selection likely to be influenced by the outcome or a cause of the outcome?		
2.4. Do start of follow-up and start of intervention coincide for most participants?	Yes	
2.5. If Y/PY to 2.2 and 2.3, or N/PN to 2.4: Were adjustment techniques used that are likely to correct for the presence of selection biases?		
**Bias in classification of interventions**		Notes
3.1 Were intervention groups clearly defined?	Yes	Adherence was clearly defined
3.2 Was the information used to define intervention groups recorded at the start of the intervention?	Yes	The definition was preregistered
3.3 Could classification of intervention status have been affected by knowledge of the outcome or risk of the outcome?	No	
**Bias due to deviations from intended interventions (See primary analysis section)**		Notes
**Bias due to missing data**		Notes
5.1 Were outcome data available for all, or nearly all, participants?	No	166 participants dropped out
5.2 Were participants excluded due to missing data on intervention status?	Yes	No imputation was used for analyses of efficacy
5.3 Were participants excluded due to missing data on other variables needed for the analysis?	Probably Yes	There is a similar amount of missing outcomes for each group but they don't say why
5.4 If PN/N to 5.1, or Y/PY to 5.2 or 5.3: Are the proportion of participants and reasons for missing data similar across interventions?	Yes	They used regression to see what was most predictive of having missing data and included those predictors in the model
5.5 If PN/N to 5.1, or Y/PY to 5.2 or 5.3: Is there evidence that results were robust to the presence of missing data?		
**Bias in measurement of outcomes**		Notes
6.1 Could the outcome measure have been influenced by knowledge of the intervention received?	Yes	
6.2 Were outcome assessors aware of the intervention received by study participants? (blinding of assessors)	Yes	
6.3 Were the methods of outcome assessment comparable across intervention groups?	Yes	
6.4 Were any systematic errors in measurement of the outcome related to intervention received?	No	
6.5 Could adherence have been influenced by study participation?	Yes	Participants could have been more adherent to the web sessions due to the minimal therapy they received
**Bias in selection of the reported result**		Notes
Were the data that produced this result analysed in accordance with a pre-specified analysis plan that was finalized before unblinded outcome data were available for analysis?	Yes	Efficacy analysis was pre-specified
Is the reported effect estimate likely to be selected, on the basis of the results, from...		
7.1. ... multiple outcome measurements within the outcome domain?	No	
7.2 ... multiple analyses of the intervention-outcome relationship?	No	
7.3 ... different subgroups?	No	

Article Key

Criteria for Adherence Metrics and Analysis		Notes
**Device name and indication for use**	Mahana (cognitive behavioral therapy for IBS)	
**Type of device** (Long-term vs short-term use)	Short-term use	
**Type of device** (Presence or absence of a prescribed dosage)	Presence of a prescribed dosage	
**Was adherence information collected?**	Yes	
**What information was collected about usage adherence?**	Completion of telephone sessions<br>Completion of web sessions	
**What information was collected about adherence to recommendations?**	NA	No explicit recommendations
**Was information collected internally in the app?**	Yes	
**Was information modified internally?**	NA	
**Was initiation reported?**	Yes	
**What was average adherence?** (if reported)	11.9% of participants had 0 phone calls and 8.1% of participants completed 0 web sessions	
**Was implementation reported?**	Yes	Percent of participants completing 4 web sessions and 1 phone call
**What was average adherence?** (if reported)	59.20%	
**Was persistence reported?**	Yes	Table 22
**What was average adherence?** (if reported)	3.7% received 4 phone calls; 21.6% completed 8 web sessions	
**Was adherence low?**	Yes	Less than 80% of participants completed 4 web sessions and 1 phone call
**Was adherence not reported or low?**	Yes	
**Was efficacy analyzed?**	Yes	
**What method was used?**	CACE	
**Were efficacy analyses preregistered?**	Yes	Outlined in the SAP
**What assumptions are required for that method to study efficacy?**	SUTVA, positivity, consistency, exclusion restriction, strong monotonicity, ignorability	
**Did the article report evidence that the assumptions were met?**	Yes	
**SUTVA**	Yes	There could have been crossover effects from the therapists but they tested to make sure that treatment was consistent across all providers regardless of treatment group
**Positivity**	Yes	Randomized trial
**Consistency** (treatment definition)	Yes	Clear treatment definition
**Consistency** (adherence definition)	Yes	Clear defintion of adherence
**Exclusion restriction**	Yes	Randomized trial
**Strong Monotonicity**	Yes	Randomized trial
**Ignorability**	Yes	Randomized trial
**Overall Notes**		
**Cochrane CDPLG**		**Notes**
**Data form completed date** (dd/mm/yyyy)	4/6/2022	
**Study author contact details**	hae1@soton.ac.uk	
Methods	**Descriptions as stated in report/paper**	**Location in text or source** (pg & ¶/fig/table/other)
Aim of study (e.g. efficacy, equivalence, pragmatic)	Clinical Effectiveness	pg. 34 Aims and Objectives
Design (e.g. parallel, crossover, non-RCT)	Randomized Controlled Trial	pg. 34 Aims and Objectives
Unit of allocation (by individuals, cluster/ groups or body parts)	Individual	pg. 39 Randomization
Participants	**Descriptions as stated in report/paper**	**Location in text or source** (pg & ¶/fig/table/other)
**Inclusion criteria**	"Patient is aged ≥ 18 years.<br>Patient has refractory IBS (clinically significant symptoms defined by an IBS SSS of > 75).<br>Patient fulfils Rome III criteria.<br>Patient has been offered first-line therapies (e.g. antispasmodics, antidepressants or fibre-based<br>medications) but still has continuing IBS symptoms for ≥ 12 months.<br>If > 60 years old, patient has had a consultant review in the previous 2 years to confirm that their<br>symptoms are related to IBS and that other serious bowel conditions have been excluded."	pg. 37 Box 1
**Exclusion criteria**	"Patient has unexplained rectal bleeding or weight loss.<br>Patient has a diagnosis of IBD.<br>Patient has coeliac disease.<br>Patient has peptic ulcer disease.<br>Patient has colorectal carcinoma.<br>Patient is unable to participate in CBT because of speech or language difficulties.<br>Patient has no access to an internet-enabled computer to be able to undertake the WCBT.<br>Patient has received CBT for IBS in the past 2 years.<br>Patient has had previous access to the MIBS website.<br>Patient is currently participating in an IBS/intervention trial."	pg. 38 Box 2
**Total no. randomised** (or total pop. at start of study for NRCTs)	558	pg. 55 Baseline Data Summary
**Clusters** (if applicable, no., type, no. people per cluster)	NA	
**Withdrawals and exclusions** (if not provided below by outcome)	Over 12 months 36 participants withdrew from treatment and 45 withdrew from the trial	pg. 564 Table 20
Intervention Groups	**Descriptions as stated in report/paper**	**Location in text or source** (pg & ¶/fig/table/other)
**Group name**	TAU<br>Continuation of current medications and usual GP or consultant follow-up with no psychological therapy, standard information sheet on lifestyle and diet in IBS	pg. 43 Treatment as usual
**No. randomised to group** (specify whether no. people or clusters)	187	pg. 55 Baseline Data Summary
**Timing** (e.g. frequency, duration of each episode)	NA	
**Co-interventions**	NA	
**Integrity of delivery**	NA	
**Compliance**	NA	
Intervention Groups	**Descriptions as stated in report/paper**	**Location in text or source** (pg & ¶/fig/table/other)
**Group name**	Telephone-delivered CBT (TCBT)	pg. 39 Interventions
**No. randomised to group** (specify whether no. people or clusters)	186	pg. 55 Baseline Data Summary
**Timing** (e.g. frequency, duration of each episode)	"six 1-hour telephone sessions with a CBT therapist at weeks 1, 2, 3, 5, 7 and 9 and homework tasks. They also received two 1-hour booster sessions at 4 and 8 months"	pg. 39 Interventions
**Co-interventions**	Treatment as usual (TAU)	pg. 43 Treatment as usual
**Integrity of delivery**	The quality of the therapy sessions was graded	pg. 42 Intervention Fidelity and Supervision
**Compliance**	84% completed 4 phone sessions	
Intervention Groups	**Descriptions as stated in report/paper**	**Location in text or source** (pg & ¶/fig/table/other)
**Group name**	Web-based CBT (WCBT) with minimal therapist support	pg. 39 Interventions
**No. randomised to group** (specify whether no. people or clusters)	85	pg. 55 Baseline Data Summary
**Timing** (e.g. frequency, duration of each episode)	"three 30-minute telephone therapy support calls at weeks 1, 3 and 5 and two 30-minute booster sessions at 4 and 8 months"	pg. 39 Interventions
**Co-interventions**	TAU	pg. 43 Treatment as usual
**Integrity of delivery**	The quality of the therapy sessions was graded	pg. 42 Intervention Fidelity and Supervision
**Compliance**	59.2% completed four web sessions and 1 phone call	
Outcomes	**Descriptions as stated in report/paper**	**Location in text or source** (pg & ¶/fig/table/other)
**Outcome name**	IBS SSS	pg. 43 Primary Outcome Measures
**Outcome definition** (with diagnostic criteria if relevant)(include name, time, and analysis method)	"five-item, self-administered questionnaire measuring severity of abdominal pain, duration of abdominal pain, abdominal distension/tightness, bowel habit and QoL. It has a maximum score of 500: a score of < 75 indicates normal bowel function, 75–174 indicates mild IBS, 175–299 indicates moderate IBS and 300–500 indicates severe IBS."	pg. 43 Primary Outcome Measures

**Imputation of missing data** (e.g. assumptions made for ITT analysis)	A multiple imputation model was used	pg. 48 Imputation Model
**Power** (e.g. power & sample size calculation, level of power achieved)	"We powered this trial to detect a 35-point difference between groups at 12 months for the sample size calculations. This was to account for a 15-point placebo response in the TAU arm (the placebo response is known to be important in IBS, and the MIBS trial1,15 showed a 24-point difference in the no-website group from baseline to 12-week follow-up; thus, allowing for a 15-point placebo response at 12 months was prudent)"<br>"Assuming a within-group IBS SSS standard deviation (SD) of 76 (taken from the MIBS pilot study1), this equated to an effect size of 0.46. To achieve 90% power to detect such an effect or larger using a two-sided independent-samples t-test at the 2.5% significance level (adjusting for two primary outcomes), it was estimated that the trial would require 119 subjects per group. Based on each of 10 therapists delivering therapy to 17 patients in the WCBT and TCBT groups, and an intraclass correlation of 0.02, taken from Baldwin et al., this sample size was increased by an inflation factor of 1.32 to take account of therapist effects. We measured IBS SSS at baseline and assumed that baseline values were predictive of post-treatment values (correlation 0.4). Accounting for this in our statistical analysis model<br>allowed us to decrease the sample size by a deflation factor of 0.84. Finally, we applied a further inflation factor of 1.25 on the assumption that attrition would be < 20%. The final sample size requirement was thus calculated as 165 patients per group or 495 patients in total"<br>"Sample size was later increased based on an updated attrition rate of 30% vs 20%"	pg. 44 Primary Outcome Measures<br>pg. 46 Sample Size
Outcomes	**Descriptions as stated in report/paper**	**Location in text or source** (pg & ¶/fig/table/other)
**Outcome name**	Work and Social Adjustment Scale (WSAS)	pg. 43 Primary Outcome Measures
**Outcome definition** (with diagnostic criteria if relevant)(include name, time, and analysis method)	"The WSAS measures the effect of the IBS on people's ability to work and manage at home and to participate in social and private leisure activities and relationships. WSAS has been shown to be sensitive to change in IBS trials. It has five aspects, each scored from 0 (not affected) to 8 (severely affected), with a possible total score of 40."	pg. 43 Primary Outcome Measures
**Imputation of missing data** (e.g. assumptions made for ITT analysis)	a multiple imputation model was used	pg. 48 Imputation Model
**Power** (e.g. power & sample size calculation, level of power achieved)	"In terms of our second primary outcome (WSAS), the initial 495 sample size was calculated to be sufficient to detect a difference between the WCBT (or TCBT) and TAU groups of ≥ 3.7. Specifically, we assumed inflation factors of 1.32 for correlation of outcomes within therapists and of 1.25 for attrition and a deflation factor of 0.84 for correlation between baseline and follow-up measures. Based on this, a moderate effect size of 0.46 could be found with 90% power at the 2.5% significance level, given 119 participants per group. Assuming a SD of 8.0 (as estimated in a study of CBT for IBS7), this would equate to a treatment difference of 3.7 on this scale. This is less than the difference of 5.4 in change of means in WSAS that was found in a trial of a CBT-based self-management intervention for IBS."<br>"Sample size was later increased based on an updated attrition rate of 30% vs 20%"	pg. 46 Sample Size
**Risk of Bias** (Based on Cochrane RoB Tool 2 and ROBINS-I)		Link to RoB Algorithm
**Primary Analysis RoB Assessment**		Notes
**Is the primary analysis evaluating effectiveness or efficacy?**	Effectiveness	
**Comparator**	TAU vs WCBT	
**Outcome Being Assessed**	ITT Effect of WBCT compared to TAU at 12 months for IBS-SSS<br>ITT Effect of WBCT compared to TAU at 12 months for WSAS	
**Specify the Numerical Result Being Assessed**	IBS SSS: WCBT (p = 0.002, standardised effect = 0.37) (-35.5)<br><br>WSAS: WCBT (p < 0.001, standardised effect = 0.3) (-3.0)	
**Bias due to randomization (RCT only)**		Notes
1.1 Was the allocation sequence random?	Yes	
1.2. Was the allocation sequence concealed until participants were enrolled and assigned to interventions?	Yes	
1.3. Did baseline differences between intervention groups suggest a problem with the randomization process?	No	
**Bias due to confounding**		Notes
1.1 Is there potential for confounding of the effect of intervention in this study?	No	Confounders not likely to affect the results due to randomization and ITT analysis<br><br>They did investigate the impact of therapist effects and include these effects in the model when they had an impact
1.2. If Y/PY to 1.1 Was the analysis based on splitting participants' follow up time according to intervention received?		
1.3. If Y/PY to 1.2 Were intervention discontinuations or switches likely to be related to factors that are prognostic for the outcome?		
**Questions relating to baseline confounding only**		
1.4. If Y/PY to 1.1 Did the authors use an appropriate analysis method that controlled for all the important confounding domains?		
1.5. If Y/PY to 1.4: Were confounding domains that were controlled for measured validly and reliably by the variables available in this study?		
1.6. Did the authors control for any post-intervention variables that could have been affected by the intervention?		
**Questions relating to baseline and time-varying confounding**		
1.7. If Y/PY to 1.3 Did the authors use an appropriate analysis method that controlled for all the important confounding domains and for time-varying confounding?		
1.8. If Y/PY to 1.7: Were confounding domains that were controlled for measured validly and reliably by the variables available in this study?		
**Bias in selection of participants into the study (Not evaluating for analyses of effectiveness)**		Notes
2.1. Was selection of participants into the analysis based on participant characteristics observed after the start of intervention?		
2.2. If Y/PY to 2.1: Were the post-intervention variables that influenced selection likely to be associated with intervention?		
2.3 If Y/PY to 2.2: Were the post-intervention variables that influenced selection likely to be influenced by the outcome or a cause of the outcome?		
2.4. Do start of follow-up and start of intervention coincide for most participants?		
2.5. If Y/PY to 2.2 and 2.3, or N/PN to 2.4: Were adjustment techniques used that are likely to correct for the presence of selection biases?		
**Bias in classification of interventions**		Notes
3.1 Were intervention groups clearly defined? Was the definition of adherence clearly defined?	Yes	
3.2 Was the information used to define intervention groups recorded at the start of the intervention?	Yes	Intervnetion groups were defined in published protocol
3.3 Could classification of intervention status have been affected by knowledge of the outcome or risk of the outcome?	No	
**Bias due to deviations from intended interventions**		Notes
**ROBINS-I**		
4.1. Were there deviations from the intended intervention beyond what would be expected in usual practice?	Yes	They followed up with participants when they did not show to therapy appointments
4.2. If Y/PY to 4.1: Were these deviations from intended intervention unbalanced between groups and likely to have affected the outcome?	No	
4.3. Were important co-interventions balanced across intervention groups?	Yes	
4.4. Was the intervention implemented successfully for most participants?	Yes	

4.5. Did study participants adhere to the assigned intervention regimen?	No	59.2% of participants met their definition of adherence
4.6. If N/PN to 4.3, 4.4 or 4.5: Was an appropriate analysis used to estimate the effect of starting and adhering to the intervention?	Yes	Analyzed with CACE and it was preregistered
Risk of bias: Assignment to intervention	**Low** / Moderate / Serious / Critical / NI	
Risk of bias: Starting and adhering to intervention	Low / **Moderate** / Serious / Critical / NI	
**Risk of bias judgement**	Low / **Moderate** / Serious / Critical / NI	
**RoB Tool (Risk of bias due to deviations from the intended interventions (effect of adhering to intervention))**		
2.1. Were participants aware of their assigned intervention during the trial?	Yes	
2.2. Were carers and people delivering the interventions aware of participants' assigned intervention during the trial?	Yes	
2.3. If Y/PY/NI to 2.1 or 2.2: Were important non protocol interventions balanced across intervention groups?	Yes	
2.4. [If applicable:] Were there failures in implementing the intervention that could have affected the outcome?	Probably No	A small amount of participants did not start the intervention
2.5. [If applicable:] Was there non-adherence to the assigned intervention regimen that could have affected participants' outcomes?	Yes	59.2% of participants did not meet their definition of adherence
2.6. If N/PN/NI to 2.3, or Y/PY/NI to 2.4 or 2.5: Was an appropriate analysis used to estimate the effect of adhering to the intervention?	Yes	Analyzed with CACE and it was preregistered
**Risk of bias judgement**	Low/High/**Some Concerns**	
**Bias due to missing data**		Notes
5.1 Were outcome data available for all, or nearly all, participants?	No	Outcome data was not available for ~30% of participants in each group
5.2 Were participants excluded due to missing data on intervention status?	No	
5.3 Were participants excluded due to missing data on other variables needed for the analysis?	No	
5.4 If PN/N to 5.1, or Y/PY to 5.2 or 5.3: Are the proportion of participants and reasons for missing data similar across interventions?	Yes	
5.5 If PN/N to 5.1, or Y/PY to 5.2 or 5.3: Is there evidence that results were robust to the presence of missing data?	Yes	They used multiple imputation to account for missing data that was not missing at random. The need for MI was assessed by an outside statistician to maintain blinding
**Bias in measurement of outcomes**		Notes
6.1 Could the outcome measure have been influenced by knowledge of the intervention received?	Probably No	There could have been a motivation to respond positively if you were in the treatment group and it was not an objective measurement
6.2 Were outcome assessors aware of the intervention received by study participants? (blinding of assessors)	Probably No	Outcomes were self assessed when possible but were blindly assessed when participants did not respond to the survey in time
6.3 Were the methods of outcome assessment comparable across intervention groups?	Yes	
6.4 Were any systematic errors in measurement of the outcome related to intervention received?	Probably No	
**Bias in selection of the reported result**		Notes
Were the data that produced this result analysed in accordance with a pre-specified analysis plan that was finalized before unblinded outcome data were available for analysis?	Yes	This is an example of the level of detail that an analysis should be preregistered with
Is the reported effect estimate likely to be selected, on the basis of the results, from...		
7.1. ... multiple outcome measurements within the outcome domain?	No	
7.2 ... multiple analyses of the intervention-outcome relationship?	No	
7.3 ... different subgroups?	No	
**Secondary Analysis RoB Assessment**		Notes
**Is the seconday analysis evaluating effectiveness or efficacy?**	Efficacy	
**Comparator**	WCBT vs TAU	
**Outcome Being Assessed**	IBS SSS and WSAS 12 month difference with CACE	
**Specify the Numerical Result Being Assessed**	IBS SSS: 50.4 (95% CI 75.2 to 25.5; p < 0.001) for WCBT<br><br>WSAS: –4.2 (95% CI –5.9 to –2.4 points; p < 0.001)	
**Bias due to randomization (See primary analysis section)**		Notes
**Bias due to confounding**		Notes
1.1 Is there potential for confounding of the effect of intervention in this study?	Probably No	Analyzed with CACE and it was a randomized controlled trial<br>They also tried to control for missing data but did not have the right information collected to do so
1.2. If Y/PY to 1.1 Was the analysis based on splitting participants' follow up time according to intervention received?		
1.3. If Y/PY to 1.2 Were intervention discontinuations or switches likely to be related to factors that are prognostic for the outcome?		
**Questions relating to baseline confounding only**		
1.4. If Y/PY to 1.1 Did the authors use an appropriate analysis method that controlled for all the important confounding domains?		
1.5. If Y/PY to 1.4: Were confounding domains that were controlled for measured validly and reliably by the variables available in this study?		
1.6. Did the authors control for any post-intervention variables that could have been affected by the intervention?		
**Questions relating to baseline and time-varying confounding**		
1.7. If Y/PY to 1.3 Did the authors use an appropriate analysis method that controlled for all the important confounding domains and for time-varying confounding?		
1.8. If Y/PY to 1.7: Were confounding domains that were controlled for measured validly and reliably by the variables available in this study?		
**Bias in selection of participants into the study (Not evaluating for analyses of effectiveness)**		Notes
2.1. Was selection of participants into the analysis based on participant characteristics observed after the start of intervention?	No	
2.2. If Y/PY to 2.1: Were the post-intervention variables that influenced selection likely to be associated with intervention?		
2.3 If Y/PY to 2.2: Were the post-intervention variables that influenced selection likely to be influenced by the outcome or a cause of the outcome?		
2.4. Do start of follow-up and start of intervention coincide for most participants?	Yes	
2.5. If Y/PY to 2.2 and 2.3, or N/PN to 2.4: Were adjustment techniques used that are likely to correct for the presence of selection biases?		
**Bias in classification of interventions**		Notes
3.1 Were intervention groups clearly defined?	Yes	
3.2 Was the information used to define intervention groups recorded at the start of the intervention?	Yes	
3.3 Could classification of intervention status have been affected by knowledge of the outcome or risk of the outcome?	No	
**Bias due to deviations from intended interventions (See primary analysis section)**		Notes
**Bias due to missing data**		Notes
5.1 Were outcome data available for all, or nearly all, participants?	No	Missing for about 30% of participants
5.2 Were participants excluded due to missing data on intervention status?	No	
5.3 Were participants excluded due to missing data on other variables needed for the analysis?	No	
5.4 If PN/N to 5.1, or Y/PY to 5.2 or 5.3: Are the proportion of participants and reasons for missing data similar across interventions?	Yes	

5.5 If PN/N to 5.1, or Y/PY to 5.2 or 5.3: Is there evidence that results were robust to the presence of missing data?	Probably Yes	They incorporated baseline values and randomization stratifier into the model to account for missing data but could not run a sensitivity analysis based on the assumption that the data was not missing at random
**Bias in measurement of outcomes**		Notes
6.1 Could the outcome measure have been influenced by knowledge of the intervention received?	Probably No	
6.2 Were outcome assessors aware of the intervention received by study participants? (blinding of assessors)	Probably No	
6.3 Were the methods of outcome assessment comparable across intervention groups?	Yes	
6.4 Were any systematic errors in measurement of the outcome related to intervention received?	Probably No	
6.5 Could adherence have been influenced by study participation?	Probably Yes	Participants were called if they did not show up to therapy appointments
**Bias in selection of the reported result**		Notes
Were the data that produced this result analysed in accordance with a pre-specified analysis plan that was finalized before unblinded outcome data were available for analysis?	Yes	
Is the reported effect estimate likely to be selected, on the basis of the results, from...		
7.1. ... multiple outcome measurements within the outcome domain?	No	
7.2 ... multiple analyses of the intervention-outcome relationship?	No	
7.3 ... different subgroups?	No	

Criteria for Adherence Metrics and Analysis		Notes
**Device name and indication for use**	Mahana (cognitive behavioral therapy for IBS)	
**Type of device** (Long-term vs short-term use)	Short-term use	
**Type of device** (Presence or absence of a prescribed dosage)	Presence of a prescribed dosage	
**Was adherence information collected?**	Yes	
**What information was collected about usage adherence?**	Web module completion and telephone based session completion	
**What information was collected about adherence to recommendations?**	NA	No explicit app recommendations
**Was information collected internally in the app?**	Yes	
**Was information modified internally?**	NA	Adherence was not modified
**Was initiation reported?**	No	
**What was average adherence?** (if reported)		
**Was implementation reported?**	Yes	Dichotomized based on completing four telephone sessions in the telephone group, completing four web sessions and one phone call in the web group, and not accessing CBT outside of the trial in all groups
**What was average adherence?** (if reported)	63% of participants were adherent in the web based group	Reported based on all participants instead of breaking out the participants with available 24 month data
**Was persistence reported?**	No	
**What was average adherence?** (if reported)		
**Was adherence low?**	Yes	Less than 80% of participants were adherent
**Was adherence not reported or low?**	Yes	
**Was efficacy analyzed?**	Yes	
**What method was used?**	Per-protocol analysis	
**Were efficacy analyses preregistered?**	No	
**What assumptions are required for that method to study efficacy?**	SUTVA, positivity, consistency, ignorability, conditional independence of adherence and outcomes	
**Did the article report evidence that the assumptions were met?**	No	
**SUTVA**	Yes	There could have been interaction issues given that the therapists worked with different units, but they checked for treatment fidelity
**Positivity**	Yes	Randomization was broken due to excluding people based on adherence which was measured after the trial started, but both groups had people excluded so the people left likely had a non-zero chance of receiving treatment or control
**Consistency** (treatment definition)	Yes	Clear definition of treatment
**Consistency** (adherence definition)	Yes	Clear definition of adherence
**Exclusion restriction**		
**Strong Monotonicity**		
**Ignorability**	known baseline predictors of missingness at 12 months (IMD and IBS-SSS)	Participants were excluded in both the treatment and control groups
**Overall Notes**		
Cochrane CDPLG		Notes
**Data form completed date** (dd/mm/yyyy)	4/4/2022	
**Study author contact details**	hae1@soton.ac.uk	
Methods	**Descriptions as stated in report/paper**	**Location in text or source** (pg & ¶/fig/table/other)
Aim of study (e.g. efficacy, equivalence, pragmatic)	Effectiveness<br>24 month follow-up study	pg. 2 Introduction
Design (e.g. parallel, crossover, non-RCT)	Randomized controlled trial	pg. 2 Introduction
Unit of allocation (by individuals, cluster/ groups or body parts)	Individual	
Participants	**Descriptions as stated in report/paper**	**Location in text or source** (pg & ¶/fig/table/other)
**Inclusion criteria**	"Individuals were eligible if they fulfilled criteria for refractory IBS, defined as fulfilling Rome III criteria for IBS; reported ongoing clinically significant symptoms according to the IBS Symptom Severity Score (IBS-SSS; ie, IBS-SSS≥75); had been offered first-line therapies (eg, antispasmodics, antidepressants, or fibre-based medications); and had had IBS symptoms for 12 months or longer"	pg. 2 Study Design and Participants
**Exclusion criteria**	"Medical exclusion criteria were unexplained rectal bleeding or weight loss, inflammatory bowel disease, coeliac disease, peptic ulcer disease, and colorectal carcinoma. Additionally, patients were excluded if they were younger than 18 years, were unable to participate in CBT because of speech or language difficulties, had no access to an internet-connected computer, had received CBT in the previous 2 years, had had previous access to the web CBT for IBS intervention (Regul8) during the Management of Irritable Bowel Syndrome in Primary Care (MIBS) trial, or were currently participating in another IBS intervention trial"	pg. 3 Study Design and Participants
**Total no. randomised** (or total pop. at start of study for NRCTs)	558	pg. 5 Results
**Clusters** (if applicable, no., type, no. people per cluster)	NA	
**Withdrawals and exclusions** (if not provided below by outcome)	323 participants provided data for the 24 month follow-up	pg. 5 Results
Intervention Groups	**Descriptions as stated in report/paper**	**Location in text or source** (pg & ¶/fig/table/other)
**Group name**	Treatment as Usual (TAU)	pg. 3 Procedures
**No. randomised to group** (specify whether no. people or clusters)	105	pg. 5 Results
**Timing** (e.g. frequency, duration of each episode)	"continuation of current medications"<br>"usual general practitioner or consultant follow-up with no psychological therapy"<br>"all participants received a standard information sheet on lifestyle and diet in IBS based on NICE guidance"	pg. 3 Procedures
**Co-interventions**	Access to Regul8 website without therapist support after the 12 month trial	pg. 3 Procedures
**Integrity of delivery**	No specific information about integrity of delivery	
**Compliance**	20 participants accessed CBT during the 24 month period (10 with regul8)	pg. 7 Table 4
Intervention Groups	**Descriptions as stated in report/paper**	**Location in text or source** (pg & ¶/fig/table/other)
**Group name**	Therapist Delivered Telephone CBT	pg. 3 Procedures
**No. randomised to group** (specify whether no. people or clusters)	119	pg. 5 Results
**Timing** (e.g. frequency, duration of each episode)	"Participants randomised to the telephone-CBT group received a detailed self-management CBT manual including homework tasks and recording sheets and were offered six 1-h telephone sessions with a CBT therapist at week 1, 2, 3, 5, 7, and 9. They also received two 1-h booster sessions at 4 months and 8 months (a total of 8 h of therapist support)"	pg. 3 Procedures
**Co-interventions**	TAU	pg. 3 Procedures
**Integrity of delivery**	No specific information about integrity of delivery	
**Compliance**	43 participants were excluded from per-protocol analyses due to not adhering to the allocated treatment during the first 12 month treatment period or from accessing CBT during the second 12 month treatment period	pg. 7 Table 4
Intervention Groups	**Descriptions as stated in report/paper**	**Location in text or source** (pg & ¶/fig/table/other)
**Group name**	Web based CBT with Minimal Therapist Support	pg. 3 Procedures
**No. randomised to group** (specify whether no. people or clusters)	99	pg. 5 Results
**Timing** (e.g. frequency, duration of each episode)	"eight online weekly sessions and homework tasks, and will receive weekly automated email reminders" (from protocol)<br>8 sessions over 9 weeks of treatment<br>"Participants in the web-CBT group received three 30-min therapy-support telephone calls at weeks 1, 3, and 5 and two 30-min booster sessions at 4 months and 8 months (2·5 h of therapist support)."	pg. 3 Procedures<br>Protocol

Article Key

**Co-interventions**	TAU<br>access to the Regul8 website without therapist support after the 12 month trial	pg. 3 Procedures
**Integrity of delivery**	No specific information about integrity of delivery	
**Compliance**	68 participants were excluded due to not adhering to the allocated treament period in the first 12 months or due to seeking CBT treatment during the second 12 month period	pg. 7 Table 4
Outcomes	**Descriptions as stated in report/paper**	**Location in text or source** (pg & ¶/fig/table/other)
**Outcome name**	IBS-SSS	
**Outcome definition** (with diagnostic criteria if relevant)(include name, time, and analysis method)	24-month IBS-SSS analyzed with ITT	
**Imputation of missing data** (e.g. assumptions made for ITT analysis)	Multiple imputation with the flexible multivariate imputation by chained equations approach	
**Power** (e.g. power & sample size calculation, level of power achieved)	No Information	
Outcomes	**Descriptions as stated in report/paper**	**Location in text or source** (pg & ¶/fig/table/other)
**Outcome name**	Work and Social Adjustment Scale (WSAS)	
**Outcome definition** (with diagnostic criteria if relevant)(include name, time, and analysis method)	Impact on life including ability to work, manage daily home tasks and participate in social activites<br>24-month WSAS analyzed with ITT	
**Imputation of missing data** (e.g. assumptions made for ITT analysis)	Multiple imputation with the flexible multivariate imputation by chained equations approach	
**Power** (e.g. power & sample size calculation, level of power achieved)	No Information	
**Risk of Bias** (Based on Cochrane RoB Tool 2 and ROBINS-I)		Link to RoB Algorithm
**Primary Analysis RoB Assessment**		Notes
**Is the primary analysis evaluating effectiveness or efficacy?**	Effectiveness	
**Comparator**	Web based CBT compared to TAU	
**Outcome Being Assessed**	ITT effect at 24 months for IBS-SSS and WSAS	
**Specify the Numerical Result Being Assessed**	At 24 months, mean IBS-SSS was 12·9 points (–12·9 to 38·8; p=0·33) lower in the web-CBT group than in the TAU group<br><br>At 24 months, the mean WSAS score was 1·9 points (0·1 to 3·7; p=0·036) lower in the web-CBT group than in the TAU group	
**Bias due to randomization (RCT only)**		Notes
1.1 Was the allocation sequence random?	Yes	
1.2. Was the allocation sequence concealed until participants were enrolled and assigned to interventions?	Probably Yes	
1.3. Did baseline differences between intervention groups suggest a problem with the randomization process?	No	
**Bias due to confounding**		Notes
1.1 Is there potential for confounding of the effect of intervention in this study?	No	Randomized controlled trial with ITT analysis
1.2. If Y/PY to 1.1 Was the analysis based on splitting participants' follow up time according to intervention received?		
1.3. If Y/PY to 1.2 Were intervention discontinuations or switches likely to be related to factors that are prognostic for the outcome?		
**Questions relating to baseline confounding only**		
1.4. If Y/PY to 1.1 Did the authors use an appropriate analysis method that controlled for all the important confounding domains?		
1.5. If Y/PY to 1.4: Were confounding domains that were controlled for measured validly and reliably by the variables available in this study?		
1.6. Did the authors control for any post-intervention variables that could have been affected by the intervention?	No	
**Questions relating to baseline and time-varying confounding**		
1.7. If Y/PY to 1.3 Did the authors use an appropriate analysis method that controlled for all the important confounding domains and for time-varying confounding?		
1.8. If Y/PY to 1.7: Were confounding domains that were controlled for measured validly and reliably by the variables available in this study?		
**Bias in selection of participants into the study (Not evaluating for analyses of effectiveness)**		Notes
2.1. Was selection of participants into the analysis based on participant characteristics observed after the start of intervention?		
2.2. If Y/PY to 2.1: Were the post-intervention variables that influenced selection likely to be associated with intervention?		
2.3 If Y/PY to 2.2: Were the post-intervention variables that influenced selection likely to be influenced by the outcome or a cause of the outcome?		
2.4. Do start of follow-up and start of intervention coincide for most participants?		
2.5. If Y/PY to 2.2 and 2.3, or N/PN to 2.4: Were adjustment techniques used that are likely to correct for the presence of selection biases?		
**Bias in classification of interventions**		Notes
3.1 Were intervention groups clearly defined? Was the definition of adherence clearly defined?	Probably Yes	The groups were clearly defined, but participants in the control group had access to the website during the second 12 month period.
3.2 Was the information used to define intervention groups recorded at the start of the intervention?	Yes	criteria for the 12 month period and not based on their use of the app in the second twelve month
3.3 Could classification of intervention status have been affected by knowledge of the outcome or risk of the outcome?	No	
**Bias due to deviations from intended interventions**		Notes
**ROBINS-I**		
4.1. Were there deviations from the intended intervention beyond what would be expected in usual practice?	Yes	
4.2. If Y/PY to 4.1: Were these deviations from intended intervention unbalanced between groups and likely to have affected the outcome?	Yes	An uneven amount of people sought CBT in each group
4.3. Were important co-interventions balanced across intervention groups?	Yes	
4.4. Was the intervention implemented successfully for most participants?	No Information	
4.5. Did study participants adhere to the assigned intervention regimen?	No	31% of participants were not adherent to the assigned regimen during the first 12 months of the trial (unclear what percent of particpants with 24 month data this is)
4.6. If N/PN to 4.3, 4.4 or 4.5: Was an appropriate analysis used to estimate the effect of starting and adhering to the intervention?	No	Per-protocol analysis was used and the needed assumptions were not met. A few confounders were controlled for but it is likely that this sufficiently accounted for confounders.
Risk of bias: Assignment to intervention	Low / Moderate / Serious / **Critical** / NI	
Risk of bias: Starting and adhering to intervention	Low / Moderate / Serious / **Critical** / NI	
**Risk of bias judgement**	Low / Moderate / Serious / **Critical** / NI	
**RoB Tool (Risk of bias due to deviations from the intended interventions (effect of adhering to intervention))**		
2.1. Were participants aware of their assigned intervention during the trial?	Yes	
2.2. Were carers and people delivering the interventions aware of participants' assigned intervention during the trial?	Yes	
2.3. If Y/PY/NI to 2.1 or 2.2: Were important non protocol interventions balanced across intervention groups?	Probably No	This is not clear sincethe total number of participants excluded from the 24 month adherence analysis was not reported in Table 4
2.4. [If applicable:] Were there failures in implementing the intervention that could have affected the outcome?	No Information	

2.5. [If applicable:] Was there non-adherence to the assigned intervention regimen that could have affected participants' outcomes?	Yes	31% of participants were not adherent to the assigned regimen during the first 12 months of the trial (unclear what percent of particpants with 24 month data this is)
2.6. If N/PN/NI to 2.3, or Y/PY/NI to 2.4 or 2.5: Was an appropriate analysis used to estimate the effect of adhering to the intervention?	No	Per-protocol analysis was used and the needed assumptions were not met. A few confounders were controlled for but it is likely that this sufficiently accounted for confounders.
**Risk of bias judgement**	Low/**High**/Some Concerns	
**Bias due to missing data**		Notes
5.1 Were outcome data available for all, or nearly all, participants?	No	
5.2 Were participants excluded due to missing data on intervention status?	No	
5.3 Were participants excluded due to missing data on other variables needed for the analysis?	No	
5.4 If PN/N to 5.1, or Y/PY to 5.2 or 5.3: Are the proportion of participants and reasons for missing data similar across interventions?	Yes	Similar proportion of missing data between WCBT and TCBT groups
5.5 If PN/N to 5.1, or Y/PY to 5.2 or 5.3: Is there evidence that results were robust to the presence of missing data?	Yes	Multiple imputation was used to account for missing data
**Bias in measurement of outcomes**		Notes
6.1 Could the outcome measure have been influenced by knowledge of the intervention received?	Probably yes	Subjective outcome assessment without blinding of assessors
6.2 Were outcome assessors aware of the intervention received by study participants? (blinding of assessors)	Yes	
6.3 Were the methods of outcome assessment comparable across intervention groups?	Yes	
6.4 Were any systematic errors in measurement of the outcome related to intervention received?	No	
**Bias in selection of the reported result**		Notes
Were the data that produced this result analysed in accordance with a pre-specified analysis plan that was finalized before unblinded outcome data were available for analysis?	No	
Is the reported effect estimate likely to be selected, on the basis of the results, from...		
7.1. ... multiple outcome measurements within the outcome domain?	Probably No	Same measurements as the 12 month preregistered analysis
7.2 ... multiple analyses of the intervention-outcome relationship?	Probably No	Same analysis method as the 12 month preregistered analysis
7.3 ... different subgroups?	No	No subgroups were analyzed
**Secondary Analysis RoB Assessment**		Notes
**Is the seconday analysis evaluating effectiveness or efficacy?**	Efficacy	
**Comparator**	WCBT vs TAU	
**Outcome Being Assessed**	Per-protocol analysis of 24 month IBS-SSS and WSAS	
**Specify the Numerical Result Being Assessed**	IBS-SSS at 24 months was 51·5 points (23·9–79·0; p<0·001) lower in the web-CBT group than in the TAU group.<br><br>Mean WSAS at 24 months was 3·7 points (1·7–5·7; p<0·001) lower in the web-CBT group than in the TAU group	
**Bias due to randomization (See primary analysis section)**		Notes
**Bias due to confounding**		Notes
1.1 Is there potential for confounding of the effect of intervention in this study?	Yes	
1.2. If Y/PY to 1.1 Was the analysis based on splitting participants' follow up time according to intervention received?	No	
1.3. If Y/PY to 1.2 Were intervention discontinuations or switches likely to be related to factors that are prognostic for the outcome?		
**Questions relating to baseline confounding only**		
1.4. If Y/PY to 1.1 Did the authors use an appropriate analysis method that controlled for all the important confounding domains?	No	They controlled for confounders of missingness but not for confounders of non-adherence.<br>I would imagine that the excluded participants in the web group would be very different than the excluded participants in the control group (non-motivated people in treatment, motivated people in control)
1.5. If Y/PY to 1.4: Were confounding domains that were controlled for measured validly and reliably by the variables available in this study?	Yes	
1.6. Did the authors control for any post-intervention variables that could have been affected by the intervention?	No	
**Questions relating to baseline and time-varying confounding**		
1.7. If Y/PY to 1.3 Did the authors use an appropriate analysis method that controlled for all the important confounding domains and for time-varying confounding?		
1.8. If Y/PY to 1.7: Were confounding domains that were controlled for measured validly and reliably by the variables available in this study?		
**Bias in selection of participants into the study (Not evaluating for analyses of effectiveness)**		Notes
2.1. Was selection of participants into the analysis based on participant characteristics observed after the start of intervention?	Yes	
2.2. If Y/PY to 2.1: Were the post-intervention variables that influenced selection likely to be associated with intervention?	Yes	Particpants who were excluded in the control group due to use of the web app which they were told they could use during months 12-14, participants excluded in the web group may not have liked the intervention
2.3 If Y/PY to 2.2: Were the post-intervention variables that influenced selection likely to be influenced by the outcome or a cause of the outcome?	Yes	Participant factors inclucencing adherence were also likely to influence the outcome as well
2.4. Do start of follow-up and start of intervention coincide for most participants?	Yes	
2.5. If Y/PY to 2.2 and 2.3, or N/PN to 2.4: Were adjustment techniques used that are likely to correct for the presence of selection biases?	No	Confounders of missingness were controlled for. IV methods such as CACE/LATE could have been used instead.
**Bias in classification of interventions**		Notes
3.1 Were intervention groups clearly defined?	Probably Yes	Adherence during the first 12 months was clearly defined. Adherence due to CBT use was not preregistered
3.2 Was the information used to define intervention groups recorded at the start of the intervention?	Probably No	Adherence to the interventions were defined but the exclusion criteria for CBT use was not predefined
3.3 Could classification of intervention status have been affected by knowledge of the outcome or risk of the outcome?	Probably Yes	There were multiple ways that they defined CBT use and it is possible that they chose the definition based on outcome results
**Bias due to deviations from intended interventions (See primary analysis section)**		Notes
**Bias due to missing data**		Notes
5.1 Were outcome data available for all, or nearly all, participants?	No	Data only available for half of all participants. It is not possible to calculate how many participants were excluded due to their adherence.
5.2 Were participants excluded due to missing data on intervention status?	No	
5.3 Were participants excluded due to missing data on other variables needed for the analysis?	No	
5.4 If PN/N to 5.1, or Y/PY to 5.2 or 5.3: Are the proportion of participants and reasons for missing data similar across interventions?	No Information	Would need to know the number of participants excluded from the WCBT based on adherence in the first 12 month period

5.5 If PN/N to 5.1, or Y/PY to 5.2 or 5.3: Is there evidence that results were robust to the presence of missing data?	No	Predictors of missingness were used in the regression but not predictors of non-adherence
**Bias in measurement of outcomes**		Notes
6.1 Could the outcome measure have been influenced by knowledge of the intervention received?	Probably yes	Subjective outcome assessment without blinding of assessors
6.2 Were outcome assessors aware of the intervention received by study participants? (blinding of assessors)	Yes	
6.3 Were the methods of outcome assessment comparable across intervention groups?	Yes	
6.4 Were any systematic errors in measurement of the outcome related to intervention received?	No	
6.5 Could adherence have been influenced by study participation?	Probably Yes	Adherence during the first 12 month period could have been influenced by study participation but adherence during the second 12 month period could not have been influenced by study participation
**Bias in selection of the reported result**		Notes
Were the data that produced this result analysed in accordance with a pre-specified analysis plan that was finalized before unblinded outcome data were available for analysis?	No	
Is the reported effect estimate likely to be selected, on the basis of the results, from...		
7.1. ... multiple outcome measurements within the outcome domain?	Probably No	Same measurements as the 12 month preregistered analysis
7.2 ... multiple analyses of the intervention-outcome relationship?	Yes	They could have attempted to account for different confounders and found different results or attempted a CACE/LATE analysis and deferred to the per-protocol analysis based on the positive findings
7.3 ... different subgroups?	Yes	They could have used different definitions of non-adherence based on their definitions of CBT therapy

Criteria for Adherence Metrics and Analysis		Notes
**Device name and indication for use**	EndeavorRx (attention control for ADHD populations)	
**Type of device** (Long-term vs short-term use)	Short-term use	
**Type of device** (Presence or absence of a prescribed dosage)	Presence of a prescribed dosage	
**Was adherence information collected?**	Yes	
**What information was collected about usage adherence?**	Information about completion of missions	Prescribed for use 5 times a day for 5 days per week
**What information was collected about adherence to recommendations?**	NA	No explicit app recommendations
**Was information collected internally in the app?**	Yes	
**Was information modified internally?**	No	Participants were contacted after two days of inactivity
**Was initiation reported?**	Yes	Lowest completion rate was 61%
**What was average adherence?** (if reported)	100%	
**Was implementation reported?**	Yes	
**What was average adherence?** (if reported)	100% +/- 20% (range=61-136%)	
**Was persistence reported?**	No	They report that 2 participants discontinued training but do not report when or how many missions they completed
**What was average adherence?** (if reported)		
**Was adherence low?**	No	
**Was adherence not reported or low?**	No	
**Was efficacy analyzed?**	Yes	
**What method was used?**	Average treatment effect analysis	
**Were efficacy analyses preregistered?**	Yes	
**What assumptions are required for that method to study efficacy?**	SUTVA, positivity, consistency, ignorability	
**Did the article report evidence that the assumptions were met?**	No	
**SUTVA**	Yes	Treatment was only administered within the app
**Positivity**	No	There was not a control condition
**Consistency** (treatment definition)	Yes	Clear definition of treatment
**Consistency** (adherence definition)		
**Exclusion restriction**		
**Strong Monotonicity**		
**Ignorability**	basic response time	
**Overall Notes**		

Cochrane CDPLG		Notes
**Data form completed date** (dd/mm/yyyy)	3/23/2022	
**Study author contact details**	joaquin.anguera@ucsf.edu (JAA); emarco@corticacare.com (EJM)	
Methods	**Descriptions as stated in report/paper**	**Location in text or source** (pg & ¶/fig/table/other)
Aim of study (e.g. efficacy, equivalence, pragmatic)	Efficacy<br>Change in a neural marker for an adherent population	pg. 2 Introduction<br>pg. 5 AKL-T01 Intervention
Design (e.g. parallel, crossover, non-RCT)	Prospective observational trial	pg. 3 Participants and Design
Unit of allocation (by individuals, cluster/ groups or body parts)	Individual	
Participants	**Descriptions as stated in report/paper**	**Location in text or source** (pg & ¶/fig/table/other)
**Inclusion criteria**	"a confirmed ADHD diagnosis (based on DSM-5 criteria), between March 2019 to February 2020. Characterization of clinical ADHD symptoms in children often relies upon parent report questionnaires for ease of administration [22–24]. As such, ADHD inclusion criteria for this study was based on the Vanderbilt Parent-Report measure as a screening tool [19]. Following the Vanderbilt criteria for inattention symptoms in ADHD (inattention subscale), parents had to report a symptom frequency of 'often' or 'very often' (2 or 3 on a scale from 0-'never' to 3-'very often') on at least six of nine items relating to inattention. In addition, we confirmed this ADHD diagnosis with the Mini-International Neuropsychiatric Interview for Children and Adolescents (MINI Kid, Section N [25]), administered by a trained clinician."	pg. 3 Participants and Design
**Exclusion criteria**	"Exclusion criteria were brain malformation or injury, movement disorders, psychiatric con ditions (e.g., bipolar, psychotic, and autism spectrum disorders), and hearing impairment. In addition, children had to be off antipsychotic or ADHD medications (stimulants, alpha-adren ergic medications, or atomoxetine) for the duration of the study and a washout period of at least 30 days prior to initiation. Further, children had to score >= 70 on the Perceptual Reason ing Index (PRI) on the Wechsler Intelligence Scale for Children–Fifth Edition and had to be able to comply with all testing and requirements."	pg. 3 Participants and Design
**Total no. randomised** (or total pop. at start of study for NRCTs)	28	pg. 3 Participants and Design
**Clusters** (if applicable, no., type, no. people per cluster)	NA	
**Withdrawals and exclusions** (if not provided below by outcome)	3 participants withdrew; Data from 3 participants could not be used	pg. 7 Figure 1
Intervention Groups	**Descriptions as stated in report/paper**	**Location in text or source** (pg & ¶/fig/table/other)
**Group name**	AKL-T01<br>Video game like intervention that targets multi-tasking	pg. 5 AKL-T01 Intervention
**No. randomised to group** (specify whether no. people or clusters)	28	pg. 3 Participants and Design
**Timing** (e.g. frequency, duration of each episode)	Participants had to complete 5 misions a day for 5 days a week over 4 weeks	pg. 5 AKL-T01 Intervention
**Co-interventions**	None	
**Integrity of delivery**	The minimum mission completion rate for participants who did not withdraw was 61%	pg 6. Participant Demographics
**Compliance**	Adherence was very high with average adherence of 100%	pg. 6 Participant Demographics
Outcomes	**Descriptions as stated in report/paper**	**Location in text or source** (pg & ¶/fig/table/other)
**Outcome name**	Post-stimulus ERSP change in peak MFT	pg. 4 ERSP Analysis
**Outcome definition** (with diagnostic criteria if relevant)(include name, time, and analysis method)	Repeated measures ANOVA after 4 weeks of treatment	pg. 5 Statistical Analysis
**Imputation of missing data** (e.g. assumptions made for ITT analysis)	Two pariticpants were excluded bassed on having excessive noise in EEG data (No imputation)	pg 6. Participant Demographics
**Power** (e.g. power & sample size calculation, level of power achieved)	No information about power	
Outcomes	**Descriptions as stated in report/paper**	**Location in text or source** (pg & ¶/fig/table/other)
**Outcome name**	Changes in 3 120 ms composit bins around the MFT peak	pg. 4 ERSP Analysis
**Outcome definition** (with diagnostic criteria if relevant)(include name, time, and analysis method)	Repeated measures ANOVA after 4 weeks of treatment	pg. 5 Statistical Analysis
**Imputation of missing data** (e.g. assumptions made for ITT analysis)	Two pariticpants were excluded bassed on having excessive noise in EEG data (No imputation)	pg 6. Participant Demographics
**Power** (e.g. power & sample size calculation, level of power achieved)	No information about power	

**Risk of Bias** (Based on Cochrane RoB Tool 2 and ROBINS-I)		Link to RoB Algorithm
**Primary Analysis RoB Assessment**		Notes
**Is the primary analysis evaluating effectiveness or efficacy?**	Efficacy	
**Comparator**	No comparison group	
**Outcome Being Assessed**	Main effect of session on MFT after 4 weeks of treatment	The study states that there was a main effect of session which refers to the pre or post intervention session
**Specify the Numerical Result Being Assessed**	"We observed a main effect of session, (F(1,21) = 4.76, p = 0.04, d = 0.44), suggesting that there was a general increase in MFT following the intervention"	
**Bias due to randomization (RCT only)**		Notes
1.1 Was the allocation sequence random?	NA	
1.2. Was the allocation sequence concealed until participants were enrolled and assigned to interventions?	NA	
1.3. Did baseline differences between intervention groups suggest a problem with the randomization process?	NA	

Article Key

**Bias due to confounding**		Notes
1.1 Is there potential for confounding of the effect of intervention in this study?	Yes	Many confounders could affect the result given the single group design
1.2. If Y/PY to 1.1 Was the analysis based on splitting participants' follow up time according to intervention received?	No	
1.3. If Y/PY to 1.2 Were intervention discontinuations or switches likely to be related to factors that are prognostic for the outcome?		
**Questions relating to baseline confounding only**		
1.4. If Y/PY to 1.1 Did the authors use an appropriate analysis method that controlled for all the important confounding domains?	No	They did consider basic response time as a potential confound
1.5. If Y/PY to 1.4: Were confounding domains that were controlled for measured validly and reliably by the variables available in this study?		
1.6. Did the authors control for any post-intervention variables that could have been affected by the intervention?	No	
**Questions relating to baseline and time-varying confounding**		
1.7. If Y/PY to 1.3 Did the authors use an appropriate analysis method that controlled for all the important confounding domains and for time-varying confounding?		
1.8. If Y/PY to 1.7: Were confounding domains that were controlled for measured validly and reliably by the variables available in this study?		
**Bias in selection of participants into the study (Not evaluating for analyses of effectiveness)**		Notes
2.1. Was selection of participants into the analysis based on participant characteristics observed after the start of intervention?		
2.2. If Y/PY to 2.1: Were the post-intervention variables that influenced selection likely to be associated with intervention?		
2.3 If Y/PY to 2.2: Were the post-intervention variables that influenced selection likely to be influenced by the outcome or a cause of the outcome?		
2.4. Do start of follow-up and start of intervention coincide for most participants?		
2.5. If Y/PY to 2.2 and 2.3, or N/PN to 2.4: Were adjustment techniques used that are likely to correct for the presence of selection biases?		
**Bias in classification of interventions**		Notes
3.1 Were intervention groups clearly defined? Was the definition of adherence clearly defined?	Yes	
3.2 Was the information used to define intervention groups recorded at the start of the intervention?	Yes	Intervention clearly defined in the preregistration
3.3 Could classification of intervention status have been affected by knowledge of the outcome or risk of the outcome?	No	
**Bias due to deviations from intended interventions**		Notes
**ROBINS-I**		
4.1. Were there deviations from the intended intervention beyond what would be expected in usual practice?	Yes	in the case of inactivity which would not happen with usual care
4.2. If Y/PY to 4.1: Were these deviations from intended intervention unbalanced between groups and likely to have affected the outcome?	NA	Only one group
4.3. Were important co-interventions balanced across intervention groups?	NA	
4.4. Was the intervention implemented successfully for most participants?	Yes	
4.5. Did study participants adhere to the assigned intervention regimen?	Yes	Adherence was high due to the help of study coordinators
4.6. If N/PN to 4.3, 4.4 or 4.5: Was an appropriate analysis used to estimate the effect of starting and adhering to the intervention?		
Risk of bias: Assignment to intervention	**Low** / Moderate / Serious / Critical / NI	
Risk of bias: Starting and adhering to intervention	**Low** / Moderate / Serious / Critical / NI	
**Risk of bias judgement**	**Low** / Moderate / Serious / Critical / NI	
**RoB Tool (Risk of bias due to deviations from the intended interventions (effect of adhering to intervention))**		
2.1. Were participants aware of their assigned intervention during the trial?	Yes	
2.2. Were carers and people delivering the interventions aware of participants' assigned intervention during the trial?	Yes	
2.3. If Y/PY/NI to 2.1 or 2.2: Were important non protocol interventions balanced across intervention groups?	NA	
2.4. [If applicable:] Were there failures in implementing the intervention that could have affected the outcome?	No	
2.5. [If applicable:] Was there non-adherence to the assigned intervention regimen that could have affected participants' outcomes?	No	
2.6. If N/PN/NI to 2.3, or Y/PY/NI to 2.4 or 2.5: Was an appropriate analysis used to estimate the effect of adhering to the intervention?		
**Risk of bias judgement**	**Low**/High/Some Concerns	
**Bias due to missing data**		Notes
5.1 Were outcome data available for all, or nearly all, participants?	Probably No	Data available for 79% of participants
5.2 Were participants excluded due to missing data on intervention status?	No	
5.3 Were participants excluded due to missing data on other variables needed for the analysis?	No	
5.4 If PN/N to 5.1, or Y/PY to 5.2 or 5.3: Are the proportion of participants and reasons for missing data similar across interventions?	NA	
5.5 If PN/N to 5.1, or Y/PY to 5.2 or 5.3: Is there evidence that results were robust to the presence of missing data?	No	No attempt to account for missing data (probably would've needed a larger sample size for that)
**Bias in measurement of outcomes**		Notes
6.1 Could the outcome measure have been influenced by knowledge of the intervention received?	No	
6.2 Were outcome assessors aware of the intervention received by study participants? (blinding of assessors)	Yes	
6.3 Were the methods of outcome assessment comparable across intervention groups?	NA	
6.4 Were any systematic errors in measurement of the outcome related to intervention received?	No	
**Bias in selection of the reported result**		Notes
Were the data that produced this result analysed in accordance with a pre-specified analysis plan that was finalized before unblinded outcome data were available for analysis?	Probably Yes	Analysis was preregistered but was not very specific
Is the reported effect estimate likely to be selected, on the basis of the results, from...		
7.1. ... multiple outcome measurements within the outcome domain?	Yes	They give multiple definitions of the primary outcome in the preregistration
7.2 ... multiple analyses of the intervention-outcome relationship?	Yes	They don't specify in the preregistration how they will analyze the data
7.3 ... different subgroups?	No	
**Secondary Analysis RoB Assessment**		Notes
**Is the seconday analysis evaluating effectiveness or efficacy?**		
**Comparator**		
**Outcome Being Assessed**		
**Specify the Numerical Result Being Assessed**		
**Bias due to randomization (See primary analysis section)**		Notes
**Bias due to confounding**		Notes
1.1 Is there potential for confounding of the effect of intervention in this study?		
1.2. If Y/PY to 1.1 Was the analysis based on splitting participants' follow up time according to intervention received?		
1.3. If Y/PY to 1.2 Were intervention discontinuations or switches likely to be related to factors that are prognostic for the outcome?		
**Questions relating to baseline confounding only**		

1.4. If Y/PY to 1.1 Did the authors use an appropriate analysis method that controlled for all the important confounding domains?		
1.5. If Y/PY to 1.4: Were confounding domains that were controlled for measured validly and reliably by the variables available in this study?		
1.6. Did the authors control for any post-intervention variables that could have been affected by the intervention?		
**Questions relating to baseline and time-varying confounding**		
1.7. If Y/PY to 1.3 Did the authors use an appropriate analysis method that controlled for all the important confounding domains and for time-varying confounding?		
1.8. If Y/PY to 1.7: Were confounding domains that were controlled for measured validly and reliably by the variables available in this study?		
**Bias in selection of participants into the study (Not evaluating for analyses of effectiveness)**		Notes
2.1. Was selection of participants into the analysis based on participant characteristics observed after the start of intervention?		
2.2. If Y/PY to 2.1: Were the post-intervention variables that influenced selection likely to be associated with intervention?		
2.3 If Y/PY to 2.2: Were the post-intervention variables that influenced selection likely to be influenced by the outcome or a cause of the outcome?		
2.4. Do start of follow-up and start of intervention coincide for most participants?		
2.5. If Y/PY to 2.2 and 2.3, or N/PN to 2.4: Were adjustment techniques used that are likely to correct for the presence of selection biases?		
**Bias in classification of interventions**		Notes
3.1 Were intervention groups clearly defined?		
3.2 Was the information used to define intervention groups recorded at the start of the intervention?		
3.3 Could classification of intervention status have been affected by knowledge of the outcome or risk of the outcome?		
**Bias due to deviations from intended interventions (See primary analysis section)**		Notes
**Bias due to missing data**		Notes
5.1 Were outcome data available for all, or nearly all, participants?		
5.2 Were participants excluded due to missing data on intervention status?		
5.3 Were participants excluded due to missing data on other variables needed for the analysis?		
5.4 If PN/N to 5.1, or Y/PY to 5.2 or 5.3: Are the proportion of participants and reasons for missing data similar across interventions?		
5.5 If PN/N to 5.1, or Y/PY to 5.2 or 5.3: Is there evidence that results were robust to the presence of missing data?		
**Bias in measurement of outcomes**		Notes
6.1 Could the outcome measure have been influenced by knowledge of the intervention received?		
6.2 Were outcome assessors aware of the intervention received by study participants? (blinding of assessors)		
6.3 Were the methods of outcome assessment comparable across intervention groups?		
6.4 Were any systematic errors in measurement of the outcome related to intervention received?		
6.5 Could adherence have been influenced by study participation?		
**Bias in selection of the reported result**		Notes
Were the data that produced this result analysed in accordance with a pre-specified analysis plan that was finalized before unblinded outcome data were available for analysis?		
Is the reported effect estimate likely to be selected, on the basis of the results, from...		
7.1. ... multiple outcome measurements within the outcome domain?		
7.2 ... multiple analyses of the intervention-outcome relationship?		
7.3 ... different subgroups?		

Criteria for Adherence Metrics and Analysis		Notes
**Device name and indication for use**	Clue (contraception)	
**Type of device** (Long-term vs short-term use)	Long-term use	
**Type of device** (Presence or absence of a prescribed dosage)	Presence of a prescribed dose	
**Was adherence information collected?**	Yes	
**What information was collected about usage adherence?**	Collects information about daily coital information and alternative contraceptive use was collected in a separate app called proofmode that fits over the intended app<br>App login information	
**What information was collected about adherence to recommendations?**	Correct and incorrect use cycles where correct means using backup protection	
**Was information collected internally in the app?**	No	
**Was information modified internally?**	NA	
**Was initiation reported?**	Yes	Inclusion criteria required users to enter two period start dates
**What was average adherence?** (if reported)	100%	
**Was implementation reported?**	No	They report the number of participants who enter their sexual history for a cycle but not the number who reported correct or incorrect cycles
**What was average adherence?** (if reported)		
**Was persistence reported?**	Yes	
**What was average adherence?** (if reported)	195/629 (31%) women exited for some reason over the six cycles of use (69%) retained)	
**Was adherence low?**	NA	
**Was adherence not reported or low and could have affected outcomes?**	No	Implementation was not reported but it was reported that all pregnancies were with imperfect use
**Was efficacy analyzed?**	No	All pregnancies were with imperfect use
**What method was used?**	NA	
**Were efficacy analyses preregistered?**		
**What assumptions are required for that method to study efficacy?**		
**Did the article report evidence that the assumptions were met?**		
**SUTVA**		
**Positivity**		
**Consistency** (treatment definition)		
**Consistency** (adherence definition)		
**Exclusion restriction**		
**Strong Monotonicity**		
**Ignorability**		
**Overall Notes**		
**Cochrane CDPLG**		**Notes**
**Data form completed date** (dd/mm/yyyy)	4/5/2022	
**Study author contact details**	Victoria.jennings@georgetown.edu	
Methods	**Descriptions as stated in report/paper**	**Location in text or source** (pg & ¶/fig/table/other)
Aim of study (e.g. efficacy, equivalence, pragmatic)	Effectiveness<br>6 cycle effectiveness (defined as efficacy but meets our criteria for effectiveness)	pg. 2 Materials and Methods
Design (e.g. parallel, crossover, non-RCT)	Prospective observational cohort study	pg. 1 Introduction
Unit of allocation (by individuals, cluster/ groups or body parts)	Individual	
Participants	**Descriptions as stated in report/paper**	**Location in text or source** (pg & ¶/fig/table/other)
**Inclusion criteria**	"between ages 18 and 39, confirming that they have cycles between 20 and 40 days with less than 10 days of variation, being sexually active with a male partner (or partners), not having used hormonal contraception in the last 3 months and having had at least three menstrual periods following the most recent (if any) pregnancy"<br><br>Participants also had to enter two period start dates	pg. 2 Materials and Methods
**Exclusion criteria**	Excluded women with too long or too variable of cycles, women with phone-related issues, and women who switched their pregnancy intentions	pg. 2 Materials and Methods<br>pg. 4 Appendix A
**Total no. randomised** (or total pop. at start of study for NRCTs)	718 (629 age 18-35)	pg. 2 Results
**Clusters** (if applicable, no., type, no. people per cluster)	NA	
**Withdrawals and exclusions** (if not provided below by outcome)	Excluded women over the age of 35	pg. 2 Materials and Methods
Intervention Groups	**Descriptions as stated in report/paper**	**Location in text or source** (pg & ¶/fig/table/other)
**Group name**	Dynamic Optimal Timing (DOT)	pg. 1 Introduction
**No. randomised to group** (specify whether no. people or clusters)	718 (629 age 18-35)	pg. 2 Results
**Timing** (e.g. frequency, duration of each episode)	Users needed to enter their period start date each month and use condoms or abstain from sex when they were in the fertile window<br>For the trial women needed to input daily coital information	pg. 2 Materials and Methods
**Co-interventions**	Condom use during the fertile window	pg. 2 Materials and Methods
**Integrity of delivery**	All women needed to input two period start dates to be included in the trial	pg. 2 Materials and Methods
**Compliance**	No information	
Outcomes	**Descriptions as stated in report/paper**	**Location in text or source** (pg & ¶/fig/table/other)
**Outcome name**	6 Cycle Effectiveness	pg. 2 Materials and Methods
**Outcome definition** (with diagnostic criteria if relevant)(include name, time, and analysis method)	Typical use pregancy rate after 6 cycles using kaplan myer life table assessments	pg. 2 Materials and Methods
**Imputation of missing data** (e.g. assumptions made for ITT analysis)	"For this sensitivity analysis, we classified women who discontinued after the onset of the fertile window and reported unprotected intercourse one or more times during the fertile window as "possibly pregnant." We classified women who discontinued prior to the onset of the fertile window and did not report unprotected intercourse during that fertile window as "unlikely to be pregnant." "	pg. 2 Materials and Methods
**Power** (e.g. power & sample size calculation, level of power achieved)	"for pregnancy rate at 13 cycles they needed 255 women to detect a 6% drop in the pregnancy rate at 90% power"<br>Based on their protocol, the assumed relative risk was 3 but do not reference that in this manuscript.	pg. 2 Materials and Methods
**Risk of Bias** (Based on Cochrane RoB Tool 2 and ROBINS-I)		Link to RoB Algorithm
**Primary Analysis RoB Assessment**		Notes
**Is the primary analysis evaluating effectiveness or efficacy?**	Effectiveness	
**Comparator**	No comparison group	
**Outcome Being Assessed**	6 Cycle Effectiveness	
**Specify the Numerical Result Being Assessed**	6-month typical-use failure rate of 3.5% [95% CI 1.7-5.2]	
**Bias due to randomization (RCT only)**		Notes
1.1 Was the allocation sequence random?	NA	
1.2. Was the allocation sequence concealed until participants were enrolled and assigned to interventions?	NA	
1.3. Did baseline differences between intervention groups suggest a problem with the randomization process?	NA	
**Bias due to confounding**		Notes
1.1 Is there potential for confounding of the effect of intervention in this study?	Yes	
1.2. If Y/PY to 1.1 Was the analysis based on splitting participants' follow up time according to intervention received?	No	
1.3. If Y/PY to 1.2 Were intervention discontinuations or switches likely to be related to factors that are prognostic for the outcome?		
**Questions relating to baseline confounding only**		
1.4. If Y/PY to 1.1 Did the authors use an appropriate analysis method that controlled for all the important confounding domains?	No	They could have done subcohort analysis to investigate the effects of confounders
1.5. If Y/PY to 1.4: Were confounding domains that were controlled for measured validly and reliably by the variables available in this study?		
1.6. Did the authors control for any post-intervention variables that could have been affected by the intervention?		

Article Key

**Questions relating to baseline and time-varying confounding**		
1.7. If Y/PY to 1.3 Did the authors use an appropriate analysis method that controlled for all the important confounding domains and for time-varying confounding?		
1.8. If Y/PY to 1.7: Were confounding domains that were controlled for measured validly and reliably by the variables available in this study?		
**Bias in selection of participants into the study (Not evaluating for analyses of effectiveness)**		Notes
2.1. Was selection of participants into the analysis based on participant characteristics observed after the start of intervention?		
2.2. If Y/PY to 2.1: Were the post-intervention variables that influenced selection likely to be associated with intervention?		
2.3 If Y/PY to 2.2: Were the post-intervention variables that influenced selection likely to be influenced by the outcome or a cause of the outcome?		
2.4. Do start of follow-up and start of intervention coincide for most participants?		
2.5. If Y/PY to 2.2 and 2.3, or N/PN to 2.4: Were adjustment techniques used that are likely to correct for the presence of selection biases?		
**Bias in classification of interventions**		Notes
3.1 Were intervention groups clearly defined? Was the definition of adherence clearly defined?	Yes	
3.2 Was the information used to define intervention groups recorded at the start of the intervention?	Yes	The intervention is clearly defined in the published protocol
3.3 Could classification of intervention status have been affected by knowledge of the outcome or risk of the outcome?	No	
**Bias due to deviations from intended interventions**		Notes
**ROBINS-I**		
4.1. Were there deviations from the intended intervention beyond what would be expected in usual practice?	No	
4.2. If Y/PY to 4.1: Were these deviations from intended intervention unbalanced between groups and likely to have affected the outcome?	NA	
4.3. Were important co-interventions balanced across intervention groups?	NA	
4.4. Was the intervention implemented successfully for most participants?	Yes	All included participants needed to initiate use of the app
4.5. Did study participants adhere to the assigned intervention regimen?	No information	The 15 women that became pregnant did not adhere to the prescribed instructions
4.6. If N/PN to 4.3, 4.4 or 4.5: Was an appropriate analysis used to estimate the effect of starting and adhering to the intervention?	NA	There were no pregnancies recorded that were for perfect use
Risk of bias: Assignment to intervention	**Low** / Moderate / Serious / Critical / NI	
Risk of bias: Starting and adhering to intervention	**Low** / Moderate / Serious / Critical / NI	
**Risk of bias judgement**	**Low** / Moderate / Serious / Critical / NI	
**RoB Tool (Risk of bias due to deviations from the intended interventions (effect of adhering to intervention))**		
2.1. Were participants aware of their assigned intervention during the trial?	Yes	
2.2. Were carers and people delivering the interventions aware of participants' assigned intervention during the trial?	NA	
2.3. If Y/PY/NI to 2.1 or 2.2: Were important non protocol interventions balanced across intervention groups?	NA	
2.4. [If applicable:] Were there failures in implementing the intervention that could have affected the outcome?	Probably Yes	The women who got pregnant did not adheren to the prescribed regimen
2.5. [If applicable:] Was there non-adherence to the assigned intervention regimen that could have affected participants' outcomes?	Probably No	There were no pregnancies recorded that were for perfect use
2.6. If N/PN/NI to 2.3, or Y/PY/NI to 2.4 or 2.5: Was an appropriate analysis used to estimate the effect of adhering to the intervention?	NA	
**Risk of bias judgement**	**Low**/High/Some Concerns	
**Bias due to missing data**		Notes
5.1 Were outcome data available for all, or nearly all, participants?	Yes	6.7% of women were lost to follow-up
5.2 Were participants excluded due to missing data on intervention status?	No	
5.3 Were participants excluded due to missing data on other variables needed for the analysis?	No	
5.4 If PN/N to 5.1, or Y/PY to 5.2 or 5.3: Are the proportion of participants and reasons for missing data similar across interventions?		
5.5 If PN/N to 5.1, or Y/PY to 5.2 or 5.3: Is there evidence that results were robust to the presence of missing data?		
**Bias in measurement of outcomes**		Notes
6.1 Could the outcome measure have been influenced by knowledge of the intervention received?	Probably No	
6.2 Were outcome assessors aware of the intervention received by study participants? (blinding of assessors)	Yes	
6.3 Were the methods of outcome assessment comparable across intervention groups?	Yes	
6.4 Were any systematic errors in measurement of the outcome related to intervention received?	No	
**Bias in selection of the reported result**		Notes
Were the data that produced this result analysed in accordance with a pre-specified analysis plan that was finalized before unblinded outcome data were available for analysis?	Yes	Data was collected based on a preregistered protocol for a 13 month study
Is the reported effect estimate likely to be selected, on the basis of the results, from...		
7.1. ... multiple outcome measurements within the outcome domain?	No	
7.2 ... multiple analyses of the intervention-outcome relationship?	Probably No	Only life table analysis reported and not the pearl index, but life tables provide a more conservative estimate
7.3 ... different subgroups?	No	No subgroups analyzed
**Secondary Analysis RoB Assessment**		Notes
**Is the seconday analysis evaluating effectiveness or efficacy?**		
**Comparator**		
**Outcome Being Assessed**		
**Specify the Numerical Result Being Assessed**		
**Bias due to randomization (See primary analysis section)**		Notes
**Bias due to confounding**		Notes
1.1 Is there potential for confounding of the effect of intervention in this study?		
1.2. If Y/PY to 1.1 Was the analysis based on splitting participants' follow up time according to intervention received?		
1.3. If Y/PY to 1.2 Were intervention discontinuations or switches likely to be related to factors that are prognostic for the outcome?		
**Questions relating to baseline confounding only**		
1.4. If Y/PY to 1.1 Did the authors use an appropriate analysis method that controlled for all the important confounding domains?		
1.5. If Y/PY to 1.4: Were confounding domains that were controlled for measured validly and reliably by the variables available in this study?		
1.6. Did the authors control for any post-intervention variables that could have been affected by the intervention?		
**Questions relating to baseline and time-varying confounding**		
1.7. If Y/PY to 1.3 Did the authors use an appropriate analysis method that controlled for all the important confounding domains and for time-varying confounding?		
1.8. If Y/PY to 1.7: Were confounding domains that were controlled for measured validly and reliably by the variables available in this study?		
**Bias in selection of participants into the study (Not evaluating for analyses of effectiveness)**		Notes

2.1. Was selection of participants into the analysis based on participant characteristics observed after the start of intervention?		
2.2. If Y/PY to 2.1: Were the post-intervention variables that influenced selection likely to be associated with intervention?		
2.3 If Y/PY to 2.2: Were the post-intervention variables that influenced selection likely to be influenced by the outcome or a cause of the outcome?		
2.4. Do start of follow-up and start of intervention coincide for most participants?		
2.5. If Y/PY to 2.2 and 2.3, or N/PN to 2.4: Were adjustment techniques used that are likely to correct for the presence of selection biases?		
**Bias in classification of interventions**		Notes
3.1 Were intervention groups clearly defined?		
3.2 Was the information used to define intervention groups recorded at the start of the intervention?		
3.3 Could classification of intervention status have been affected by knowledge of the outcome or risk of the outcome?		
**Bias due to deviations from intended interventions (See primary analysis section)**		Notes
**Bias due to missing data**		Notes
5.1 Were outcome data available for all, or nearly all, participants?		
5.2 Were participants excluded due to missing data on intervention status?		
5.3 Were participants excluded due to missing data on other variables needed for the analysis?		
5.4 If PN/N to 5.1, or Y/PY to 5.2 or 5.3: Are the proportion of participants and reasons for missing data similar across interventions?		
5.5 If PN/N to 5.1, or Y/PY to 5.2 or 5.3: Is there evidence that results were robust to the presence of missing data?		
**Bias in measurement of outcomes**		Notes
6.1 Could the outcome measure have been influenced by knowledge of the intervention received?		
6.2 Were outcome assessors aware of the intervention received by study participants? (blinding of assessors)		
6.3 Were the methods of outcome assessment comparable across intervention groups?		
6.4 Were any systematic errors in measurement of the outcome related to intervention received?		
6.5 Could adherence have been influenced by study participation?		
**Bias in selection of the reported result**		Notes
Were the data that produced this result analysed in accordance with a pre-specified analysis plan that was finalized before unblinded outcome data were available for analysis?		
Is the reported effect estimate likely to be selected, on the basis of the results, from...		
7.1. ... multiple outcome measurements within the outcome domain?		
7.2 ... multiple analyses of the intervention-outcome relationship?		
7.3 ... different subgroups?		

Criteria for Adherence Metrics and Analysis		Notes
**Device name and indication for use**	Clue (contraception)	
**Type of device** (Long-term vs short-term use)	Long-term use	
**Type of device** (Presence or absence of a prescribed dosage)	Presence of a prescribed dose	
**Was adherence information collected?**	Yes	Input
**What information was collected about usage adherence?**	Input of first cycle date<br>Proofmode and Amplitude software and interviews to categorize cycles into perfect and imperfect use	
**What information was collected about adherence to recommendations?**	Information about menstruation logs and sexual activity were collected	
**Was information collected internally in the app?**	No	
**Was information modified internally?**	NA	
**Was initiation reported?**	Yes	All included participants needed to download the app
**What was average adherence?** (if reported)	100%	
**Was implementation reported?**	Yes	
**What was average adherence?** (if reported)	24% of cycles were considered perfect use cycles	
**Was persistence reported?**	Yes	
**What was average adherence?** (if reported)	47% of users used the app for 13 cycles	
**Was adherence low?**	Yes	Less than 80% of cycles were perfect use
**Was adherence not reported or low and could have affected outcomes?**	Yes	
**Was efficacy analyzed?**	Yes	Perfect-use effectiveness calculated
**What method was used?**	Per-protocol analysis	Kaplan-Meier lifetables looking only at perfect-use cycles
**Were efficacy analyses preregistered?**	Yes	
**What assumptions are required for that method to study efficacy?**	SUTVA, positivity, consistency, ignorability, conditional independence of adherence and outcomes	
**Did the article report evidence that the assumptions were met?**	No	
**SUTVA**	Yes	Treatment only delivered through the app
**Positivity**	No	No control condition
**Consistency** (treatment definition)	Yes	Clear definition of treatment
**Consistency** (adherence definition)	Yes	Clear definition of perfect use
**Exclusion restriction**		
**Strong Monotonicity**		
**Ignorability**	None	
**Overall Notes**		
**Cochrane CDPLG**		**Notes**
**Data form completed date** (dd/mm/yyyy)	2/7/2022	
**Study author contact details**	victoria.jennings@georgetown.edu	
Methods	**Descriptions as stated in report/paper**	**Location in text or source** (pg & ¶/fig/table/other)
Aim of study (e.g. efficacy, equivalence, pragmatic)	Effectiveness and efficacy	pg. 3 Introduction
Design (e.g. parallel, crossover, non-RCT)	Prospective Observational Study	pg. 3 Introduction
Unit of allocation (by individuals, cluster/ groups or body parts)	Individual	
Participants	**Descriptions as stated in report/paper**	**Location in text or source** (pg & ¶/fig/table/other)
Inclusion criteria	- "Fertile women at risk of pregnancy and wanting to avoid pregnancy for at least 1 year"<br>- 18–39 years old<br>- Intending to use Dot for pregnancy prevention for at least 1 year<br>- Had not used hormonal contraception in the last three cycles<br>- "Sexually active with a male partner (or partners)"<br>'- "Had consistent cycles between 20-40 days long with less than 10 days' variation"<br>- "Had at least three menstrual periods following the most recent (if any) pregnancy"	pg. 3 Inclusion Criteria
**Exclusion criteria**		
**Total no. randomised** (or total pop. at start of study for NRCTs)	718	pg. 3 Inclusion Criteria
**Clusters** (if applicable, no., type, no. people per cluster)	NA	
**Withdrawals and exclusions** (if not provided below by outcome)	357 women exited the study	pg. 4 Table 2<br>pg. 6 Attribution Analysis
Intervention Groups	**Descriptions as stated in report/paper**	**Location in text or source** (pg & ¶/fig/table/other)
**Group name**	DOT (Dynamic Optimal Timing)	pg. 2 Abstract
**No. randomised to group** (specify whether no. people or clusters)	718	pg. 3 Inclusion Criteria
**Timing** (e.g. frequency, duration of each episode)	Start of each menstruation	pg. 2 Introduction
**Co-interventions**	Condom use and/or emergency contraception use	pg. 3 Data Collection
**Integrity of delivery**	Women had to have downloaded the app to enroll in the study	pg. 3 Participant Recruitment
**Compliance**	99% of women who completed a cycle completed 100% of their daily diaries<br>The percent of women recording the start of menstruation was not reported	pg. 5 Typical- and Perfect-Use Effectiveness and Discontinuation
Outcomes	**Descriptions as stated in report/paper**	**Location in text or source** (pg & ¶/fig/table/other)
**Outcome name**	13 Cycle failure rate	pg. 4 Data Analysis
**Outcome definition** (with diagnostic criteria if relevant)(include name, time, and analysis method)	Likelihood of pregancy within one year of use	
**Imputation of missing data** (e.g. assumptions made for ITT analysis)	92 people lost to follow up<br>Participants categorized as very unlikely pregnant or likely pregnant based on behavior in fertile window	pg. 4 Data Analysis
**Power** (e.g. power & sample size calculation, level of power achieved)	"We estimated that we needed to recruit over 700 women in order to achieve a sample size of > 255. This sample size provided 90% power to detect a 6% decrease in the 1-year pregnancy rate of app users." (unclear what estimate this was compared to)	pg. 3 Participant Recruitment
**Risk of Bias** (Based on Cochrane RoB Tool 2 and ROBINS-I)		Link to RoB Algorithm
**Primary Analysis RoB Assessment**		Notes
**Is the primary analysis evaluating effectiveness or efficacy?**	Effectiveness	
**Comparator**	No comparator stated	Their power statement mentions a 6% drop but don't say what the comparison is
**Outcome Being Assessed**	13-cycle typical use failure rate measured with Kaplan Meier	
**Specify the Numerical Result Being Assessed**	5.8% (95% CI: 3.57%, 8.09%)	
**Bias due to randomization (RCT only)**		Notes
1.1 Was the allocation sequence random?	NA	
1.2. Was the allocation sequence concealed until participants were enrolled and assigned to interventions?	NA	
1.3. Did baseline differences between intervention groups suggest a problem with the randomization process?	NA	
**Bias due to confounding**		Notes
1.1 Is there potential for confounding of the effect of intervention in this study?	Yes	
1.2. If Y/PY to 1.1 Was the analysis based on splitting participants' follow up time according to intervention received?	No	
1.3. If Y/PY to 1.2 Were intervention discontinuations or switches likely to be related to factors that are prognostic for the outcome?		
**Questions relating to baseline confounding only**		
1.4. If Y/PY to 1.1 Did the authors use an appropriate analysis method that controlled for all the important confounding domains?	Yes	They performed a Cox regression to see if demographics were related to pregnancy
1.5. If Y/PY to 1.4: Were confounding domains that were controlled for measured validly and reliably by the variables available in this study?	Yes	
1.6. Did the authors control for any post-intervention variables that could have been affected by the intervention?	No	
**Questions relating to baseline and time-varying confounding**		
1.7. If Y/PY to 1.3 Did the authors use an appropriate analysis method that controlled for all the important confounding domains and for time-varying confounding?		

Article Key

1.8. If Y/PY to 1.7: Were confounding domains that were controlled for measured validly and reliably by the variables available in this study?			
**Bias in selection of participants into the study (Not evaluating for analyses of effectiveness)**		Notes	Should fill out
2.1. Was selection of participants into the analysis based on participant characteristics observed after the start of intervention?			
2.2. If Y/PY to 2.1: Were the post-intervention variables that influenced selection likely to be associated with intervention?			
2.3 If Y/PY to 2.2: Were the post-intervention variables that influenced selection likely to be influenced by the outcome or a cause of the outcome?			
2.4. Do start of follow-up and start of intervention coincide for most participants?			
2.5. If Y/PY to 2.2 and 2.3, or N/PN to 2.4: Were adjustment techniques used that are likely to correct for the presence of selection biases?			
**Bias in classification of interventions**		Notes	
3.1 Were intervention groups clearly defined? Was the definition of adherence clearly defined?	Yes		
3.2 Was the information used to define intervention groups recorded at the start of the intervention?	Yes	Clearly defined in preregistration	
3.3 Could classification of intervention status have been affected by knowledge of the outcome or risk of the outcome?	No		
**Bias due to deviations from intended interventions**		Notes	
**ROBINS-I**			
4.1. Were there deviations from the intended intervention beyond what would be expected in usual practice?	No		
4.2. If Y/PY to 4.1: Were these deviations from intended intervention unbalanced between groups and likely to have affected the outcome?			
4.3. Were important co-interventions balanced across intervention groups?	NA		
4.4. Was the intervention implemented successfully for most participants?	Yes	All included participants needed to download the app	
4.5. Did study participants adhere to the assigned intervention regimen?	No	99% of women who completed a cycle provided information for all days but only 24% of cycles were considered perfect use where women did not report unprotected sex on risky days	
4.6. If N/PN to 4.3, 4.4 or 4.5: Was an appropriate analysis used to estimate the effect of starting and adhering to the intervention?	No	They did not meet the criteria needed for the chosen method of analysis	
Risk of bias: Assignment to intervention	**Low** / Moderate / Serious / Critical / NI		
Risk of bias: Starting and adhering to intervention	Low / Moderate / Serious / **Critical** / NI		
**Risk of bias judgement**	Low / Moderate / Serious / **Critical** / NI		
**RoB Tool (Risk of bias due to deviations from the intended interventions (effect of adhering to intervention))**			
2.1. Were participants aware of their assigned intervention during the trial?	Yes		
2.2. Were carers and people delivering the interventions aware of participants' assigned intervention during the trial?	NA		
2.3. If Y/PY/NI to 2.1 or 2.2: Were important non protocol interventions balanced across intervention groups?	No		
2.4. [If applicable:] Were there failures in implementing the intervention that could have affected the outcome?	No		
2.5. [If applicable:] Was there non-adherence to the assigned intervention regimen that could have affected participants' outcomes?	No	99% of women who completed a cycle provided information for all days but only 24% of cycles were considered perfect use where women did not report unprotected sex on risky days	
2.6. If N/PN/NI to 2.3, or Y/PY/NI to 2.4 or 2.5: Was an appropriate analysis used to estimate the effect of adhering to the intervention?	No	They did not meet the criteria needed for the chosen method of analysis	
**Risk of bias judgement**	Low/**High**/Some Concerns		
**Bias due to missing data**		Notes	
5.1 Were outcome data available for all, or nearly all, participants?	No	Data missing for 92/718 participants	
5.2 Were participants excluded due to missing data on intervention status?	Probably no	Cycles were excluded when no sexual history was entered but particpants were not excluded	
5.3 Were participants excluded due to missing data on other variables needed for the analysis?	No		
5.4 If PN/N to 5.1, or Y/PY to 5.2 or 5.3: Are the proportion of participants and reasons for missing data similar across interventions?	NA		
5.5 If PN/N to 5.1, or Y/PY to 5.2 or 5.3: Is there evidence that results were robust to the presence of missing data?	No	They performed a sensitivity analysis looking at the impact of missing data and found that the CI for the failure rate increased to 9.7-16%	
**Bias in measurement of outcomes**		Notes	
6.1 Could the outcome measure have been influenced by knowledge of the intervention received?	No		
6.2 Were outcome assessors aware of the intervention received by study participants? (blinding of assessors)	Yes		
6.3 Were the methods of outcome assessment comparable across intervention groups?	NA		
6.4 Were any systematic errors in measurement of the outcome related to intervention received?	No		
**Bias in selection of the reported result**		Notes	
Were the data that produced this result analysed in accordance with a pre-specified analysis plan that was finalized before unblinded outcome data were available for analysis?	Yes	The article was preregistered on clinicaltrials.gov and they provided their full protocol	
Is the reported effect estimate likely to be selected, on the basis of the results, from...			
7.1. ... multiple outcome measurements within the outcome domain?	No		
7.2 ... multiple analyses of the intervention-outcome relationship?	No		
7.3 ... different subgroups?	No		
**Secondary Analysis RoB Assessment**		Notes	
**Is the seconday analysis evaluating effectiveness or efficacy?**	Efficacy		
**Comparator**	Not clearly stated		
**Outcome Being Assessed**	Perfect-use failure rate		
**Specify the Numerical Result Being Assessed**	1.0% (95% CI: 0.9%, 2.9%)		
**Bias due to randomization (See primary analysis section)**		Notes	
**Bias due to confounding**		Notes	
1.1 Is there potential for confounding of the effect of intervention in this study?	Yes		
1.2. If Y/PY to 1.1 Was the analysis based on splitting participants' follow up time according to intervention received?	No		
1.3. If Y/PY to 1.2 Were intervention discontinuations or switches likely to be related to factors that are prognostic for the outcome?			
**Questions relating to baseline confounding only**			
1.4. If Y/PY to 1.1 Did the authors use an appropriate analysis method that controlled for all the important confounding domains?	No	A Cox regression was not performed when looking at perfect use	
1.5. If Y/PY to 1.4: Were confounding domains that were controlled for measured validly and reliably by the variables available in this study?			
1.6. Did the authors control for any post-intervention variables that could have been affected by the intervention?	No		
**Questions relating to baseline and time-varying confounding**			
1.7. If Y/PY to 1.3 Did the authors use an appropriate analysis method that controlled for all the important confounding domains and for time-varying confounding?			
1.8. If Y/PY to 1.7: Were confounding domains that were controlled for measured validly and reliably by the variables available in this study?			
**Bias in selection of participants into the study (Not evaluating for analyses of effectiveness)**		Notes	

2.1. Was selection of participants into the analysis based on participant characteristics observed after the start of intervention?	Yes	
2.2. If Y/PY to 2.1: Were the post-intervention variables that influenced selection likely to be associated with intervention?	Probably No	Participants uploaded sufficient data about their sexual behavior for most cycles
2.3 If Y/PY to 2.2: Were the post-intervention variables that influenced selection likely to be influenced by the outcome or a cause of the outcome?		
2.4. Do start of follow-up and start of intervention coincide for most participants?	Yes	
2.5. If Y/PY to 2.2 and 2.3, or N/PN to 2.4: Were adjustment techniques used that are likely to correct for the presence of selection biases?	No	No potential confounders were accounted for when estimating perfect use
**Bias in classification of interventions**		Notes
3.1 Were intervention groups clearly defined?	Yes	The definition for perfect adherence was clearly defined and preregistered
3.2 Was the information used to define intervention groups recorded at the start of the intervention?	Yes	The definition for perfect adherence was clearly defined and preregistered
3.3 Could classification of intervention status have been affected by knowledge of the outcome or risk of the outcome?	No	Classification was based on a preregistered definition of adherence
**Bias due to deviations from intended interventions (See primary analysis section)**		Notes
**Bias due to missing data**		Notes
5.1 Were outcome data available for all, or nearly all, participants?	Yes	There would have been full data for all perfect use cycles
5.2 Were participants excluded due to missing data on intervention status?	Probably no	Cycles were excluded when no sexual history was entered but particpants were not excluded
5.3 Were participants excluded due to missing data on other variables needed for the analysis?	Yes	Participants who didn't report activity for all of their days would not have been included
5.4 If PN/N to 5.1, or Y/PY to 5.2 or 5.3: Are the proportion of participants and reasons for missing data similar across interventions?	Yes	The same form of censoring was used for correct and incorrect use groups
5.5 If PN/N to 5.1, or Y/PY to 5.2 or 5.3: Is there evidence that results were robust to the presence of missing data?	No	There couldn't be missing outcome data for the perfect use calculation they couldn't perform this sensitivity analysis
**Bias in measurement of outcomes**		Notes
6.1 Could the outcome measure have been influenced by knowledge of the intervention received?	No	
6.2 Were outcome assessors aware of the intervention received by study participants? (blinding of assessors)	Yes	
6.3 Were the methods of outcome assessment comparable across intervention groups?	Yes	
6.4 Were any systematic errors in measurement of the outcome related to intervention received?	Probably Yes	Participants only received a pop-up message after >40 days since entering their last cycle start.<br>It is possible that women interacted with the app more since they had to record their sexual behavior and normally wouldn't have to
6.5 Could adherence have been influenced by study participation?		
**Bias in selection of the reported result**		Notes
Were the data that produced this result analysed in accordance with a pre-specified analysis plan that was finalized before unblinded outcome data were available for analysis?	Yes	
Is the reported effect estimate likely to be selected, on the basis of the results, from...		
7.1. ... multiple outcome measurements within the outcome domain?	No	
7.2 ... multiple analyses of the intervention-outcome relationship?	No	
7.3 ... different subgroups?	no	

Article Key

Criteria for Adherence Metrics and Analysis		Notes
**Device name and indication for use**	EndeavorRx (game-like app intended to improve attention and relative cognitive control processes for ADHD)	
**Type of device** (Long-term vs short-term use)	Short-term use	
**Type of device** (Presence or absence of a prescribed dosage)	Presence of prescribed dose	
**Was adherence information collected?**	Yes	
**What information was collected about usage adherence?**	Information about the number of sessions completed in the treatment group and number of minutes played in the control group	
**What information was collected about adherence to recommendations?**	NA	No explicit app recommendations
**Was information collected internally in the app?**	Yes	
**Was information modified internally?**	No	Parents were emailed in the case of 48 hours of inactivity
**Was initiation reported?**	No	
**What was average adherence?** (if reported)		
**Was implementation reported?**	Yes	
**What was average adherence?** (if reported)	83%	
**Was persistence reported?**	No	
**What was average adherence?** (if reported)		
**Was adherence low?**	No	
**Was adherence not reported or low and could have affected outcomes?**	No	
**Was efficacy analyzed?**	Yes	
**What method was used?**	ITT analysis	
**Were efficacy analyses preregistered?**		
**What assumptions are required for that method to study efficacy?**	SUTVA, positivity, consistency, randomization	
**Did the article report evidence that the assumptions were met?**	Yes	
**SUTVA**	Yes	Treatment only administered through the app
**Positivity**	Yes	Randomized controlled trial
**Consistency** (treatment definition)	Yes	Clear definition of treatment
**Consistency** (adherence definition)		
**Exclusion restriction**		
**Strong Monotonicity**		
**Ignorability**	Yes	Randomized controlled trial
**Overall Notes**		
**Cochrane CDPLG**		**Notes**
**Data form completed date** (dd/mm/yyyy)	2/22/2022	
**Study author contact details**	scott.kollins@duke.edu	
Methods	**Descriptions as stated in report/paper**	**Location in text or source** (pg & ¶/fig/table/other)
Aim of study (e.g. efficacy, equivalence, pragmatic)	Efficacy and tolerability	pg. 3 Introduction
Design (e.g. parallel, crossover, non-RCT)	Randomized controlled trial	pg. 3 Study Design
Unit of allocation (by individuals, cluster/ groups or body parts)	Individual	pg. 3 Randomization and Masking
Participants	**Descriptions as stated in report/paper**	**Location in text or source** (pg & ¶/fig/table/other)
**Inclusion criteria**	"● Age 8 years to 12 years, inclusive, at the time of parental informed consent<br>● Male or female<br>● Confirmed ADHD diagnosis, any presentation, at Screening based on DSM-V criteria and established via<br>the MINI-KID1 administered by a trained clinician<br>● Screening/Baseline (visit 1 or 1a) score on the clinician-rated ADHD-RS-IV2 score ≥28<br>● Screening/Baseline (visit 1 or 1a) score on the TOVA API ≤−1·8<br>● Not undergoing pharmacological treatment with methylphenidate or amphetamine-based products at time<br>of Screening; or, if undergoing pharmacological treatment, must be willing and appropriate (i.e., not<br>optimally treated in the investigator's judgment) to wash out of current regimen<br>● Ability to follow written and verbal instructions (English), as assessed by the PI and/or study coordinator<br>● Estimated IQ score ≥80 as assessed by the Kaufmann Brief Intelligence Test, Second Edition (KBIT-II)<br>● Ability to comply with all the testing and requirements"	eAppendix 1. Inclusion Criteria
**Exclusion criteria**	● Current, controlled (requiring a restricted medication) or uncontrolled, comorbid psychiatric diagnosis , based on MINI-KID and subsequent clinical interviewing, with significant symptoms including but not limited to posttraumatic stress disorder, psychosis, bipolar illness, pervasive developmental disorder, severe obsessive compulsive disorder, severe depressive or severe anxiety disorder, conduct disorder, or other symptomatic manifestations that in the opinion of the Investigator that may confound study data/assessments. Patients with clinical history of learning disorders will be allowed to participate, provided the disorder does not impact their ability to participate in the trial based on PI judgment<br>● Patients who are currently treated with a non-stimulant medication for ADHD (i.e., atomoxetine, clonidine, guanfacine)<br>● Initiation within the last 4 weeks of behavioral therapy. Patients who have been in behavior therapy consistently for more than 4 weeks may participate provided their routine is unchanged during the course of the study. Patients planning on changing or initiating behavior therapy during the course of the study will be excluded<br>● Patient is currently considered a suicide risk in the opinion of the Investigator, has previously made a suicide attempt, or has a prior history of, or is currently demonstrating active suicidal ideation or self- injurious behavior as measured by C-SSRS at screening<br>● Motor condition (e.g., physical deformity of the hands/arms; prostheses) that prevents game playing as reported by the parent or observed by the investigator<br>● Recent history (within the past 6 months) of suspected substance abuse or dependence<br>● History of seizures (exclusive of febrile seizures), or significant motor or vocal tics, including but not<br>limited to Tourette's Disorder<br>● Has participated in a clinical trial within 90 days prior to screening<br>● Diagnosis of or parent-reported color blindness (Confirmed in-clinic via ICBT)<br>● Uncorrected visual acuity (Confirmed in-clinic via ability of subject to play the game)<br>● Regular use of psychoactive drugs (other than stimulant) that in the opinion of the Investigator may	eAppendix 2. Exclusion Criteria
**Total no. randomised** (or total pop. at start of study for NRCTs)	348	pg. 5 Figure 1
**Clusters** (if applicable, no., type, no. people per cluster)		
**Withdrawals and exclusions** (if not provided below by outcome)	19 withdrawn or lost to follow-up	pg. 5 Figure 1
Intervention Groups	**Descriptions as stated in report/paper**	**Location in text or source** (pg & ¶/fig/table/other)
**Group name**	AKL-T01	pg. 5 Figure 1
**No. randomised to group** (specify whether no. people or clusters)	180	pg. 5 Figure 1
**Timing** (e.g. frequency, duration of each episode)	5 sessions per day (total time on task about 25 min), 5 days per week, for 4 weeks	pg. 4 Procedures
**Co-interventions**	Participants were not allowed to be on disorder related medications	pg. 1 Methods
**Integrity of delivery**	1 participant was assigned the wrong group	pg. 5 Figure 1
**Compliance**	83% of instructed use (A mean of 83 out of 100 expected sessions played)	pg. 6 Results
Intervention Groups	**Descriptions as stated in report/paper**	**Location in text or source** (pg & ¶/fig/table/other)
**Group name**	Control	pg. 5 Figure 1
**No. randomised to group** (specify whether no. people or clusters)	168	pg. 5 Figure 1
**Timing** (e.g. frequency, duration of each episode)	5 sessions per day (total time on task about 25 min), 5 days per week, for 4 weeks	pg. 4 Procedures
**Co-interventions**	Participants were not allowed to be on disorder related medications	pg. 1 Methods
**Integrity of delivery**	1 participant was assigned the wrong group	pg. 5 Figure 1

**Compliance**	96% of instructed use (480.7/500 expected minutes of play)	pg. 6 Results
Outcomes	**Descriptions as stated in report/paper**	**Location in text or source** (pg & ¶/fig/table/other)
**Outcome name**	Mean change in TOVA API from pre to post intervention with ITT analysis	pg. 4 Outcomes
**Outcome definition** (with diagnostic criteria if relevant)(include name, time, and analysis method)	"validated, computerised, continuous performance test that objectively measures attention and inhibitory control, normalised by age and sex."<br>"composite score of the sum of three scores: reaction time (RT) mean Half-1 (highly infrequent targets), RT variability total (both halves), and d-prime Half-2 (highly frequent targets)"	pg. 4 Outcomes
**Imputation of missing data** (e.g. assumptions made for ITT analysis)		
**Power** (e.g. power & sample size calculation, level of power achieved)	"Power analyses determined that a sample size of 150 patients per intervention group would be sufficient to detect an effect size of 0·40 with 90% or more power on a two-tailed, between-patients t test and α criterion of 0·05"	pg. 4 Statistical Analysis
**Risk of Bias** (Based on Cochrane RoB Tool 2 and ROBINS-I)		Link to RoB Algorithm
**Primary Analysis RoB Assessment**		Notes
**Is the primary analysis evaluating effectiveness or efficacy?**	Efficacy	
**Comparator**	Game control	
**Outcome Being Assessed**	TOVA API (4 week change form baseline, ITT effect)	
**Specify the Numerical Result Being Assessed**	"There was a significant difference between intervention groups on the primary efficacy endpoint (adjusted p=0·0060); non-parametric estimate of the population median change (Hodges-Lehmann estimate) was 0·88 (95% CI 0·24–1·49). The mean (SD) change from baseline on the TOVA API was 0·93 (3·15) in the AKL-T01 group and 0·03 (3·16) in the control group (figure 2)"	
**Bias due to randomization (RCT only)**		Notes
1.1 Was the allocation sequence random?	Yes	
1.2. Was the allocation sequence concealed until participants were enrolled and assigned to interventions?	Yes	
1.3. Did baseline differences between intervention groups suggest a problem with the randomization process?	No	
**Bias due to confounding**		Notes
1.1 Is there potential for confounding of the effect of intervention in this study?	Yes	
1.2. If Y/PY to 1.1 Was the analysis based on splitting participants' follow up time according to intervention received?	No	
1.3. If Y/PY to 1.2 Were intervention discontinuations or switches likely to be related to factors that are prognostic for the outcome?		
**Questions relating to baseline confounding only**		
1.4. If Y/PY to 1.1 Did the authors use an appropriate analysis method that controlled for all the important confounding domains?	Yes	
1.5. If Y/PY to 1.4: Were confounding domains that were controlled for measured validly and reliably by the variables available in this study?	Yes	They used an RCT and ITT analysis and checked for baseline confounders
1.6. Did the authors control for any post-intervention variables that could have been affected by the intervention?	No	
**Questions relating to baseline and time-varying confounding**		
1.7. If Y/PY to 1.3 Did the authors use an appropriate analysis method that controlled for all the important confounding domains and for time-varying confounding?		
1.8. If Y/PY to 1.7: Were confounding domains that were controlled for measured validly and reliably by the variables available in this study?		
**Bias in selection of participants into the study (Not evaluating for analyses of effectiveness)**		Notes
2.1. Was selection of participants into the analysis based on participant characteristics observed after the start of intervention?		
2.2. If Y/PY to 2.1: Were the post-intervention variables that influenced selection likely to be associated with intervention?		
2.3 If Y/PY to 2.2: Were the post-intervention variables that influenced selection likely to be influenced by the outcome or a cause of the outcome?		
2.4. Do start of follow-up and start of intervention coincide for most participants?		
2.5. If Y/PY to 2.2 and 2.3, or N/PN to 2.4: Were adjustment techniques used that are likely to correct for the presence of selection biases?		
**Bias in classification of interventions**		Notes
3.1 Were intervention groups clearly defined? Was the definition of adherence clearly defined?	Yes	
3.2 Was the information used to define intervention groups recorded at the start of the intervention?	Yes	Clear definition of intervention on preregistration
3.3 Could classification of intervention status have been affected by knowledge of the outcome or risk of the outcome?	No	
**Bias due to deviations from intended interventions**		Notes
**ROBINS-I**		
4.1. Were there deviations from the intended intervention beyond what would be expected in usual practice?	No	
4.2. If Y/PY to 4.1: Were these deviations from intended intervention unbalanced between groups and likely to have affected the outcome?		
4.3. Were important co-interventions balanced across intervention groups?	Yes	
4.4. Was the intervention implemented successfully for most participants?	Probably Yes	Participants were monitored at the first visit to make sure they were usingthe app correctly
4.5. Did study participants adhere to the assigned intervention regimen?	Probably Yes	An average of 83% of the sessions were completed
4.6. If N/PN to 4.3, 4.4 or 4.5: Was an appropriate analysis used to estimate the effect of starting and adhering to the intervention?		
Risk of bias: Assignment to intervention	**Low** / Moderate / Serious / Critical / NI	
Risk of bias: Starting and adhering to intervention	**Low** / Moderate / Serious / Critical / NI	
**Risk of bias judgement**	**Low** / Moderate / Serious / Critical / NI	
**RoB Tool (Risk of bias due to deviations from the intended interventions (effect of adhering to intervention))**		
2.1. Were participants aware of their assigned intervention during the trial?	No	Participants were aware in both groups but blinded to what group they were in
2.2. Were carers and people delivering the interventions aware of participants' assigned intervention during the trial?	No	They were aware of the intervention but not the group assignment
2.3. If Y/PY/NI to 2.1 or 2.2: Were important non protocol interventions balanced across intervention groups?	NA	
2.4. [If applicable:] Were there failures in implementing the intervention that could have affected the outcome?	No information	The article did not explicit address this
2.5. [If applicable:] Was there non-adherence to the assigned intervention regimen that could have affected participants' outcomes?	Probably No	An average of 83% of the sessions were completed
2.6. If N/PN/NI to 2.3, or Y/PY/NI to 2.4 or 2.5: Was an appropriate analysis used to estimate the effect of adhering to the intervention?		Adherence was high so it made sense that they did not analyze adherence information
**Risk of bias judgement**	**Low**/High/Some Concerns	
**Bias due to missing data**		Notes
5.1 Were outcome data available for all, or nearly all, participants?	Yes	19/348 participants withdrew or were lost to follow-up<br>They also had a prespecified rule for when they would account for missing data
5.2 Were participants excluded due to missing data on intervention status?	No	
5.3 Were participants excluded due to missing data on other variables needed for the analysis?	No	
5.4 If PN/N to 5.1, or Y/PY to 5.2 or 5.3: Are the proportion of participants and reasons for missing data similar across interventions?		
5.5 If PN/N to 5.1, or Y/PY to 5.2 or 5.3: Is there evidence that results were robust to the presence of missing data?		
**Bias in measurement of outcomes**		Notes

6.1 Could the outcome measure have been influenced by knowledge of the intervention received?	No	Participants were sufficiently blind to their intervention group
6.2 Were outcome assessors aware of the intervention received by study participants? (blinding of assessors)	No	
6.3 Were the methods of outcome assessment comparable across intervention groups?	Yes	
6.4 Were any systematic errors in measurement of the outcome related to intervention received?	No	
**Bias in selection of the reported result**		Notes
Were the data that produced this result analysed in accordance with a pre-specified analysis plan that was finalized before unblinded outcome data were available for analysis?	Yes	
Is the reported effect estimate likely to be selected, on the basis of the results, from...		
7.1. ... multiple outcome measurements within the outcome domain?	No	
7.2 ... multiple analyses of the intervention-outcome relationship?	Probably No	They mention using a per-protocol t-test in the protocol but also say that this test will not be used if the necessary assumptions do not hold
7.3 ... different subgroups?	No	
**Secondary Analysis RoB Assessment**		Notes
**Is the seconday analysis evaluating effectiveness or efficacy?**		
**Comparator**		
**Outcome Being Assessed**		
**Specify the Numerical Result Being Assessed**		
**Bias due to randomization (See primary analysis section)**		Notes
**Bias due to confounding**		Notes
1.1 Is there potential for confounding of the effect of intervention in this study?		
1.2. If Y/PY to 1.1 Was the analysis based on splitting participants' follow up time according to intervention received?		
1.3. If Y/PY to 1.2 Were intervention discontinuations or switches likely to be related to factors that are prognostic for the outcome?		
**Questions relating to baseline confounding only**		
1.4. If Y/PY to 1.1 Did the authors use an appropriate analysis method that controlled for all the important confounding domains?		
1.5. If Y/PY to 1.4: Were confounding domains that were controlled for measured validly and reliably by the variables available in this study?		
1.6. Did the authors control for any post-intervention variables that could have been affected by the intervention?		
**Questions relating to baseline and time-varying confounding**		
1.7. If Y/PY to 1.3 Did the authors use an appropriate analysis method that controlled for all the important confounding domains and for time-varying confounding?		
1.8. If Y/PY to 1.7: Were confounding domains that were controlled for measured validly and reliably by the variables available in this study?		
**Bias in selection of participants into the study (Not evaluating for analyses of effectiveness)**		Notes
2.1. Was selection of participants into the analysis based on participant characteristics observed after the start of intervention?		
2.2. If Y/PY to 2.1: Were the post-intervention variables that influenced selection likely to be associated with intervention?		
2.3 If Y/PY to 2.2: Were the post-intervention variables that influenced selection likely to be influenced by the outcome or a cause of the outcome?		
2.4. Do start of follow-up and start of intervention coincide for most participants?		
2.5. If Y/PY to 2.2 and 2.3, or N/PN to 2.4: Were adjustment techniques used that are likely to correct for the presence of selection biases?		
**Bias in classification of interventions**		Notes
3.1 Were intervention groups clearly defined?		
3.2 Was the information used to define intervention groups recorded at the start of the intervention?		
3.3 Could classification of intervention status have been affected by knowledge of the outcome or risk of the outcome?		
**Bias due to deviations from intended interventions (See primary analysis section)**		Notes
**Bias due to missing data**		Notes
5.1 Were outcome data available for all, or nearly all, participants?		
5.2 Were participants excluded due to missing data on intervention status?		
5.3 Were participants excluded due to missing data on other variables needed for the analysis?		
5.4 If PN/N to 5.1, or Y/PY to 5.2 or 5.3: Are the proportion of participants and reasons for missing data similar across interventions?		
5.5 If PN/N to 5.1, or Y/PY to 5.2 or 5.3: Is there evidence that results were robust to the presence of missing data?		
**Bias in measurement of outcomes**		Notes
6.1 Could the outcome measure have been influenced by knowledge of the intervention received?		
6.2 Were outcome assessors aware of the intervention received by study participants? (blinding of assessors)		
6.3 Were the methods of outcome assessment comparable across intervention groups?		
6.4 Were any systematic errors in measurement of the outcome related to intervention received?		
6.5 Could adherence have been influenced by study participation?		
**Bias in selection of the reported result**		Notes
Were the data that produced this result analysed in accordance with a pre-specified analysis plan that was finalized before unblinded outcome data were available for analysis?		
Is the reported effect estimate likely to be selected, on the basis of the results, from...		
7.1. ... multiple outcome measurements within the outcome domain?		
7.2 ... multiple analyses of the intervention-outcome relationship?		
7.3 ... different subgroups?		

Criteria for Adherence Metrics and Analysis		Notes
**Device name and indication for use**	EndeavorRx (Video game treatment for ADHD)	
**Type of device** (Long-term vs short-term use)	Short-term use	
**Type of device** (Presence or absence of a prescribed dosage)	Presence of a prescribed dose	
**Was adherence information collected?**	Yes	
**What information was collected about usage adherence?**	Information about completion of missions	
**What information was collected about adherence to recommendations?**	NA	No explicit app recommendations
**Was information collected internally in the app?**	Yes	
**Was information modified internally?**	Yes	Reminders were sent through the app
**Was initiation reported?**	Yes	
**What was average adherence?** (if reported)	100%	All participants in both groups used the app in the first week
**Was implementation reported?**	Yes	
**What was average adherence?** (if reported)	81.1 (28.37) missions for the On Stimulants cohort<br>73.0 (33.83) missions for the No Stimulants cohort	It was recommended that 100 missions should be completed in this time period
**Was persistence reported?**	No	
**What was average adherence?** (if reported)		
**Was adherence low?**	Yes	Average adherence was below 80% between the two groups
**Was adherence not reported or low and could have affected outcomes?**	Yes	
**Was efficacy analyzed?**	No	
**What method was used?**	NA	
**Were efficacy analyses preregistered?**		
**What assumptions are required for that method to study efficacy?**		
**Did the article report evidence that the assumptions were met?**		
**SUTVA**		
**Positivity**		
**Consistency** (treatment definition)		
**Consistency** (adherence definition)		
**Exclusion restriction**		
**Strong Monotonicity**		
**Ignorability**		
**Overall Notes**		

Article Key

Cochrane CDPLG		Notes
**Data form completed date** (dd/mm/yyyy)	2/17/2022	
**Study author contact details**	scott.kollins@duke.edu	
Methods	**Descriptions as stated in report/paper**	**Location in text or source** (pg & ¶/fig/table/other)
Aim of study (e.g. efficacy, equivalence, pragmatic)	Effectiveness	pg. 1 Title and Introduction
Design (e.g. parallel, crossover, non-RCT)	Observational Cohort Trial	pg. 6 Study Design
Unit of allocation (by individuals, cluster/ groups or body parts)	Individual	
Participants	**Descriptions as stated in report/paper**	**Location in text or source** (pg & ¶/fig/table/other)
**Inclusion criteria**	"- Male and female children between the ages of 8-14 years<br>- Confirmed ADHD diagnosis (primarily inattentive or combined subtype) at Screening based on DSM-V criteria and established via the MINI-KID, administered by a trained clinician.<br>- Experiencing suboptimal treatment of ADHD (IRS ≥ 3 Overall Impairment Score), at Screening.<br>- Estimated IQ score ≥ 80 as assessed by the Kaufmann Brief Intelligence Test, Second Edition (KBIT-II).<br>- For the On Stimulants cohort, participants must have been stable on stimulant medication, at an approved dose, for ≥ 30 days prior to enrollment.<br>- For the No Stimulants cohort, participants must be stable off stimulant medication for ≥ 30 days prior to enrollment"	Supplementary Note 1
**Exclusion criteria**	"● Current, controlled (requiring a restricted medication) or uncontrolled, comorbid psychiatric diagnosis, based on MINI-KID<br>● Participants currently treated with a nonstimulant medication for ADHD (i.e., atomoxetine, clonidine, or guanfacine).<br>● Initiation or planned change of behavioral therapy within the last 4 weeks from the time of<br>consent or during trial.<br>● Participant was considered a suicide risk in the opinion of the Investigator, had previously<br>made a suicide attempt, or had a prior history of, or was demonstrating active suicidal ideation or self-injurious behavior as measured by Columbia-Suicide Severity Rating Scale (C-SSRS) at Screening.<br>● Motor condition (e.g., physical deformity of the hands/arms; prostheses) that prevented<br>playing the digital treatment as reported by the parent or observed by the investigator.<br>● Recent history (within the past six months) of suspected substance abuse or dependence.<br>● History of seizures (exclusive of febrile seizures), or significant motor or vocal tics, including but not limited to Tourette's Disorder.<br>● Diagnosis of or parent-reported color blindness (Confirmed in-clinic via Ishihara Color Blindness Test).<br>● Uncorrected visual acuity (confirmed via ability of participant to log-in to T01 app and complete in-clinic game play, at baseline).<br>● Regular use of psychoactive drugs (nonstimulant) that in the opinion of the Investigator may confound study data/assessments"	Supplementary Note 1
**Total no. randomised** (or total pop. at start of study for NRCTs)	206	pg. 1 Results
**Clusters** (if applicable, no., type, no. people per cluster)		
**Withdrawals and exclusions** (if not provided below by outcome)	27 dropouts	pg. 2 Results
Intervention Groups	**Descriptions as stated in report/paper**	**Location in text or source** (pg & ¶/fig/table/other)
**Group name**	On Stimulants	pg. 1 Results
**No. randomised to group** (specify whether no. people or clusters)	130	pg. 1 Results
**Timing** (e.g. frequency, duration of each episode)	Participants were instructed to take medications as prescribed and use the app 25 min per day for 5 days/week of app over two four week treatment periods	pg. 2 Compliance
**Co-interventions**		
**Integrity of delivery**	Intervention was preloaded and administered to all participants at baseline visit<br>100% of participants played at least one mission in the first week	pg. 6 Procedures<br>Supplementary Figure 1
**Compliance**	Four participants stopped stimulant use during treatment period<br>135.2 (56.20) missions completed in 2 month period (200 max)	pg. 2 Participants<br>pg. 2 Compliance
Intervention Groups	**Descriptions as stated in report/paper**	**Location in text or source** (pg & ¶/fig/table/other)
**Group name**	No Stimulants	pg. 1 Results
**No. randomised to group** (specify whether no. people or clusters)	76	pg. 1 Results
**Timing** (e.g. frequency, duration of each episode)	Participants were instructed to use the app 25 min per day for 5 days/week of app over two four week treatment periods	pg. 2 Compliance
**Co-interventions**		
**Integrity of delivery**	Intervention was preloaded and administered to all participants at baseline visit<br>100% of participants played at least one mission in the first week	pg. 6 Procedures<br>Supplementary Figure 1

**Compliance**	All participants stayed off stimulants<br>116.4 (65.69) missions completed in 2 months (200 max)	pg. 2 Participants<br>pg. 2 Compliance
Outcomes	**Descriptions as stated in report/paper**	**Location in text or source** (pg & ¶/fig/table/other)
**Outcome name**	Change in adhd related impairment measured on the impairment rating scale (IRS)	pg. 1 Abstract
**Outcome definition** (with diagnostic criteria if relevant)(include name, time, and analysis method)	"impairment in several areas, such as academic functioning, shy and passive social behavior, and impaired adaptive functioning in children/adolescents"	pg. 1 Introduction
**Imputation of missing data** (e.g. assumptions made for ITT analysis)	Missing data were not imputed	pg. 7 Statistical Power
**Power** (e.g. power & sample size calculation, level of power achieved)	planned for 203 participants for a 10% dropout rate and 90% power	pg. 7 Statistical Power
**Risk of Bias** (Based on Cochrane RoB Tool 2 and ROBINS-I)		Link to RoB Algorithm
**Primary Analysis RoB Assessment**		Notes
**Is the primary analysis evaluating effectiveness or efficacy?**	Effectiveness	
**Comparator**	Baseline IRS	
**Outcome Being Assessed**	Change in IRS from Baseline to 28 days	
**Specify the Numerical Result Being Assessed**	"−0.7 (95% confidence interval (CI): [−0.86, −0.50]; DOF: 127; Cohen's d: .65; p < 0.001) in the On Stimulants cohort and −0.5 (95% CI: [−0.73, −0.32]; DOF: 73; Cohen's d: .59; p < 0.001) in the No Stimulants cohort (Fig. 2)."	
**Bias due to randomization (RCT only)**		Notes
1.1 Was the allocation sequence random?	NA	
1.2. Was the allocation sequence concealed until participants were enrolled and assigned to interventions?	NA	
1.3. Did baseline differences between intervention groups suggest a problem with the randomization process?	NA	
**Bias due to confounding**		Notes
1.1 Is there potential for confounding of the effect of intervention in this study?	Yes	Because a within group test was used it is impossible to parse out what effect was from the intervention or confounder
1.2. If Y/PY to 1.1 Was the analysis based on splitting participants' follow up time according to intervention received?	No	
1.3. If Y/PY to 1.2 Were intervention discontinuations or switches likely to be related to factors that are prognostic for the outcome?		
**Questions relating to baseline confounding only**		
1.4. If Y/PY to 1.1 Did the authors use an appropriate analysis method that controlled for all the important confounding domains?	No	They wanted to see if there was a similar effect between groups on and off stimulants but did not do a between group comparison controlling for potential confounders. They do acknowledge that they needed a controlled study to account for all confounders
1.5. If Y/PY to 1.4: Were confounding domains that were controlled for measured validly and reliably by the variables available in this study?		
1.6. Did the authors control for any post-intervention variables that could have been affected by the intervention?	No	
**Questions relating to baseline and time-varying confounding**		
1.7. If Y/PY to 1.3 Did the authors use an appropriate analysis method that controlled for all the important confounding domains and for time-varying confounding?		
1.8. If Y/PY to 1.7: Were confounding domains that were controlled for measured validly and reliably by the variables available in this study?		
**Bias in selection of participants into the study (Not evaluating for analyses of effectiveness)**		Notes
2.1. Was selection of participants into the analysis based on participant characteristics observed after the start of intervention?		
2.2. If Y/PY to 2.1: Were the post-intervention variables that influenced selection likely to be associated with intervention?		
2.3 If Y/PY to 2.2: Were the post-intervention variables that influenced selection likely to be influenced by the outcome or a cause of the outcome?		
2.4. Do start of follow-up and start of intervention coincide for most participants?		
2.5. If Y/PY to 2.2 and 2.3, or N/PN to 2.4: Were adjustment techniques used that are likely to correct for the presence of selection biases?		
**Bias in classification of interventions**		Notes
3.1 Were intervention groups clearly defined? Was the definition of adherence clearly defined?	Yes	
3.2 Was the information used to define intervention groups recorded at the start of the intervention?	Yes	Cohort definitions and treament definition were clearly defined in the preregistration
3.3 Could classification of intervention status have been affected by knowledge of the outcome or risk of the outcome?	No	
**Bias due to deviations from intended interventions**		Notes
**ROBINS-I**		
4.1. Were there deviations from the intended intervention beyond what would be expected in usual practice?	Probably No	is unclear how this relates to the general population
4.2. If Y/PY to 4.1: Were these deviations from intended intervention unbalanced between groups and likely to have affected the outcome?	Probably Yes	No participants switched in the no stimulants group. I would think that the stimulants group would have had more of a drop in the outcome if those people had stayed on the stimulants. They made up a small piece of the sample size though
4.3. Were important co-interventions balanced across intervention groups?	No information	They mention that behavioral therapy is often a co-intervention with ADHD and that participants were instructed not to change any behavioral therapy practices but they don't say how many participants in each group were receiving this
4.4. Was the intervention implemented successfully for most participants?	Yes	Reported well in the supplementary material
4.5. Did study participants adhere to the assigned intervention regimen?	Probably No	For the 28 day treatment period adherence was generally high but it was below 80%
4.6. If N/PN to 4.3, 4.4 or 4.5: Was an appropriate analysis used to estimate the effect of starting and adhering to the intervention?	No	Adherence was summarized well but the effect of adherence was not analyzed
Risk of bias: Assignment to intervention	**Low** / Moderate / Serious / Critical / NI	
Risk of bias: Starting and adhering to intervention	Low / Moderate / **Serious** / Critical / NI	
**Risk of bias judgement**	Low / **Moderate** / Serious / Critical / NI	
**RoB Tool (Risk of bias due to deviations from the intended interventions (effect of adhering to intervention))**		
2.1. Were participants aware of their assigned intervention during the trial?	Yes	
2.2. Were carers and people delivering the interventions aware of participants' assigned intervention during the trial?	Yes	
2.3. If Y/PY/NI to 2.1 or 2.2: Were important non protocol interventions balanced across intervention groups?	No	Four participants in the simulant group stopped simulant use
2.4. [If applicable:] Were there failures in implementing the intervention that could have affected the outcome?	No	All participants started using the intervention
2.5. [If applicable:] Was there non-adherence to the assigned intervention regimen that could have affected participants' outcomes?	Yes	On average participants completed 70-80% of recommended missions which could have affected outcomes
2.6. If N/PN/NI to 2.3, or Y/PY/NI to 2.4 or 2.5: Was an appropriate analysis used to estimate the effect of adhering to the intervention?	No	Adherence was summarized well but the effect of adherence was not analyzed
**Risk of bias judgement**	Low/**High**/Some Concerns	
**Bias due to missing data**		Notes
5.1 Were outcome data available for all, or nearly all, participants?	Yes	Outcome data were available for 95% of participants
5.2 Were participants excluded due to missing data on intervention status?	No	

5.3 Were participants excluded due to missing data on other variables needed for the analysis?	Yes	Participants were excluded due to missing outcome data
5.4 If PN/N to 5.1, or Y/PY to 5.2 or 5.3: Are the proportion of participants and reasons for missing data similar across interventions?	Probably Yes	There was a higher percent of dropouts in the off simulants group but it likely wasn't significantly different
5.5 If PN/N to 5.1, or Y/PY to 5.2 or 5.3: Is there evidence that results were robust to the presence of missing data?	No	No data imputation was used
**Bias in measurement of outcomes**		Notes
6.1 Could the outcome measure have been influenced by knowledge of the intervention received?	Probably Yes	Participants and patients were aware of the intervention and the outcomes were parent-reported
6.2 Were outcome assessors aware of the intervention received by study participants? (blinding of assessors)	Yes	Outcomes were parent-reported and clinician rated
6.3 Were the methods of outcome assessment comparable across intervention groups?	Yes	
6.4 Were any systematic errors in measurement of the outcome related to intervention received?	No	
**Bias in selection of the reported result**		Notes
Were the data that produced this result analysed in accordance with a pre-specified analysis plan that was finalized before unblinded outcome data were available for analysis?	Yes	https://clinicaltrials.gov/ct2/show/NCT03649074?term=NCT03649074&draw=2&rank=1
Is the reported effect estimate likely to be selected, on the basis of the results, from...		
7.1. ... multiple outcome measurements within the outcome domain?	No	
7.2 ... multiple analyses of the intervention-outcome relationship?	Probably No	
7.3 ... different subgroups?	No	
**Secondary Analysis RoB Assessment**		Notes
**Is the seconday analysis evaluating effectiveness or efficacy?**		
**Comparator**		
**Outcome Being Assessed**		
**Specify the Numerical Result Being Assessed**		
**Bias due to randomization (See primary analysis section)**		Notes
**Bias due to confounding**		Notes
1.1 Is there potential for confounding of the effect of intervention in this study?		
1.2. If Y/PY to 1.1 Was the analysis based on splitting participants' follow up time according to intervention received?		
1.3. If Y/PY to 1.2 Were intervention discontinuations or switches likely to be related to factors that are prognostic for the outcome?		
**Questions relating to baseline confounding only**		
1.4. If Y/PY to 1.1 Did the authors use an appropriate analysis method that controlled for all the important confounding domains?		
1.5. If Y/PY to 1.4: Were confounding domains that were controlled for measured validly and reliably by the variables available in this study?		
1.6. Did the authors control for any post-intervention variables that could have been affected by the intervention?		
**Questions relating to baseline and time-varying confounding**		
1.7. If Y/PY to 1.3 Did the authors use an appropriate analysis method that controlled for all the important confounding domains and for time-varying confounding?		
1.8. If Y/PY to 1.7: Were confounding domains that were controlled for measured validly and reliably by the variables available in this study?		
**Bias in selection of participants into the study (Not evaluating for analyses of effectiveness)**		Notes
2.1. Was selection of participants into the analysis based on participant characteristics observed after the start of intervention?		
2.2. If Y/PY to 2.1: Were the post-intervention variables that influenced selection likely to be associated with intervention?		
2.3 If Y/PY to 2.2: Were the post-intervention variables that influenced selection likely to be influenced by the outcome or a cause of the outcome?		
2.4. Do start of follow-up and start of intervention coincide for most participants?		
2.5. If Y/PY to 2.2 and 2.3, or N/PN to 2.4: Were adjustment techniques used that are likely to correct for the presence of selection biases?		
**Bias in classification of interventions**		Notes
3.1 Were intervention groups clearly defined?		
3.2 Was the information used to define intervention groups recorded at the start of the intervention?		
3.3 Could classification of intervention status have been affected by knowledge of the outcome or risk of the outcome?		
**Bias due to deviations from intended interventions (See primary analysis section)**		Notes
**Bias due to missing data**		Notes
5.1 Were outcome data available for all, or nearly all, participants?		
5.2 Were participants excluded due to missing data on intervention status?		
5.3 Were participants excluded due to missing data on other variables needed for the analysis?		
5.4 If PN/N to 5.1, or Y/PY to 5.2 or 5.3: Are the proportion of participants and reasons for missing data similar across interventions?		
5.5 If PN/N to 5.1, or Y/PY to 5.2 or 5.3: Is there evidence that results were robust to the presence of missing data?		
**Bias in measurement of outcomes**		Notes
6.1 Could the outcome measure have been influenced by knowledge of the intervention received?		
6.2 Were outcome assessors aware of the intervention received by study participants? (blinding of assessors)		
6.3 Were the methods of outcome assessment comparable across intervention groups?		
6.4 Were any systematic errors in measurement of the outcome related to intervention received?		
6.5 Could adherence have been influenced by study participation?		
**Bias in selection of the reported result**		Notes
Were the data that produced this result analysed in accordance with a pre-specified analysis plan that was finalized before unblinded outcome data were available for analysis?		
Is the reported effect estimate likely to be selected, on the basis of the results, from...		
7.1. ... multiple outcome measurements within the outcome domain?		
7.2 ... multiple analyses of the intervention-outcome relationship?		
7.3 ... different subgroups?		

Criteria for Adherence Metrics and Analysis		Notes
**Device name and indication for use**	ReSET-O (Cognitive Behavioral Therapy for Opioid use disorder)	
**Type of device** (Long-term vs short-term use)	Short-term use	
**Type of device** (Presence or absence of a prescribed dosage)	Presence of a prescribed dosage	
**Was adherence information collected?**	Yes	
**What information was collected about usage adherence?**	Active days of use<br>Core completion	
**What information was collected about adherence to recommendations?**	NA	No explicit app recommendations
**Was information collected internally in the app?**	Yes	
**Was information modified internally?**	NA	
**Was initiation reported?**	Yes	
**What was average adherence?** (if reported)	100% of participants completed a lesson	
**Was implementation reported?**	Yes	
**What was average adherence?** (if reported)	80% completed 8 or more core modules<br>66% completed half of all core modules	
**Was persistence reported?**	Yes	
**What was average adherence?** (if reported)	55% of individuals used the app in week 12<br>49% completed all core modules	gradual reduction in the use of the therapeutic with time starting from 100% in the first week
**Was adherence low?**	Yes	Less the 80% of participants completed all core modules
**Was adherence not reported or low and could have affected outcomes?**	Yes	
**Was efficacy analyzed?**	Yes	
**What method was used?**	Dose-response	
**Were efficacy analyses preregistered?**	No	
**What assumptions are required for that method to study efficacy?**	SUTVA, positivity, consistency, conditional independence between adherence and outcomes	
**Did the article report evidence that the assumptions were met?**	No	
**SUTVA**	Yes	Therapy only delivered through the app
**Positivity**	No	No control condition
**Consistency** (treatment definition)	Yes	Clear definition of therapy
**Consistency** (adherence definition)		
**Exclusion restriction**		
**Strong Monotonicity**		
**Ignorability**	None	
**Overall Notes**	This is a good example of how to report all facets of adherence	

Article Key

Cochrane CDPLG		Notes
**Data form completed date** (dd/mm/yyyy)	3/29/2022	
**Study author contact details**	yuri.maricich@peartherapeutics.com	
Methods	**Descriptions as stated in report/paper**	**Location in text or source** (pg & ¶/fig/table/other)
Aim of study (e.g. efficacy, equivalence, pragmatic)	Effectiveness	pg. 8 Discussion<br>pg. 8 Conclusions
Design (e.g. parallel, crossover, non-RCT)	Real-world Observational evaluation	pg. 3 Methods
Unit of allocation (by individuals, cluster/ groups or body parts)	Individual	
Participants	**Descriptions as stated in report/paper**	**Location in text or source** (pg & ¶/fig/table/other)
**Inclusion criteria**	Received their first prescription for reSET-O from their clinician<br>Consented to use<br>Redeemed their prescription<br>Completed at least one module	pg. 4 Demographics
**Exclusion criteria**		
**Total no. randomised** (or total pop. at start of study for NRCTs)	3,144	pg. 4 Demographics
**Clusters** (if applicable, no., type, no. people per cluster)	NA	
**Withdrawals and exclusions** (if not provided below by outcome)	873 individuals had missing data for the last 4 weeks of treatment	pg. 4 Substance Use/Abstinence
Intervention Groups	**Descriptions as stated in report/paper**	**Location in text or source** (pg & ¶/fig/table/other)
**Group name**	reSET-O	pg. 3 Methods
**No. randomised to group** (specify whether no. people or clusters)	3,144	pg. 4 Demographics
**Timing** (e.g. frequency, duration of each episode)	84 day treatment period<br>67 30 minute modules<br>4 modules per week	pg. 3 Introduction
**Co-interventions**	Routine Care	pg. 3 Methods
**Integrity of delivery**	Completion of one module was used as an inclusion criteria	pg. 4 Demographics
**Compliance**	49% of participants completed all core modules<br>55% used the app in the last week	pg. 4 Therapeutic Use/Patient Engagement
Outcomes	**Descriptions as stated in report/paper**	**Location in text or source** (pg & ¶/fig/table/other)
**Outcome name**	Abstinence in last 4 weeks of treatment	pg. 4 Methods
**Outcome definition** (with diagnostic criteria if relevant)(include name, time, and analysis method)	Abstinence in last 4 weeks of treatment ("To be deemed abstinent the patient must not have any positive UDS or self-reports during the last 4 weeks")<br>Summarized treatment group and did not use a statistical test to compare	pg. 4 Substance Use/Abstinence
**Imputation of missing data** (e.g. assumptions made for ITT analysis)	Missing data were excluded and treated as positive to calculate the bounds of abstinence.<br>"missing abstinence data for any given week was imputed in two different ways. The first approach was "missing data excluded (patients with no data as positive)," where weeks with no outcomes were excluded and participants were considered positive if any self report or urine drug screen was positive in the last 4 weeks or if all results were missing. The second approach was "missing data removed (patients with no data excluded)," where patients without any self-reports or urine drug screen results during the last 4 weeks were dropped from the analysis population."	pg. 4 Methods
**Power** (e.g. power & sample size calculation, level of power achieved)		

**Risk of Bias** (Based on Cochrane RoB Tool 2 and ROBINS-I)		Link to RoB Algorithm
**Primary Analysis RoB Assessment**		Notes
**Is the primary analysis evaluating effectiveness or efficacy?**	Effectiveness	
**Comparator**	No comparator	
**Outcome Being Assessed**	Abstinence in last 4 weeks of treatment	
**Specify the Numerical Result Being Assessed**	66% abstinent in last 4 weeks if patients with no data are positive<br>91% if patients with no data are excluded	
**Bias due to randomization (RCT only)**		Notes
1.1 Was the allocation sequence random?	NA	
1.2. Was the allocation sequence concealed until participants were enrolled and assigned to interventions?	NA	
1.3. Did baseline differences between intervention groups suggest a problem with the randomization process?	NA	
**Bias due to confounding**		Notes
1.1 Is there potential for confounding of the effect of intervention in this study?	Yes	
1.2. If Y/PY to 1.1 Was the analysis based on splitting participants' follow up time according to intervention received?	No	
1.3. If Y/PY to 1.2 Were intervention discontinuations or switches likely to be related to factors that are prognostic for the outcome?		
**Questions relating to baseline confounding only**		
1.4. If Y/PY to 1.1 Did the authors use an appropriate analysis method that controlled for all the important confounding domains?	No	The study collected limited demographic data so researchers couldn't account for confounders
1.5. If Y/PY to 1.4: Were confounding domains that were controlled for measured validly and reliably by the variables available in this study?		

1.6. Did the authors control for any post-intervention variables that could have been affected by the intervention?	No	
**Questions relating to baseline and time-varying confounding**		
1.7. If Y/PY to 1.3 Did the authors use an appropriate analysis method that controlled for all the important confounding domains and for time-varying confounding?		
1.8. If Y/PY to 1.7: Were confounding domains that were controlled for measured validly and reliably by the variables available in this study?		
**Bias in selection of participants into the study (Not evaluating for analyses of effectiveness)**		Notes
2.1. Was selection of participants into the analysis based on participant characteristics observed after the start of intervention?		
2.2. If Y/PY to 2.1: Were the post-intervention variables that influenced selection likely to be associated with intervention?		
2.3 If Y/PY to 2.2: Were the post-intervention variables that influenced selection likely to be influenced by the outcome or a cause of the outcome?		
2.4. Do start of follow-up and start of intervention coincide for most participants?		
2.5. If Y/PY to 2.2 and 2.3, or N/PN to 2.4: Were adjustment techniques used that are likely to correct for the presence of selection biases?		
**Bias in classification of interventions**		Notes
3.1 Were intervention groups clearly defined? Was the definition of adherence clearly defined?	Yes	
3.2 Was the information used to define intervention groups recorded at the start of the intervention?	No	The study was not preregistered
3.3 Could classification of intervention status have been affected by knowledge of the outcome or risk of the outcome?	Probably No	For inclusion into the study only one module was required to be completed
**Bias due to deviations from intended interventions**		Notes
**ROBINS-I**		
4.1. Were there deviations from the intended intervention beyond what would be expected in usual practice?	No	
4.2. If Y/PY to 4.1: Were these deviations from intended intervention unbalanced between groups and likely to have affected the outcome?		
4.3. Were important co-interventions balanced across intervention groups?	NA	
4.4. Was the intervention implemented successfully for most participants?	Yes	
4.5. Did study participants adhere to the assigned intervention regimen?	No	
4.6. If N/PN to 4.3, 4.4 or 4.5: Was an appropriate analysis used to estimate the effect of starting and adhering to the intervention?	No	The needed assumptions for the analysis method were not met
Risk of bias: Assignment to intervention	**Low** / Moderate / Serious / Critical / NI	
Risk of bias: Starting and adhering to intervention	Low / Moderate / Serious / **Critical** / NI	
**Risk of bias judgement**	Low / Moderate / Serious / **Critical** / NI	
**RoB Tool (Risk of bias due to deviations from the intended interventions (effect of adhering to intervention))**		
2.1. Were participants aware of their assigned intervention during the trial?	Yes	
2.2. Were carers and people delivering the interventions aware of participants' assigned intervention during the trial?	Yes	
2.3. If Y/PY/NI to 2.1 or 2.2: Were important non protocol interventions balanced across intervention groups?	NA	
2.4. [If applicable:] Were there failures in implementing the intervention that could have affected the outcome?	No	
2.5. [If applicable:] Was there non-adherence to the assigned intervention regimen that could have affected participants' outcomes?	Yes	
2.6. If N/PN/NI to 2.3, or Y/PY/NI to 2.4 or 2.5: Was an appropriate analysis used to estimate the effect of adhering to the intervention?	No	The needed assumptions for the analysis method were not met
**Risk of bias judgement**	Low/**High**/Some Concerns	
**Bias due to missing data**		Notes
5.1 Were outcome data available for all, or nearly all, participants?	No	Missing for 873 participants
5.2 Were participants excluded due to missing data on intervention status?	No	
5.3 Were participants excluded due to missing data on other variables needed for the analysis?	No	
5.4 If PN/N to 5.1, or Y/PY to 5.2 or 5.3: Are the proportion of participants and reasons for missing data similar across interventions?	NA	
5.5 If PN/N to 5.1, or Y/PY to 5.2 or 5.3: Is there evidence that results were robust to the presence of missing data?	Probably No	A sensitivity analysis was conducted where missing participants were considered positive or excluded. This gave a wide range for the potential abstinence rate of the sample
**Bias in measurement of outcomes**		Notes
6.1 Could the outcome measure have been influenced by knowledge of the intervention received?	No information	A mix of objective (drug screens) and subjective (self-reports) were used but it is unclear how often each were available
6.2 Were outcome assessors aware of the intervention received by study participants? (blinding of assessors)	Probably Yes	UDS measures were collected by participants clinics. Given that this is an observational study the clinicians were likely unaware of the participants participation in the study
6.3 Were the methods of outcome assessment comparable across intervention groups?	Probably No	While there was only one intervention group, there were many different schedules for collecting UDS information depending on the practice
6.4 Were any systematic errors in measurement of the outcome related to intervention received?	NA	
**Bias in selection of the reported result**		Notes
Were the data that produced this result analysed in accordance with a pre-specified analysis plan that was finalized before unblinded outcome data were available for analysis?	No	
Is the reported effect estimate likely to be selected, on the basis of the results, from...		
7.1. ... multiple outcome measurements within the outcome domain?	No	Only two measurements
7.2 ... multiple analyses of the intervention-outcome relationship?	Yes	Only summary statistics were reported but researchers could have originally tried another method of analysis
7.3 ... different subgroups?	No	All participants were included in the main analysis and a senstivity analysis was used to estimate effects of missing data
**Secondary Analysis RoB Assessment**		Notes
**Is the seconday analysis evaluating effectiveness or efficacy?**	Efficacy	
**Comparator**	No comparator	
**Outcome Being Assessed**	Abstinence for the participants who appropriately and consistently used the app in the first 4 weeks	
**Specify the Numerical Result Being Assessed**	"abstinence ("missing data excluded [patients with no data as positive]") was observed to be 88.1%"	
**Bias due to randomization (See primary analysis section)**		Notes
**Bias due to confounding**		Notes
1.1 Is there potential for confounding of the effect of intervention in this study?	Yes	Participants who completed more modules were likely more motivated to adhere to routine care as well
1.2. If Y/PY to 1.1 Was the analysis based on splitting participants' follow up time according to intervention received?		
1.3. If Y/PY to 1.2 Were intervention discontinuations or switches likely to be related to factors that are prognostic for the outcome?		
**Questions relating to baseline confounding only**		

1.4. If Y/PY to 1.1 Did the authors use an appropriate analysis method that controlled for all the important confounding domains?	No	
1.5. If Y/PY to 1.4: Were confounding domains that were controlled for measured validly and reliably by the variables available in this study?		
1.6. Did the authors control for any post-intervention variables that could have been affected by the intervention?	No	
**Questions relating to baseline and time-varying confounding**		
1.7. If Y/PY to 1.3 Did the authors use an appropriate analysis method that controlled for all the important confounding domains and for time-varying confounding?		
1.8. If Y/PY to 1.7: Were confounding domains that were controlled for measured validly and reliably by the variables available in this study?		
**Bias in selection of participants into the study (Not evaluating for analyses of effectiveness)**		Notes
2.1. Was selection of participants into the analysis based on participant characteristics observed after the start of intervention?	Probably No	This was not preregistered so it is probable that they chose the criteria based on their prescribed dosage but this cannot be confirmed<br>Only 29% of participants were included in this analysis
2.2. If Y/PY to 2.1: Were the post-intervention variables that influenced selection likely to be associated with intervention?		
2.3 If Y/PY to 2.2: Were the post-intervention variables that influenced selection likely to be influenced by the outcome or a cause of the outcome?		
2.4. Do start of follow-up and start of intervention coincide for most participants?	No Information	It is not clear when the trial started or ended
2.5. If Y/PY to 2.2 and 2.3, or N/PN to 2.4: Were adjustment techniques used that are likely to correct for the presence of selection biases?	No	No confounders were accounted for
**Bias in classification of interventions**		Notes
3.1 Were intervention groups clearly defined?	Yes	The definition of adherence was clearly defined due to the prescribed dose of the app
3.2 Was the information used to define intervention groups recorded at the start of the intervention?	No information	This study was not preregistered
3.3 Could classification of intervention status have been affected by knowledge of the outcome or risk of the outcome?	Yes	The analysis was not preregistered
**Bias due to deviations from intended interventions (See primary analysis section)**		Notes
**Bias due to missing data**		Notes
5.1 Were outcome data available for all, or nearly all, participants?	Probably Yes	
5.2 Were participants excluded due to missing data on intervention status?	No	
5.3 Were participants excluded due to missing data on other variables needed for the analysis?	No	They were considered to be non-abstinent
5.4 If PN/N to 5.1, or Y/PY to 5.2 or 5.3: Are the proportion of participants and reasons for missing data similar across interventions?	NA	
5.5 If PN/N to 5.1, or Y/PY to 5.2 or 5.3: Is there evidence that results were robust to the presence of missing data?	Yes	The missing data were consdidered positive for the analysis and the percent of abstinent people was still high
**Bias in measurement of outcomes**		Notes
6.1 Could the outcome measure have been influenced by knowledge of the intervention received?	No information	A mix of objective (drug screens) and subjective (self-reports) were used but it is unclear how often each were available
6.2 Were outcome assessors aware of the intervention received by study participants? (blinding of assessors)	Probably Yes	
6.3 Were the methods of outcome assessment comparable across intervention groups?	Yes	Methods were similar across adherence groups
6.4 Were any systematic errors in measurement of the outcome related to intervention received?	Probably No	While there was only one intervention group, there were many different schedules for collecting UDS information depending on the practice
6.5 Could adherence have been influenced by study participation?	No	
**Bias in selection of the reported result**		Notes
Were the data that produced this result analysed in accordance with a pre-specified analysis plan that was finalized before unblinded outcome data were available for analysis?	No	
Is the reported effect estimate likely to be selected, on the basis of the results, from...		
7.1. ... multiple outcome measurements within the outcome domain?	No	Only two measurements
7.2 ... multiple analyses of the intervention-outcome relationship?	Yes	Only summary statistics were reported but researchers could have originally tried another method of analysis
7.3 ... different subgroups?	Yes	They could have considered different subgroups of adherent and non-adherent

Article Key

Criteria for Adherence Metrics and Analysis		Notes
**Device name and indication for use**	ReSET-O (Cognitive Behavioral Therapy for Opioid use disorder)	
**Type of device** (Long-term vs short-term use)	Short-term use	
**Type of device** (Presence or absence of a prescribed dosage)	Presence of a prescribed dosage	
**Was adherence information collected?**	Yes	
**What information was collected about usage adherence?**	Active days<br>Modules completed	
**What information was collected about adherence to recommendations?**	NA	No explicit app recommendations
**Was information collected internally in the app?**	Yes	
**Was information modified internally?**	NA	
**Was initiation reported?**	Yes	
**What was average adherence?** (if reported)	100%	
**Was implementation reported?**	Yes	
**What was average adherence?** (if reported)	93% completed eight or more core modules, 85% completed at least half of core modules	
**Was persistence reported?**	Yes	
**What was average adherence?** (if reported)	93% after week 2 to 75% after week 12<br>64% completed all 32 core modules	
**Was adherence low?**	Yes	Less than 80% completed all core modules
**Was adherence not reported or low and could have affected outcomes?**	Yes	
**Was efficacy analyzed?**	Yes	
**What method was used?**	Dose-response	Compared the minimum modules completed in each of the first four weeks of treatment to absitnence in the last 4 weeks of treatment
**Were efficacy analyses preregistered?**	No	
**What assumptions are required for that method to study efficacy?**	SUTVA, positivity, consistency, conditional independence between adherence and outcomes	
**Did the article report evidence that the assumptions were met?**	No	
**SUTVA**	Yes	Therapy only delivered through the app
**Positivity**	No	No control condition
**Consistency** (treatment definition)	Yes	Clear definition of therapy
**Consistency** (adherence definition)		
**Exclusion restriction**		
**Strong Monotonicity**		
**Ignorability**	None	
**Overall Notes**		
**Cochrane CDPLG**		**Notes**
**Data form completed date** (dd/mm/yyyy)	3/30/2021	
**Study author contact details**	yuri.maricich@peartherapeutics.com	
Methods	**Descriptions as stated in report/paper**	**Location in text or source** (pg & ¶/fig/table/other)
Aim of study (e.g. efficacy, equivalence, pragmatic)	Effectiveness (Effectivess of "refill" prescription)	pg. 3 Introduction
Design (e.g. parallel, crossover, non-RCT)	Real-world Retrospective Observational evaluation	pg. 3 Methods
Unit of allocation (by individuals, cluster/ groups or body parts)	Individual	
Participants	**Descriptions as stated in report/paper**	**Location in text or source** (pg & ¶/fig/table/other)
**Inclusion criteria**	"patients under routine care by their clinician who filled an initial prescription for the reSET-O PDT between 1/1/19 and 12/31/20 (12-week cohort) and the subpopulation of these patients who were given a second 'refill' prescription with identical content (24-week cohort)."	pg. 3 Methods
**Exclusion criteria**		
**Total no. randomised** (or total pop. at start of study for NRCTs)	643	pg. 3 Methods
**Clusters** (if applicable, no., type, no. people per cluster)	NA	
**Withdrawals and exclusions** (if not provided below by outcome)	57 participants were missing UDS data	pg. 4 Figure 1
Intervention Groups	**Descriptions as stated in report/paper**	**Location in text or source** (pg & ¶/fig/table/other)
**Group name**	Refill cohort	pg. 4 Results
**No. randomised to group** (specify whether no. people or clusters)	643	pg. 4 Results
**Timing** (e.g. frequency, duration of each episode)	4 30 minute modules per week for 12 weeks	pg. 3 Methods
**Co-interventions**	Routine Care	pg. 3 Methods
**Integrity of delivery**	100% of participants used the app initially	pg. 5 Figure 4
**Compliance**	64% of participants completed all core modules	pg. 5 Figure 4
Outcomes	**Descriptions as stated in report/paper**	**Location in text or source** (pg & ¶/fig/table/other)
**Outcome name**	Abstinence in last 4 weeks of treatment (weeks 21-24)	pg. 3 Methods
**Outcome definition** (with diagnostic criteria if relevant)(include name, time, and analysis method)	Abstinence in last 4 weeks of treatment ("To be deemed abstinent the patient must not have any positive UDS or self-reports during the last 4 weeks" (from Maricich 2021a))<br>Summarized treatment group and did not use a statistical test to compare	pg. 6 Substance Use and Retention
**Imputation of missing data** (e.g. assumptions made for ITT analysis)	"Missing abstinence data for any given week was imputed either with a 'missing data positive' approach (i.e., patients with no data in the last 4 weeks were assumed to be positive) or with a 'missing data excluded' approach (i.e., patients with no data were excluded from analysis)"	pg. 3 Methods
**Power** (e.g. power & sample size calculation, level of power achieved)		
**Risk of Bias** (Based on Cochrane RoB Tool 2 and ROBINS-I)		Link to RoB Algorithm
**Primary Analysis RoB Assessment**		Notes
**Is the primary analysis evaluating effectiveness or efficacy?**	Effectiveness	
**Comparator**	No comparator	All plots were in comparison to the 12 week cohort
**Outcome Being Assessed**	Abstinence (i.e., no positive UDS or self-reports) during the last 4 weeks of the second PDT prescription period (weeks 21–24)	
**Specify the Numerical Result Being Assessed**	"In an analysis of abstinence in the last 4 weeks of treatment (i.e., weeks 21–24 of 24), 86% of patients were abstinent in an analysis where patients with no data are assumed to be positive, and 94% were abstinent in an analysis where 59 patients with no data were excluded (n = 584)."	
**Bias due to randomization (RCT only)**		Notes
1.1 Was the allocation sequence random?	NA	
1.2. Was the allocation sequence concealed until participants were enrolled and assigned to interventions?	NA	
1.3. Did baseline differences between intervention groups suggest a problem with the randomization process?	NA	
**Bias due to confounding**		Notes
1.1 Is there potential for confounding of the effect of intervention in this study?	Yes	
1.2. If Y/PY to 1.1 Was the analysis based on splitting participants' follow up time according to intervention received?	No	
1.3. If Y/PY to 1.2 Were intervention discontinuations or switches likely to be related to factors that are prognostic for the outcome?		
**Questions relating to baseline confounding only**		
1.4. If Y/PY to 1.1 Did the authors use an appropriate analysis method that controlled for all the important confounding domains?	No	The study collected limited demographic data so researchers couldn't account for confounders
1.5. If Y/PY to 1.4: Were confounding domains that were controlled for measured validly and reliably by the variables available in this study?		
1.6. Did the authors control for any post-intervention variables that could have been affected by the intervention?	No	
**Questions relating to baseline and time-varying confounding**		
1.7. If Y/PY to 1.3 Did the authors use an appropriate analysis method that controlled for all the important confounding domains and for time-varying confounding?		

1.8. If Y/PY to 1.7: Were confounding domains that were controlled for measured validly and reliably by the variables available in this study?		
**Bias in selection of participants into the study (Not evaluating for analyses of effectiveness)**		Notes
2.1. Was selection of participants into the analysis based on participant characteristics observed after the start of intervention?		
2.2. If Y/PY to 2.1: Were the post-intervention variables that influenced selection likely to be associated with intervention?		
2.3 If Y/PY to 2.2: Were the post-intervention variables that influenced selection likely to be influenced by the outcome or a cause of the outcome?		
2.4. Do start of follow-up and start of intervention coincide for most participants?		
2.5. If Y/PY to 2.2 and 2.3, or N/PN to 2.4: Were adjustment techniques used that are likely to correct for the presence of selection biases?		
**Bias in classification of interventions**		Notes
3.1 Were intervention groups clearly defined? Was the definition of adherence clearly defined?	Yes	
3.2 Was the information used to define intervention groups recorded at the start of the intervention?	No	The study was not preregistered
3.3 Could classification of intervention status have been affected by knowledge of the outcome or risk of the outcome?	Probably Yes	They created two cohorts and could have created groups for comparison after analyzing the results
**Bias due to deviations from intended interventions**		Notes
**ROBINS-I**		
4.1. Were there deviations from the intended intervention beyond what would be expected in usual practice?	No	
4.2. If Y/PY to 4.1: Were these deviations from intended intervention unbalanced between groups and likely to have affected the outcome?		
4.3. Were important co-interventions balanced across intervention groups?	NA	
4.4. Was the intervention implemented successfully for most participants?	Yes	
4.5. Did study participants adhere to the assigned intervention regimen?	No	68% of participants completed all core modules
4.6. If N/PN to 4.3, 4.4 or 4.5: Was an appropriate analysis used to estimate the effect of starting and adhering to the intervention?	No	The needed assumptions for the analysis method were not met
Risk of bias: Assignment to intervention	**Low** / Moderate / Serious / Critical / NI	
Risk of bias: Starting and adhering to intervention	Low / Moderate / Serious / **Critical** / NI	
**Risk of bias judgement**	Low / Moderate / Serious / **Critical** / NI	
**RoB Tool (Risk of bias due to deviations from the intended interventions (effect of adhering to intervention))**		
2.1. Were participants aware of their assigned intervention during the trial?	Yes	
2.2. Were carers and people delivering the interventions aware of participants' assigned intervention during the trial?	Yes	
2.3. If Y/PY/NI to 2.1 or 2.2: Were important non protocol interventions balanced across intervention groups?	NA	
2.4. [If applicable:] Were there failures in implementing the intervention that could have affected the outcome?	No	
2.5. [If applicable:] Was there non-adherence to the assigned intervention regimen that could have affected participants' outcomes?	Yes	68% of participants completed all core modules
2.6. If N/PN/NI to 2.3, or Y/PY/NI to 2.4 or 2.5: Was an appropriate analysis used to estimate the effect of adhering to the intervention?	No	The needed assumptions for the analysis method were not met
**Risk of bias judgement**	Low/**High**/Some Concerns	
**Bias due to missing data**		Notes
5.1 Were outcome data available for all, or nearly all, participants?	Yes	59/634 participants were missing outcomes
5.2 Were participants excluded due to missing data on intervention status?	No	
5.3 Were participants excluded due to missing data on other variables needed for the analysis?	No	
5.4 If PN/N to 5.1, or Y/PY to 5.2 or 5.3: Are the proportion of participants and reasons for missing data similar across interventions?		
5.5 If PN/N to 5.1, or Y/PY to 5.2 or 5.3: Is there evidence that results were robust to the presence of missing data?		Participants with missing data were considered positive as part of a sensitivity analysis
**Bias in measurement of outcomes**		Notes
6.1 Could the outcome measure have been influenced by knowledge of the intervention received?	Probably No	A mix of objective (drug screens) and subjective (self-reports) were used but it is unclear how often each were available
6.2 Were outcome assessors aware of the intervention received by study participants? (blinding of assessors)	Yes	
6.3 Were the methods of outcome assessment comparable across intervention groups?	NA	
6.4 Were any systematic errors in measurement of the outcome related to intervention received?	No	
**Bias in selection of the reported result**		Notes
Were the data that produced this result analysed in accordance with a pre-specified analysis plan that was finalized before unblinded outcome data were available for analysis?	No	
Is the reported effect estimate likely to be selected, on the basis of the results, from...		
7.1. ... multiple outcome measurements within the outcome domain?	Probably No	Only two measurements with one primary measure that was used in a previous study
7.2 ... multiple analyses of the intervention-outcome relationship?	Probably Yes	Only summary statistics were reported but researchers could have originally tried another method of analysis
7.3 ... different subgroups?	Probably No	All participants were included in the main analysis and a senstivity analysis was used to estimate effects of missing data
**Secondary Analysis RoB Assessment**		Notes
**Is the seconday analysis evaluating effectiveness or efficacy?**	Efficacy	
**Comparator**	No comparator	
**Outcome Being Assessed**	Abstinence for the participants who appropriately and consistently used the app in the first 4 weeks	
**Specify the Numerical Result Being Assessed**	"Correlation was observed between minimum number of modules completed each week during the first 4 weeks of the second prescription (weeks 13–16) and retention in treatment (Figure 8), with modestly higher levels again observed in the 24-week cohort compared to the 12-week cohort."	
**Bias due to randomization (See primary analysis section)**		Notes
**Bias due to confounding**		Notes
1.1 Is there potential for confounding of the effect of intervention in this study?	Yes	Participants who completed more modules were likely more motivated to adhere to routine care as well
1.2. If Y/PY to 1.1 Was the analysis based on splitting participants' follow up time according to intervention received?		
1.3. If Y/PY to 1.2 Were intervention discontinuations or switches likely to be related to factors that are prognostic for the outcome?		
**Questions relating to baseline confounding only**		
1.4. If Y/PY to 1.1 Did the authors use an appropriate analysis method that controlled for all the important confounding domains?	No	
1.5. If Y/PY to 1.4: Were confounding domains that were controlled for measured validly and reliably by the variables available in this study?		
1.6. Did the authors control for any post-intervention variables that could have been affected by the intervention?	No	
**Questions relating to baseline and time-varying confounding**		

1.7. If Y/PY to 1.3 Did the authors use an appropriate analysis method that controlled for all the important confounding domains and for time-varying confounding?		
1.8. If Y/PY to 1.7: Were confounding domains that were controlled for measured validly and reliably by the variables available in this study?		
**Bias in selection of participants into the study (Not evaluating for analyses of effectiveness)**		Notes
2.1. Was selection of participants into the analysis based on participant characteristics observed after the start of intervention?	No	
2.2. If Y/PY to 2.1: Were the post-intervention variables that influenced selection likely to be associated with intervention?		
2.3 If Y/PY to 2.2: Were the post-intervention variables that influenced selection likely to be influenced by the outcome or a cause of the outcome?		
2.4. Do start of follow-up and start of intervention coincide for most participants?	No Information	It is not clear when the trial started or ended
2.5. If Y/PY to 2.2 and 2.3, or N/PN to 2.4: Were adjustment techniques used that are likely to correct for the presence of selection biases?		
**Bias in classification of interventions**		Notes
3.1 Were intervention groups clearly defined?	Yes	The definition of adherence was clearly defined due to the prescribed dose of the app
3.2 Was the information used to define intervention groups recorded at the start of the intervention?	No information	This study was not preregistered
3.3 Could classification of intervention status have been affected by knowledge of the outcome or risk of the outcome?	Probably No	
**Bias due to deviations from intended interventions (See primary analysis section)**		Notes
**Bias due to missing data**		Notes
5.1 Were outcome data available for all, or nearly all, participants?	Yes	
5.2 Were participants excluded due to missing data on intervention status?	No	
5.3 Were participants excluded due to missing data on other variables needed for the analysis?		
5.4 If PN/N to 5.1, or Y/PY to 5.2 or 5.3: Are the proportion of participants and reasons for missing data similar across interventions?		
5.5 If PN/N to 5.1, or Y/PY to 5.2 or 5.3: Is there evidence that results were robust to the presence of missing data?		
**Bias in measurement of outcomes**		Notes
6.1 Could the outcome measure have been influenced by knowledge of the intervention received?	Probably No	A mix of objective (drug screens) and subjective (self-reports) were used but it is unclear how often each were available
6.2 Were outcome assessors aware of the intervention received by study participants? (blinding of assessors)	Yes	
6.3 Were the methods of outcome assessment comparable across intervention groups?	No information	It is possible that less adherent participants were more likely to have missing data leading to a lower likelihood of abstinence
6.4 Were any systematic errors in measurement of the outcome related to intervention received?	No	
6.5 Could adherence have been influenced by study participation?	No	
**Bias in selection of the reported result**		Notes
Were the data that produced this result analysed in accordance with a pre-specified analysis plan that was finalized before unblinded outcome data were available for analysis?	No	
Is the reported effect estimate likely to be selected, on the basis of the results, from...		
7.1. ... multiple outcome measurements within the outcome domain?	No	Only two measurements with one primary measure that was used in a previous study
7.2 ... multiple analyses of the intervention-outcome relationship?	Yes	Researchers didn't report the correlation and just said there was correlation according to the figure
7.3 ... different subgroups?	Probably No	Researchers didn't preregister the groups but the groups have a natural meaning

Article Key

Criteria for Adherence Metrics and Analysis		Notes
**Device name and indication for use**	ReSET-O (Cognitive Behavioral Therapy for Opioid use disorder)	
**Type of device** (Long-term vs short-term use)	Short-term use	
**Type of device** (Presence or absence of a prescribed dosage)	Presence of a prescribed dosage	
**Was adherence information collected?**	Yes	
**What information was collected about usage adherence?**	module completion	
**What information was collected about adherence to recommendations?**	NA	No explicit app recommendations
**Was information collected internally in the app?**	Yes	
**Was information modified internally?**	NA	
**Was initiation reported?**	Yes	
**What was average adherence?** (if reported)	Reported that the range was of module completion was 4-150	
**Was implementation reported?**	Yes	
**What was average adherence?** (if reported)	a mean of 77.3 modules completed (SD = 32.36; range = 4–150)<br>Mean of 42.2 core modules completed (SD = 15.31; range = 4-78)	There are 48 core modules and 67 total modules according to their website and previous studies
**Was persistence reported?**	No	They measured the percent of participants remaining in treatment but not the percent of participants still using the app
**What was average adherence?** (if reported)		
**Was adherence low?**	No	Over 80% of core modules were completed on average<br>Over 100% of all modules were completed on average
**Was adherence not reported or low and could have affected outcomes?**	No	
**Was efficacy analyzed?**	No	
**What method was used?**	NA	
**Were efficacy analyses preregistered?**		
**What assumptions are required for that method to study efficacy?**		
**Did the article report evidence that the assumptions were met?**		
**SUTVA**		
**Positivity**		
**Consistency** (treatment definition)		
**Consistency** (adherence definition)		
**Exclusion restriction**		
**Strong Monotonicity**		
**Ignorability**		
**Overall Notes**		
**Cochrane CDPLG**		**Notes**
**Data form completed date** (dd/mm/yyyy)	3/30/2022	
**Study author contact details**	hilary@peartherapeutics.com	
Methods	**Descriptions as stated in report/paper**	**Location in text or source** (pg & ¶/fig/table/other)
Aim of study (e.g. efficacy, equivalence, pragmatic)	Effectiveness	pg. 7 Limitations
Design (e.g. parallel, crossover, non-RCT)	Randomized Controlled Trial	pg. 2 Abstract
Unit of allocation (by individuals, cluster/ groups or body parts)	Individual	
Participants	**Descriptions as stated in report/paper**	**Location in text or source** (pg & ¶/fig/table/other)
**Inclusion criteria**	"At least 18 years old, in good health, met DSM-IV criteria for opioid dependence, qualified for buprenorphine treatment, had no active psychiatric disorder, no unstable or significant medical illness, were not pregnant, and were not incarcerated."	pg. 3 Participants and Setting
**Exclusion criteria**		
**Total no. randomised** (or total pop. at start of study for NRCTs)	170	pg. 3 Participants and Setting
**Clusters** (if applicable, no., type, no. people per cluster)	NA	
**Withdrawals and exclusions** (if not provided below by outcome)	25 participants dropped in the TAU group and 16 participants dropped in the TAU + DT group	pg. 5 Figure 1
Intervention Groups	**Descriptions as stated in report/paper**	**Location in text or source** (pg & ¶/fig/table/other)
**Group name**	TAU + Digital Therapeutic	pg. 3 TAU Plus Digital Therapeutic
**No. randomised to group** (specify whether no. people or clusters)	91	pg. 4 Table 1
**Timing** (e.g. frequency, duration of each episode)	TAU and 30 minute module three times per week without clinician visit and twice per week with a clinician visit	pg. 3 TAU Plus Digital Therapeutic
**Co-interventions**		
**Integrity of delivery**	No information	
**Compliance**	18% of participants dropped out in the TAU+digital therapeutic group	pg. 5 Figure 1
Intervention Groups	**Descriptions as stated in report/paper**	**Location in text or source** (pg & ¶/fig/table/other)
**Group name**	TAU	pg. 3 Treatment-as-Usual
**No. randomised to group** (specify whether no. people or clusters)	79	pg. 4 Table 1
**Timing** (e.g. frequency, duration of each episode)	Buprenorphine/naloxone and every other week 30 minute meeting with a clinician	pg. 3 Treatment-as-Usual
**Co-interventions**		
**Integrity of delivery**	No information	
**Compliance**	32% of participants dropped out in the TAU group	pg. 5 Figure 1
Outcomes	**Descriptions as stated in report/paper**	**Location in text or source** (pg & ¶/fig/table/other)
**Outcome name**	Abstinence during last 4 weeks of treatment	pg. 4 Outcomes
**Outcome definition** (with diagnostic criteria if relevant)(include name, time, and analysis method)	Abstinence during last 4 weeks of treatment analyzed with a repeated measures logistic generalized estimating equations model with factors for treatment, time, and treatment x time	pg. 4 Statistical Analysis
**Imputation of missing data** (e.g. assumptions made for ITT analysis)	Missing data were considered non-abstinent	pg. 4 Outcomes
**Power** (e.g. power & sample size calculation, level of power achieved)		
**Risk of Bias** (Based on Cochrane RoB Tool 2 and ROBINS-I)		Link to RoB Algorithm
**Primary Analysis RoB Assessment**		Notes
**Is the primary analysis evaluating effectiveness or efficacy?**	Effectiveness	
**Comparator**	TAU + Digital Therapeutic vs TAU	
**Outcome Being Assessed**	Abstinence in last 4 weeks of treatment	
**Specify the Numerical Result Being Assessed**	Table 2<br>Primary endpoint (weeks 9–12)<br>Total abstinence from opioids and cocaine 60.6% (TAU) 75.9% (TAU +digital therapeutic) OR =2.05 (1.07, 3.90) p= .03<br>Abstinence from opioids only 62.1% (TAU) 77.3% (TAU +digital therapeutic) OR = 2.08 (1.10, 3.95) p=.02<br>Abstinence from cocaine only 64.5% (TAU) 82.4% (TAU +digital therapeutic) OR = 2.58 (1.37, 4.86) p=.003	
**Bias due to randomization (RCT only)**		Notes
1.1 Was the allocation sequence random?	Yes	
1.2. Was the allocation sequence concealed until participants were enrolled and assigned to interventions?	Yes	
1.3. Did baseline differences between intervention groups suggest a problem with the randomization process?	No	
**Bias due to confounding**		Notes
1.1 Is there potential for confounding of the effect of intervention in this study?	No	RCT without any indication of unbalanced groups
1.2. If Y/PY to 1.1 Was the analysis based on splitting participants' follow up time according to intervention received?		
1.3. If Y/PY to 1.2 Were intervention discontinuations or switches likely to be related to factors that are prognostic for the outcome?		

**Questions relating to baseline confounding only**		
1.4. If Y/PY to 1.1 Did the authors use an appropriate analysis method that controlled for all the important confounding domains?		
1.5. If Y/PY to 1.4: Were confounding domains that were controlled for measured validly and reliably by the variables available in this study?		
1.6. Did the authors control for any post-intervention variables that could have been affected by the intervention?	No	
**Questions relating to baseline and time-varying confounding**		
1.7. If Y/PY to 1.3 Did the authors use an appropriate analysis method that controlled for all the important confounding domains and for time-varying confounding?		
1.8. If Y/PY to 1.7: Were confounding domains that were controlled for measured validly and reliably by the variables available in this study?		
**Bias in selection of participants into the study (Not evaluating for analyses of effectiveness)**		Notes
2.1. Was selection of participants into the analysis based on participant characteristics observed after the start of intervention?		
2.2. If Y/PY to 2.1: Were the post-intervention variables that influenced selection likely to be associated with intervention?		
2.3 If Y/PY to 2.2: Were the post-intervention variables that influenced selection likely to be influenced by the outcome or a cause of the outcome?		
2.4. Do start of follow-up and start of intervention coincide for most participants?		
2.5. If Y/PY to 2.2 and 2.3, or N/PN to 2.4: Were adjustment techniques used that are likely to correct for the presence of selection biases?		
**Bias in classification of interventions**		Notes
3.1 Were intervention groups clearly defined? Was the definition of adherence clearly defined?	Yes	
3.2 Was the information used to define intervention groups recorded at the start of the intervention?	Yes	Treatment arm definitions were preregistered on clinicaltrials.gov
3.3 Could classification of intervention status have been affected by knowledge of the outcome or risk of the outcome?	No	
**Bias due to deviations from intended interventions**		Notes
**ROBINS-I**		
4.1. Were there deviations from the intended intervention beyond what would be expected in usual practice?	No	
4.2. If Y/PY to 4.1: Were these deviations from intended intervention unbalanced between groups and likely to have affected the outcome?		
4.3. Were important co-interventions balanced across intervention groups?	Yes	
4.4. Was the intervention implemented successfully for most participants?	Yes	
4.5. Did study participants adhere to the assigned intervention regimen?	Yes	On average 77.3 modules were completed (SD= 32.36; range = 4–150)) out of 67 total modules
4.6. If N/PN to 4.3, 4.4 or 4.5: Was an appropriate analysis used to estimate the effect of starting and adhering to the intervention?		
Risk of bias: Assignment to intervention	**Low** / Moderate / Serious / Critical / NI	
Risk of bias: Starting and adhering to intervention	**Low** / Moderate / Serious / Critical / NI	
**Risk of bias judgement**	**Low** / Moderate / Serious / Critical / NI	
**RoB Tool (Risk of bias due to deviations from the intended interventions (effect of adhering to intervention))**		
2.1. Were participants aware of their assigned intervention during the trial?	Yes	
2.2. Were carers and people delivering the interventions aware of participants' assigned intervention during the trial?	Yes	
2.3. If Y/PY/NI to 2.1 or 2.2: Were important non protocol interventions balanced across intervention groups?	Yes	
2.4. [If applicable:] Were there failures in implementing the intervention that could have affected the outcome?	No	
2.5. [If applicable:] Was there non-adherence to the assigned intervention regimen that could have affected participants' outcomes?	Probably No	On average 77.3 modules were completed (SD= 32.36; range = 4–150)) out of 67 total modules
2.6. If N/PN/NI to 2.3, or Y/PY/NI to 2.4 or 2.5: Was an appropriate analysis used to estimate the effect of adhering to the intervention?		
**Risk of bias judgement**	**Low**/High/Some Concerns	
**Bias due to missing data**		Notes
5.1 Were outcome data available for all, or nearly all, participants?	No	
5.2 Were participants excluded due to missing data on intervention status?	No	
5.3 Were participants excluded due to missing data on other variables needed for the analysis?	No	
5.4 If PN/N to 5.1, or Y/PY to 5.2 or 5.3: Are the proportion of participants and reasons for missing data similar across interventions?	No	
5.5 If PN/N to 5.1, or Y/PY to 5.2 or 5.3: Is there evidence that results were robust to the presence of missing data?	No	Researchers considered missing data as non-abstinent and did not perform a sensitivity analysis<br><br>And the paper reports that it is common practice to consider missing data as non-abstinent for this treatment area (however positive UDS imputation procedures can show a slightly inflated treatment effect at times)
**Bias in measurement of outcomes**		Notes
6.1 Could the outcome measure have been influenced by knowledge of the intervention received?	No	Objective measurement
6.2 Were outcome assessors aware of the intervention received by study participants? (blinding of assessors)	Yes	
6.3 Were the methods of outcome assessment comparable across intervention groups?	Yes	
6.4 Were any systematic errors in measurement of the outcome related to intervention received?	No	
**Bias in selection of the reported result**		Notes
Were the data that produced this result analysed in accordance with a pre-specified analysis plan that was finalized before unblinded outcome data were available for analysis?	Yes	Secondary analysis of preregistered trial
Is the reported effect estimate likely to be selected, on the basis of the results, from...		
7.1. ... multiple outcome measurements within the outcome domain?	No	
7.2 ... multiple analyses of the intervention-outcome relationship?	Probably No	This analysis is based on new NIDA recommendations
7.3 ... different subgroups?	No	
**Secondary Analysis RoB Assessment**		Notes
**Is the seconday analysis evaluating effectiveness or efficacy?**		
**Comparator**		
**Outcome Being Assessed**		
**Specify the Numerical Result Being Assessed**		
**Bias due to randomization (See primary analysis section)**		Notes
**Bias due to confounding**		Notes
1.1 Is there potential for confounding of the effect of intervention in this study?		
1.2. If Y/PY to 1.1 Was the analysis based on splitting participants' follow up time according to intervention received?		
1.3. If Y/PY to 1.2 Were intervention discontinuations or switches likely to be related to factors that are prognostic for the outcome?		
**Questions relating to baseline confounding only**		
1.4. If Y/PY to 1.1 Did the authors use an appropriate analysis method that controlled for all the important confounding domains?		

1.5. If Y/PY to 1.4: Were confounding domains that were controlled for measured validly and reliably by the variables available in this study?		
1.6. Did the authors control for any post-intervention variables that could have been affected by the intervention?		
**Questions relating to baseline and time-varying confounding**		
1.7. If Y/PY to 1.3 Did the authors use an appropriate analysis method that controlled for all the important confounding domains and for time-varying confounding?		
1.8. If Y/PY to 1.7: Were confounding domains that were controlled for measured validly and reliably by the variables available in this study?		
**Bias in selection of participants into the study (Not evaluating for analyses of effectiveness)**		Notes
2.1. Was selection of participants into the analysis based on participant characteristics observed after the start of intervention?		
2.2. If Y/PY to 2.1: Were the post-intervention variables that influenced selection likely to be associated with intervention?		
2.3 If Y/PY to 2.2: Were the post-intervention variables that influenced selection likely to be influenced by the outcome or a cause of the outcome?		
2.4. Do start of follow-up and start of intervention coincide for most participants?		
2.5. If Y/PY to 2.2 and 2.3, or N/PN to 2.4: Were adjustment techniques used that are likely to correct for the presence of selection biases?		
**Bias in classification of interventions**		Notes
3.1 Were intervention groups clearly defined?		
3.2 Was the information used to define intervention groups recorded at the start of the intervention?		
3.3 Could classification of intervention status have been affected by knowledge of the outcome or risk of the outcome?		
**Bias due to deviations from intended interventions (See primary analysis section)**		Notes
**Bias due to missing data**		Notes
5.1 Were outcome data available for all, or nearly all, participants?		
5.2 Were participants excluded due to missing data on intervention status?		
5.3 Were participants excluded due to missing data on other variables needed for the analysis?		
5.4 If PN/N to 5.1, or Y/PY to 5.2 or 5.3: Are the proportion of participants and reasons for missing data similar across interventions?		
5.5 If PN/N to 5.1, or Y/PY to 5.2 or 5.3: Is there evidence that results were robust to the presence of missing data?		
**Bias in measurement of outcomes**		Notes
6.1 Could the outcome measure have been influenced by knowledge of the intervention received?		
6.2 Were outcome assessors aware of the intervention received by study participants? (blinding of assessors)		
6.3 Were the methods of outcome assessment comparable across intervention groups?		
6.4 Were any systematic errors in measurement of the outcome related to intervention received?		
6.5 Could adherence have been influenced by study participation?		
**Bias in selection of the reported result**		Notes
Were the data that produced this result analysed in accordance with a pre-specified analysis plan that was finalized before unblinded outcome data were available for analysis?		
Is the reported effect estimate likely to be selected, on the basis of the results, from...		
7.1. ... multiple outcome measurements within the outcome domain?		
7.2 ... multiple analyses of the intervention-outcome relationship?		
7.3 ... different subgroups?		

Criteria for Adherence Metrics and Analysis		Notes
**Device name and indication for use**	Natural Cycles (contraception)	
**Type of device** (Long-term vs short-term use)	Long-term use	
**Type of device** (Presence or absence of a prescribed dosage)	Presence of a prescribed dose	
**Was adherence information collected?**	Yes	
**What information was collected about usage adherence?**	Percentage of days with temperature logging	
**What information was collected about adherence to recommendations?**	Intercourse logging and backup contraception use on red days	
**Was information collected internally in the app?**	Yes	
**Was information modified internally?**	NA	
**Was initiation reported?**	Yes	Users needed to log at least 20 days of data
**What was average adherence?** (if reported)	100%	
**Was implementation reported?**	Yes	
**What was average adherence?** (if reported)	55.1% of users recorded temperature less than 50% of the time; 25% 50-70% of the time; 19.9% over 70% of the time<br>23% of red day intercourse was unprotected	
**Was persistence reported?**	Yes	
**What was average adherence?** (if reported)	41.9% (5137) users were still actively using the app at the end of the study period; The percentage of women retained after 13 cycles was 35.7%	
**Was adherence low?**	Yes	Most users recorded their temperature less than 50% of the time. Even if the 19.9% of participants logged their temperature 100% of the time, on average all participants would have logged their temperature 64% of the time
**Was adherence not reported or low and could have affected outcomes?**	Yes	
**Was efficacy analyzed?**	Yes	
**What method was used?**	Per-protocol analysis	Pearl index for perfect-use cycles
**Were efficacy analyses preregistered?**	No	
**What assumptions are required for that method to study efficacy?**	SUTVA, positivity, consistency, ignorability, conditional independence of adherence and outcomes	
**Did the article report evidence that the assumptions were met?**	No	
**SUTVA**	Yes	Treatment only through the app
**Positivity**	No	No control condition
**Consistency** (treatment definition)	Yes	Clear treatment definition
**Consistency** (adherence definition)	Yes	Clear adherence definition
**Exclusion restriction**		
**Strong Monotonicity**		
**Ignorability**	None	
**Overall Notes**		
**Cochrane CDPLG**		**Notes**
**Data form completed date** (dd/mm/yyyy)	3/29/2022	
**Study author contact details**	raoul@naturalcycles.com	
Methods	**Descriptions as stated in report/paper**	**Location in text or source** (pg & ¶/fig/table/other)
Aim of study (e.g. efficacy, equivalence, pragmatic)	Effectiveness	pg. 3 Introduction
Design (e.g. parallel, crossover, non-RCT)	Prospecitve Observational Cohort Study	pg. 4 Study Design
Unit of allocation (by individuals, cluster/ groups or body parts)	Individual	
Participants	**Descriptions as stated in report/paper**	**Location in text or source** (pg & ¶/fig/table/other)
**Inclusion criteria**	"women using the Natural Cycles app as their primary means of contraception who had registered as a paying user on an annual subscription between September 1st 2017 and 1st April 2018 and were therefore new users. Users had to have entered at least 20 daily data entries, have registered with the app in the UK, and were aged 18–45"	pg. Study Design
**Exclusion criteria**		
**Total no. randomised** (or total pop. at start of study for NRCTs)	12247	pg. 5 Study Population
**Clusters** (if applicable, no., type, no. people per cluster)	NA	
**Withdrawals and exclusions** (if not provided below by outcome)		
Intervention Groups	**Descriptions as stated in report/paper**	**Location in text or source** (pg & ¶/fig/table/other)
**Group name**	Natural Cycles App	pg. 4 Study Design
**No. randomised to group** (specify whether no. people or clusters)	12247	pg. 5 Study Population
**Timing** (e.g. frequency, duration of each episode)	Daily temperature reading every morning<br>Optional LH tests<br>Abstain from sex or use alternative contraceptive method on red days	pg. 3 Introduction
**Co-interventions**	Alternative contraceptives on red days	pg. 3 Introduction
**Integrity of delivery**	All participants had to provide at least 20 days of data to meet the study's inclusion criteria	pg. 4 Study Design
**Compliance**	19.9% of users had a measuring frequency greater than 70%	pg. 5 Intercourse Logging and Percentage Green
Outcomes	**Descriptions as stated in report/paper**	**Location in text or source** (pg & ¶/fig/table/other)
**Outcome name**	Typical use effectiveness	pg. 4 Data Analysis
**Outcome definition** (with diagnostic criteria if relevant)(include name, time, and analysis method)	Typical use effectiveness analyzed using life table analysis and pearl index	pg. 4 Data Analysis
**Imputation of missing data** (e.g. assumptions made for ITT analysis)	Cycles without any data were excluded from analysis<br>Participants were classified as likely, possibly or unlikely pregnant based on ovulation schedule and temperature readings	pg. 4 Determining Pregnancy and Discontinuation
**Power** (e.g. power & sample size calculation, level of power achieved)		
Outcomes	**Descriptions as stated in report/paper**	**Location in text or source** (pg & ¶/fig/table/other)
**Outcome name**	Perfect use effectiveness	pg. 4 Data Analysis
**Outcome definition** (with diagnostic criteria if relevant)(include name, time, and analysis method)	Pearl index for perfect use cycles	pg. 4 Data Analysis
**Imputation of missing data** (e.g. assumptions made for ITT analysis)	No missing data for perfect use cycles	pg. 4 Determining Pregnancy and Discontinuation
**Power** (e.g. power & sample size calculation, level of power achieved)		
**Risk of Bias** (Based on Cochrane RoB Tool 2 and ROBINS-I)		Link to RoB Algorithm
**Primary Analysis RoB Assessment**		Notes
**Is the primary analysis evaluating effectiveness or efficacy?**	Effectiveness	
**Comparator**	No comparator	
**Outcome Being Assessed**	13-cycle cumulative pregnancy probability	
**Specify the Numerical Result Being Assessed**	13-cycle cumulative pregnancy probability of 7.1% (95% CI: 6.5, 7.7)	
**Bias due to randomization (RCT only)**		Notes
1.1 Was the allocation sequence random?	NA	
1.2. Was the allocation sequence concealed until participants were enrolled and assigned to interventions?	NA	
1.3. Did baseline differences between intervention groups suggest a problem with the randomization process?	NA	
**Bias due to confounding**		Notes
1.1 Is there potential for confounding of the effect of intervention in this study?	Yes	In additional to other possible confounders, "users that had recently discontinued hormonal contraceptives, had diagnosed infertility or were breastfeeding were also included in the analysis"
1.2. If Y/PY to 1.1 Was the analysis based on splitting participants' follow up time according to intervention received?	No	
1.3. If Y/PY to 1.2 Were intervention discontinuations or switches likely to be related to factors that are prognostic for the outcome?		

Article Key

**Questions relating to baseline confounding only**		
1.4. If Y/PY to 1.1 Did the authors use an appropriate analysis method that controlled for all the important confounding domains?	No	
1.5. If Y/PY to 1.4: Were confounding domains that were controlled for measured validly and reliably by the variables available in this study?		
1.6. Did the authors control for any post-intervention variables that could have been affected by the intervention?	No	
**Questions relating to baseline and time-varying confounding**		
1.7. If Y/PY to 1.3 Did the authors use an appropriate analysis method that controlled for all the important confounding domains and for time-varying confounding?		
1.8. If Y/PY to 1.7: Were confounding domains that were controlled for measured validly and reliably by the variables available in this study?		
**Bias in selection of participants into the study (Not evaluating for analyses of effectiveness)**		Notes
2.1. Was selection of participants into the analysis based on participant characteristics observed after the start of intervention?		Users could have been more likely to be interested in pregnancy since the app can switch to a mode for planning pregnancies and conception
2.2. If Y/PY to 2.1: Were the post-intervention variables that influenced selection likely to be associated with intervention?		
2.3 If Y/PY to 2.2: Were the post-intervention variables that influenced selection likely to be influenced by the outcome or a cause of the outcome?		
2.4. Do start of follow-up and start of intervention coincide for most participants?		
2.5. If Y/PY to 2.2 and 2.3, or N/PN to 2.4: Were adjustment techniques used that are likely to correct for the presence of selection biases?		
**Bias in classification of interventions**		Notes
3.1 Were intervention groups clearly defined? Was the definition of adherence clearly defined?	Yes	
3.2 Was the information used to define intervention groups recorded at the start of the intervention?	No	This study was not preregistered
3.3 Could classification of intervention status have been affected by knowledge of the outcome or risk of the outcome?	Probably No	Only one cohort was defined in this study and the inclusion criteria match prior studies
**Bias due to deviations from intended interventions**		Notes
**ROBINS-I**		
4.1. Were there deviations from the intended intervention beyond what would be expected in usual practice?	No	
4.2. If Y/PY to 4.1: Were these deviations from intended intervention unbalanced between groups and likely to have affected the outcome?	NA	
4.3. Were important co-interventions balanced across intervention groups?	NA	
4.4. Was the intervention implemented successfully for most participants?	Yes	Users needed to log at least 20 days of data
4.5. Did study participants adhere to the assigned intervention regimen?	No	Most users recorded their temperature less than 50% of the time. Even if the 19.9% of participants logged their temperature 100% of the time, on average all participants would have logged their temperature 64% of the time
4.6. If N/PN to 4.3, 4.4 or 4.5: Was an appropriate analysis used to estimate the effect of starting and adhering to the intervention?	No	The required assumptions for this analysis method were not met
Risk of bias: Assignment to intervention	**Low** / Moderate / Serious / Critical / NI	
Risk of bias: Starting and adhering to intervention	Low / Moderate / Serious / **Critical** / NI	
**Risk of bias judgement**	Low / Moderate / Serious / **Critical** / NI	
**RoB Tool (Risk of bias due to deviations from the intended interventions (effect of adhering to intervention))**		
2.1. Were participants aware of their assigned intervention during the trial?	Yes	
2.2. Were carers and people delivering the interventions aware of participants' assigned intervention during the trial?	NA	
2.3. If Y/PY/NI to 2.1 or 2.2: Were important non protocol interventions balanced across intervention groups?	NA	
2.4. [If applicable:] Were there failures in implementing the intervention that could have affected the outcome?	No	Users needed to log at least 20 days of data
2.5. [If applicable:] Was there non-adherence to the assigned intervention regimen that could have affected participants' outcomes?	Yes	Most users recorded their temperature less than 50% of the time. Even if the 19.9% of participants logged their temperature 100% of the time, on average all participants would have logged their temperature 64% of the time
2.6. If N/PN/NI to 2.3, or Y/PY/NI to 2.4 or 2.5: Was an appropriate analysis used to estimate the effect of adhering to the intervention?	No	The required assumptions for this analysis method were not met
**Risk of bias judgement**	Low/**High**/Some Concerns	
**Bias due to missing data**		Notes
5.1 Were outcome data available for all, or nearly all, participants?	No	Outcomes were missing for 35% of participants
5.2 Were participants excluded due to missing data on intervention status?	No	
5.3 Were participants excluded due to missing data on other variables needed for the analysis?	No	Users empty cycles were excluded<br>Participants were classified as likely, possibly or unlikely pregnant based on ovulation schedule and temperature readings
5.4 If PN/N to 5.1, or Y/PY to 5.2 or 5.3: Are the proportion of participants and reasons for missing data similar across interventions?	NA	
5.5 If PN/N to 5.1, or Y/PY to 5.2 or 5.3: Is there evidence that results were robust to the presence of missing data?	No information	
**Bias in measurement of outcomes**		Notes
6.1 Could the outcome measure have been influenced by knowledge of the intervention received?	Probably No	
6.2 Were outcome assessors aware of the intervention received by study participants? (blinding of assessors)	Yes	
6.3 Were the methods of outcome assessment comparable across intervention groups?	NA	
6.4 Were any systematic errors in measurement of the outcome related to intervention received?	Yes	They classified pregnancies using a similar algorithm to how they predict red days in the app
**Bias in selection of the reported result**		Notes
Were the data that produced this result analysed in accordance with a pre-specified analysis plan that was finalized before unblinded outcome data were available for analysis?	Probably No	There is not a published protocol or SAP
Is the reported effect estimate likely to be selected, on the basis of the results, from...		
7.1. ... multiple outcome measurements within the outcome domain?	No	One outcome measure
7.2 ... multiple analyses of the intervention-outcome relationship?	No	Two very common methods of analysis used
7.3 ... different subgroups?	No	No subgroups
**Secondary Analysis RoB Assessment**		Notes
**Is the seconday analysis evaluating effectiveness or efficacy?**	Efficacy	
**Comparator**	No comparator	
**Outcome Being Assessed**	Perfect use Pearl Index	
**Specify the Numerical Result Being Assessed**	2.0 (95% CI: 1.3, 2.8)	
**Bias due to randomization (See primary analysis section)**		Notes
**Bias due to confounding**		Notes

1.1 Is there potential for confounding of the effect of intervention in this study?	Yes	Participants who reported perfect use during red days were probably more likely to use other forms of contraception on green days too<br>Participants also may have been more concerned with pregnancy (not in a relationship? younger? etc.) and therefore more adherent<br>The study didn't list the demographics of participants included in this sub population
1.2. If Y/PY to 1.1 Was the analysis based on splitting participants' follow up time according to intervention received?	No	
1.3. If Y/PY to 1.2 Were intervention discontinuations or switches likely to be related to factors that are prognostic for the outcome?		
**Questions relating to baseline confounding only**		
1.4. If Y/PY to 1.1 Did the authors use an appropriate analysis method that controlled for all the important confounding domains?	No	
1.5. If Y/PY to 1.4: Were confounding domains that were controlled for measured validly and reliably by the variables available in this study?		
1.6. Did the authors control for any post-intervention variables that could have been affected by the intervention?	No	
**Questions relating to baseline and time-varying confounding**		
1.7. If Y/PY to 1.3 Did the authors use an appropriate analysis method that controlled for all the important confounding domains and for time-varying confounding?		
1.8. If Y/PY to 1.7: Were confounding domains that were controlled for measured validly and reliably by the variables available in this study?		
**Bias in selection of participants into the study (Not evaluating for analyses of effectiveness)**		Notes
2.1. Was selection of participants into the analysis based on participant characteristics observed after the start of intervention?	Probably No	The study was not preregistered but used the same criteria as their previous studies
2.2. If Y/PY to 2.1: Were the post-intervention variables that influenced selection likely to be associated with intervention?		
2.3 If Y/PY to 2.2: Were the post-intervention variables that influenced selection likely to be influenced by the outcome or a cause of the outcome?		
2.4. Do start of follow-up and start of intervention coincide for most participants?	Unknown	
2.5. If Y/PY to 2.2 and 2.3, or N/PN to 2.4: Were adjustment techniques used that are likely to correct for the presence of selection biases?	No	
**Bias in classification of interventions**		Notes
3.1 Were intervention groups clearly defined?	Yes	Adherence was clearly defined
3.2 Was the information used to define intervention groups recorded at the start of the intervention?	Probably No	There was not a published SAP or preregistration so this is impossible to know
3.3 Could classification of intervention status have been affected by knowledge of the outcome or risk of the outcome?	Probably No	Correct use of the app was used to dichotomize participants
**Bias due to deviations from intended interventions (See primary analysis section)**		Notes
**Bias due to missing data**		Notes
5.1 Were outcome data available for all, or nearly all, participants?	Probably Yes	Probably more likely that perfect use participants also provided outcome data
5.2 Were participants excluded due to missing data on intervention status?	No	
5.3 Were participants excluded due to missing data on other variables needed for the analysis?	No	Only 18% of all cycles were included
5.4 If PN/N to 5.1, or Y/PY to 5.2 or 5.3: Are the proportion of participants and reasons for missing data similar across interventions?	NA	
5.5 If PN/N to 5.1, or Y/PY to 5.2 or 5.3: Is there evidence that results were robust to the presence of missing data?	No	Possible sensitivity analyses could have included analyzing participants who tracked their temperature most frequently, cycles where participants did not report unprotected sex, or participants with mostly perfect cycles
**Bias in measurement of outcomes**		Notes
6.1 Could the outcome measure have been influenced by knowledge of the intervention received?	Probably No	
6.2 Were outcome assessors aware of the intervention received by study participants? (blinding of assessors)	Yes	
6.3 Were the methods of outcome assessment comparable across intervention groups?	NA	
6.4 Were any systematic errors in measurement of the outcome related to intervention received?	Yes	
6.5 Could adherence have been influenced by study participation?	No	
**Bias in selection of the reported result**		Notes
Were the data that produced this result analysed in accordance with a pre-specified analysis plan that was finalized before unblinded outcome data were available for analysis?	Probably No	There is not a published protocol or SAP
Is the reported effect estimate likely to be selected, on the basis of the results, from...		
7.1. ... multiple outcome measurements within the outcome domain?	No	One outcome measure
7.2 ... multiple analyses of the intervention-outcome relationship?	Probably No	Threshold is based on the prescribed protocol for the app
7.3 ... different subgroups?	No	No subgroups

Article Key

Criteria for Adherence Metrics and Analysis		Notes
**Device name and indication for use**	Natural Cycles (contraception)	
**Type of device** (Long-term vs short-term use)	Long-term use	
**Type of device** (Presence or absence of a prescribed dosage)	Presence of a prescribed dose	
**Was adherence information collected?**	Yes	
**What information was collected about usage adherence?**	Percentage of days with temperature logging	
**What information was collected about adherence to recommendations?**	Intercourse logging and backup contraception use on red days	
**Was information collected internally in the app?**	Yes	
**Was information modified internally?**	NA	
**Was initiation reported?**	Yes	All included users had to input 20 days of data
**What was average adherence?** (if reported)	100%	
**Was implementation reported?**	Yes	
**What was average adherence?** (if reported)	56.1% of users had a measuring frequency of <50%, 23.9% 50-70%, 20% >70%<br>23% of red day intercourse was unprotected	
**Was persistence reported?**	Yes	
**What was average adherence?** (if reported)	40.9% of women remained using the app after 13 cycles	
**Was adherence low?**	Yes	Most users recorded their temperature less than 50% of the time. Even if the 19.9% of participants logged their temperature 100% of the time, on average all participants would have logged their temperature 64% of the time
**Was adherence not reported or low?**	Yes	
**Was efficacy analyzed?**	Yes	
**What method was used?**	Per-protocol analysis	Perfect use pearl index
**Were efficacy analyses preregistered?**	No	
**What assumptions are required for that method to study efficacy?**	SUTVA, positivity, consistency, ignorability, conditional independence of adherence and outcomes	
**Did the article report evidence that the assumptions were met?**	No	
**SUTVA**	Yes	Treatment only delivered through the app
**Positivity**	No	No control condition
**Consistency** (treatment definition)	Yes	Clear definition of treatment
**Consistency** (adherence definition)	Yes	Clear definition of perfect use
**Exclusion restriction**		
**Strong Monotonicity**		
**Ignorability**	None	
**Overall Notes**		
**Cochrane CDPLG**		**Notes**
**Data form completed date** (dd/mm/yyyy)	4/4/2022	
**Study author contact details**	raoul@naturalcycles.com	
Methods	**Descriptions as stated in report/paper**	**Location in text or source** (pg & ¶/fig/table/other)
Aim of study (e.g. efficacy, equivalence, pragmatic)	Effectiveness	pg. 1 Abstract
Design (e.g. parallel, crossover, non-RCT)	Prospecitve Observational Cohort Study	pg. 2 Study Design
Unit of allocation (by individuals, cluster/ groups or body parts)	Individual	
Participants	**Descriptions as stated in report/paper**	**Location in text or source** (pg & ¶/fig/table/other)
**Inclusion criteria**	"The inclusion criteria were women using the Natural Cycles app as their primary means of contraception who had registered as a paying user on an annual subscription between September 1st 2017 and March 31st 2018, were residents in the US (at time of registration), and were 18–45 years of age"	pg. 2 Study Design
**Exclusion criteria**	Users who entered less than 20 days of data	pg. 4 Discussion
**Total no. randomised** (or total pop. at start of study for NRCTs)	5879	pg. 2 Results
**Clusters** (if applicable, no., type, no. people per cluster)	NA	
**Withdrawals and exclusions** (if not provided below by outcome)		
Intervention Groups	**Descriptions as stated in report/paper**	**Location in text or source** (pg & ¶/fig/table/other)
**Group name**	Natural Cycles Users	pg. 2 Study Design
**No. randomised to group** (specify whether no. people or clusters)	5879	pg. 2 Results
**Timing** (e.g. frequency, duration of each episode)	Participants had to enter the BBT first thing every morning<br>Participants had to abstain from sex or use an alternate form of protection on red days	pg. 1 Introduction
**Co-interventions**	Participants could use condoms or other barrier methods on red days	pg. 2 Study Design
**Integrity of delivery**	Users had to enter at least 20 days of data to be included in the study	pg. 4 Discussion
**Compliance**	56.1% of users had a measuring frequency of <50% and 57% of acts of intercourse were on red days	pg. 3 Intercourse Logging and Percentage Green Days<br>pg. ?? Table 4
Outcomes	**Descriptions as stated in report/paper**	**Location in text or source** (pg & ¶/fig/table/other)
**Outcome name**	Typical use effectiveness	pg. 2 Data Analysis
**Outcome definition** (with diagnostic criteria if relevant)(include name, time, and analysis method)	Typical use effectiveness analyzed using 13-cycle life table analysis and pearl index	pg. 2 Data Analysis
**Imputation of missing data** (e.g. assumptions made for ITT analysis)	"In cases where no confirmation or refutation of pregnancy was available, users who discontinued in the follicular phase, or later in cycle, but no ovulation was detected, were flagged as unlikely to be pregnant. Those who discontinued in the luteal phase after ovulation had been confirmed were considered possibly pregnant. Those who were lost to follow-up and grouped as ''very unlikely pregnant'' were not included in the effectiveness analysis; those who were classed as ''possibly pregnant'' were included in a worst case scenario effectiveness calculation as their pregnancy status was unknown. Users' empty cycles were excluded from the study if they failed to add data for an entire cycle."	pg. 2 Determining Pregnancy and Discontinuation
**Power** (e.g. power & sample size calculation, level of power achieved)		
Outcomes	**Descriptions as stated in report/paper**	**Location in text or source** (pg & ¶/fig/table/other)
**Outcome name**	Perfect use effectiveness	pg. 2 Data Analysis
**Outcome definition** (with diagnostic criteria if relevant)(include name, time, and analysis method)	One year pearl index	pg. 2 Data Analysis
**Imputation of missing data** (e.g. assumptions made for ITT analysis)	Likely no missing data for perfect use cycles	pg. 2 Determining Pregnancy and Discontinuation
**Power** (e.g. power & sample size calculation, level of power achieved)		
**Risk of Bias** (Based on Cochrane RoB Tool 2 and ROBINS-I)		Link to RoB Algorithm
**Primary Analysis RoB Assessment**		Notes
**Is the primary analysis evaluating effectiveness or efficacy?**	Effectiveness	
**Comparator**	No comparator	
**Outcome Being Assessed**	13-cycle cumulative pregnancy probability	
**Specify the Numerical Result Being Assessed**	7.2% (95% confidence interval [CI]: 6.4, 8.1)	
**Bias due to randomization (RCT only)**		Notes
1.1 Was the allocation sequence random?	NA	
1.2. Was the allocation sequence concealed until participants were enrolled and assigned to interventions?	NA	
1.3. Did baseline differences between intervention groups suggest a problem with the randomization process?	NA	
**Bias due to confounding**		Notes
1.1 Is there potential for confounding of the effect of intervention in this study?	Yes	
1.2. If Y/PY to 1.1 Was the analysis based on splitting participants' follow up time according to intervention received?	No	
1.3. If Y/PY to 1.2 Were intervention discontinuations or switches likely to be related to factors that are prognostic for the outcome?		

**Questions relating to baseline confounding only**		
1.4. If Y/PY to 1.1 Did the authors use an appropriate analysis method that controlled for all the important confounding domains?	Probably No	Researchers performed a subgroup analysis to look at important demographic features but they did not look at important factors such as frequency of sex and use of protection on green days
1.5. If Y/PY to 1.4: Were confounding domains that were controlled for measured validly and reliably by the variables available in this study?	Yes	
1.6. Did the authors control for any post-intervention variables that could have been affected by the intervention?	No	
**Questions relating to baseline and time-varying confounding**		
1.7. If Y/PY to 1.3 Did the authors use an appropriate analysis method that controlled for all the important confounding domains and for time-varying confounding?		
1.8. If Y/PY to 1.7: Were confounding domains that were controlled for measured validly and reliably by the variables available in this study?		
**Bias in selection of participants into the study (Not evaluating for analyses of effectiveness)**		Notes
2.1. Was selection of participants into the analysis based on participant characteristics observed after the start of intervention?		
2.2. If Y/PY to 2.1: Were the post-intervention variables that influenced selection likely to be associated with intervention?		
2.3 If Y/PY to 2.2: Were the post-intervention variables that influenced selection likely to be influenced by the outcome or a cause of the outcome?		
2.4. Do start of follow-up and start of intervention coincide for most participants?		
2.5. If Y/PY to 2.2 and 2.3, or N/PN to 2.4: Were adjustment techniques used that are likely to correct for the presence of selection biases?		
**Bias in classification of interventions**		Notes
3.1 Were intervention groups clearly defined? Was the definition of adherence clearly defined?	Yes	
3.2 Was the information used to define intervention groups recorded at the start of the intervention?	No	This study was not preregistered
3.3 Could classification of intervention status have been affected by knowledge of the outcome or risk of the outcome?	Probably No	Only one cohort was defined in this study and the inclusion criteria match prior studies
**Bias due to deviations from intended interventions**		Notes
**ROBINS-I**		
4.1. Were there deviations from the intended intervention beyond what would be expected in usual practice?	No	
4.2. If Y/PY to 4.1: Were these deviations from intended intervention unbalanced between groups and likely to have affected the outcome?		
4.3. Were important co-interventions balanced across intervention groups?	NA	
4.4. Was the intervention implemented successfully for most participants?	Yes	Users needed to log at least 20 days of data
4.5. Did study participants adhere to the assigned intervention regimen?	No	Most users recorded their temperature less than 50% of the time<br>Most acts of intercourse were logged on red days
4.6. If N/PN to 4.3, 4.4 or 4.5: Was an appropriate analysis used to estimate the effect of starting and adhering to the intervention?	No	The required assumptions for this analysis method were not met
Risk of bias: Assignment to intervention	**Low** / Moderate / Serious / Critical / NI	
Risk of bias: Starting and adhering to intervention	Low / Moderate / Serious / **Critical** / NI	
**Risk of bias judgement**	Low / Moderate / Serious / **Critical** / NI	
**RoB Tool (Risk of bias due to deviations from the intended interventions (effect of adhering to intervention))**		
2.1. Were participants aware of their assigned intervention during the trial?	Yes	
2.2. Were carers and people delivering the interventions aware of participants' assigned intervention during the trial?	NA	
2.3. If Y/PY/NI to 2.1 or 2.2: Were important non protocol interventions balanced across intervention groups?	NA	
2.4. [If applicable:] Were there failures in implementing the intervention that could have affected the outcome?	No	Users needed to log at least 20 days of data
2.5. [If applicable:] Was there non-adherence to the assigned intervention regimen that could have affected participants' outcomes?	Yes	Most users recorded their temperature less than 50% of the time<br>Most acts of intercourse were logged on red days
2.6. If N/PN/NI to 2.3, or Y/PY/NI to 2.4 or 2.5: Was an appropriate analysis used to estimate the effect of adhering to the intervention?	No	The required assumptions for this analysis method were not met
**Risk of bias judgement**	Low/**High**/Some Concerns	
**Bias due to missing data**		Notes
5.1 Were outcome data available for all, or nearly all, participants?	No	787 women discontinued the app for unknown reasons(13% of women)
5.2 Were participants excluded due to missing data on intervention status?	No	
5.3 Were participants excluded due to missing data on other variables needed for the analysis?	No	
5.4 If PN/N to 5.1, or Y/PY to 5.2 or 5.3: Are the proportion of participants and reasons for missing data similar across interventions?	NA	
5.5 If PN/N to 5.1, or Y/PY to 5.2 or 5.3: Is there evidence that results were robust to the presence of missing data?	No information	They did a sensitivity analysis in how they categorize pregnancies for the PI calculation but not for the life table analysis
**Bias in measurement of outcomes**		Notes
6.1 Could the outcome measure have been influenced by knowledge of the intervention received?	Probably No	
6.2 Were outcome assessors aware of the intervention received by study participants? (blinding of assessors)	No	
6.3 Were the methods of outcome assessment comparable across intervention groups?	NA	
6.4 Were any systematic errors in measurement of the outcome related to intervention received?	Proabably Yes	They did a sensitivity analysis based on how they categorized/followed up with women and did find a slight increase in the PI when all women were followed up with.<br>This shows that there is some inherent measurement bias in how they categorize pregnancies and should have included these additional pregnancies in the life table assessment
**Bias in selection of the reported result**		Notes
Were the data that produced this result analysed in accordance with a pre-specified analysis plan that was finalized before unblinded outcome data were available for analysis?	No	There is not a published protocol or SAP
Is the reported effect estimate likely to be selected, on the basis of the results, from...		
7.1. ... multiple outcome measurements within the outcome domain?	Probably No	One outcome measure
7.2 ... multiple analyses of the intervention-outcome relationship?	Probably No	Two very common methods of analysis used
7.3 ... different subgroups?	Probably No	No subgroups
**Secondary Analysis RoB Assessment**		Notes
**Is the seconday analysis evaluating effectiveness or efficacy?**	Efficacy	
**Comparator**	No comparator	
**Outcome Being Assessed**	Perfect use pearl index	
**Specify the Numerical Result Being Assessed**	with perfect use, [PI] was 2.0 (95% CI: 0.9, 3.0)	
**Bias due to randomization (See primary analysis section)**		Notes
**Bias due to confounding**		Notes

1.1 Is there potential for confounding of the effect of intervention in this study?	Yes	
1.2. If Y/PY to 1.1 Was the analysis based on splitting participants' follow up time according to intervention received?	No	
1.3. If Y/PY to 1.2 Were intervention discontinuations or switches likely to be related to factors that are prognostic for the outcome?		
**Questions relating to baseline confounding only**		
1.4. If Y/PY to 1.1 Did the authors use an appropriate analysis method that controlled for all the important confounding domains?	No	Study did not report on the characteristics of included participants or perform subgroup analyses
1.5. If Y/PY to 1.4: Were confounding domains that were controlled for measured validly and reliably by the variables available in this study?		
1.6. Did the authors control for any post-intervention variables that could have been affected by the intervention?	No	
**Questions relating to baseline and time-varying confounding**		
1.7. If Y/PY to 1.3 Did the authors use an appropriate analysis method that controlled for all the important confounding domains and for time-varying confounding?		
1.8. If Y/PY to 1.7: Were confounding domains that were controlled for measured validly and reliably by the variables available in this study?		
**Bias in selection of participants into the study (Not evaluating for analyses of effectiveness)**		Notes
2.1. Was selection of participants into the analysis based on participant characteristics observed after the start of intervention?	Yes	
2.2. If Y/PY to 2.1: Were the post-intervention variables that influenced selection likely to be associated with intervention?	Yes	Participants who inputted more BBT info into the app were more likely to be included
2.3 If Y/PY to 2.2: Were the post-intervention variables that influenced selection likely to be influenced by the outcome or a cause of the outcome?	Yes	Participants who used the app more often were probably more likely to have accurate predictions and they may have been more likely to have protected sex on green days
2.4. Do start of follow-up and start of intervention coincide for most participants?	Probably No	7 month timespan between trial start and when participants could have started the app
2.5. If Y/PY to 2.2 and 2.3, or N/PN to 2.4: Were adjustment techniques used that are likely to correct for the presence of selection biases?	No	Researchers based the analysis on the number of cycles each women provided but did not adjust for other things that could vary with time such as frequency of intercourse. It is possible that there are not differences in pregnancy rates depending on the time of year.<br>Researchers don't state who is included or excluded in this analysis or conduct sensitivity analyses or subgroup analyses to investigate this
**Bias in classification of interventions**		Notes
3.1 Were intervention groups clearly defined?	Yes	The same definition of perfect use was used across Natural Cycles papers
3.2 Was the information used to define intervention groups recorded at the start of the intervention?	Probably No	It was not preregistered but they use the same definition throughout their papers
3.3 Could classification of intervention status have been affected by knowledge of the outcome or risk of the outcome?	Probably No	The same definition of perfect use was used across Natural Cycles papers
**Bias due to deviations from intended interventions (See primary analysis section)**		Notes
**Bias due to missing data**		Notes
5.1 Were outcome data available for all, or nearly all, participants?	No	Only 18% of cycles had enough information to classify them as perfect or imperfect; they don't state what percentage of cycles were perfect cycles
5.2 Were participants excluded due to missing data on intervention status?	No	
5.3 Were participants excluded due to missing data on other variables needed for the analysis?	Yes	Participants were excluded if they didn't log intercourse events in the app
5.4 If PN/N to 5.1, or Y/PY to 5.2 or 5.3: Are the proportion of participants and reasons for missing data similar across interventions?	NA	
5.5 If PN/N to 5.1, or Y/PY to 5.2 or 5.3: Is there evidence that results were robust to the presence of missing data?	No	
**Bias in measurement of outcomes**		Notes
6.1 Could the outcome measure have been influenced by knowledge of the intervention received?	Probably No	
6.2 Were outcome assessors aware of the intervention received by study participants? (blinding of assessors)	Yes	
6.3 Were the methods of outcome assessment comparable across intervention groups?	Probably No	There was likely complete data for the perfect use cycles and no assumptions about pregnancy data for people lost to follow-up
6.4 Were any systematic errors in measurement of the outcome related to intervention received?	Probably No	
6.5 Could adherence have been influenced by study participation?	No	
**Bias in selection of the reported result**		Notes
Were the data that produced this result analysed in accordance with a pre-specified analysis plan that was finalized before unblinded outcome data were available for analysis?	No	
Is the reported effect estimate likely to be selected, on the basis of the results, from...		
7.1. ... multiple outcome measurements within the outcome domain?	Probably No	One outcome measure
7.2 ... multiple analyses of the intervention-outcome relationship?	Probably No	Same analysis method used across studies
7.3 ... different subgroups?	Probably No	Same inclusion criteria used across studies

Criteria for Adherence Metrics and Analysis		Notes
**Device name and indication for use**	Apple watch irregular arrhythmia notification	
**Type of device** (Long-term vs short-term use)	Long-term use of watch, short-term use of app	
**Type of device** (Presence or absence of a prescribed dosage)	No prescribed dosage for use of watch, prescribed dosage for use of app	One time use of app in the case of an arrhythmia notification
**Was adherence information collected?**	Yes	
**What information was collected about usage adherence?**	Initial use of app recorded for consent purposes	No information about use of watch
**What information was collected about adherence to recommendations?**	Initiation of visit in response to alert<br>Return of ECG patch	
**Was information collected internally in the app?**	NA	
**Was information modified internally?**	NA	
**Was initiation reported?**	Yes	In-app consent required to participate in research<br>Also initiation of visit in response to alert and return of ECG patch
**What was average adherence?** (if reported)	Use of app: 100%<br>Initiation of first visit: 43.7% (initiated first visit)<br>Return of ECG patch: 69.6% (658/945 or 658/2161)	Return of ECG patch was not an app recommendation
**Was implementation reported?**	No	
**What was average adherence?** (if reported)		
**Was persistence reported?**	No	
**What was average adherence?** (if reported)		
**Was adherence low?**	No information	
**Was adherence not reported or low?**	Yes	
**Was efficacy analyzed?**	No	
**What method was used?**	NA	
**Were efficacy analyses preregistered?**		
**What assumptions are required for that method to study efficacy?**		
**Did the article report evidence that the assumptions were met?**		
**SUTVA**		
**Positivity**		
**Consistency** (treatment definition)		
**Consistency** (adherence definition)		
**Exclusion restriction**		
**Strong Monotonicity**		
**Ignorability**		
**Overall Notes**		
**Cochrane CDPLG**		**Notes**
**Data form completed date** (dd/mm/yyyy)	3/21/2022	
**Study author contact details**	mvperez@stanford.edu or mintu@stanford.edu	
Methods	**Descriptions as stated in report/paper**	**Location in text or source** (pg & ¶/fig/table/other)
Aim of study (e.g. efficacy, equivalence, pragmatic)	Effectiveness<br>Positive predictive value of irregular pulse itervals	pg. 1 Abstract
Design (e.g. parallel, crossover, non-RCT)	"propective, single-group, open-label, siteless, pragmatic study"	pg. 2 Study Design and Oversight
Unit of allocation (by individuals, cluster/ groups or body parts)	Individual	
Participants	**Descriptions as stated in report/paper**	**Location in text or source** (pg & ¶/fig/table/other)
**Inclusion criteria**	"Major eligibility criteria in cluded possession of a compatible Apple iPhone and Apple Watch, an age of 22 years or older, United States residency, and proficiency in English, as reported by the participant"	pg. 2 Study Population
**Exclusion criteria**	"Participants who reported previous atrial fibrillation or current use of oral anticoagulation agents were not eligible"	pg. 2 Study Population
**Total no. randomised** (or total pop. at start of study for NRCTs)	419297	pg. 4 Figure 1
**Clusters** (if applicable, no., type, no. people per cluster)	NA	
**Withdrawals and exclusions** (if not provided below by outcome)	NA	
Intervention Groups	**Descriptions as stated in report/paper**	**Location in text or source** (pg & ¶/fig/table/other)
**Group name**	Apple Watch monitoring<br>participants were monitored for irregular pulse with apple watch and sent a notification if irregular pulse was detected and prompted to have a telemedicine visit	pg. 2-3 Monitoring and Study Intervention
**No. randomised to group** (specify whether no. people or clusters)	419297	pg. 4 Figure 1
**Timing** (e.g. frequency, duration of each episode)	No information about instructions given to participants to wear the watch	pg. 3 Statistical Analysis
**Co-interventions**		
**Integrity of delivery**	100% of people had the app downloaded since it was used for informed consent	pg. 2 Study Population
**Compliance**	No information reported about compliance in terms of wearing the watch	
Intervention Groups	**Descriptions as stated in report/paper**	**Location in text or source** (pg & ¶/fig/table/other)
**Group name**	Notified subgroup	pg. 2-3 Monitoring and Study Intervention
**No. randomised to group** (specify whether no. people or clusters)	2161	pg. 4 Figure 1
**Timing** (e.g. frequency, duration of each episode)	No information reported about compliance in terms of wearing the watch. *I* f a positive notification was received, participants were instructed to meet with a telemedicine provider, wear an ECG patch within 14 days for at least 1 hour and up to 7 days and mail the patch back within 45 days	pg. 3 Statistical Analysis
**Co-interventions**		
**Integrity of delivery**	100% of people had the app downloaded since it was used for informed consent	pg. 2 Study Population
**Compliance**	450/2161 sent back an ECG patch in the alloted time	pg. ?? Table 1
Outcomes	**Descriptions as stated in report/paper**	**Location in text or source** (pg & ¶/fig/table/other)
**Outcome name**	Atrial fibrillation of greater than 30 seconds' duration on ECG patch monitoring in a participant who received anirregular pulse notification	pg. 3 Primary and Secondary Outcomes
**Outcome definition** (with diagnostic criteria if relevant)(include name, time, and analysis method)	Proportion and CI of notified participants with confirmed afib	pg. 3 Statistical Analysis
**Imputation of missing data** (e.g. assumptions made for ITT analysis)	No imputation	
**Power** (e.g. power & sample size calculation, level of power achieved)	Goal was to collect 503 ECG patches in each of two age groups to have a 97.5% CI no wider than 0.1 for PPV. The study was underpowered for this goal	pg. 3 Statistical Analysis
Outcomes	**Descriptions as stated in report/paper**	**Location in text or source** (pg & ¶/fig/table/other)
**Outcome name**	Simultaneous atrial fibrillation on ECG patch monitoring during intervals when the participant had an irregular tachogram	pg. 3 Primary and Secondary Outcomes
**Outcome definition** (with diagnostic criteria if relevant)(include name, time, and analysis method)	Proportion and CI of notified participants with confirmed afib and PPV	pg. 3 Statistical Analysis
**Imputation of missing data** (e.g. assumptions made for ITT analysis)	No imputation	
**Power** (e.g. power & sample size calculation, level of power achieved)	Goal was to collect 503 ECG patches in each of two age groups to have a 97.5% CI no wider than 0.1 for PPV. The study was underpowered for this goal	pg. 3 Statistical Analysis
**Risk of Bias** (Based on Cochrane RoB Tool 2 and ROBINS-I)		Link to RoB Algorithm
**Primary Analysis RoB Assessment**		Notes
**Is the primary analysis evaluating effectiveness or efficacy?**	Effectiveness	
**Comparator**	Apple watch compared to ECG patch review by physicians	
**Outcome Being Assessed**	Proportion of ECG patches with confirmed atrial fibrillation<br>Positive predictive value (proportion and confidence interval)	
**Specify the Numerical Result Being Assessed**	34% (97.5% CI, 29 to 39)<br>0.71 (97.5% CI, 0.69 to 0.74)	
**Bias due to randomization (RCT only)**		Notes
1.1 Was the allocation sequence random?	NA	
1.2. Was the allocation sequence concealed until participants were enrolled and assigned to interventions?	NA	

Article Key

1.3. Did baseline differences between intervention groups suggest a problem with the randomization process?	NA	
**Bias due to confounding**		Notes
1.1 Is there potential for confounding of the effect of intervention in this study?	Yes	Participants who wore the watch often could have been more likely to test with the patch<br>Age could also have confounded the results
1.2. If Y/PY to 1.1 Was the analysis based on splitting participants' follow up time according to intervention received?	No	
1.3. If Y/PY to 1.2 Were intervention discontinuations or switches likely to be related to factors that are prognostic for the outcome?		
**Questions relating to baseline confounding only**		
1.4. If Y/PY to 1.1 Did the authors use an appropriate analysis method that controlled for all the important confounding domains?	Probably No	Researchers looked into age and baseline differences between groups but didn't look at things such as symptoms
1.5. If Y/PY to 1.4: Were confounding domains that were controlled for measured validly and reliably by the variables available in this study?	Yes	
1.6. Did the authors control for any post-intervention variables that could have been affected by the intervention?	No	
**Questions relating to baseline and time-varying confounding**		
1.7. If Y/PY to 1.3 Did the authors use an appropriate analysis method that controlled for all the important confounding domains and for time-varying confounding?		
1.8. If Y/PY to 1.7: Were confounding domains that were controlled for measured validly and reliably by the variables available in this study?		
**Bias in selection of participants into the study (Not evaluating for analyses of effectiveness)**		Notes
2.1. Was selection of participants into the analysis based on participant characteristics observed after the start of intervention?		
2.2. If Y/PY to 2.1: Were the post-intervention variables that influenced selection likely to be associated with intervention?		
2.3 If Y/PY to 2.2: Were the post-intervention variables that influenced selection likely to be influenced by the outcome or a cause of the outcome?		
2.4. Do start of follow-up and start of intervention coincide for most participants?		
2.5. If Y/PY to 2.2 and 2.3, or N/PN to 2.4: Were adjustment techniques used that are likely to correct for the presence of selection biases?		
**Bias in classification of interventions**		Notes
3.1 Were intervention groups clearly defined? Was the definition of adherence clearly defined?	Yes	
3.2 Was the information used to define intervention groups recorded at the start of the intervention?	Yes	Preregistered description of the intervention
3.3 Could classification of intervention status have been affected by knowledge of the outcome or risk of the outcome?	No	
**Bias due to deviations from intended interventions**		Notes
**ROBINS-I**		
4.1. Were there deviations from the intended intervention beyond what would be expected in usual practice?	No	
4.2. If Y/PY to 4.1: Were these deviations from intended intervention unbalanced between groups and likely to have affected the outcome?		
4.3. Were important co-interventions balanced across intervention groups?	NA	
4.4. Was the intervention implemented successfully for most participants?	Yes	
4.5. Did study participants adhere to the assigned intervention regimen?	No information	
4.6. If N/PN to 4.3, 4.4 or 4.5: Was an appropriate analysis used to estimate the effect of starting and adhering to the intervention?	No	No analysis performed
Risk of bias: Assignment to intervention	**Low** / Moderate / Serious / Critical / NI	
Risk of bias: Starting and adhering to intervention	Low / Moderate / Serious / **Critical** / NI	
**Risk of bias judgement**	Low / Moderate / Serious / **Critical** / NI	
**RoB Tool (Risk of bias due to deviations from the intended interventions (effect of adhering to intervention))**		
2.1. Were participants aware of their assigned intervention during the trial?	Yes	
2.2. Were carers and people delivering the interventions aware of participants' assigned intervention during the trial?	NA	
2.3. If Y/PY/NI to 2.1 or 2.2: Were important non protocol interventions balanced across intervention groups?	NA	
2.4. [If applicable:] Were there failures in implementing the intervention that could have affected the outcome?	No	
2.5. [If applicable:] Was there non-adherence to the assigned intervention regimen that could have affected participants' outcomes?	No information	
2.6. If N/PN/NI to 2.3, or Y/PY/NI to 2.4 or 2.5: Was an appropriate analysis used to estimate the effect of adhering to the intervention?	No	No analysis performed
**Risk of bias judgement**	Low/**High**/Some Concerns	
**Bias due to missing data**		Notes
5.1 Were outcome data available for all, or nearly all, participants?	No	1216/2161 participants did not initiate a telemedicine visit
5.2 Were participants excluded due to missing data on intervention status?	No	
5.3 Were participants excluded due to missing data on other variables needed for the analysis?	No	
5.4 If PN/N to 5.1, or Y/PY to 5.2 or 5.3: Are the proportion of participants and reasons for missing data similar across interventions?	NA	
5.5 If PN/N to 5.1, or Y/PY to 5.2 or 5.3: Is there evidence that results were robust to the presence of missing data?	No	Researchers compared baseline statistics of the notified group between participants who did and didn't submit and ECG recording and not those who did and didn't initiate a visit
**Bias in measurement of outcomes**		Notes
6.1 Could the outcome measure have been influenced by knowledge of the intervention received?	No	Only looked at PPV
6.2 Were outcome assessors aware of the intervention received by study participants? (blinding of assessors)	Yes	
6.3 Were the methods of outcome assessment comparable across intervention groups?	NA	
6.4 Were any systematic errors in measurement of the outcome related to intervention received?	No	
**Bias in selection of the reported result**		Notes
Were the data that produced this result analysed in accordance with a pre-specified analysis plan that was finalized before unblinded outcome data were available for analysis?	Yes	Thorough registration and published SAP
Is the reported effect estimate likely to be selected, on the basis of the results, from...		
7.1. ... multiple outcome measurements within the outcome domain?	No	
7.2 ... multiple analyses of the intervention-outcome relationship?	No	
7.3 ... different subgroups?	No	
**Secondary Analysis RoB Assessment**		Notes
**Is the seconday analysis evaluating effectiveness or efficacy?**		
**Comparator**		
**Outcome Being Assessed**		
**Specify the Numerical Result Being Assessed**		
**Bias due to randomization (See primary analysis section)**		Notes
**Bias due to confounding**		Notes
1.1 Is there potential for confounding of the effect of intervention in this study?		
1.2. If Y/PY to 1.1 Was the analysis based on splitting participants' follow up time according to intervention received?		

1.3. If Y/PY to 1.2 Were intervention discontinuations or switches likely to be related to factors that are prognostic for the outcome?		
**Questions relating to baseline confounding only**		
1.4. If Y/PY to 1.1 Did the authors use an appropriate analysis method that controlled for all the important confounding domains?		
1.5. If Y/PY to 1.4: Were confounding domains that were controlled for measured validly and reliably by the variables available in this study?		
1.6. Did the authors control for any post-intervention variables that could have been affected by the intervention?		
**Questions relating to baseline and time-varying confounding**		
1.7. If Y/PY to 1.3 Did the authors use an appropriate analysis method that controlled for all the important confounding domains and for time-varying confounding?		
1.8. If Y/PY to 1.7: Were confounding domains that were controlled for measured validly and reliably by the variables available in this study?		
**Bias in selection of participants into the study (Not evaluating for analyses of effectiveness)**		Notes
2.1. Was selection of participants into the analysis based on participant characteristics observed after the start of intervention?		
2.2. If Y/PY to 2.1: Were the post-intervention variables that influenced selection likely to be associated with intervention?		
2.3 If Y/PY to 2.2: Were the post-intervention variables that influenced selection likely to be influenced by the outcome or a cause of the outcome?		
2.4. Do start of follow-up and start of intervention coincide for most participants?		
2.5. If Y/PY to 2.2 and 2.3, or N/PN to 2.4: Were adjustment techniques used that are likely to correct for the presence of selection biases?		
**Bias in classification of interventions**		Notes
3.1 Were intervention groups clearly defined?		
3.2 Was the information used to define intervention groups recorded at the start of the intervention?		
3.3 Could classification of intervention status have been affected by knowledge of the outcome or risk of the outcome?		
**Bias due to deviations from intended interventions (See primary analysis section)**		Notes
**Bias due to missing data**		Notes
5.1 Were outcome data available for all, or nearly all, participants?		
5.2 Were participants excluded due to missing data on intervention status?		
5.3 Were participants excluded due to missing data on other variables needed for the analysis?		
5.4 If PN/N to 5.1, or Y/PY to 5.2 or 5.3: Are the proportion of participants and reasons for missing data similar across interventions?		
5.5 If PN/N to 5.1, or Y/PY to 5.2 or 5.3: Is there evidence that results were robust to the presence of missing data?		
**Bias in measurement of outcomes**		Notes
6.1 Could the outcome measure have been influenced by knowledge of the intervention received?		
6.2 Were outcome assessors aware of the intervention received by study participants? (blinding of assessors)		
6.3 Were the methods of outcome assessment comparable across intervention groups?		
6.4 Were any systematic errors in measurement of the outcome related to intervention received?		
6.5 Could adherence have been influenced by study participation?		
**Bias in selection of the reported result**		Notes
Were the data that produced this result analysed in accordance with a pre-specified analysis plan that was finalized before unblinded outcome data were available for analysis?		
Is the reported effect estimate likely to be selected, on the basis of the results, from...		
7.1. ... multiple outcome measurements within the outcome domain?		
7.2 ... multiple analyses of the intervention-outcome relationship?		
7.3 ... different subgroups?		

Criteria for Adherence Metrics and Analysis		Notes
**Device name and indication for use**	Welldoc (personalized coaching for diabetes)	
**Type of device** (Long-term vs short-term use)	Long-term use	
**Type of device** (Presence or absence of a prescribed dosage)	Absence of a prescribed dosage	There is a set notification schedule based on each patient's risk level but this is not reported in the article
**Was adherence information collected?**	No	
**What information was collected about usage adherence?**		
**What information was collected about adherence to recommendations?**	NA	No explicit app recommendations
**Was information collected internally in the app?**	NA	
**Was information modified internally?**	NA	
**Was initiation reported?**		
**What was average adherence?** (if reported)		
**Was implementation reported?**		
**What was average adherence?** (if reported)		
**Was persistence reported?**		
**What was average adherence?** (if reported)		
**Was adherence low?**	NA	
**Was adherence not reported or low?**	Yes	
**Was efficacy analyzed?**	No	
**What method was used?**	NA	
**Were efficacy analyses preregistered?**		
**What assumptions are required for that method to study efficacy?**		
**Did the article report evidence that the assumptions were met?**		
**SUTVA**		
**Positivity**		
**Consistency** (treatment definition)		
**Consistency** (adherence definition)		
**Exclusion restriction**		
**Strong Monotonicity**		
**Ignorability**		
**Overall Notes**		
**Cochrane CDPLG**		**Notes**
**Data form completed date** (dd/mm/yyyy)	3/21/2022	
**Study author contact details**	cquinn@epi.umaryland.edu	
Methods	**Descriptions as stated in report/paper**	**Location in text or source** (pg & ¶/fig/table/other)
Aim of study (e.g. efficacy, equivalence, pragmatic)	Effectiveness	pg. 1 Introduction
Design (e.g. parallel, crossover, non-RCT)	Cluster Randomized Controlled Trial	pg. 1 Eligibility and Study Design
Unit of allocation (by individuals, cluster/ groups or body parts)	Cluster	pg. 1 Eligibility and Study Design
Participants	**Descriptions as stated in report/paper**	**Location in text or source** (pg & ¶/fig/table/other)
**Inclusion criteria**	"Physician diagnosis of type 2 diabetes for >=6 months<br>Glycated hemoglobin >=7.5% within 3 months<br>Age 18–64 years"	pg. 2 Eligibility and Study Design
**Exclusion criteria**	Medicare or medicaid beneficiary<br>"Uninsured<br>Insulin pump users<br>Not currently managed by study physicians<br>Pregnant<br>Active substance, alcohol, or drug abuser (sober ,1 year)<br>Psychotic or schizophrenic under active care<br>Severe hearing or visual impairment; or<br>No Internet or e-mail access."	pg. 2 Eligibility and Study Design
**Total no. randomised** (or total pop. at start of study for NRCTs)	213 in all 4 groups (142 in main groups)	pg. ?? Figure 1
**Clusters** (if applicable, no., type, no. people per cluster)	26	pg. 1 Eligibility and Study Design
**Withdrawals and exclusions** (if not provided below by outcome)	23 individuals dropped out; 27 did not reconsent	pg. ?? Figure 1
Intervention Groups	**Descriptions as stated in report/paper**	**Location in text or source** (pg & ¶/fig/table/other)
**Group name**	Control: Usual Care (UC)	pg. ?? Figure 1
**No. randomised to group** (specify whether no. people or clusters)	62 (3 did not reconsent)	pg. ?? Figure 1
**Timing** (e.g. frequency, duration of each episode)	No information (It is likely that participants were asked to provide daily blood glucose readings)	
**Co-interventions**	Glucose meter and supplies	pg. 3 Eligibility and Study Design
**Integrity of delivery**	NA	
**Compliance**	NA	
Intervention Groups	**Descriptions as stated in report/paper**	**Location in text or source** (pg & ¶/fig/table/other)
**Group name**	Coach-PCP Portal with Decision Support (CPDS)	pg. ?? Figure 1
**No. randomised to group** (specify whether no. people or clusters)	80 (12 did not reconsent)	pg. ?? Figure 1
**Timing** (e.g. frequency, duration of each episode)	No information (It is likely that participants were asked to provide daily blood glucose readings)	
**Co-interventions**	Glucose meter and supplies<br>Patients in the active groups received a mobile phone, 1-year unlimited service and data plan	pg. 3 Eligibility and Study Design
**Integrity of delivery**	No information	
**Compliance**	No information	
Outcomes	**Descriptions as stated in report/paper**	**Location in text or source** (pg & ¶/fig/table/other)
**Outcome name**	Change in percent of glycated hemoglobin at one year between CPDS and UC	pg. 3 Primary Outcome
**Outcome definition** (with diagnostic criteria if relevant)(include name, time, and analysis method)		
**Imputation of missing data** (e.g. assumptions made for ITT analysis)	Used a mixed effects model to calculate the outcome and weighted estimating equations	pg. 3 Statistical Analyses
**Power** (e.g. power & sample size calculation, level of power achieved)	"80% power to detect a difference in mean glycated hemoglobin changes of 0.65 SD, corresponding to 1.0% if SD was 1.58%, using a two-sided test with 0.05 type I error after accounting for a within cluster correlation of 0.10"	pg. 3 Statistical Analyses
**Risk of Bias** (Based on Cochrane RoB Tool 2 and ROBINS-I)		Link to RoB Algorithm
**Primary Analysis RoB Assessment**		Notes
**Is the primary analysis evaluating effectiveness or efficacy?**	Effectiveness	
**Comparator**	CPDS vs UC	
**Outcome Being Assessed**	Percent of glycated hemoglobin at 12 months analyzed with ITT using a mixed effects model	
**Specify the Numerical Result Being Assessed**	"12 month decrease in CPDS glycated hemoglobin was 1.2% more than UC (95% CI 0.5-1.9%; p=0.001)"	
**Bias due to randomization (RCT only)**		Notes
1.1 Was the allocation sequence random?	Yes	
1.2. Was the allocation sequence concealed until participants were enrolled and assigned to interventions?	No information	Physician clusters were blinded until allocated but not before patients were recruited
1.3. Did baseline differences between intervention groups suggest a problem with the randomization process?	No	Only one covariate with a significant difference which would be expected given the number of covariates collected
**Bias due to confounding**		Notes

Article Key

1.1 Is there potential for confounding of the effect of intervention in this study?	Yes	Randomization was not perfect given that there was not blinding prior to allocation of the treatment. Participants also needed to reconsent to join the trial which would have also led to imperfect randomization.
1.2. If Y/PY to 1.1 Was the analysis based on splitting participants' follow up time according to intervention received?	No	
1.3. If Y/PY to 1.2 Were intervention discontinuations or switches likely to be related to factors that are prognostic for the outcome?		
**Questions relating to baseline confounding only**		
1.4. If Y/PY to 1.1 Did the authors use an appropriate analysis method that controlled for all the important confounding domains?	Probably Yes	They used a mixed effects model to account for within practice and within patient effects and accounted for time effects
1.5. If Y/PY to 1.4: Were confounding domains that were controlled for measured validly and reliably by the variables available in this study?	Yes	
1.6. Did the authors control for any post-intervention variables that could have been affected by the intervention?	No	
**Questions relating to baseline and time-varying confounding**		
1.7. If Y/PY to 1.3 Did the authors use an appropriate analysis method that controlled for all the important confounding domains and for time-varying confounding?		
1.8. If Y/PY to 1.7: Were confounding domains that were controlled for measured validly and reliably by the variables available in this study?		
**Bias in selection of participants into the study (Not evaluating for analyses of effectiveness)**		Notes
2.1. Was selection of participants into the analysis based on participant characteristics observed after the start of intervention?		
2.2. If Y/PY to 2.1: Were the post-intervention variables that influenced selection likely to be associated with intervention?		
2.3 If Y/PY to 2.2: Were the post-intervention variables that influenced selection likely to be influenced by the outcome or a cause of the outcome?		
2.4. Do start of follow-up and start of intervention coincide for most participants?		
2.5. If Y/PY to 2.2 and 2.3, or N/PN to 2.4: Were adjustment techniques used that are likely to correct for the presence of selection biases?		
**Bias in classification of interventions**		Notes
3.1 Were intervention groups clearly defined? Was the definition of adherence clearly defined?	Yes	
3.2 Was the information used to define intervention groups recorded at the start of the intervention?	Yes	Definitions were preregistered in a published protocol
3.3 Could classification of intervention status have been affected by knowledge of the outcome or risk of the outcome?	No	
**Bias due to deviations from intended interventions**		Notes
**ROBINS-I**		
4.1. Were there deviations from the intended intervention beyond what would be expected in usual practice?	No	
4.2. If Y/PY to 4.1: Were these deviations from intended intervention unbalanced between groups and likely to have affected the outcome?		
4.3. Were important co-interventions balanced across intervention groups?	No	The free mobile plan was not balanced and could make the results harder to extrapolate to other populations
4.4. Was the intervention implemented successfully for most participants?	No Information	
4.5. Did study participants adhere to the assigned intervention regimen?	No Information	
4.6. If N/PN to 4.3, 4.4 or 4.5: Was an appropriate analysis used to estimate the effect of starting and adhering to the intervention?	No	No method was used to analyze efficacy
Risk of bias: Assignment to intervention	**Low** / Moderate / Serious / Critical / NI	
Risk of bias: Starting and adhering to intervention	Low / Moderate / Serious / **Critical** / NI	
**Risk of bias judgement**	Low / Moderate / Serious / **Critical** / NI	
**RoB Tool (Risk of bias due to deviations from the intended interventions (effect of adhering to intervention))**		
2.1. Were participants aware of their assigned intervention during the trial?	Yes	
2.2. Were carers and people delivering the interventions aware of participants' assigned intervention during the trial?	Yes	
2.3. If Y/PY/NI to 2.1 or 2.2: Were important non protocol interventions balanced across intervention groups?	NA	
2.4. [If applicable:] Were there failures in implementing the intervention that could have affected the outcome?	No Information	
2.5. [If applicable:] Was there non-adherence to the assigned intervention regimen that could have affected participants' outcomes?	No Information	
2.6. If N/PN/NI to 2.3, or Y/PY/NI to 2.4 or 2.5: Was an appropriate analysis used to estimate the effect of adhering to the intervention?	No	No method was used to analyze efficacy
**Risk of bias judgement**	Low/**High**/Some Concerns	
**Bias due to missing data**		Notes
5.1 Were outcome data available for all, or nearly all, participants?	Probably No	Missing for 25% of participants
5.2 Were participants excluded due to missing data on intervention status?	No	
5.3 Were participants excluded due to missing data on other variables needed for the analysis?	Yes	Many participants failed to reconsent into the trial
5.4 If PN/N to 5.1, or Y/PY to 5.2 or 5.3: Are the proportion of participants and reasons for missing data similar across interventions?	No	A higher percentage of participants failed to reconsent in the treatment group
5.5 If PN/N to 5.1, or Y/PY to 5.2 or 5.3: Is there evidence that results were robust to the presence of missing data?	Yes	They used two methods to imput missing data and found similar results
**Bias in measurement of outcomes**		Notes
6.1 Could the outcome measure have been influenced by knowledge of the intervention received?	No	An objective measurement was used
6.2 Were outcome assessors aware of the intervention received by study participants? (blinding of assessors)	Probably Yes	Assessors were blinded for assessment at baseline but it was not clear if they were also blinded for the final outcome assessment
6.3 Were the methods of outcome assessment comparable across intervention groups?	Yes	
6.4 Were any systematic errors in measurement of the outcome related to intervention received?	No	
**Bias in selection of the reported result**		Notes
Were the data that produced this result analysed in accordance with a pre-specified analysis plan that was finalized before unblinded outcome data were available for analysis?	Yes	https://www.sciencedirect.com/science/article/pii/S1551714409000342?via%3Dihub
Is the reported effect estimate likely to be selected, on the basis of the results, from...		
7.1. ... multiple outcome measurements within the outcome domain?	No	
7.2 ... multiple analyses of the intervention-outcome relationship?	No	
7.3 ... different subgroups?	No	
**Secondary Analysis RoB Assessment**		Notes
**Is the seconday analysis evaluating effectiveness or efficacy?**		
**Comparator**		
**Outcome Being Assessed**		
**Specify the Numerical Result Being Assessed**		
**Bias due to randomization (See primary analysis section)**		Notes
**Bias due to confounding**		Notes
1.1 Is there potential for confounding of the effect of intervention in this study?		
1.2. If Y/PY to 1.1 Was the analysis based on splitting participants' follow up time according to intervention received?		

1.3. If Y/PY to 1.2 Were intervention discontinuations or switches likely to be related to factors that are prognostic for the outcome?		
**Questions relating to baseline confounding only**		
1.4. If Y/PY to 1.1 Did the authors use an appropriate analysis method that controlled for all the important confounding domains?		
1.5. If Y/PY to 1.4: Were confounding domains that were controlled for measured validly and reliably by the variables available in this study?		
1.6. Did the authors control for any post-intervention variables that could have been affected by the intervention?		
**Questions relating to baseline and time-varying confounding**		
1.7. If Y/PY to 1.3 Did the authors use an appropriate analysis method that controlled for all the important confounding domains and for time-varying confounding?		
1.8. If Y/PY to 1.7: Were confounding domains that were controlled for measured validly and reliably by the variables available in this study?		
**Bias in selection of participants into the study (Not evaluating for analyses of effectiveness)**		Notes
2.1. Was selection of participants into the analysis based on participant characteristics observed after the start of intervention?		
2.2. If Y/PY to 2.1: Were the post-intervention variables that influenced selection likely to be associated with intervention?		
2.3 If Y/PY to 2.2: Were the post-intervention variables that influenced selection likely to be influenced by the outcome or a cause of the outcome?		
2.4. Do start of follow-up and start of intervention coincide for most participants?		
2.5. If Y/PY to 2.2 and 2.3, or N/PN to 2.4: Were adjustment techniques used that are likely to correct for the presence of selection biases?		
**Bias in classification of interventions**		Notes
3.1 Were intervention groups clearly defined?		
3.2 Was the information used to define intervention groups recorded at the start of the intervention?		
3.3 Could classification of intervention status have been affected by knowledge of the outcome or risk of the outcome?		
**Bias due to deviations from intended interventions (See primary analysis section)**		Notes
**Bias due to missing data**		Notes
5.1 Were outcome data available for all, or nearly all, participants?		
5.2 Were participants excluded due to missing data on intervention status?		
5.3 Were participants excluded due to missing data on other variables needed for the analysis?		
5.4 If PN/N to 5.1, or Y/PY to 5.2 or 5.3: Are the proportion of participants and reasons for missing data similar across interventions?		
5.5 If PN/N to 5.1, or Y/PY to 5.2 or 5.3: Is there evidence that results were robust to the presence of missing data?		
**Bias in measurement of outcomes**		Notes
6.1 Could the outcome measure have been influenced by knowledge of the intervention received?		
6.2 Were outcome assessors aware of the intervention received by study participants? (blinding of assessors)		
6.3 Were the methods of outcome assessment comparable across intervention groups?		
6.4 Were any systematic errors in measurement of the outcome related to intervention received?		
6.5 Could adherence have been influenced by study participation?		
**Bias in selection of the reported result**		Notes
Were the data that produced this result analysed in accordance with a pre-specified analysis plan that was finalized before unblinded outcome data were available for analysis?		
Is the reported effect estimate likely to be selected, on the basis of the results, from...		
7.1. ... multiple outcome measurements within the outcome domain?		
7.2 ... multiple analyses of the intervention-outcome relationship?		
7.3 ... different subgroups?		

Criteria for Adherence Metrics and Analysis		Notes
**Device name and indication for use**	Somryst (Cognitive behavioral therapy for insomnia)	
**Type of device** (Long-term vs short-term use)	Short-term use	
**Type of device** (Presence or absence of a prescribed dosage)	Presence of a prescribed dosage	
**Was adherence information collected?**	Yes	
**What information was collected about usage adherence?**	Login count<br>Diary count<br>Number of Cores completed	
**What information was collected about adherence to recommendations?**	NA	No explicit app recommendations
**Was information collected internally in the app?**	Yes	
**Was information modified internally?**	NA	
**Was initiation reported?**	Yes	
**What was average adherence?** (if reported)	3.3% (5 of 151) of the SHUTi group did not complete any of the cores, 3.3% (5 of 151) completed only the tutorial core explaining how the program works	
**Was implementation reported?**	No	They reported the number of participants who compled each core
**What was average adherence?** (if reported)		
**Was persistence reported?**	Yes	
**What was average adherence?** (if reported)	"A total of 60.3% (91 of 151) of the SHUTi group completed all 6 cores of the program"	
**Was adherence low?**	Yes	Less than 80% of participants completed 6 cores
**Was adherence not reported or low and could have affected outcomes?**	Yes	
**Was efficacy analyzed?**	Yes	
**What method was used?**	As-treated analysis	Estimated marginal means using ANCOVA where 6 core completion is the predictor and baseline ISI is controlled for
**Were efficacy analyses preregistered?**	No	
**What assumptions are required for that method to study efficacy?**	SUTVA, Positivity, Consistency, Ignorability, Conditional independence between adherence and outcomes	
**Did the article report evidence that the assumptions were met?**	No	
**SUTVA**	Yes	Treatment would not change based on the intervention
**Positivity**	Yes	Treatment was not random but it is likely that there was a non-zero chance of receiving treatment
**Consistency** (treatment definition)	Yes	Clear treatment definition
**Consistency** (adherence definition)	Yes	Clear definition of adherence
**Exclusion restriction**		
**Strong Monotonicity**		
**Ignorability**	Baseline ISI	No information about how this predictor was chosen
**Overall Notes**		

Article Key

Cochrane CDPLG		Notes
**Data form completed date** (dd/mm/yyyy)	2/21/2022	
**Study author contact details**	leer@virginia.edu	
Methods	**Descriptions as stated in report/paper**	**Location in text or source** (pg & ¶/fig/table/other)
Aim of study (e.g. efficacy, equivalence, pragmatic)	Effectiveness according to our definition	pg. 4 Statistical Analyses
Design (e.g. parallel, crossover, non-RCT)	RCT	pg. 2 Design
Unit of allocation (by individuals, cluster/ groups or body parts)	Individual	
Participants	**Descriptions as stated in report/paper**	**Location in text or source** (pg & ¶/fig/table/other)
**Inclusion criteria**	"(1) they required more than 30 minutes to fall asleep at the beginning of the night (sleep-onset insomnia) ormore than 30minutes of time awake after initially falling asleep (sleep maintenance insomnia) for at least 3 nights per week for at least 6 months,<br>(2) their total sleep time averaged 6.5 hours or less, and<br>(3) they manifested sleep disturbances (or associated daytime symptoms) causing significant distress or impairment in social, occupational, or other areas of functioning. "	pg. 2 Participants
**Exclusion criteria**	"(1) the presence of another untreated sleep disorder,<br>(2) an irregular schedule that would prevent adoption of intervention strategies (ie, work schedule resulting in usual bedtime earlier than 8 PM or later than 2 AM or arising time earlier than 4 AM or later than 10 AM),<br>(3) pregnancy,<br>(4) current or past behavioral treatment for insomnia, and<br>(5) initiation of any psychological treatment within the previous 3 months.<br>Comorbid psychiatric disorders were included unless the individual met criteria for any of the following: moderate to high current risk of suicide, severe major depression, bipolar disorder, or alcohol or drug abuse within the past year. Comorbid medical conditions were included unless they were deemed active, unstable, and degenerative (eg, congestive heart failure or multiple sclerosis) in a manner that could worsen the insomnia. Participants could be taking medications, including sleep medications, if the medication regimen had not been changed in the previous 3 months."	pg. 2 Participants
**Total no. randomised** (or total pop. at start of study for NRCTs)	303	pg. 3 Figure 1
**Clusters** (if applicable, no., type, no. people per cluster)		
**Withdrawals and exclusions** (if not provided below by outcome)	53 drop outs	pg. 7 Discussion
Intervention Groups	**Descriptions as stated in report/paper**	**Location in text or source** (pg & ¶/fig/table/other)
**Group name**	SHUTi	pg. 3 Figure 1
**No. randomised to group** (specify whether no. people or clusters)	151	pg. 3 Figure 1
**Timing** (e.g. frequency, duration of each episode)	6 cores over 9 weeks. The next core is available 7 days after the last core completion and it's expected to take 12 hours to complete all of the material	Supplemental Material 1
**Co-interventions**	Normal treatment, such as medications, if it hadn't changed in the last 3 months	pg. 2 Participants
**Integrity of delivery**	"3.3% (5 of 151) of the SHUTi group did not complete any of the cores, 3.3% (5 of 151) completed only the tutorial core explaining how the program works"	pg. 6 Use and Adherence
**Compliance**	"A total of 60.3% (91 of 151) of the SHUTi group completed all 6 cores of the program"	pg. 6 Use and Adherence
Intervention Groups	**Descriptions as stated in report/paper**	**Location in text or source** (pg & ¶/fig/table/other)
**Group name**	Patient Education	pg. 3 Figure 1
**No. randomised to group** (specify whether no. people or clusters)	152	pg. 3 Figure 1
**Timing** (e.g. frequency, duration of each episode)	Participants could access static information as often as they liked	Supplemental Material 1
**Co-interventions**	Normal treatment, such as medications, if it hadn't changed in the last 3 months	pg. 2 Participants
**Integrity of delivery**		
**Compliance**		
Outcomes	**Descriptions as stated in report/paper**	**Location in text or source** (pg & ¶/fig/table/other)
**Outcome name**	Insomnia Severity Index (ISI)	pg. 2 Introduction
**Outcome definition** (with diagnostic criteria if relevant)(include name, time, and analysis method)	7-item global index of self-reported insomnia syptom severity	pg. 2 Outcome Measures
**Imputation of missing data** (e.g. assumptions made for ITT analysis)	No information about imputation	
**Power** (e.g. power & sample size calculation, level of power achieved)	"Sample size was calculated based on an effect size between groups of Cohen d = 0.20 on each of the 3 primary sleep outcomes (ISI, SOL, andWASO) and an anticipated 35.0% attrition rate across the 1-year study. Powering the study at 80% to detect pre-post differences between groups with α = .05 (using G*Power 3 software), a sample size of 300 individuals was planned to achieve 220 participants after expected attrition."	pg. 3 Statistical Analysis

Outcomes	**Descriptions as stated in report/paper**	**Location in text or source** (pg & ¶/fig/table/other)	
**Outcome name**	Sleep-Onset Latency (SOL)	pg. 2 Introduction	
**Outcome definition** (with diagnostic criteria if relevant)(include name, time, and analysis method)	SOL is the time it takes someone to fall asleep<br>1 year outcomes, ITT effect using t-tests	pg. 3 Statistical Analysis	
**Imputation of missing data** (e.g. assumptions made for ITT analysis)	No information about imputation		
**Power** (e.g. power & sample size calculation, level of power achieved)	Sample size was calculated based on an effect size between groups of Cohen d = 0.20 on each of the 3 primary sleep outcomes (ISI, SOL, andWASO) and an anticipated 35.0% attrition rate across the 1-year study. Powering the study at 80% to detect pre-post differences between groups with $\alpha = .05$ (using G*Power 3 software), a sample size of 300 individuals was planned to achieve 220 participants after expected attrition.	pg. 3 Statistical Analysis	
Outcomes	**Descriptions as stated in report/paper**	**Location in text or source** (pg & ¶/fig/table/other)	
**Outcome name**	Wake after sleep onset (WASO)	pg. 2 Introduction	
**Outcome definition** (with diagnostic criteria if relevant)(include name, time, and analysis method)	1 year outcomes, ITT effect using t-tests	pg. 3 Statistical Analysis	
**Imputation of missing data** (e.g. assumptions made for ITT analysis)	No information about imputation		
**Power** (e.g. power & sample size calculation, level of power achieved)	Sample size was calculated based on an effect size between groups of Cohen d = 0.20 on each of the 3 primary sleep outcomes (ISI, SOL, andWASO) and an anticipated 35.0% attrition rate across the 1-year study. Powering the study at 80% to detect pre-post differences between groups with $\alpha = .05$ (using G*Power 3 software), a sample size of 300 individuals was planned to achieve 220 participants after expected attrition.	pg. 3 Statistical Analysis	
**Risk of Bias** (Based on Cochrane RoB Tool 2 and ROBINS-I)		Link to RoB Algorithm	
**Primary Analysis RoB Assessment**		Notes	
**Is the primary analysis evaluating effectiveness or efficacy?**	Effectiveness		
**Comparator**	SHUTi vs Patient Education Group		
**Outcome Being Assessed**	ISI, SOL, WASO		
**Specify the Numerical Result Being Assessed**	ISI ($F3,1063 = 20.65, P < .001$)<br>SOL ($F3,1042 = 6.01, P < .001$)<br>WASO ($F3,1042 = 12.68, P < .001$).		
**Bias due to randomization (RCT only)**		Notes	
1.1 Was the allocation sequence random?	Yes	From supplemental material	
1.2. Was the allocation sequence concealed until participants were enrolled and assigned to interventions?	Yes	From supplemental material	
1.3. Did baseline differences between intervention groups suggest a problem with the randomization process?	No	There was one significantly different baseline covariate but that was likely due to random chance	
**Bias due to confounding**		Notes	
1.1 Is there potential for confounding of the effect of intervention in this study?	No	Randomized controlled trial used	
1.2. If Y/PY to 1.1 Was the analysis based on splitting participants' follow up time according to intervention received?			
1.3. If Y/PY to 1.2 Were intervention discontinuations or switches likely to be related to factors that are prognostic for the outcome?			
**Questions relating to baseline confounding only**			
1.4. If Y/PY to 1.1 Did the authors use an appropriate analysis method that controlled for all the important confounding domains?			
1.5. If Y/PY to 1.4: Were confounding domains that were controlled for measured validly and reliably by the variables available in this study?			
1.6. Did the authors control for any post-intervention variables that could have been affected by the intervention?			
**Questions relating to baseline and time-varying confounding**			
1.7. If Y/PY to 1.3 Did the authors use an appropriate analysis method that controlled for all the important confounding domains and for time-varying confounding?			
1.8. If Y/PY to 1.7: Were confounding domains that were controlled for measured validly and reliably by the variables available in this study?			
**Bias in selection of participants into the study (Not evaluating for analyses of effectiveness)**		Notes	Need to fill out
2.1. Was selection of participants into the analysis based on participant characteristics observed after the start of intervention?			
2.2. If Y/PY to 2.1: Were the post-intervention variables that influenced selection likely to be associated with intervention?			
2.3 If Y/PY to 2.2: Were the post-intervention variables that influenced selection likely to be influenced by the outcome or a cause of the outcome?			
2.4. Do start of follow-up and start of intervention coincide for most participants?			
2.5. If Y/PY to 2.2 and 2.3, or N/PN to 2.4: Were adjustment techniques used that are likely to correct for the presence of selection biases?			
**Bias in classification of interventions**		Notes	
3.1 Were intervention groups clearly defined? Was the definition of adherence clearly defined?	Yes		
3.2 Was the information used to define intervention groups recorded at the start of the intervention?	Yes	Definitions of interventions clearly stated in the preregistration	
3.3 Could classification of intervention status have been affected by knowledge of the outcome or risk of the outcome?	No		
**Bias due to deviations from intended interventions**		Notes	
**ROBINS-I**			
4.1. Were there deviations from the intended intervention beyond what would be expected in usual practice?	Probably No		
4.2. If Y/PY to 4.1: Were these deviations from intended intervention unbalanced between groups and likely to have affected the outcome?			
4.3. Were important co-interventions balanced across intervention groups?	Yes		
4.4. Was the intervention implemented successfully for most participants?	Yes	6.6% of participants did not complete first core	
4.5. Did study participants adhere to the assigned intervention regimen?	Probably No	60% completed 6 cores<br>70% completed 5 cores	
4.6. If N/PN to 4.3, 4.4 or 4.5: Was an appropriate analysis used to estimate the effect of starting and adhering to the intervention?	No	The required assumptions were not met	
Risk of bias: Assignment to intervention	**Low** / Moderate / Serious / Critical / NI		
Risk of bias: Starting and adhering to intervention	**Low** / Moderate / Serious / Critical / NI		
**Risk of bias judgement**	**Low** / Moderate / Serious / Critical / NI		
**RoB Tool (Risk of bias due to deviations from the intended interventions (effect of adhering to intervention))**			
2.1. Were participants aware of their assigned intervention during the trial?	Probably Yes	Participants were blinded but the study discloses that people were likely to know if they were in the treatment or control groups	
2.2. Were carers and people delivering the interventions aware of participants' assigned intervention during the trial?	No	Application delivered care with no physician involvement	
2.3. If Y/PY/NI to 2.1 or 2.2: Were important non protocol interventions balanced across intervention groups?	NA		
2.4. [If applicable:] Were there failures in implementing the intervention that could have affected the outcome?	Yes	6.6% of participants did not complete first core	
2.5. [If applicable:] Was there non-adherence to the assigned intervention regimen that could have affected participants' outcomes?	Yes	60% completed 6 cores<br>70% completed 5 cores	
2.6. If N/PN/NI to 2.3, or Y/PY/NI to 2.4 or 2.5: Was an appropriate analysis used to estimate the effect of adhering to the intervention?	No	The required assumptions were not met	
**Risk of bias judgement**	Low/**High**/Some Concerns		
**Bias due to missing data**		Notes	
5.1 Were outcome data available for all, or nearly all, participants?	Yes	Missing data for 55 participants	
5.2 Were participants excluded due to missing data on intervention status?	No		

5.3 Were participants excluded due to missing data on other variables needed for the analysis?	No		
5.4 If PN/N to 5.1, or Y/PY to 5.2 or 5.3: Are the proportion of participants and reasons for missing data similar across interventions?			
5.5 If PN/N to 5.1, or Y/PY to 5.2 or 5.3: Is there evidence that results were robust to the presence of missing data?		Data was not imputed but this likely did not have a large impact	
**Bias in measurement of outcomes**		Notes	
6.1 Could the outcome measure have been influenced by knowledge of the intervention received?	Yes		
6.2 Were outcome assessors aware of the intervention received by study participants? (blinding of assessors)	Yes	Participants self-reported their symptoms and were likely aware of the intervention despite the control intervention	
6.3 Were the methods of outcome assessment comparable across intervention groups?	Yes		
6.4 Were any systematic errors in measurement of the outcome related to intervention received?	No		
**Bias in selection of the reported result**		Notes	
Were the data that produced this result analysed in accordance with a pre-specified analysis plan that was finalized before unblinded outcome data were available for analysis?	Yes	Preregistered on clinicaltrials.gov and the analysis plan was uploaded as supplemental material	
Is the reported effect estimate likely to be selected, on the basis of the results, from...			
7.1. ... multiple outcome measurements within the outcome domain?	No		
7.2 ... multiple analyses of the intervention-outcome relationship?	No		
7.3 ... different subgroups?	No		
**Secondary Analysis RoB Assessment**		Notes	
**Is the seconday analysis evaluating effectiveness or efficacy?**	Efficacy		
**Comparator**	SHUTi vs Patient Education Group		
**Outcome Being Assessed**	Estimated marginal mean given core completion		
**Specify the Numerical Result Being Assessed**	At the 6-month follow-up and 1-year follow-up, those who completed all 6 cores had EMM (SD) ISIs of 7.90 (0.53) and 6.91 (0.57), respectively, while those who did not complete all 6 cores had EMM (SD) ISIs of 9.98 (0.97) and 8.56 (0.93), respectively.		Check if quotes are needed
**Bias due to randomization (See primary analysis section)**		Notes	
**Bias due to confounding**		Notes	
1.1 Is there potential for confounding of the effect of intervention in this study?	Yes		
1.2. If Y/PY to 1.1 Was the analysis based on splitting participants' follow up time according to intervention received?	No		
1.3. If Y/PY to 1.2 Were intervention discontinuations or switches likely to be related to factors that are prognostic for the outcome?			
**Questions relating to baseline confounding only**			
1.4. If Y/PY to 1.1 Did the authors use an appropriate analysis method that controlled for all the important confounding domains?	Probably No	Baseline ISI was controlled for but the authors did not say why	
1.5. If Y/PY to 1.4: Were confounding domains that were controlled for measured validly and reliably by the variables available in this study?			
1.6. Did the authors control for any post-intervention variables that could have been affected by the intervention?	No		
**Questions relating to baseline and time-varying confounding**			
1.7. If Y/PY to 1.3 Did the authors use an appropriate analysis method that controlled for all the important confounding domains and for time-varying confounding?			
1.8. If Y/PY to 1.7: Were confounding domains that were controlled for measured validly and reliably by the variables available in this study?			
**Bias in selection of participants into the study (Not evaluating for analyses of effectiveness)**		Notes	
2.1. Was selection of participants into the analysis based on participant characteristics observed after the start of intervention?	No		
2.2. If Y/PY to 2.1: Were the post-intervention variables that influenced selection likely to be associated with intervention?			
2.3 If Y/PY to 2.2: Were the post-intervention variables that influenced selection likely to be influenced by the outcome or a cause of the outcome?			
2.4. Do start of follow-up and start of intervention coincide for most participants?	Yes		
2.5. If Y/PY to 2.2 and 2.3, or N/PN to 2.4: Were adjustment techniques used that are likely to correct for the presence of selection biases?			
**Bias in classification of interventions**		Notes	
3.1 Were intervention groups clearly defined?	Yes	Clear definition of adherence	
3.2 Was the information used to define intervention groups recorded at the start of the intervention?	Probably no	It does not appear that the definition of adherence was preregistered	
3.3 Could classification of intervention status have been affected by knowledge of the outcome or risk of the outcome?	Yes	It is possible given that this analysis was not preregistered	
**Bias due to deviations from intended interventions (See primary analysis section)**		Notes	
**Bias due to missing data**		Notes	
5.1 Were outcome data available for all, or nearly all, participants?	Probably Not	30 partipants dropped out from the treatment group	
5.2 Were participants excluded due to missing data on intervention status?	No		
5.3 Were participants excluded due to missing data on other variables needed for the analysis?	No		
5.4 If PN/N to 5.1, or Y/PY to 5.2 or 5.3: Are the proportion of participants and reasons for missing data similar across interventions?	No Information	It doesn't seem that they considered if missingness was related to adherence	
5.5 If PN/N to 5.1, or Y/PY to 5.2 or 5.3: Is there evidence that results were robust to the presence of missing data?	No		
**Bias in measurement of outcomes**		Notes	
6.1 Could the outcome measure have been influenced by knowledge of the intervention received?	Probably No	The long follow-up time likely reduced the likelihood of a placebo or white coat effect	
6.2 Were outcome assessors aware of the intervention received by study participants? (blinding of assessors)	Yes		
6.3 Were the methods of outcome assessment comparable across intervention groups?	Yes		
6.4 Were any systematic errors in measurement of the outcome related to intervention received?	No		
6.5 Could adherence have been influenced by study participation?	Probably No	There was not a check-in during the 9-week treatment period	
**Bias in selection of the reported result**		Notes	
Were the data that produced this result analysed in accordance with a pre-specified analysis plan that was finalized before unblinded outcome data were available for analysis?	Probably no	The adherence analysis was not part of the preregistered analysis plan	
Is the reported effect estimate likely to be selected, on the basis of the results, from...			
7.1. ... multiple outcome measurements within the outcome domain?	No	This was part of the preregistered plan	
7.2 ... multiple analyses of the intervention-outcome relationship?	Probably Yes	Only the ISI outcome was reported instead of all three outcomes	
7.3 ... different subgroups?	No information	Unclear since this was not a preregistered analysis	

Criteria for Adherence Metrics and Analysis		Notes
**Device name and indication for use**	Natural Cycles (contraception)	
**Type of device** (Long-term vs short-term use)	Long-term use	
**Type of device** (Presence or absence of a prescribed dosage)	Presence of a prescribed dose	
**Was adherence information collected?**	Yes	
**What information was collected about usage adherence?**	Frequency of data input into the app	Data input could include BBT, LH, menstruation, and sexual activity
**What information was collected about adherence to recommendations?**	Sexual activity for cycles where pregancy occured<br>Frequency of unprotected sex on red days	
**Was information collected internally in the app?**	Yes	
**Was information modified internally?**	NA	
**Was initiation reported?**	Yes	Participants were included in the study if they provided 20 days of data input
**What was average adherence?** (if reported)	100%	
**Was implementation reported?**	Yes	
**What was average adherence?** (if reported)	Participants added data on 63% of the days (88% of this data was temperature data); 51% of women reported unprotected intercourse for cycles where pregnancy occurred; [67% of women reported using protection on red days in a survey (not a continuous measurement)]	
**Was persistence reported?**	Yes	
**What was average adherence?** (if reported)	34% of women discontinued app use prior to the end of the study (66% used the app at the end of the study)	
**Was adherence low?**	Yes	Adherence for data input was less than 80%
**Was adherence not reported or low?**	Yes	
**Was efficacy analyzed?**	No	
**What method was used?**	NA	
**Were efficacy analyses preregistered?**		
**What assumptions are required for that method to study efficacy?**		
**Did the article report evidence that the assumptions were met?**		
**SUTVA**		
**Positivity**		
**Consistency** (treatment definition)		
**Consistency** (adherence definition)		
**Exclusion restriction**		
**Strong Monotonicity**		
**Ignorability**		
**Overall Notes**		
**Cochrane CDPLG**		**Notes**
**Data form completed date** (dd/mm/yyyy)	4/5/2022	
**Study author contact details**	raoul.scherwitzl@naturalcycles.com	
Methods	**Descriptions as stated in report/paper**	**Location in text or source** (pg & ¶/fig/table/other)
Aim of study (e.g. efficacy, equivalence, pragmatic)	Effectiveness	pg. 1 Introduction
Design (e.g. parallel, crossover, non-RCT)	Observational Cohort Trial (retrospective and prospective aspects)	pg. 2 Study Design
Unit of allocation (by individuals, cluster/ groups or body parts)	Individual	
Participants	**Descriptions as stated in report/paper**	**Location in text or source** (pg & ¶/fig/table/other)
**Inclusion criteria**	"The participant had to have access to the application for at least three months during the study period.<br><br>The participant had to enter data for at least 20 days in total. Each daily data point can be any kind of combination of the possible entries (menstruation, temperature, LH test results, sexual activity or a personal note) for a specific date.<br><br>The participant had to be older than 18 years and not planning a pregnancy during the study period"	pg. 2 Study Design
**Exclusion criteria**	"women with a medical condition in whom becoming pregnant would be dangerous to them or their fetus"	pg. 2 Study Design
**Total no. randomised** (or total pop. at start of study for NRCTs)	4054	pg. 2 Study Design
**Clusters** (if applicable, no., type, no. people per cluster)	NA	
**Withdrawals and exclusions** (if not provided below by outcome)	1397 women stopped using the app prior to study completion (not sure how many responded to follow-up)	pg. 4 Results
Intervention Groups	**Descriptions as stated in report/paper**	**Location in text or source** (pg & ¶/fig/table/other)
**Group name**	Natural Cycles	pg. 1 Introduction
**No. randomised to group** (specify whether no. people or clusters)	4054	pg. 2 Study Design
**Timing** (e.g. frequency, duration of each episode)	Daily entries of temperature and option entries of LH, menstruation, and sexual activity	pg. 1 Digital Fertility Monitor
**Co-interventions**	Backup contraception use on red days	pg. 2 Digital Fertility Monitor
**Integrity of delivery**	Participants had to provide 20 days of data to be included in the study	pg. 2 Study Design
**Compliance**	Participants added data on 63% of the days (88% of this data was temperature data)	pg. 3 Results
Outcomes	**Descriptions as stated in report/paper**	**Location in text or source** (pg & ¶/fig/table/other)
**Outcome name**	Typical Use Pearl Index	pg. 2 Pearl Index
**Outcome definition** (with diagnostic criteria if relevant)(include name, time, and analysis method)	Typical use pearl index for one year of use	pg. 2 Pearl Index
**Imputation of missing data** (e.g. assumptions made for ITT analysis)	"The possibility of pregnancy is detected by scrutinising the data to search for a combination of: (1) delayed menstruation, identified by comparing the time elapsed since the last ovulation with the user's normal luteal phase duration; and (2) consistently high temperature levels, as progesterone increases rather than decreases in the body if a fertilised egg has been implanted in the uterus."	pg. 2 Digital Fertility Monitor
**Power** (e.g. power & sample size calculation, level of power achieved)		
**Risk of Bias** (Based on Cochrane RoB Tool 2 and ROBINS-I)		Link to RoB Algorithm
**Primary Analysis RoB Assessment**		Notes
**Is the primary analysis evaluating effectiveness or efficacy?**	Effectiveness	
**Comparator**	No comparator	
**Outcome Being Assessed**	Typical use pearl index	
**Specify the Numerical Result Being Assessed**	"Excluding these women [61 dropout cases with unknown pregnancy status] from the study gives a Pearl Index of 7.0 for typical use. To estimate the most conservative upper limit of the Pearl Index, all unknown cases were considered as pregnant, yielding a value of 9.8"	
**Bias due to randomization (RCT only)**		Notes
1.1 Was the allocation sequence random?	NA	
1.2. Was the allocation sequence concealed until participants were enrolled and assigned to interventions?	NA	
1.3. Did baseline differences between intervention groups suggest a problem with the randomization process?	NA	
**Bias due to confounding**		Notes
1.1 Is there potential for confounding of the effect of intervention in this study?	Yes	
1.2. If Y/PY to 1.1 Was the analysis based on splitting participants' follow up time according to intervention received?	No	
1.3. If Y/PY to 1.2 Were intervention discontinuations or switches likely to be related to factors that are prognostic for the outcome?		
**Questions relating to baseline confounding only**		

Article Key

1.4. If Y/PY to 1.1 Did the authors use an appropriate analysis method that controlled for all the important confounding domains?	No	Researchers did not do subgroup analyses based on demographics and their follow-up survey and analysis found that some women likely became interested in planning a pregnancy during the study period	
1.5. If Y/PY to 1.4: Were confounding domains that were controlled for measured validly and reliably by the variables available in this study?			
1.6. Did the authors control for any post-intervention variables that could have been affected by the intervention?	No		
**Questions relating to baseline and time-varying confounding**			
1.7. If Y/PY to 1.3 Did the authors use an appropriate analysis method that controlled for all the important confounding domains and for time-varying confounding?			
1.8. If Y/PY to 1.7: Were confounding domains that were controlled for measured validly and reliably by the variables available in this study?			
**Bias in selection of participants into the study (Not evaluating for analyses of effectiveness)**		Notes	Need to fill in
2.1. Was selection of participants into the analysis based on participant characteristics observed after the start of intervention?			
2.2. If Y/PY to 2.1: Were the post-intervention variables that influenced selection likely to be associated with intervention?			
2.3 If Y/PY to 2.2: Were the post-intervention variables that influenced selection likely to be influenced by the outcome or a cause of the outcome?			
2.4. Do start of follow-up and start of intervention coincide for most participants?			
2.5. If Y/PY to 2.2 and 2.3, or N/PN to 2.4: Were adjustment techniques used that are likely to correct for the presence of selection biases?			
**Bias in classification of interventions**		Notes	
3.1 Were intervention groups clearly defined? Was the definition of adherence clearly defined?	Yes	Clear definition of the intervention	
3.2 Was the information used to define intervention groups recorded at the start of the intervention?	No	This study was not preregistered	
3.3 Could classification of intervention status have been affected by knowledge of the outcome or risk of the outcome?	No	Only one cohort in this study	
**Bias due to deviations from intended interventions**		Notes	
**ROBINS-I**			
4.1. Were there deviations from the intended intervention beyond what would be expected in usual practice?	No		
4.2. If Y/PY to 4.1: Were these deviations from intended intervention unbalanced between groups and likely to have affected the outcome?			
4.3. Were important co-interventions balanced across intervention groups?	NA		
4.4. Was the intervention implemented successfully for most participants?	Yes	Participants were included in the study if they provided 20 days of data input	
4.5. Did study participants adhere to the assigned intervention regimen?	No	Participants input data on 63% of the days of which 88% were temperature readings	
4.6. If N/PN to 4.3, 4.4 or 4.5: Was an appropriate analysis used to estimate the effect of starting and adhering to the intervention?	No	Researchers analyzed method failure instead of perfect use	
Risk of bias: Assignment to intervention	**Low** / Moderate / Serious / Critical / NI		
Risk of bias: Starting and adhering to intervention	Low / Moderate / Serious / **Critical** / NI		
**Risk of bias judgement**	Low / Moderate / Serious / **Critical** / NI		
**RoB Tool (Risk of bias due to deviations from the intended interventions (effect of adhering to intervention))**			
2.1. Were participants aware of their assigned intervention during the trial?	Yes		
2.2. Were carers and people delivering the interventions aware of participants' assigned intervention during the trial?	NA		
2.3. If Y/PY/NI to 2.1 or 2.2: Were important non protocol interventions balanced across intervention groups?	NA		
2.4. [If applicable:] Were there failures in implementing the intervention that could have affected the outcome?	No	Participants were included in the study if they provided 20 days of data input	
2.5. [If applicable:] Was there non-adherence to the assigned intervention regimen that could have affected participants' outcomes?	Yes	Participants input data on 63% of the days of which 88% were temperature readings	
2.6. If N/PN/NI to 2.3, or Y/PY/NI to 2.4 or 2.5: Was an appropriate analysis used to estimate the effect of adhering to the intervention?	No	Researchers analyzed method failure instead of perfect use	
**Risk of bias judgement**	Low/**High**/Some Concerns		
**Bias due to missing data**		Notes	
5.1 Were outcome data available for all, or nearly all, participants?	No information	34% of women dropped before the study was over but they don't report how many women responded to the follow-up survey	
5.2 Were participants excluded due to missing data on intervention status?	No		
5.3 Were participants excluded due to missing data on other variables needed for the analysis?	No		
5.4 If PN/N to 5.1, or Y/PY to 5.2 or 5.3: Are the proportion of participants and reasons for missing data similar across interventions?	NA		
5.5 If PN/N to 5.1, or Y/PY to 5.2 or 5.3: Is there evidence that results were robust to the presence of missing data?	Probably Yes	Researchers were able to classify 98.5% of women based on data they had entered in the app prior to drop out but this makes the assumption that 100% of the women who were categorized as not pregnant by the app were not pregnant while they recognize that method failure does happen	
**Bias in measurement of outcomes**		Notes	
6.1 Could the outcome measure have been influenced by knowledge of the intervention received?	Probably No		
6.2 Were outcome assessors aware of the intervention received by study participants? (blinding of assessors)	Yes		
6.3 Were the methods of outcome assessment comparable across intervention groups?	NA		
6.4 Were any systematic errors in measurement of the outcome related to intervention received?	Probably Yes	Researchers relied on self-reported pregnancy and app data to classify pregnancy instead of clinic testing that would normally occur during controlled trials<br>Missing data were categorized as pregnant or not based on tempterature readings	
**Bias in selection of the reported result**		Notes	
Were the data that produced this result analysed in accordance with a pre-specified analysis plan that was finalized before unblinded outcome data were available for analysis?	No		
Is the reported effect estimate likely to be selected, on the basis of the results, from...			
7.1. ... multiple outcome measurements within the outcome domain?	Probably No	Researchers only looked at pregnancy but could have looked at different methods of measuring/identifying pregnancy	
7.2 ... multiple analyses of the intervention-outcome relationship?	Probably No	Researchers used two standard analysis methods for calculating the typical use effectiveness and reported a range in effectiveness based on sensitivity analyses and emphasized the more conservative estimate when comparing the PI to the 12 cycle Kaplan-Meier estimate	
7.3 ... different subgroups?	No		
**Secondary Analysis RoB Assessment**		Notes	

**Is the seconday analysis evaluating effectiveness or efficacy?**		
**Comparator**		
**Outcome Being Assessed**		
**Specify the Numerical Result Being Assessed**		
**Bias due to randomization (See primary analysis section)**		Notes
**Bias due to confounding**		Notes
1.1 Is there potential for confounding of the effect of intervention in this study?		
1.2. If Y/PY to 1.1 Was the analysis based on splitting participants' follow up time according to intervention received?		
1.3. If Y/PY to 1.2 Were intervention discontinuations or switches likely to be related to factors that are prognostic for the outcome?		
**Questions relating to baseline confounding only**		
1.4. If Y/PY to 1.1 Did the authors use an appropriate analysis method that controlled for all the important confounding domains?		
1.5. If Y/PY to 1.4: Were confounding domains that were controlled for measured validly and reliably by the variables available in this study?		
1.6. Did the authors control for any post-intervention variables that could have been affected by the intervention?		
**Questions relating to baseline and time-varying confounding**		
1.7. If Y/PY to 1.3 Did the authors use an appropriate analysis method that controlled for all the important confounding domains and for time-varying confounding?		
1.8. If Y/PY to 1.7: Were confounding domains that were controlled for measured validly and reliably by the variables available in this study?		
**Bias in selection of participants into the study (Not evaluating for analyses of effectiveness)**		Notes
2.1. Was selection of participants into the analysis based on participant characteristics observed after the start of intervention?		
2.2. If Y/PY to 2.1: Were the post-intervention variables that influenced selection likely to be associated with intervention?		
2.3 If Y/PY to 2.2: Were the post-intervention variables that influenced selection likely to be influenced by the outcome or a cause of the outcome?		
2.4. Do start of follow-up and start of intervention coincide for most participants?		
2.5. If Y/PY to 2.2 and 2.3, or N/PN to 2.4: Were adjustment techniques used that are likely to correct for the presence of selection biases?		
**Bias in classification of interventions**		Notes
3.1 Were intervention groups clearly defined?		
3.2 Was the information used to define intervention groups recorded at the start of the intervention?		
3.3 Could classification of intervention status have been affected by knowledge of the outcome or risk of the outcome?		
**Bias due to deviations from intended interventions (See primary analysis section)**		Notes
**Bias due to missing data**		Notes
5.1 Were outcome data available for all, or nearly all, participants?		
5.2 Were participants excluded due to missing data on intervention status?		
5.3 Were participants excluded due to missing data on other variables needed for the analysis?		
5.4 If PN/N to 5.1, or Y/PY to 5.2 or 5.3: Are the proportion of participants and reasons for missing data similar across interventions?		
5.5 If PN/N to 5.1, or Y/PY to 5.2 or 5.3: Is there evidence that results were robust to the presence of missing data?		
**Bias in measurement of outcomes**		Notes
6.1 Could the outcome measure have been influenced by knowledge of the intervention received?		
6.2 Were outcome assessors aware of the intervention received by study participants? (blinding of assessors)		
6.3 Were the methods of outcome assessment comparable across intervention groups?		
6.4 Were any systematic errors in measurement of the outcome related to intervention received?		
6.5 Could adherence have been influenced by study participation?		
**Bias in selection of the reported result**		Notes
Were the data that produced this result analysed in accordance with a pre-specified analysis plan that was finalized before unblinded outcome data were available for analysis?		
Is the reported effect estimate likely to be selected, on the basis of the results, from...		
7.1. ... multiple outcome measurements within the outcome domain?		
7.2 ... multiple analyses of the intervention-outcome relationship?		
7.3 ... different subgroups?		

Criteria for Adherence Metrics and Analysis		Notes	
**Device name and indication for use**	Natural Cycles (contraception)		Article Key
**Type of device** (Long-term vs short-term use)	Long-term use		
**Type of device** (Presence or absence of a prescribed dosage)	Presence of a prescribed dose		
**Was adherence information collected?**	Yes		
**What information was collected about usage adherence?**	Daily log of data input	Data input could include menstruation, BBT, LH test, pregnancy test result, sexual activity and personal notes	
**What information was collected about adherence to recommendations?**	Sexual activity and use of backup protection on red days		
**Was information collected internally in the app?**	Yes		
**Was information modified internally?**	NA		
**Was initiation reported?**	Yes		
**What was average adherence?** (if reported)	97%	778 participants were not included because they did not log any information	
**Was implementation reported?**	Yes		
**What was average adherence?** (if reported)	They report that 6.9% of cycles were perfect use meaning that there were reports of sexual activity on fertile days	They do not report how report on implementation for input of data into the app	
**Was persistence reported?**	Yes		
**What was average adherence?** (if reported)	46% were using the app at the end of a one year period		
**Was adherence low?**	Yes	Very low percentage of cycles were perfect use	
**Was adherence not reported or low and could have affected outcomes?**	Yes		
**Was efficacy analyzed?**	Yes		
**What method was used?**	Per-protocol analysis	Pearl index for perfect use	
**Were efficacy analyses preregistered?**	No		
**What assumptions are required for that method to study efficacy?**	SUTVA, positivity, consistency, ignorability, conditional independence of adherence and outcomes		
**Did the article report evidence that the assumptions were met?**	No		
**SUTVA**	Yes	Treatment was only administered through the app	
**Positivity**	No	No control condition	
**Consistency** (treatment definition)	Yes	Clear definition of treatment	
**Consistency** (adherence definition)	Yes	Clear definition of adherence	
**Exclusion restriction**			
**Strong Monotonicity**			
**Ignorability**	None		
**Overall Notes**			
**Cochrane CDPLG**		**Notes**	
**Data form completed date** (dd/mm/yyyy)	2/1/2022		
**Study author contact details**	raoul.scherwitzl@naturalcycles.com		
Methods	**Descriptions as stated in report/paper**	**Location in text or source** (pg & ¶/fig/table/other)	
Aim of study (e.g. efficacy, equivalence, pragmatic)	Effectiveness and Efficacy	pg. 1 Abstract	
Design (e.g. parallel, crossover, non-RCT)	Prospective Observational Study	pg. 1 Abstract	
Unit of allocation (by individuals, cluster/ groups or body parts)	Individual	pg. 2 Study Design	
Participants	**Descriptions as stated in report/paper**	**Location in text or source** (pg & ¶/fig/table/other)	
**Inclusion criteria**	Participants had to log 20 days of activity	pg. 2 Study Design	
**Exclusion criteria**	Excluded people who later reported they intended to become pregnant	pg. 3 Study Design	
**Total no. randomised** (or total pop. at start of study for NRCTs)	22785	pg. 3 Results	
**Clusters** (if applicable, no., type, no. people per cluster)	NA		
**Withdrawals and exclusions** (if not provided below by outcome)	54% of participants discontinued using the app within a year	pg. 4 Results	
Intervention Groups	**Descriptions as stated in report/paper**	**Location in text or source** (pg & ¶/fig/table/other)	
**Group name**	Natural Cycles Cohort		
**No. randomised to group** (specify whether no. people or clusters)	22785	pg. 3 Results	
**Timing** (e.g. frequency, duration of each episode)	No explicit timing listed but it is implied that data input is expect on a daily basis: "such a daily log can contain…"	pg. 2 Study Design	
**Co-interventions**	Participants could use other forms of contraception throughout. Optionally reported this co-intervention on red days. (Researchers do not explicitly state in this study that users were instructed to use backup contraception on red days)	pg. 2 The Mobile Application	
**Integrity of delivery**			
**Compliance**	54% of women discontinued the app in a year. No clear recommended dosage of the app	pg. 4 Results	
Outcomes	**Descriptions as stated in report/paper**	**Location in text or source** (pg & ¶/fig/table/other)	
**Outcome name**	Pregnancy	pg. 2 Study Design	
**Outcome definition** (with diagnostic criteria if relevant)(include name, time, and analysis method)	Measured 13-cycle pregnancy rate and one year pearl index		
**Imputation of missing data** (e.g. assumptions made for ITT analysis)	Assumed that all women who discontinued between menstruation and ovulation as not pregnant, assumed women were likely pregnant if they discontinued in the late luteal phase or reported a high BBT at discontinuation, if there was not enough information they assumed women were possibly pregnant but considered them not pregnant for the primary analysis	pg. 2 Study Design	
**Power** (e.g. power & sample size calculation, level of power achieved)	No power or sample size calculation		
**Risk of Bias** (Based on Cochrane RoB Tool 2 and ROBINS-I)		Link to RoB Algorithm	
**Primary Analysis RoB Assessment**		Notes	
**Is the primary analysis evaluating effectiveness or efficacy?**	Effectiveness		
**Comparator**	No comparator		
**Outcome Being Assessed**	Pregnancy rate analyzed with kaplan meier life tables over 13 cycles		
**Specify the Numerical Result Being Assessed**	typical use pregnancy rate 8.3%		
**Bias due to randomization (RCT only)**		Notes	
1.1 Was the allocation sequence random?	NA		
1.2. Was the allocation sequence concealed until participants were enrolled and assigned to interventions?	NA		
1.3. Did baseline differences between intervention groups suggest a problem with the randomization process?	NA		
**Bias due to confounding**		Notes	
1.1 Is there potential for confounding of the effect of intervention in this study?	Yes		
1.2. If Y/PY to 1.1 Was the analysis based on splitting participants' follow up time according to intervention received?	No		
1.3. If Y/PY to 1.2 Were intervention discontinuations or switches likely to be related to factors that are prognostic for the outcome?			
**Questions relating to baseline confounding only**			
1.4. If Y/PY to 1.1 Did the authors use an appropriate analysis method that controlled for all the important confounding domains?	No		
1.5. If Y/PY to 1.4: Were confounding domains that were controlled for measured validly and reliably by the variables available in this study?			
1.6. Did the authors control for any post-intervention variables that could have been affected by the intervention?	No		
**Questions relating to baseline and time-varying confounding**			
1.7. If Y/PY to 1.3 Did the authors use an appropriate analysis method that controlled for all the important confounding domains and for time-varying confounding?			
1.8. If Y/PY to 1.7: Were confounding domains that were controlled for measured validly and reliably by the variables available in this study?			
**Bias in selection of participants into the study (Not evaluating for analyses of effectiveness)**		Notes	Need to fill out
2.1. Was selection of participants into the analysis based on participant characteristics observed after the start of intervention?			
2.2. If Y/PY to 2.1: Were the post-intervention variables that influenced selection likely to be associated with intervention?			

2.3 If Y/PY to 2.2: Were the post-intervention variables that influenced selection likely to be influenced by the outcome or a cause of the outcome?		
2.4. Do start of follow-up and start of intervention coincide for most participants?		
2.5. If Y/PY to 2.2 and 2.3, or N/PN to 2.4: Were adjustment techniques used that are likely to correct for the presence of selection biases?		
**Bias in classification of interventions**		Notes
3.1 Were intervention groups clearly defined? Was the definition of adherence clearly defined?	Yes	Clear definition of the intervention
3.2 Was the information used to define intervention groups recorded at the start of the intervention?	No	This study was not preregistered
3.3 Could classification of intervention status have been affected by knowledge of the outcome or risk of the outcome?	No	Only one cohort in this study
**Bias due to deviations from intended interventions**		Notes
**ROBINS-I**		
4.1. Were there deviations from the intended intervention beyond what would be expected in usual practice?	No	
4.2. If Y/PY to 4.1: Were these deviations from intended intervention unbalanced between groups and likely to have affected the outcome?	NA	
4.3. Were important co-interventions balanced across intervention groups?	NA	
4.4. Was the intervention implemented successfully for most participants?	Yes	
4.5. Did study participants adhere to the assigned intervention regimen?	No	Unclear how often women logged information in the app and only 9.6% of cycles were considered "perfect use"
4.6. If N/PN to 4.3, 4.4 or 4.5: Was an appropriate analysis used to estimate the effect of starting and adhering to the intervention?	No	The assumptions needed for their method of efficacy analysis were not met
Risk of bias: Assignment to intervention	**Low** / Moderate / Serious / Critical / NI	
Risk of bias: Starting and adhering to intervention	Low / Moderate / Serious / **Critical** / NI	
**Risk of bias judgement**	Low / Moderate / Serious / **Critical** / NI	
**RoB Tool (Risk of bias due to deviations from the intended interventions (effect of adhering to intervention))**		
2.1. Were participants aware of their assigned intervention during the trial?	Yes	
2.2. Were carers and people delivering the interventions aware of participants' assigned intervention during the trial?	NA	
2.3. If Y/PY/NI to 2.1 or 2.2: Were important non protocol interventions balanced across intervention groups?	NA	
2.4. [If applicable:] Were there failures in implementing the intervention that could have affected the outcome?	Yes	
2.5. [If applicable:] Was there non-adherence to the assigned intervention regimen that could have affected participants' outcomes?	Yes	Unclear how often women logged information in the app and only 9.6% of cycles were considered "perfect use"
2.6. If N/PN/NI to 2.3, or Y/PY/NI to 2.4 or 2.5: Was an appropriate analysis used to estimate the effect of adhering to the intervention?	No	The assumptions needed for their method of efficacy analysis were not met
**Risk of bias judgement**	Low/**High**/Some Concerns	
**Bias due to missing data**		Notes
5.1 Were outcome data available for all, or nearly all, participants?	No	
5.2 Were participants excluded due to missing data on intervention status?	No	
5.3 Were participants excluded due to missing data on other variables needed for the analysis?	No	
5.4 If PN/N to 5.1, or Y/PY to 5.2 or 5.3: Are the proportion of participants and reasons for missing data similar across interventions?	NA	
5.5 If PN/N to 5.1, or Y/PY to 5.2 or 5.3: Is there evidence that results were robust to the presence of missing data?	Probably Yes	They did not fully report how many people had missing data but they use a method of classifying women as pregnant or not based on a (likely) accurate method
**Bias in measurement of outcomes**		Notes
6.1 Could the outcome measure have been influenced by knowledge of the intervention received?	No	
6.2 Were outcome assessors aware of the intervention received by study participants? (blinding of assessors)	Yes	
6.3 Were the methods of outcome assessment comparable across intervention groups?	NA	
6.4 Were any systematic errors in measurement of the outcome related to intervention received?	Probably Yes	They categorized women with missing data as pregnant or not based on temperature readings
**Bias in selection of the reported result**		Notes
Were the data that produced this result analysed in accordance with a pre-specified analysis plan that was finalized before unblinded outcome data were available for analysis?	No	
Is the reported effect estimate likely to be selected, on the basis of the results, from...		
7.1. ... multiple outcome measurements within the outcome domain?	No	
7.2 ... multiple analyses of the intervention-outcome relationship?	Probably No	
7.3 ... different subgroups?	No	
**Secondary Analysis RoB Assessment**		Notes
**Is the seconday analysis evaluating effectiveness or efficacy?**	Efficacy	
**Comparator**	No comparator	
**Outcome Being Assessed**	Perfect-use pregnancy rate analyzed with Pearl Index	
**Specify the Numerical Result Being Assessed**	1%	
**Bias due to randomization (See primary analysis section)**		Notes
**Bias due to confounding**		Notes
1.1 Is there potential for confounding of the effect of intervention in this study?	Yes	
1.2. If Y/PY to 1.1 Was the analysis based on splitting participants' follow up time according to intervention received?	No	
1.3. If Y/PY to 1.2 Were intervention discontinuations or switches likely to be related to factors that are prognostic for the outcome?		
**Questions relating to baseline confounding only**		
1.4. If Y/PY to 1.1 Did the authors use an appropriate analysis method that controlled for all the important confounding domains?	No	
1.5. If Y/PY to 1.4: Were confounding domains that were controlled for measured validly and reliably by the variables available in this study?		
1.6. Did the authors control for any post-intervention variables that could have been affected by the intervention?	No	
**Questions relating to baseline and time-varying confounding**		
1.7. If Y/PY to 1.3 Did the authors use an appropriate analysis method that controlled for all the important confounding domains and for time-varying confounding?		
1.8. If Y/PY to 1.7: Were confounding domains that were controlled for measured validly and reliably by the variables available in this study?		
**Bias in selection of participants into the study (Not evaluating for analyses of effectiveness)**		Notes
2.1. Was selection of participants into the analysis based on participant characteristics observed after the start of intervention?	Yes	
2.2. If Y/PY to 2.1: Were the post-intervention variables that influenced selection likely to be associated with intervention?	Yes	Participants who inputted more temperature readings and sexual behavior info were more likely to be selected into this analysis
2.3 If Y/PY to 2.2: Were the post-intervention variables that influenced selection likely to be influenced by the outcome or a cause of the outcome?	Yes	If participants inputted more temperature info and more sexual behavior info the app was more likely to be accurate and other confounders could have caused them to have fewer pregnancies

2.4. Do start of follow-up and start of intervention coincide for most participants?	Probably Yes	
2.5. If Y/PY to 2.2 and 2.3, or N/PN to 2.4: Were adjustment techniques used that are likely to correct for the presence of selection biases?	No	
**Bias in classification of interventions**		Notes
3.1 Were intervention groups clearly defined?	Yes	
3.2 Was the information used to define intervention groups recorded at the start of the intervention?	Probably No	Not preregistered
3.3 Could classification of intervention status have been affected by knowledge of the outcome or risk of the outcome?	No	
**Bias due to deviations from intended interventions (See primary analysis section)**		Notes
**Bias due to missing data**		Notes
5.1 Were outcome data available for all, or nearly all, participants?	Probably Yes	For the cycles included in these analyses there was likely full outcome information
5.2 Were participants excluded due to missing data on intervention status?	Probably Yes	Participants were evaluated based on sexual activity info which they were not required to log
5.3 Were participants excluded due to missing data on other variables needed for the analysis?	NA	
5.4 If PN/N to 5.1, or Y/PY to 5.2 or 5.3: Are the proportion of participants and reasons for missing data similar across interventions?		
5.5 If PN/N to 5.1, or Y/PY to 5.2 or 5.3: Is there evidence that results were robust to the presence of missing data?		
**Bias in measurement of outcomes**		Notes
6.1 Could the outcome measure have been influenced by knowledge of the intervention received?	No	
6.2 Were outcome assessors aware of the intervention received by study participants? (blinding of assessors)	Yes	
6.3 Were the methods of outcome assessment comparable across intervention groups?	Probably Yes	
6.4 Were any systematic errors in measurement of the outcome related to intervention received?	Probably Yes	They categorized women with missing data as pregnant or not based on temperature readings
6.5 Could adherence have been influenced by study participation?	No	
**Bias in selection of the reported result**		Notes
Were the data that produced this result analysed in accordance with a pre-specified analysis plan that was finalized before unblinded outcome data were available for analysis?	No	
Is the reported effect estimate likely to be selected, on the basis of the results, from...		
7.1. ... multiple outcome measurements within the outcome domain?	No	
7.2 ... multiple analyses of the intervention-outcome relationship?	Probably No	
7.3 ... different subgroups?	No	

Article Key

Criteria for Adherence Metrics and Analysis		Notes
**Device name and indication for use**		
**Type of device** (Chronic vs acute use)		
**Type of device** (Presence or absence of a prescribed dosage)		
**Was adherence information collected?**		
**What information was collected about usage adherence?**		
**What information was collected about adherence to recommendations?**		
**Was information collected internally in the app?**		
**Was information modified internally?**		
**Was initiation reported?**		
**What was average adherence?** (if reported)		
**Was implementation reported?**		
**What was average adherence?** (if reported)		
**Was persistence reported?**		
**What was average adherence?** (if reported)		
**Was adherence low?**		
**Was adherence not reported or low and could have affected outcomes?**		
**Was adherence information analyzed?**		
**What method was used?**		
**Were efficacy analyses preregistered?**		
**What assumptions are required for that method to study efficacy?**		
**Did the article report evidence that the assumptions were met?**		
**SUTVA**		
**Positivity**		
**Consistency** (treatment definition)		
**Consistency** (adherence definition)		
**Exclusion restriction**		
**Strong Monotonicity**		
**Ignorability**		
**Conditional Independence of Adherence and Outcomes**		
**Overall Notes**		
**Cochrane CDPLG**		**Notes**
**Data form completed date** (dd/mm/yyyy)		
**Study author contact details**		
Methods	**Descriptions as stated in report/paper**	**Location in text or source** (pg & ¶/fig/table/other)
Aim of study (e.g. efficacy, equivalence, pragmatic)		
Design (e.g. parallel, crossover, non-RCT)		
Unit of allocation (by individuals, cluster/ groups or body parts)		
Participants	**Descriptions as stated in report/paper**	**Location in text or source** (pg & ¶/fig/table/other)
**Inclusion criteria**		
**Exclusion criteria**		
**Total no. randomised** (or total pop. at start of study for NRCTs)		
**Clusters** (if applicable, no., type, no. people per cluster)		
**Withdrawals and exclusions** (if not provided below by outcome)		
Intervention Groups	**Descriptions as stated in report/paper**	**Location in text or source** (pg & ¶/fig/table/other)
**Group name**		
**No. randomised to group** (specify whether no. people or clusters)		
**Timing** (e.g. frequency, duration of each episode)		
**Co-interventions**		
**Integrity of delivery**		
**Compliance**		
Intervention Groups	**Descriptions as stated in report/paper**	**Location in text or source** (pg & ¶/fig/table/other)
**Group name**		
**No. randomised to group** (specify whether no. people or clusters)		
**Timing** (e.g. frequency, duration of each episode)		
**Co-interventions**		
**Integrity of delivery**		
**Compliance**		
Outcomes	**Descriptions as stated in report/paper**	**Location in text or source** (pg & ¶/fig/table/other)
**Outcome name**		
**Outcome definition** (with diagnostic criteria if relevant)(include name, time, and analysis method)		
**Imputation of missing data** (e.g. assumptions made for ITT analysis)		
**Power** (e.g. power & sample size calculation, level of power achieved)		
**Risk of Bias** (Based on Cochrane RoB Tool 2 and ROBINS-I)		Link to RoB Algorithm
**Primary Analysis RoB Assessment**		Notes
**Is the primary analysis evaluating effectiveness or efficacy?**		
**Comparator**		
**Outcome Being Assessed**		
**Specify the Numerical Result Being Assessed**		
**Bias due to randomization (RCT only)**		Notes
1.1 Was the allocation sequence random?		
1.2. Was the allocation sequence concealed until participants were enrolled and assigned to interventions?		
1.3. Did baseline differences between intervention groups suggest a problem with the randomization process?		
**Bias due to confounding**		Notes
1.1 Is there potential for confounding of the effect of intervention in this study?		
1.2. If Y/PY to 1.1 Was the analysis based on splitting participants' follow up time according to intervention received?		
1.3. If Y/PY to 1.2 Were intervention discontinuations or switches likely to be related to factors that are prognostic for the outcome?		
**Questions relating to baseline confounding only**		
1.4. If Y/PY to 1.1 Did the authors use an appropriate analysis method that controlled for all the important confounding domains?		
1.5. If Y/PY to 1.4: Were confounding domains that were controlled for measured validly and reliably by the variables available in this study?		
1.6. Did the authors control for any post-intervention variables that could have been affected by the intervention?		
**Questions relating to baseline and time-varying confounding**		
1.7. If Y/PY to 1.3 Did the authors use an appropriate analysis method that controlled for all the important confounding domains and for time-varying confounding?		
1.8. If Y/PY to 1.7: Were confounding domains that were controlled for measured validly and reliably by the variables available in this study?		
**Bias in selection of participants into the study (Not evaluating for analyses of effectiveness)**		Notes
2.1. Was selection of participants into the analysis based on participant characteristics observed after the start of intervention?		
2.2. If Y/PY to 2.1: Were the post-intervention variables that influenced selection likely to be associated with intervention?		
2.3 If Y/PY to 2.2: Were the post-intervention variables that influenced selection likely to be influenced by the outcome or a cause of the outcome?		

2.4. Do start of follow-up and start of intervention coincide for most participants?		
2.5. If Y/PY to 2.2 and 2.3, or N/PN to 2.4: Were adjustment techniques used that are likely to correct for the presence of selection biases?		
**Bias in classification of interventions**		Notes
3.1 Were intervention groups clearly defined? Was the definition of adherence clearly defined?		
3.2 Was the information used to define intervention groups recorded at the start of the intervention?		
3.3 Could classification of intervention status have been affected by knowledge of the outcome or risk of the outcome?		
**Bias due to deviations from intended interventions**		Notes
**ROBINS-I**		
4.1. Were there deviations from the intended intervention beyond what would be expected in usual practice?		
4.2. If Y/PY to 4.1: Were these deviations from intended intervention unbalanced between groups and likely to have affected the outcome?		
4.3. Were important co-interventions balanced across intervention groups?		
4.4. Was the intervention implemented successfully for most participants?		
4.5. Did study participants adhere to the assigned intervention regimen?		
4.6. If N/PN to 4.3, 4.4 or 4.5: Was an appropriate analysis used to estimate the effect of starting and adhering to the intervention?		
Risk of bias: Assignment to intervention	Low / Moderate / Serious / Critical / NI	
Risk of bias: Starting and adhering to intervention	Low / Moderate / Serious / Critical / NI	
**Risk of bias judgement**	Low / Moderate / Serious / Critical / NI	
**RoB Tool (Risk of bias due to deviations from the intended interventions (effect of adhering to intervention))**		
2.1. Were participants aware of their assigned intervention during the trial?		
2.2. Were carers and people delivering the interventions aware of participants' assigned intervention during the trial?		
2.3. If Y/PY/NI to 2.1 or 2.2: Were important non protocol interventions balanced across intervention groups?		
2.4. [If applicable:] Were there failures in implementing the intervention that could have affected the outcome?		
2.5. [If applicable:] Was there non-adherence to the assigned intervention regimen that could have affected participants' outcomes?		
2.6. If N/PN/NI to 2.3, or Y/PY/NI to 2.4 or 2.5: Was an appropriate analysis used to estimate the effect of adhering to the intervention?		
**Risk of bias judgement**	Low/High/Some Concerns	
**Bias due to missing data**		Notes
5.1 Were outcome data available for all, or nearly all, participants?		
5.2 Were participants excluded due to missing data on intervention status?		
5.3 Were participants excluded due to missing data on other variables needed for the analysis?		
5.4 If PN/N to 5.1, or Y/PY to 5.2 or 5.3: Are the proportion of participants and reasons for missing data similar across interventions?		
5.5 If PN/N to 5.1, or Y/PY to 5.2 or 5.3: Is there evidence that results were robust to the presence of missing data?		
**Bias in measurement of outcomes**		Notes
6.1 Could the outcome measure have been influenced by knowledge of the intervention received?		
6.2 Were outcome assessors aware of the intervention received by study participants? (blinding of assessors)		
6.3 Were the methods of outcome assessment comparable across intervention groups?		
6.4 Were any systematic errors in measurement of the outcome related to intervention received?		
**Bias in selection of the reported result**		Notes
Were the data that produced this result analysed in accordance with a pre-specified analysis plan that was finalized before unblinded outcome data were available for analysis?		
Is the reported effect estimate likely to be selected, on the basis of the results, from...		
7.1. ... multiple outcome measurements within the outcome domain?		
7.2 ... multiple analyses of the intervention-outcome relationship?		
7.3 ... different subgroups?		
**Secondary Analysis RoB Assessment**		Notes
**Is the seconday analysis evaluating effectiveness or efficacy?**		
**Comparator**		
**Outcome Being Assessed**		
**Specify the Numerical Result Being Assessed**		
**Bias due to randomization (See primary analysis section)**		Notes
**Bias due to confounding**		Notes
1.1 Is there potential for confounding of the effect of intervention in this study?		
1.2. If Y/PY to 1.1 Was the analysis based on splitting participants' follow up time according to intervention received?		
1.3. If Y/PY to 1.2 Were intervention discontinuations or switches likely to be related to factors that are prognostic for the outcome?		
**Questions relating to baseline confounding only**		
1.4. If Y/PY to 1.1 Did the authors use an appropriate analysis method that controlled for all the important confounding domains?		
1.5. If Y/PY to 1.4: Were confounding domains that were controlled for measured validly and reliably by the variables available in this study?		
1.6. Did the authors control for any post-intervention variables that could have been affected by the intervention?		
**Questions relating to baseline and time-varying confounding**		
1.7. If Y/PY to 1.3 Did the authors use an appropriate analysis method that controlled for all the important confounding domains and for time-varying confounding?		
1.8. If Y/PY to 1.7: Were confounding domains that were controlled for measured validly and reliably by the variables available in this study?		
**Bias in selection of participants into the study (Not evaluating for analyses of effectiveness)**		Notes
2.1. Was selection of participants into the analysis based on participant characteristics observed after the start of intervention?		
2.2. If Y/PY to 2.1: Were the post-intervention variables that influenced selection likely to be associated with intervention?		
2.3 If Y/PY to 2.2: Were the post-intervention variables that influenced selection likely to be influenced by the outcome or a cause of the outcome?		
2.4. Do start of follow-up and start of intervention coincide for most participants?		
2.5. If Y/PY to 2.2 and 2.3, or N/PN to 2.4: Were adjustment techniques used that are likely to correct for the presence of selection biases?		
**Bias in classification of interventions**		Notes
3.1 Were intervention groups clearly defined?		
3.2 Was the information used to define intervention groups recorded at the start of the intervention?		
3.3 Could classification of intervention status have been affected by knowledge of the outcome or risk of the outcome?		
**Bias due to deviations from intended interventions (See primary analysis section)**		Notes
**Bias due to missing data**		Notes

5.1 Were outcome data available for all, or nearly all, participants?		
5.2 Were participants excluded due to missing data on intervention status?		
5.3 Were participants excluded due to missing data on other variables needed for the analysis?		
5.4 If PN/N to 5.1, or Y/PY to 5.2 or 5.3: Are the proportion of participants and reasons for missing data similar across interventions?		
5.5 If PN/N to 5.1, or Y/PY to 5.2 or 5.3: Is there evidence that results were robust to the presence of missing data?		
**Bias in measurement of outcomes**		Notes
6.1 Could the outcome measure have been influenced by knowledge of the intervention received?		
6.2 Were outcome assessors aware of the intervention received by study participants? (blinding of assessors)		
6.3 Were the methods of outcome assessment comparable across intervention groups?		
6.4 Were any systematic errors in measurement of the outcome related to intervention received?		
Could adherence have been influenced by study participation?		
**Bias in selection of the reported result**		Notes
Were the data that produced this result analysed in accordance with a pre-specified analysis plan that was finalized before unblinded outcome data were available for analysis?		
Is the reported effect estimate likely to be selected, on the basis of the results, from...		
7.1. ... multiple outcome measurements within the outcome domain?		
7.2 ... multiple analyses of the intervention-outcome relationship?		
7.3 ... different subgroups?		
