# Supplementary material for: Collection and Analysis of Adherence Information for Software as a Medical Device Clinical Trials: Systematic Review
Source: JMIR Mhealth Uhealth. 2023 Nov 15;11:e46237. doi: 10.2196/46237 (PMC10687688; doi:10.2196/46237)
Supplement: Multimedia Appendix 4 [file mhealth_v11i1e46237_app4.pdf]

Study ID	QA Assignment	Device Indication for Use	Device Indication for Use subcategory	Long-term/Short-term Use	Prescribed Dosage/No Prescribed Dosage	RCT/Observational	Participants in analyses	Participants in full study	Trial Length	Study collected information about app usage?
Agarwal 2019	Lydia	Diabetes management		Long-term	No Prescribed Dosage	RCT	240	240	90	yes
Akturk 2021	Katie	Diabetes management		Long-term	No Prescribed Dosage	Observational	2637	2637	180	yes
Bull 2019	Olivia	Contraceptive		Long-term	Prescribed dose	Observational	16331	16331	365	yes
Campbell 2014	Lydia	CBT	Substance use disorder	Short-term	Prescribed dose	RCT	507	507	84	yes
Christensen 2014	Olivia	CBT	Substance use disorder	Short-term	Prescribed dose	RCT	170	170	84	yes
Dugas 2020	Lydia	Diabetes management		Long-term	No Prescribed Dosage	Observational	372	3142	90	yes
Everitt 2019a	Olivia	CBT	IBS	Short-term	Prescribed dose	RCT	372	558	365	yes
Everitt 2019b	Lydia	CBT	IBS	Short-term	Prescribed dose	RCT	372	558	365	yes
Everitt 2019c	Lydia	CBT	IBS	Short-term	Prescribed dose	RCT	204	323	730	yes
Gallen 2021	Olivia	Videogame treatment for ADHD		Short-term	Prescribed dose	Observational	28	28	28	yes
Jennings 2018	Lydia	Contraceptive		Long-term	Prescribed dose	Observational	629	718	180	yes
Jennings 2019	Olivia	Contraceptive		Long-term	Prescribed dose	Observational	718	718	365	yes
Kollins 2020	Olivia	Videogame treatment for ADHD		Short-term	Prescribed dose	RCT	348	348	28	yes
Kollins 2021	Olivia	Videogame treatment for ADHD		Short-term	Prescribed dose	Observational	206	206	28	yes
Maricich 2021a	Olivia	CBT	Substance use disorder	Short-term	Prescribed dose	Observational	3144	3144	84	yes
Maricich 2021b	Olivia	CBT	Substance use disorder	Short-term	Prescribed dose	Observational	643	643	168	yes
Maricich 2021c	Olivia	CBT	Substance use disorder	Short-term	Prescribed dose	RCT	170	170	84	yes
Pearson 2021 a	Lydia	Contraceptive		Long-term	Prescribed dose	Observational	12247	12247	365	yes
Pearson 2021 b	Olivia	Contraceptive		Long-term	Prescribed dose	Observational	5879	5879	365	yes
Perez 2019	Lydia	Irregular arrhythmia notification		Short-term	Prescribed dose	Observational	2161	419297	240	yes
Quinn 2011	Lydia	Diabetes management		Long-term	No Prescribed Dosage	RCT	142	213	365	no
Ritterband 2017	Lydia	CBT	Insomnia	Short-term	Prescribed dose	RCT	303	303	635	yes
Scherwitzl 2016	Olivia	Contraceptive		Long-term	Prescribed dose	Observational	4054		365	yes
Scherwitzl 2017	Lydia	Contraceptive		Long-term	Prescribed dose	Observational	22785	22785	365	yes
Blank Sheet										

Study collected information about adherence to app recommendations?	Was adherence information collected within the app?	Were all three facets reported?	Was adherence modified within the app?	Initiation (yes/no)	Implementation (yes/no)	Persistence (yes/no)	Was implementation low?	Was adherence not reported or low and could have affected the outcome?
NA	yes	no	NA	No	yes	No	yes	yes
NA	yes	no	NA	yes	no	No	NA	yes
yes	yes	no	NA	yes	yes	No	yes	yes
NA	yes	no	NA	no	yes	Yes	yes	yes
NA	yes	no	NA	no	no	yes	NA	yes
NA	yes	yes	NA	yes	yes	yes	yes	yes
NA	yes	no	NA	no	yes	no	yes	yes
NA	yes	yes	NA	yes	yes	yes	yes	yes
NA	yes	no	NA	no	yes	no	yes	yes
NA	yes	no	no	yes	yes	no	no	no
yes	no	no	NA	yes	no	yes	NA	no
yes	no	yes	NA	yes	yes	yes	yes	yes
NA	yes	no	no	no	yes	No	No	No
NA	yes	no	yes	yes	yes	No	yes	yes
NA	yes	yes	NA	yes	yes	yes	yes	yes
NA	yes	yes	NA	yes	yes	yes	yes	yes
NA	yes	no	NA	yes	yes	no	no	no
yes	yes	yes	NA	yes	yes	yes	yes	yes
yes	yes	yes	NA	yes	yes	yes	yes	yes
yes	yes	no	NA	yes	no	no	NA	yes
NA	NA	no	NA	no	no	no	NA	yes
NA	yes	no	NA	yes	no	yes	yes	yes
yes	yes	yes	NA	yes	yes	yes	yes	yes
yes	yes	yes	NA	yes	yes	yes	yes	yes

Cochrane RoB: Was there non-adherence to the assigned intervention regimen that could have affected participants' outcomes?	Was efficacy analysis not needed or efficacy analyzed and criteria satisfied?	Cochrane RoB: Was an appropriate analysis used to estimate the effect of adhering to the intervention?	preregistered effectiveness analysis	Effectiveness estimate	Preregistered efficacy analysis	Efficacy estimate
yes	no	no	yes	average treatment effect	no	dose-response effect
yes	no	no	NA	none	no	per-protocol effect
yes	no	no	no	average treatment effect	NA	none
yes	no	no	yes	average treatment effect	NA	none
yes	no	no	no	average treatment effect	NA	none
yes	no	no	NA	none	no	per-protocol effect
yes	yes	yes	yes	average treatment effect	yes	per-protocol effect
yes	yes	yes	yes	average treatment effect	yes	per-protocol effect
yes	no	no	no	average treatment effect	no	per-protocol effect
no	no	no	NA	none	yes	average treatment effect
no	yes	yes	yes	average treatment effect	NA	none
yes	no	no	yes	average treatment effect	yes	per-protocol effect
No	yes	yes	NA	none	yes	average treatment effect
yes	no	no	yes	average treatment effect	NA	none
yes	no	no	no	average treatment effect	no	dose-response effect
yes	no	no	no	average treatment effect	no	dose-response effect
no	yes	yes	yes	average treatment effect	NA	none
yes	no	no	no	average treatment effect	no	per-protocol effect
yes	no	no	no	average treatment effect	no	per-protocol effect
yes	no	no	yes	average treatment effect	NA	none
yes	no	no	yes	average treatment effect	NA	none
yes	no	no	yes	average treatment effect	no	per-protocol effect
yes	no	no	no	average treatment effect	NA	none
yes	no	no	no	average treatment effect	no	per-protocol effect

Study ID	(Multiple Items)

Row Labels	Count of Study ID	Count of Study ID2
**CBT**	**7**	**32%**
IBS	2	29%
Insomnia	1	14%
Substance use disorder	4	57%
**Contraceptive**	**7**	**32%**
**Diabetes management**	**4**	**18%**
**Irregular arrhythmia notification**	**1**	**5%**
**Videogame treatment for ADHD**	**3**	**14%**
**Grand Total**	**22**	**100%**

Study ID	(Multiple Items)

	Column Labels					
	Long-term		Short-term		Total Count of Study ID	Total Count of Study ID2
Row Labels	Count of Study ID	Count of Study ID2	Count of Study ID	Count of Study ID2		
No Prescribed Dosage	4	18%		0%	4	18%
Prescribed dose	7	32%	11	50%	18	82%
**Grand Total**	**11**	**50%**	**11**	**50%**	**22**	**100%**

Study ID	(Multiple Items)

Row Labels	Count of Study ID	Count of Study ID2	Average of Particip	StdDev of Participar	Average of Trial Length	StdDev of Trial Length2
Observational	14	64%	5131	7023	228	136
RCT	8	36%	286	122	298	272
**Grand Total**	**22**	**100%**	**3369**	**6019**	**253**	**193**

Study ID	(Multiple Items)

Row Labels	Count of Study ID	Count of Study ID2
no	9	50%
yes	9	50%
**Grand Total**	**18**	**100%**

Study ID	(All)

Collected info about app usag	Count of Study ID	Count of Study ID2
no	1	4%
yes	23	96%
**Grand Total**	**24**	**100%**

Study ID	(All)

Collected info about adherenc	Count of Study ID	Count of Study ID2
yes	8	100%
**Grand Total**	**8**	**100%**

Study ID	(All)

Collected info within the app	Count of Study ID	Count of Study ID2
no	2	9%
yes	21	91%
**Grand Total**	**23**	**100%**

Study ID	(All)

modified adherence in the app	Count of Study ID	Count of Study ID2
no	2	67%
yes	1	33%
**Grand Total**	**3**	**100%**

Study ID	(All)

addressed all three facets	Count of Study ID	Count of Study ID2
no	15	63%
yes	9	38%
**Grand Total**	**24**	**100%**

Measure value	(Multiple Items)		
**Row Labels**	**Count of Study ID**		
**Initiation**	**18**		17 unique
Download of app/informed consent	3	0.176	
Initial app/core completion/activity use	9	0.529	
Initiation of video in response to app alert	1	0.059	
Provided at least 20 days of data	5	0.294	
**Grand Total**	**18**		

Row Labels	Count of Study ID	Count of Study ID2
No	7	29.17%
yes	17	70.83%
**Grand Total**	**24**	**100.00%**

Study ID	(All)	
**Row Labels**	**Average of Measure value**	**StdDev of Measure value**
**Initiation**	**0.886526316**	**0.238701433**
Download of app/informed consent	1	0
Initial app/core completion/activity use	0.8407	0.278792256
Initiation of video in response to app alert	0.437	#DIV/0!
Provided at least 20 days of data	1	0
**Grand Total**	**0.886526316**	**0.238701433**

Measure value	(Multiple Items)		
**Row Labels**	**Count of Study ID**		
**Implementation**	**23**		18 unique
Completed 8 or more core modules	2	0.111	
Completed at least 4 sessions and one call	3	0.167	
Completed half of modules	2	0.111	
Completed sessions/ modules/activities	6	0.333	
Login Days	4	0.222	
Percent of logged intercourse on red days	2	0.111	
Percent of perfect use cycles	2	0.111	
Percent of pregnancies where unprotected intercc	1	0.056	
Percent of total days intercourse logged on red da	1	0.056	
**Grand Total**	**23**		

Row Labels	Count of Study ID	Count of Study ID2
no	6	25.00%
yes	18	75.00%
**Grand Total**	**24**	**100.00%**

Study ID	(All)	
**Row Labels**	**Average of Measure value**	**StdDev of Measure value2**
**Implementation**	**0.527690627**	**0.319717459**
Completed 8 or more core modules	0.865	0.091923882
Completed at least 4 sessions and one call	0.638	0.050477718
Completed half of modules	0.755	0.134350288
Completed sessions/ modules/activities	0.710018814	0.355601255
Login Days	0.432531172	0.19652185
Percent of logged intercourse on red days	0.23	0
Percent of perfect use cycles	0.168	0.101823376
Percent of pregnancies where unprotected intercc	0.51	#DIV/0!
Percent of total days intercourse logged on red da	0.02	0.014142136
**Grand Total**	**0.527690627**	**0.319717459**

Measure value	(Multiple Items)		
**Row Labels**	**Count of Study ID**		
**Persistence**	**15**		13 unique
Number of days participants used app	1	0.077	
Participants using the app at week 12	4	0.308	
Percent of participants continuing use at 1 year	6	0.462	
Completed all core modules	4	0.308	
**Grand Total**	**15**		

Row Labels	Count of Study ID	Count of Study ID2
No	11	45.83%
Yes	13	54.17%
**Grand Total**	**24**	**100.00%**

Study ID	(All)	
**Row Labels**	**Average of Measure value**	**StdDev of Measure value2**
**Persistence**	**19.6004375**	**52.69004973**
Number of days participants used app	153	31.11269837
Participants using the app at week 12	0.6375	0.165201897
Percent of participants continuing use at 1 year	0.518	0.124175682
Completed all core modules	0.48725	0.191759181
**Grand Total**	**19.6004375**	**52.69004973**

Cochrane: was there non-adherence	Count of Study ID	Count of Study ID2
no	4	17%
yes	20	83%
**Grand Total**	**24**	**100%**

Cochrane: was there an appropriate method used	Count of Study ID	Count of Study ID2
no	19	79%
yes	5	21%
**Grand Total**	**24**	**100%**

Row Labels	Count of Study ID	Count of Study ID2
average treatment ef	20	83%
none	4	17%
**Grand Total**	**24**	**100%**

Row Labels	Count of Study ID	Count of Study ID2	
**no**	**9**	**45%**	
Observational	7	78%	
RCT	2	22%	
**yes**	**11**	**55%**	
Observational	4	36%	0.363636
RCT	7	64%	0.777778
**Grand Total**	**20**	**100%**	

Row Labels	Count of Study ID	Count of Study ID2	
average treatment ef	2	8.3%	
dose-response effect	3	12.5%	
none	9	37.5%	
per-protocol effect	10	41.7%	
**Grand Total**	**24**	**100.0%**	0.625

Row Labels	Count of Study ID	Count of Study ID2	
**no**	**10**	**67%**	
Observational	7	70%	
RCT	3	30%	
**yes**	**5**	**33%**	
Observational	2	40%	0.222222
RCT	3	60%	0.5
**Grand Total**	**15**	**100%**	

Study ID	QA Assignment	Measure type	Measure name	Measure value
Agarwal 2019	Lydia	Implementation	Login Days	0.232967033
Akturk 2021	Katie	Initiation	Initial app/core completion/activity use	0.922
Bull 2019	Olivia	Initiation	Provided at least 20 days of data	1
Bull 2019	Olivia	Implementation	Percent of total days intercourse logged on red day	0.01
Bull 2019	Olivia	Implementation	Percent of total days intercourse logged on red day	0.03
Campbell 2014	Lydia	Implementation	Completed sessions/ modules/activities	0.762
Campbell 2014	Lydia	Persistence	Participants using the app at week 12	0.45
Christensen 2014	Olivia	Persistence	Participants using the app at week 12	0.8
Dugas 2020	Lydia	Initiation	Initial app/core completion/activity use	0.299
Dugas 2020	Lydia	Initiation	Initial app/core completion/activity use	0.333
Dugas 2020	Lydia	Implementation	Completed sessions/ modules/activities	0.039341917
Dugas 2020	Lydia	Implementation	Completed sessions/ modules/activities	0.354077253
Dugas 2020	Lydia	Persistence	Number of days participants used app	131
Dugas 2020	Lydia	Persistence	Number of days participants used app	175
Everitt 2019a	Olivia	Implementation	Completed at least 4 sessions and one call	0.692
Everitt 2019b	Lydia	Initiation	Initial app/core completion/activity use	0.919
Everitt 2019b	Lydia	Implementation	Completed at least 4 sessions and one call	0.592
Everitt 2019b	Lydia	Persistence	Completed all core modules	0.216
Everitt 2019c	Lydia	Implementation	Completed at least 4 sessions and one call	0.63
Gallen 2021	Olivia	Initiation	Initial app/core completion/activity use	1
Gallen 2021	Olivia	Implementation	Completed sessions/ modules/activities	1
Jennings 2018	Lydia	Initiation	Download of app/informed consent	1
Jennings 2018	Lydia	Persistence	Percent of participants continuing use at 1 year	0.69
Jennings 2019	Olivia	Initiation	Download of app/informed consent	1
Jennings 2019	Olivia	Implementation	Percent of perfect use cycles	0.24
Jennings 2019	Olivia	Persistence	Percent of participants continuing use at 1 year	0.47
Kollins 2020	Olivia	Implementation	Completed sessions/ modules/activities	0.83
Kollins 2021	Olivia	Initiation	Initial app/core completion/activity use	1
Kollins 2021	Olivia	Implementation	Completed sessions/ modules/activities	0.73
Kollins 2021	Olivia	Implementation	Completed sessions/ modules/activities	0.811
Maricich 2021a	Olivia	Initiation	Initial app/core completion/activity use	1
Maricich 2021a	Olivia	Persistence	Completed all core modules	0.49
Maricich 2021a	Olivia	Implementation	Completed half of modules	0.66
Maricich 2021a	Olivia	Implementation	Completed 8 or more core modules	0.8
Maricich 2021a	Olivia	Persistence	Participants using the app at week 12	0.55
Maricich 2021b	Olivia	Initiation	Initial app/core completion/activity use	1
Maricich 2021b	Olivia	Persistence	Completed all core modules	0.64
Maricich 2021b	Olivia	Implementation	Completed half of modules	0.85
Maricich 2021b	Olivia	Implementation	Completed 8 or more core modules	0.93
Maricich 2021b	Olivia	Persistence	Participants using the app at week 12	0.75
Maricich 2021c	Olivia	Initiation	Initial app/core completion/activity use	1
Maricich 2021c	Olivia	Implementation	Completed sessions/ modules/activities	1.153731343
Pearson 2021 a	Lydia	Initiation	Provided at least 20 days of data	1
Pearson 2021 a	Lydia	Implementation	Percent of logged intercourse on red days	0.23
Pearson 2021 a	Lydia	Implementation	Login Days	0.26981
Pearson 2021 a	Lydia	Implementation	Login Days	0.6395
Pearson 2021 a	Lydia	Persistence	Percent of participants continuing use at 1 year	0.419
Pearson 2021 b	Olivia	Initiation	Provided at least 20 days of data	1
Pearson 2021 b	Olivia	Implementation	Percent of logged intercourse on red days	0.23
Pearson 2021 b	Olivia	Implementation	Login Days	0.26511
Pearson 2021 b	Olivia	Implementation	Login Days	0.6378
Pearson 2021 b	Olivia	Persistence	Percent of participants continuing use at 1 year	0.409
Perez 2019	Lydia	Initiation	Download of app/informed consent	1
Perez 2019	Lydia	Initiation	Initiation of video in response to app alert	0.437
Quinn 2011	Lydia			

Ritterband 2017	Lydia	Initiation	Initial app/core completion/activity use	0.934
Ritterband 2017	Lydia	Persistence	Completed all core modules	0.603
Scherwitzl 2016	Olivia	Initiation	Provided at least 20 days of data	1
Scherwitzl 2016	Olivia	Implementation	Percent of pregnancies where unprotected interco	0.51
Scherwitzl 2016	Olivia	Implementation	Login Days	0.55
Scherwitzl 2016	Olivia	Persistence	Percent of participants continuing use at 1 year	0.66
Scherwitzl 2017	Lydia	Initiation	Provided at least 20 days of data	1
Scherwitzl 2017	Lydia	Implementation	Percent of perfect use cycles	0.096
Scherwitzl 2017	Lydia	Persistence	Percent of participants continuing use at 1 year	0.46

Blank Sheet
